# Stereoselective Syntheses of Cyclic Microsclerodermin Derivatives

**DOI:** 10.1002/chem.202502459

**Published:** 2025-09-26

**Authors:** Kevin Bauer, Uli Kazmaier

**Affiliations:** ^1^ Organic Chemistry Saarland University Campus C4.2 66123 Saarbrücken Germany; ^2^ Pharma Science Hub Saarland University Campus, A2.3 D‐66123 Saarbrücken Germany

**Keywords:** Arndt‐Eistert homologation, cyclic peptides, microsclerodermins, sakurai reaction, tetrahydrofurans

## Abstract

Starting from l‐xylose and d‐arabinose, six different cyclic microscleroderma derivatives were successfully obtained. Key steps of the syntheses are, on the one hand, Sakurai allylations, whose stereochemical course depends on the Lewis acid used, and on the other hand, photochemical Wolff rearrangements in the presence of complex aminofuranosides. Finally, aromatic side chains were introduced via cross metathesis.

## Graphical abstract

Six different cyclic microsclerodermin derivatives are obtained from l‐xylose and d‐arabinose via stereoselective Sakurai allylations and photochemical Arndt‐Eistert homologations.

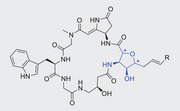

## 1 Introduction

**Figure 1:**
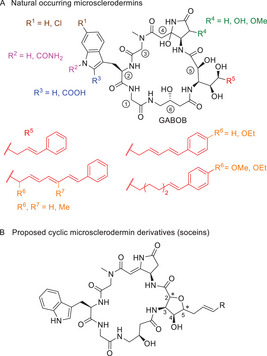
Natural occurring microsclerodermins A) and proposed cyclic derivatives B).

From 1994 until 2000, the Faulkner Group isolated the microsclerodermin A‐I from marine lithistid sponges of the *Microscleroderma* and *Theonella species*.^[^ ^1^ ^]^ Further derivatives have been isolated later on by the groups of Li^[^ ^2^ ^]^ and Matsunaga from other sponges.^[^ ^3^ ^]^ In 2008, Kunze et al. isolated the pedeins A and B from the terrestrial myxobacteria *Chondromyces pediculatus* that share the above‐mentioned basic structural motifs and the antifungal activity of the microsclerodermin family.^[^ ^4^ ^]^ Finally, in 2013, the Müller group described some more microsclerodermins from three different *genera* of terrestrial *Myxobacteria*,^[^ ^5^ ^]^ which underpins the theory that these “sponge metabolites” might, in fact, originate from microbial symbionts genetically related with myxobacteria.^[^ ^4^, ^5^ ^]^ The core motif of these cyclic peptides is a 23‐membered ring that features six amino acids ①–⑥ (Figure 1A). While glycine ①, sarcosine ③, and (*R*)‐γ‐amino‐β‐hydroxybutyric acid (GABOB) ⑥ are common to all members of the microsclerodermin family, the other three amino acids, best described as a modified tryptophan residue (R^1^–R^3^) ②, an unusual 3‐amino pyrrolidone‐4‐acetic acid (R^4^) ④, and a ω‐aromatic 3‐amino‐2,4,5‐trihydroxyacid (R^5^) ⑤, are variable units. The amino pyrrolidone unit ④ contains a hemiaminal position that readily eliminates water when treated with mild acid, forming a dehydromicrosclerodermin.

During the workup of the myxobacterial culture broth some side metabolites were isolated in such tiny amounts that only ^1^H NMR spectra could be recorded which showed differences to the so far known microsclerodermins. It was assumed that these new compounds (named soceins) might be cyclic derivatives containing a highly substituted furan ring connected to an (emininated) 3‐amino pyrrolidone‐4‐acetic acid (Figure 1B).

Most members of the microsclerodermin family including the dehydrated derivatives, show potent antifungal activity against *Candida albicans* ^[^ ^1^, ^2^, ^5^ ^]^ as well as activities toward various cancer cell lines.^[^ ^1^, ^3^, ^6^ ^]^

Taking into account these interesting biological activities, it is not surprising that numerous synthetic routes have been developed for the assembly of the unusual building blocks, especially for the synthesis of the polyhydroxylated β‐amino acids^[^ ^7^ ^]^ and the amino pyrrolidone unit.^[^ ^7^, ^8^ ^]^ The first total synthesis of a member of the microsclerodermin family, microsclerodermin E, was reported by Ma and Zhu,^[^ ^7b^ ^]^ while Donohoe et al reported the syntheses of dehydromicrosclerodermin B and microsclerodermin J.^[^ ^7a,g^ ^]^

## 2 Results and Discussion

Since our research group has been working for years on the synthesis of natural products, in particular peptides^[^ ^9^ ^]^ and peptide‐polyketide conjugates,^[^ ^10^ ^]^ we became interested in the synthesis of these unusual structural features. Although substituted tetrahydrofurans are widespread found in natural products,^[^ ^11^ ^]^ derivatives with a carboxyl function in the 2‐position, as postulated for the cyclic microsclerodermines are extremely rare. Some of the few examples are the two marine natural products formosalide (2,5‐disubstituted)^[^ ^12^ ^]^ and chagosenine (2,3,5‐trisubstituted),^[^ ^13^ ^]^ although it is not yet clear how the 2‐tetrahydrofuran carboxylic acids are biosynthetically generated. However, a highly substituted tetrahydrofuran ring system, as postulated for the cyclic microsclerodermin derivative, is unique and a serious challenge for synthetic chemists, which sparked our interest in the synthesis of this hypothetical compound. We hoped to be able to confirm the postulated structure and to get enough material to investigate the biological properties of this compound.^[^ ^14^ ^]^

Assuming that the tetrahydrofuran ring somehow is formed from the polyhydroxylated β‐amino acid, it can probably be assumed that the configuration of the 3‐NH group and the 4‐OH group is identical to that in the linear microsclerodermines. However, since the biosynthetic cyclization mode is not known, no statement can be made about the configurations at positions 2 and 5. We therefore developed a synthetic concept that would give us access to all four stereoisomers. The idea was to cyclize the molecule between the glycine and the GABOB and to introduce a simplified side chain on the tetrahydrofuran ring at the end via metathesis.

**Scheme 1:**
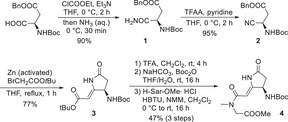
Synthesis of the amino pyrrolidone building block **4**.

For the synthesis of the amino pyrrolidone building block we decided to follow the route described by Donohoe^[^ ^7a^ ^]^ which features a Blaise reaction as a crucial step (Scheme 1). Activation of (*R*)‐Boc‐Asp(OBn)‐OH as a mixed anhydride and quenching with aqueous ammonia gave primary amide **1**, which was dehydrated with trifluoroacetic anhydride (TFAA) and pyridine to nitrile **2**. The Blaise reaction with *tert*‐butyl bromoacetate and subsequent cyclization afforded the amino pyrrolidones **3** in good yields. In the next step, both protecting groups were cleaved simultaneously with TFA, and the amine was protected again as Boc‐carbamate. Due to the challenging purification of the free carboxylic acid, the crude acid was directly coupled with the hydrochloride salt of sarcosine methyl ester to enantiomerically pure **4** ^[^ ^15^ ^]^ in an acceptable yield of 47% yield over 3 steps.

Before continuing with the synthesis of the stereoisomeric tetrahydrofuran rings we first focused on the other unusual building block (*R*)‐GABOB. Even though several syntheses already exist,^[^ ^16^ ^]^ a new route starting from l‐isoserine (Ise) was investigated. An Arndt‐Eistert homologation of Ise should give access to (*R*)‐GABOB in a straightforward fashion, with the benefit of several substitution options by using different nucleophiles in the Wolff rearrangement. Ideally, using suitable substituted tetrahydrofuran building blocks as amine nucleophiles would avoid potential reactivity issues of the sterically hindered amines due to the high reactivity and the low sterical demand of the ketene formed in the Wolff rearrangement.

**Scheme 2:**
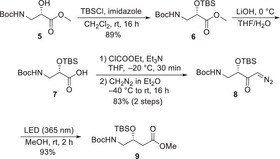
Synthesis and reaction of diazoketones **8**.

Starting from known Boc‐Ise‐OMe **5** ^[^ ^17^ ^]^ protection of the secondary hydroxy functionality with TBS‐Cl gave the protected isoserine **6** (Scheme 2). The methyl ester was cleaved under standard conditions and immediately after saponification, the carboxylic acids **7** was activated as mixed anhydrides and, by treatment with freshly prepared diazomethane^[^ ^18^ ^]^ converted to the diazoketones **8**. The ester was found to improve the storability of the compound because the free carboxylic acid **7** suffers from autocatalyzed TBS‐deprotection within hours. To investigate the reactivity of **8** we first tested a silver‐catalyzed Wolff rearrangement with methanol which provided the corresponding homologated methyl ester **9** in 73% yield. Alternatively, a variety of light sources, from UV to visible light, can be used for the activation of diazoketones.^[^ ^19^ ^]^ And indeed, irradiation of the diazoketone **8** in methanol, with an 18 W blue LED (365 nm), gave the desired (*R*)‐GABOB derivative **9** in excellent yield.

**Scheme 3:**
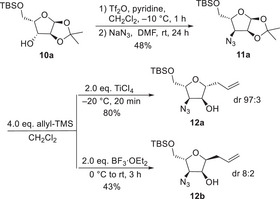
Synthesis and Sakurai allylation of azidofuranoside **11a**.

Getting access to the different stereoisomers of the highly functionalized THF‐ring a chiral pool approach utilizing the stereocenters of monosaccharides was examined (Scheme 3). Introduction of the allyl moiety was planned using a Hosomi‐Sakurai allylation, which is especially suited to introduce allyl functionality onto carbohydrates.^[^ ^20^ ^]^ In 1976, Hosomi and Sakurai reported a protocol for the allylation of aldehydes and ketones using allyl trimethylsilane and TiCl_4_ or BF_3_∙OEt_2_ as Lewis acids,^[^ ^21^ ^]^ which they expanded shortly after on the allylation of acetals.^[^ ^22^ ^]^ The first use of the Hosomi‐Sakurai allylation on glycosyl acetals was reported by Kazikowski and Sorgi in 1982.^[^ ^23^ ^]^ The stereochemical outcome of this reactions were intensively studied by the groups of Martin,^[^ ^24^ ^]^ Reissig,^[^ ^25^ ^]^ Woerpel,^[^ ^26^ ^]^ Tellado^[^ ^27^ ^]^ and Sartillo‐Piscil.^[^ ^28^ ^]^

We started our investigations with silyl protected acetonide **10a**, which is easily accessible from l‐xylose.^[^ ^29^ ^]^ Activation of the OH‐functionality and substitution by azide provided the desired azidofuranoside **11a** (Scheme 3). The Sakurai allylation using Tellado's protocol^[^ ^27^ ^]^ worked well with TiCl_4_, affording the C‐furanoside **12a** in good yield and excellent diastereoselectivity. Surprisingly, the allylation with BF_3_∙OEt_2_ gave the isomeric product **12b** with inverted configuration at the allyl substituent and a significantly lower yield and selectivity. Such a stereochemical inversion by changing the Lewis acid was, to the best of our knowledge, not reported so far, neither by Tellado et al.^[^ ^27^ ^]^ nor the other groups working in this field.^[^ ^20^ ^]^

To figure out the configuration of the new formed stereogenic center we subjected both diastereomers to ozonolysis. **12a**, obtained in the TiCl_4_‐catalzed reaction gave rise to the corresponding aldehyde, while ozonolysis of **12b** resulted in a diastereomeric mixture of the corresponding hemiacetals, clearly indicating that **12b** is the *cis*‐configured product, since only this one can spontaneously cyclize to the hemiacetal.

**Scheme 4:**
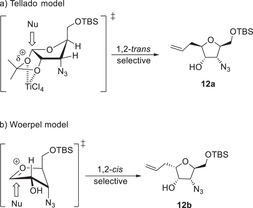
Stereochemical models for the Sakurai allylation of **11a**.

Stereochemical models for the allylation might explain the outcome of the reaction (Scheme 4). The first model (a) by Tellado et al. shows an S_N_2‐type mechanism via Lewis acid activation of the 1,2‐*O*‐isopropylidene group, which directs the nucleophilic attack to the *exo*‐face of the bicyclic system.^[^ ^27^ ^]^ On the other hand, if the reaction proceeds through an oxonium ion intermediate, the stereochemical outcome strongly depends on the furanoside substituents, according to Woerpel's model (b).^[^ ^26a,c,d^ ^]^

Assuming the azide does not stabilize the transition state via electrostatic interaction like an *O*‐substituent and also does not exert a large steric influence, only the oxy‐substituent in the 2‐position should play a significant role for the stereochemical outcome of the allylation. This would result in the transition state with the C‐2 substituent in pseudoequatorial position, maximizing the hyperconjugative effect of the σ_C–H_ orbital and the vacant orbital of the oxonium ion. The Tellado group reported a 1,2‐*trans* selectivity in all their experiments, independent of using BF_3_∙OEt_2_ or TiCl_4_, which would follow model (a).^[^ ^27^ ^]^ The 1,2‐*cis* selectivity in the BF_3_‐allylation shown in this work strongly suggests an oxonium ion intermediate according to model (b) because an *endo*‐facial attack of a bicyclic system like model (a) is unlikely. Furthermore, the BF_3_‐allylation in this study showed comparable stereoselectivity to the *C*‐allylation of a similar furanoside with no substitution in C‐3 position, reported in the synthesis of Hagen's gland lactones.^[^ ^28a^ ^]^

**Scheme 5:**
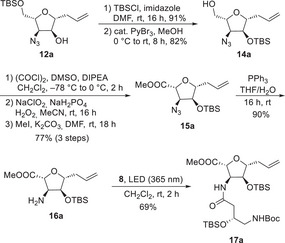
Synthesis of tetrahydrofuran derivative **17a**.

Due to the high yield and diastereoselectivity, the TiCl_4_‐allylation product **12a** was further transformed to finish the first diastereomer of the desired tetrahydrofurans (Scheme 5). First, the secondary alcohol of **12a** was TBS‐protected, and the primary TBS‐ether of **13a** was selectively cleaved with in situ generated HBr according to a protocol by Martinez‐Solorio and Jennings.^[^ ^30^ ^]^ Initial attempts with an alternative orthogonal protecting group (PMB) for the secondary alcohol resulted in low yields in the protection step and were discontinued. After the direct oxidation of **14a** with TEMPO gave only unsatisfactory results, a two‐step sequence of Swern and Pinnick oxidation was used. Esterification with iodomethane in DMF provided **15a** in 77% yield over 3 steps. The reaction sequence was concluded with a Staudinger reduction of the azide to give the *C*‐furanoside **16a**. In order to figure out whether such highly substituted tetrahydrofurans can still be coupled at all, **16a** was reacted with diazoketone **8** under the previously optimized conditions. By irradiation at 365 nm, the desired dipeptide **17a** could be obtained in good yield.

**Scheme 6:**
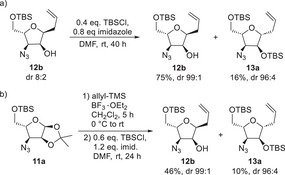
Silylation of allyl furanosides.

After we had completed the first stereoisomeric building block **16a**, we turned to the second diastereomer **12b**. Unfortunately, the stereoisomers formed during the Sakurai reaction could not be chromatographically separated from each other. Therefore, we tried to further convert the diastereomeric mixture analogous to **12a**, hoping to be able to separate the stereoisomers at a later stage. In contrast to **12a**, the reaction of **12b** with TBSCl showed hardly any conversion by TLC, even if an excess was used, or the more reactive TBSOTf. A more detailed analysis of the reaction products showed that the minor stereoisomer **12a** reacted cleanly to the silyl ether **13a** as described, but that the *cis* isomer **12b** hardly reacted at all and accumulated in enantiomerically pure form (Scheme 6a). Silyl ether **13a** was also obtained in high diastereoselectivity and could thus be used for the synthesis of **16a** as decribed above. This sequence could be further improved by using a “one‐pot” procedure of Sakurai allylation and subsequent TBS‐protection of the crude mixture (Scheme 6b). After some optimizations, this procedure improved the yield to 56% over 2 steps, affording the two C‐furanoside building blocks **12b** and **13a** in high diastereomeric purities.

Since we were not able to silylate the secondary alcohol of **12b**, we tried a selective oxidation of the primary alcohol with sterically demanding oxidizing agents, such as Dess Martin periodinane, after splitting off the TBS protection group. Unfortunately, this approach was just as unsuccessful as TEMPO‐based oxidations.^[^ ^31^ ^]^ These reactions did not proceed selectively, and the isolation of the highly polar product and side products was tedious.

**Scheme 7:**
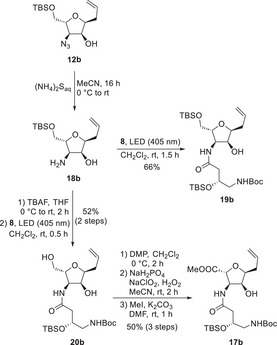
Synthesis of tetrahydrofuran derivatives **17b**.

To avoid these issues and to improve the selectivity of the oxidation by further increasing the steric hindrance at the secondary alcohol, the amine reduction and reaction with diazoketone **8** was performed prior to the oxidation step (Scheme 7). In this case, the azide was reduced with ammonium sulfide by a protocol described by Suna et al.^[^ ^32^, ^33^ ^]^ Overall, this biphasic procedure allowed easier isolation of the desired amino alcohol **18b** in better yield and purity compared to the Staudinger reduction with PPh_3_. The first tests of the Wolff rearrangement with **18b** afforded the dipeptide **19b** in 66% yield, while the best results were obtained irradiating the reaction mixture at 405 nm.

To avoid exchanging the protecting group or selective mono‐TBS‐deprotection, another attempt with the crude amino alcohol **18b** after TBS‐deprotection was performed. Reaction with **8** provided amide **20b** in acceptable yield, considering the three competing nucleophilic centers. Selective oxidation of the primary alcohol was achieved with the sterically demanding Dess Martin periodinane (DMP)^[^ ^34^, ^35^ ^]^ along with only 10% additional oxidation of the secondary alcohol. The aldehyde was further oxidized under Pinnick conditions, followed by methylation of the intermediary carboxylic acid to simplify the purification process of the desired dipeptide **17b**.

**Scheme 8:**
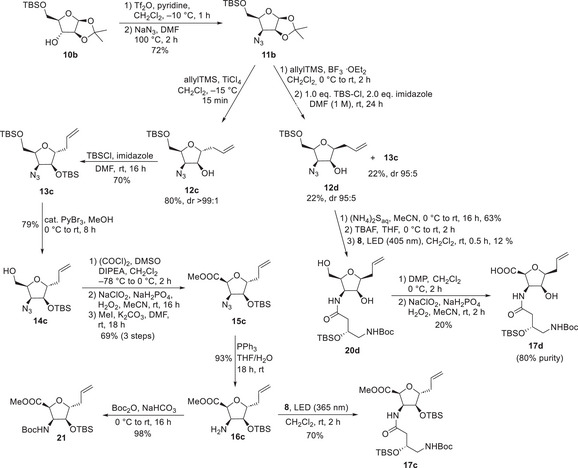
Synthesis of tetrahydrofuran derivatives **17c** and **17 d**.

A third and fourth diastereomer should be accessible by a similar route, using d‐arabinose as the starting point (Scheme 8). Ketale **10b** was obtained in two steps according to the literature.^[^ ^36^ ^]^ Due to the inverted C‐2 stereocenter and the resulting increase in sterical hindrance, the concave‐sided attack is even more disfavored than in case of the l‐xylose derivative **10a**. Surprisingly, the azide substitution protocol yielded the desired azide **11b** in good yield after increasing the temperature to 100 °C.

The absence of the antiperiplanar hydrogen atom in the triflate of **10b** allowed for the increase in temperature without competing E_2_‐elimination. The Sakurai allylation with TiCl_4_ gave the C‐furanoside **12c** in excellent yield and diastereoselectivity. The postulated 1,2‐*trans* stereochemistry, identical to the previously performed Sakurai allylation, was again confirmed by ozonolysis. In this case, the allylation with BF_3_∙OEt_2_ of **11b** gave C‐furanosides **12c and 12d** in low yield (26%) and as a 1:1 diastereomeric mixture. The significantly lower selectivity in comparison to the l‐xylose route (Scheme 3) is most likely a direct result of the high sterical demand of the all‐*cis* diastereomer. After these unsatisfying results, the BF_3_‐catalyzed Sakurai allylation of **11b** was reevaluated under the previously optimized “one‐pot” conditions. Finally, both diastereomeric products **12d** and **13c** were obtained in 22% yield each with reasonable diastereomeric purities, confirming the earlier mentioned dr of 1:1 for the allylation step.

**13c** was also obtained from **12c** using the previously optimized silylation conditions. Selective deprotection of the primary OH‐functionality provided the primary alcohol **14c** which was converted in a three‐step protocol of oxidation and methylation into the corresponding methyl ester **15c** in 69% overall yield. Staudinger reduction of the azide and photochemical reaction with diazoketone **8** provided the third dipeptide building block **17c**. In this case, the Staudinger reduction was additionally performed in the presence of Boc_2_O to obtain Boc‐protected amine **21** as a storable intermediate in almost quantitative yield.

Although, the BF_3_‐catalyzed allylation of **11b** provided the all *cis*‐isomer **12d** only in low yields, we finally obtained **12d** in sufficient amounts to continue our synthetic route to the desired forth dipeptide **17d**. The azide of **12d** was reduced with ammonium sulfide, followed by TBS‐deprotection with TBAF. The resulting crude amino alcohol was used in a Wolff rearrangement of diazoketone **8**, which yielded the amide **20d** in only 12% yield.

Reaction control via LC‐MS showed that the conversion of the amine was rather low, but the diazoketone was completely consumed, mainly due to [2 + 2]‐cycloaddition.^[^ ^37^ ^]^ Indeed, the reactivity of the amino alcohol seems to be much lower compared to the other stereoisomers due to the increased sterical hindrance in the all‐*cis* configuration. The selective oxidation with DMP was tested with the small amount of isolated **20d**. A low conversion rate and insufficient selectivity toward the primary alcohol of **20d** further complicated the progress on the all‐*cis* diastereomer. Pinnick oxidation of the crude aldehyde yielded the carboxylic acid **17d** in only 20% with a low purity of around 80%.Due to several consecutive low‐yielding steps, the remaining material proved insufficient to complete the synthesis of the corresponding cyclic microsclerodermine derivative. We decided to continue with the other three stereoisomers **17a**‐**17c** to figure out if we can verify the proposed tetrahydrofuran motif at all.

**Scheme 9:**
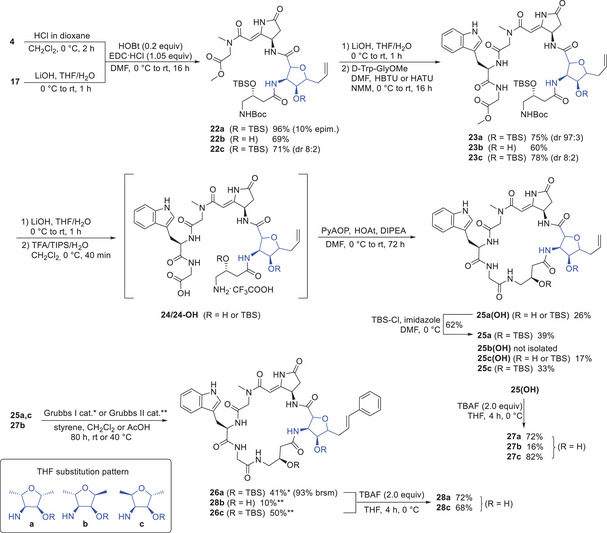
Synthesis of cyclic mycoplanecin derivatives **27** and **28**.

Therefore, our attention was turned toward the coupling of these building blocks to afford the cyclic microsclerodermine target structures (Scheme 9). The dipeptides **17a**‐**17c** were saponified using LiOH in THF and the pyrrolidone fragment **4** was *N*‐deprotected with HCl in dioxane. A first standard peptide coupling of the deprotected components with EDC/HOBt provided tetrapeptide **22a** in 70% yield, containing 25% of an epimer. Different conditions and peptide coupling agents (HATU, PyAOP, and COMU) were tested to reduce the epimerization rate. None of the other coupling reagents decreased the epimerization rate below 13%. On the contrary, the COMU‐mediated coupling increased the epimerization significantly to 33%. Best results in the EDC/HOBt coupling were obtained by using an excess of the amine component (1.5 equiv). Under these conditions the epimerization rate could be reduced to 10% and the overall yield could be increased to 96%. Saponification and subsequent HBTU‐mediated coupling with dipeptide D‐Trp‐Gly‐OMe provided hexapeptide **23a** in 75% yield and a diastereomeric ratio of 97:3. The minor epimer from the last step could be separated by reversed‐phase column chromatography. Based upon previous experiences of acidic deprotections of larger peptides, a cleavage cocktail of TFA/TIPS was used to achieve Boc‐deprotection to the TFA‐salt **24a**. Even under these rather mild conditions, one of the TBS‐groups was partially cleaved. Therefore, a mixture of the bis‐TBS‐protected peptide **24a** and the mono‐TBS‐protected peptide **24aOH** (ratio 6:4) was used in the subsequent macrocyclization. A high dilution protocol using PyAOP,^[^ ^7^, ^16^ ^]^ gave cyclic dehydromicrosclerodermine precursor **25a** and the mono TBS‐deprotected derivative **25a(OH)** in a total yield of 65% over 3 steps. Transformation of **25a(OH)** to the desired bis‐protected **25a** was achieved by treatment with TBS‐Cl and imidazole.

As a final step, the incorporation of an aromatic side chain was required. Hence, an olefin cross‐metathesis of **25a** with styrene was planned, aiming to introduce the simplified side‐chain resembling that of the microsclerodermins C, D, and L. Donohoe and co‐workers used 4‐methoxy styrene and a first‐generation Grubbs catalyst for a late‐stage olefination during their synthesis of dehydromicrosclerodermin B.^[^ ^7a^ ^]^ First experiments were therefore also carried out using Grubbs I catalyst in CH_2_Cl_2_ at room temperature which resulted in around 50% conversion. Addition of more Grubbs I catalyst barely improved the conversion. Therefore, the reaction was stopped, the desired product **26a** was isolated in 41% yield, and 52% of the starting material **25a** was recovered. Due to the low conversion of the metathesis reaction, the recovered starting material was also reacted with second‐generation Grubbs‐, and Hoveyda‐Grubbs‐catalyst. Grubbs II showed 87% conversion, and although the Hoveyda‐Grubbs catalyst provided similar results, more side products were formed with this catalyst. Thus, the second‐generation Grubbs catalyst was used in the further modifications of the other stereosiomers. Deprotection of **25a(OH)** and **26a** by treatment with TBAF afforded the two cyclic microsclerodermine derivatives, **27a** and **28a**.

The two remaining diastereomeric building blocks (**17b**, **17c**) were converted similarly. The EDC/HOBt‐coupling of **17b** with deprotected **4** proceeded also with 9% epimerization, but provided the tetrapeptide **22b** in 69% yield as a single diastereomer after preparative HPLC. The hexapeptide **23b** was isolated in 60% yield after HATU coupling with D‐Trp‐Gly‐OMe.

The cyclization of this hexapeptide however proved more problematic. Saponification and acidic deprotection proceeded cleanly, and no TBS‐cleavage was observed. The cyclization with PyAOP/HOAt showed complete conversion to the desired macrolactam as monitored by LC‐MS, however, separation of the cyclized product **25b(OH)** from the side products of the coupling reagents, like the tripyrrolidinyl phosphine oxide, turned out to be impossible. Numerous attempts using column chromatography and preparative HPLC proved unsuccessful. Consequently, a subsequent TBS‐deprotection to **27b** was carried out to modify the polarity and, ultimately, the retention time of the product.

Due to the insolubility of the hexapeptide **27b** in toluene and CH_2_Cl_2_, the Grubbs‐metathesis was conducted in AcOH, a solvent for cross‐metathesis with remarkable reaction kinetics.^[^ ^38^ ^]^ The cyclic dehydromicrosclerodermine derivative **28b** could be isolated in only 10% yield even after several additions of catalyst and increasing the temperature to 40 °C.

Starting with **17c**, saponification followed by EDC/HOBt coupling led to the tetrapeptide **22c**. Even though the previously optimized base‐free carbodiimide coupling was used, the reaction resulted in 20% epimerization. The diastereomeric mixture of **22c** was saponified and coupled with dipeptide D‐Trp‐Gly‐OMe using HATU. Utilizing the same sequence of saponification, acidic deprotection, and high‐dilution macrocyclization on the isomeric mixture of **24c** afforded **25c** in 33% yield along with 17% of mono‐TBS‐protected derivative **25c(OH)**. Here, the stereoisomers could be separated via preparative HPLC. The olefin cross‐metathesis of **25c** with the second‐generation Grubbs catalyst only marginally improved the yield to 50%. After removal of the TBS‐protecting groups with TBAF, derivative **28c** was isolated in good yield. Deprotection of the mono‐protected cyclopeptide **25c(OH)** under identical conditions gave access to **27c** in 82% yield.

All dehydrosocein derivatives **27** and **28** were investigated regarding their biological activity. Unfortunately, they were complete inactive against yeasts (*C. albicans*), fungi (*C. neoformans*, *P. anomala)*, Gram‐negative (*E.coli* WT) as well as Gram‐positive bacteria (*S. aureus* Newman, *M. smegmatis*) and tumor cell lines (HepG2). The NMR spectra of the compounds also did not match the ^1^H NMR of the originally isolated sample. Although interesting chemistry could be developed in this project, the question arises whether such postulated cyclic microsclerodermines really exist.

## 3 Conclusions

In conclusion, starting from the commercially available carbohydrates l‐xylose and d‐arabinose six different cyclic microsclerodermin derivatives containing a highly substituted tetrahydrofuran ring systems could successfully be synthesized. Only the all‐*cis*‐configured derivative provided serious problems due to steric reasons. The stereochemical outcome of the Sakurai allylations, one of the key steps showed an interesting dependence of the Lewis acid used. An excellent 4,5‐*anti*‐selectivity was observed using TiCl_4_, while with BF_3_ as Lewis acid preferentially the 4,5‐*syn*‐product could be obtained. A photochemical Wolff rearrangement was used to connect the unusual (*R*)‐γ‐amino‐β‐hydroxybutyric acid directly with the highly substituted 3‐aminotetrahydrofuran building blocks. Finally, aromatic side chains were introduced via cross metathesis. Unfortunately, none of the new microsclerodermin derivatives showed any biological activity.

## 4 Experimental Section

### General remarks

All reactions were carried out in oven‐dried glassware under a nitrogen atmosphere. Anhydrous THF was prepared by distillation over sodium/benzophenone. Other anhydrous solvents were purchased from Acros Organics and Thermo Scientific. Petroleum ether (PE), pentane, and ethyl acetate (EtOAc) were distilled prior use. The products were purified by flash chromatography on silica gel (0.063–0.2 mm). Mixtures of EtOAc and PE were generally used as eluents. Analytical TLC was performed on pre‐coated silica gel plates (Macherey–Nagel, PolygramS SIL G/UV254). Visualization was accomplished with UV‐light and KMnO4, cerium‐molybdate, or ninhydrin solution. For column chromatography, silica gel 60 M 40 – 63 µm by Macherey Nagel was used. Automated flash column chromatography was done on a Büchi Pure C815 Flash with Teledyne Isco RediSep R_f_ cartridges. Automated reversed‐phase column chromatography was done on a Büchi Reveleris Prep with Büchi FlashPure Select C18 (spherical) cartridges or Kinesis Telos C18 cartridges. Preparative HPLC was done with a Büchi Reveleris Prep with a Phenomenex Luna (C18, 5 µm, 21.2 × 250 mm). Melting points were measured in open glass capillaries on an M3000 from *Krüss Optronic GmbH*. Specific optical rotation was measured on a P‐8000‐T polarimeter by *A. Krüss Optronic GmbH* with a PT80 thermostat by *A. Krüss Optronic GmbH* at 20 °C (λ  =  589 nm). Photochemical reactions were conducted in a EvoluChem PhotoRedOx Box by *HepatoChem* using 18 W LED‐lamps (365 nm, 405 nm and 450 nm). NMR spectra were measured on a Bruker Avance II 400 (400 MHz, 5 mm BBO Probe, 298 K), a Bruker Avance I 500 (500 MHz, 5 mm TCI Probe, 298 K), or a Bruker Avance Neo 500 (500 MHz, 5 mm TCI Prodigy CryoProbe, 298 K). The spectral data were analyzed with MestReNova 14.2 from MestreLabReasearch S.L. Chemical shifts are reported in ppm relative to TMS, and CHCl_3_ was used as the internal standard. Assignments were done using 2D measurements like H,H‐COSY, HSQCED, HMBC, and TOCSY. High‐resolution mass spectra (HRMS) were recorded on a Finnigan MAT 95 spectrometer (quadrupole, CI), or a *Bruker* maXis 4 G hr‐ToF (ESI, ToF).

### Compound 8

Protected isoserine 6 (940 mg, 2.82 mmol) was dissolved in THF (15 mL) and cooled to 0 °C. After the addition of 0.20 M LiOH_aq_ (15.5 mL, 3.10 mmol), the solution was stirred for 1 hour while slowly reaching room temperature. After full conversion, the reaction mixture was acidified with 0.2 M KHSO_4_ solution and extracted thrice with Et_2_O. The combined organic layers were dried with MgSO_4_ and concentrated in vacuo to give the crude carboxylic acid 7, which was immediately used in the following reaction. 7 was dissolved in anhydrous THF (15 mL) and cooled to − 20 °C. Triethylamine (412 µL, 2.96 mmol) and ethyl chloroformate (284 µL, 2.96 mmol) were added subsequently. The reaction mixture was stirred for 30 minutes before being cooled to − 40 °C. Diazomethane in Et_2_O was added dropwise to the active ester suspension. The resulting mixture was stirred for 18 hours while slowly reaching room temperature. The reaction mixture was diluted with H_2_O, and the aqueous layer was extracted with Et_2_O. The combined organic layers were washed with sat. NaHCO_3_ solution, dried with MgSO_4_ and concentrated in vacuo. The crude product was purified by column chromatography (silica, pentane:EtOAc 9:1) to give the diazoketone 8 (845 mg, 2.34 mmol, 83%) as a light‐yellow solid. R_f_ (8) = 0.33 (silica, pentane:EtOAc 8:2)$;\;{\left[\alpha \right]}_{D}^{20}$=  −66.8 (c  =  1.0, CHCl_3_); ^1^H NMR (400 MHz, CDCl_3_): δ = 5.75 (s, 1 H), 4.82 (m, 1 H), 4.15 (t, *J*  = 5.7 Hz, 1 H), 3.38 (m, 2 H), 1.44 (s, 9 H), 0.94 (s, 9 H), 0.13 (s, 3 H), 0.11 (s, 3 H) ppm; ^13^C NMR (100 MHz, CDCl_3_): δ = 196.4 (s), 155.6 (s), 79.5 (s), 75.9 (d), 53.2 (d), 44.7 (t), 28.4 (q), 25.8 (q), 18.1 (s), −5.0 (q), −5.1 (q) ppm; HRMS (ESI) calcd for: C_15_H_30_N_3_O_4_Si [M + H]^+^: 344.2000, found: 344.2000.

### Compound 11a

Triflic anhydride (4.51 mL, 26.7 mmol) was added dropwise to a − 15 °C cold solution of monoacetonide **10** (5.42 g, 17.8 mmol) and pyridine (4.32 mL, 53.4 mmol) in anhydrous CH_2_Cl_2_ (60 mL). The reaction mixture was stirred for 1 hour at − 10 °C. After full conversion, the mixture was diluted with CH_2_Cl_2_ and washed with 1.0 M HCl_aq_ and brine. The organic layer was dried with MgSO_4_ and concentrated in vacuo to give the crude triflate. The triflate and tetrabutylammonium hydrogen sulfate (30.0 mg, 89.0 µmol, 0.5 mol%) were dissolved in anhydrous DMF (90 mL). After the addition of NaN_3_ (5.79 g, 89.0 mmol), the suspension was stirred for 24 h at room temperature. The reaction mixture was diluted with EtOAc and washed with 5wt% LiCl_aq_, 1.0 M HCl_aq_, sat. NaHCO_3_ solution and brine. The organic layer was dried over MgSO_4_ and concentrated in vacuo. The crude product was purified by column chromatography (silica, PE:EtOAc 97:3) to give the azido‐sugar **11a** (2.80 g, 8.50 mmol, 48%) as a colorless oil. R_f_ (**11a**) = 0.53 (silica, PE:EtOAc 8:2);$\;{\left[\alpha \right]}_{D}^{20}$=  −88.7 (c  =  1.0, CHCl_3_); ^1^H NMR (400 MHz, CDCl_3_): δ = 5.79 (d, *J*  = 3.8 Hz, 1 H), 4.73 (dd, *J*  = 4.2, 4.2 Hz, 1 H), 4.11 (ddd, *J*  = 9.4, 2.8, 2.8 Hz, 1 H), 3.95 (dd, *J*  = 11.9, 2.7 Hz, 1 H), 3.82 (dd, *J*  = 12.0, 2.8 Hz, 1 H), 3.61 (dd, *J*  = 9.3, 4.6 Hz, 1 H), 1.58 (s, 3 H), 1.37 (s, 3 H), 0.91 (s, 9 H), 0.09 (s, 3 H), 0.08 (s, 3 H) ppm; ^13^C NMR (100 MHz, CDCl_3_): δ = 113.2 (s), 104.2 (d), 80.3 (d), 78.6 (d), 61.2 (d), 60.2 (t), 26.6 (q), 26.0 (q), 18.5 (s), −5.3 (q), −5.2 (q) ppm; HRMS (CI) calcd for: C_14_H_28_NO_4_Si [M‐N_2 _+ H]^+^: 302.1782, found: 302.1811.

### Compound 12a

In a 100 mL Schlenk flask under an atmosphere of nitrogen, azido‐sugar 11a (1.17 g, 3.46 mmol) and allyltrimethylsilane (2.20 mL, 13.9 mmol) were dissolved in anhydrous CH_2_Cl_2_ (35 mL). After cooling to − 20 °C, TiCl_4_ (759 µL, 6.92 mmol) was added dropwise, and stirring continued for 20 minutes. The reaction mixture was quenched by the addition of sat. NaHCO_3_ solution (3.5 mL). After pouring into more sat. NaHCO_3_ solution, the aqueous phase was extracted once with CH_2_Cl_2_. The combined organic layers were washed with brine, dried with MgSO_4,_ and concentrated in vacuo. The crude product was purified by column chromatography (silica, PE:EtOAc 9:1) to give *C*‐furanoside 12a (866 mg, 2.76 mmol, 80%, dr 97:3) as a colorless oil. R_f_ (12a) = 0.23 (silica, PE:EtOAc 9:1); ${\left[\alpha \right]}_{D}^{20}$=  −8.0 (c  =  1.0, CHCl_3_); ^1^H NMR (500 MHz, DMSO‐d_6_): δ = 5.80 (ddt, *J*  = 17.1, 10.2, 6.9 Hz, 1 H), 5.65 (d, *J*  = 5.6 Hz, 1 H), 5.08 (ddt, *J*  = 17.2, 2.2, 1.4 Hz, 1 H), 5.01 (ddt, *J*  = 10.2, 2.3, 1.2 Hz, 1 H), 3.93 (ddd, *J*  = 5.6, 5.6, 5.6 Hz, 1 H), 3.76–3.70 (m, 2 H), 3.66 (ddd, *J*  = 7.2, 5.9, 4.9 Hz, 1 H), 3.62 (d, *J*  = 3.4 Hz, 2 H), 2.31 (dddt, *J*  = 14.4, 6.5, 4.9, 1.4 Hz, 1 H), 2.14 (dddt, *J*  = 14.4, 7.1, 7.1, 1.3 Hz, 1 H), 0.87 (s, 9 H), 0.09 (s, 3 H), 0.08 (s, 3 H) ppm; ^13^C NMR (100 MHz, DMSO‐d_6_): δ = 134.8 (d), 116.9 (t), 81.9 (d), 80.8 (d), 74.8 (d, C‐4), 63.2 (t), 62.5 (d), 37.2 (t), 25.8 (q), 18.0 (s), −5.4 (q), −5.5 (q) ppm; HRMS (CI) calcd for: C_14_H_28_NO_3_Si [M‐N_2 _+ H]^+^: 286.183, found: 286.1838.

### Compound 12b

In a 25 mL Schlenk tube under an atmosphere of nitrogen, allyltrimethylsilane (965 µL, 6.07 mmol) was added to a 0 °C cold solution of compound 11a (500 mg, 1.52 mmol) in anhydrous CH_2_Cl_2_ (10 mL). After stirring for 10 minutes, BF_3_⋅OEt_2_ (801 µL, 3.04 mmol) was added, and stirring continued for 30 minutes. The cooling bath was removed, and the stirring continued for 3 hours at room temperature. Another portion of allyltrimethylsilane (965 µL, 6.07 mmol) and BF_3_⋅OEt_2_ (801 µL, 3.04 mmol) were added, and the mixture was stirred for another 2 hours at room temperature. The reaction mixture was quenched with sat. NaHCO_3_ solution (20 mL) and brine (2.0 mL) before being extracted three times with CH_2_Cl_2_. The combined organic layers were washed with brine, dried with MgSO_4_, and concentrated in vacuo. The residue was dissolved in anhydrous DMF (1.5 mL) before imidazole (124 mg, 1.82 mmol) and TBS‐Cl (137 mg, 911 µmol, 0.6 eq.) was added at room temperature. After stirring for 24 hours, the reaction mixture was diluted with EtOAc and washed with 5 wt% LiCl_aq_, 1.0 M HCl_aq_, sat. NaHCO_3_ solution and brine. The organic layer was dried over MgSO_4_ and concentrated in vacuo. The crude product was purified by automated flash chromatography (silica, CyH:EtOAc 0% to 20% EtOAc) to give *C*‐furanoside 12b (220 mg, 702 µmol, 46%, dr 99:1) and compound 13a (70.0 mg, 164 µmol, 11%, dr 96:4) as a colorless oil. R_f_ (12b) = 0.35 (silica, pentane:EtOAc 8:2); ${\left[\alpha \right]}_{D}^{20}$=  +18.4 (c  =  1.0, CHCl_3_); ^1^H NMR (500 MHz, DMSO‐d_6_): δ = 5.77 (ddt, *J*  = 17.2, 10.2, 6.9 Hz, 1 H), 5.61 (d, *J*  = 5.8 Hz, 1 H), 5.09 (ddt, *J*  = 17.2, 2.2, 1.5 Hz, 1 H), 5.00 (ddt, *J*  = 10.2, 2.2, 1.1 Hz, 1 H), 4.15 (ddd, *J*  = 5.8, 4.3, 2.9 Hz, 1 H), 4.04 (dd, *J*  = 4.6, 3.6 Hz, 1 H), 3.89 (ddd, *J*  = 8.1, 3.9, 3.9 Hz, 1 H), 3.69 (dd, *J*  = 11.2, 3.9 H, 1 H), 3.67–3.62 (m, 2 H, 5‐H), 2.27 (m, 2 H), 0.86 (s, 9 H), 0.05 (s, 3 H), 0.04 (s, 3 H) ppm; ^13^C NMR (125 MHz, DMSO‐d_6_): δ = 135.2 (d), 116.7 (t), 80.8 (d), 78.2 (d), 72.6 (d), 63.3 (t), 62.5 (d), 33.9 (t), 25.8 (q), 18.0 (s), −5.35 (q), −5.40 (q) ppm; HRMS (CI) calcd for: C_14_H_28_N_3_O_3_Si [M + H]^+^: 314.1894, found: 314.1893.

### Compound 13a

Imidazole (541 mg, 7.94 mmol) and TBS‐Cl (599 mg, 3.97 mmol) were subsequently added to a 0 °C cold solution of C‐furanoside 12a (830 mg, 2.65 mmol) in anhydrous DMF (12 mL). The reaction mixture was stirred for 16 hours while slowly reaching room temperature. After dilution with EtOAc, the mixture was washed with 5 wt% LiCl_aq_, 1.0 M HCl_aq_, sat. NaHCO_3_ solution and brine. The organic layer was dried over MgSO_4_ and concentrated in vacuo. The crude product was purified by column chromatography (silica, pentane:EtOAc 97:3) to give compound 13a (1.03 g, 2.40 mmol, 91%) as a colorless oil. R_f_ (13a) = 0.72 (silica, PE:EtOAc 9:1); ${\left[\alpha \right]}_{D}^{20}$=  −7.0 (c  =  1.0, CHCl_3_); ^1^H NMR (400 MHz, CDCl_3_): δ = 5.85 (ddt, *J*  = 17.1, 10.2, 6.9 Hz, 1 H), 5.11–5.06 (m, 2 H), 4.02 – 3.96 (m, 2 H), 3.84 (ddd, *J*  = 7.2, 5.7, 4.7 Hz, 1 H), 3.75 (dd, *J*  = 11.3, 3.3 Hz, 1 H), 3.70 (dd, *J*  = 11.3, 3.1 Hz, 1 H), 3.66 (dd, *J*  = 5.3, 5.3 Hz, 1 H), 2.38 (dddt, *J*  = 14.2, 6.3, 4.7, 1.5 Hz, 1 H), 2.20 (dddt, *J*  = 14.4, 7.4, 7.4, 1.4 Hz, 1 H), 0.94 (s, 9 H), 0.92 (s, 9 H), 0.15 (s, 3 H), 0.12 (s, 3 H), 0.09 (s, 3 H), 0.08 (s, 3 H) ppm; ^13^C NMR (100 MHz, CDCl_3_): δ = 134.4 (d), 117.5 (t), 82.7 (d), 81.6 (d), 76.6 (d), 63.3 (t), 62.9 (d), 37.6 (t), 26.1 (q), 25.9 (q), 18.5 (s), 18.2 (s), −4.4 (q), −4.7 (q), −5.2 (q), −5.4 (q) ppm; HRMS (CI) calcd for: C_20_H_42_N_3_O_3_Si_2_ [M + H]^+^: 428.2759, found: 428.2758.

### Compound 14a

A solution of compound 13a (1.02 g, 2.26 mmol) in anhydrous MeOH (16 mL) was cooled to 0 °C. After the addition of PyBr_3_ (36.1 mg, 113 µmol, 5mol%), the reaction mixture was stirred for 8 hours while slowly reaching room temperature. The solution was diluted with EtOAc and washed with 1.0 M HCl_aq_, sat. NaHCO_3_ solution, and brine. The organic layer was dried with MgSO_4_ and concentrated in vacuo. The crude product was purified by column chromatography (silica, PE:EtOAc 8:2) to give the primary alcohol 14a (580 mg, 1.85 mmol, 82%) as a colorless oil. R_f_ (14a) = 0.10 (silica, PE:EtOAc 9:1); ${\left[\alpha \right]}_{D}^{20}$=  −3.4 (c  =  1.0, CHCl_3_); ^1^H NMR (500 MHz, CDCl_3_): δ = 5.82 (ddt, *J*  = 17.2, 10.3, 7.0 Hz, 1 H), 5.17–5.11 (m, 2 H), 4.05–3.99 (m, 2 H, 2‐H), 3.92–3.85 (m, 2 H, 1‐H‘), 3.64 (ddd, *J*  = 11.9, 8.4, 3.1 Hz, 1 H), 3.58 (dd, *J*  = 6.8, 5.7 Hz, 1 H), 2.39 (dddt, *J*  = 14.6, 6.6, 5.0, 1.4 Hz, 1 H), 2.24 (dddt, *J*  = 14.3, 7.0, 7.0, 1.3 Hz, 1 H), 1.87 (m, 1 H), 0.95 (s, 9 H), 0.16 (s, 3 H), 0.13 (s, 3 H) ppm; ^13^C NMR (125 MHz, CDCl_3_): δ = 133.7 (d), 118.3 (t), 83.9 (d), 80.8 (d), 76.5 (d), 62.1 (t), 61.6 (d), 37.6 (t), 25.9 (q), 18.2 (s), −4.5 (q), −4.7 (q) ppm; HRMS (CI) calcd for: C_14_H_30_NO_3_Si [M‐N_2 _+ 3H]^+^: 288.1989, found: 288.1995.

### Compound 15a

A solution of DMSO (380 µL, 5.36 mmol) in anhydrous CH_2_Cl_2_ (1.2 mL) was added dropwise to a − 78 °C cold solution of oxalyl dichloride (235 µL, 2.68 mmol) in anhydrous CH_2_Cl_2_ (4.8 mL) keeping the temperature below − 70 °C. After complete addition, the mixture was stirred at − 60 to − 70 °C for 30 minutes. A solution of primary alcohol 14a (560 mg, 1.79 mmol) in anhydrous CH_2_Cl_2_ (4.0 mL) was added dropwise while keeping the temperature below − 60 °C. After stirring for another 45 minutes, a solution of DIPEA (1.56 mL, 8.93 mmol) in anhydrous CH_2_Cl_2_ (1.2 mL) was added dropwise while keeping the temperature around − 60 °C. The stirring was continued for 30 minutes before warming to 0 °C. After the addition of 1.0 M HCl_aq_ (20 mL), the aqueous layer was extracted thrice with CH_2_Cl_2_. The combined organic layers were washed with phosphate buffer (pH 7), dried with MgSO_4,_ and concentrated in vacuo to give the crude aldehyde.

The crude aldehyde was dissolved in MeCN (12 mL). A solution of NaH_2_PO_4_ (55.7 mg, 357 µmol) in H_2_O (1.2 mL) and H_2_O_2_ (182 µL, 1.79 mmol, 30 wt%) were subsequently added. After cooling to 0 °C, a solution of NaClO_2_ (323 mg, 2.86 mmol) in H_2_O (1.2 mL) was added dropwise. The resulting solution was stirred for 16 hours while slowly reaching room temperature. After the addition of Na_2_SO_4_ (50 mg) and brine (2.0 mL), the mixture was extracted thrice with EtOAc. The combined organic layers were dried with MgSO_4_ and concentrated in vacuo to give the carboxylic acid (589 mg, 1.69 mmol, 95%) as a colorless resin.

K_2_CO_3_ (367 mg, 2.65 mmol) and MeI (332 µL, 5.30 mmol) were added to a solution of the above prepared carboxylic acid (579 mg, 1.66 mmol, 94 wt%) in anhydrous DMF (12 mL). The resulting suspension was stirred for 18 hours at room temperature. After full conversion, the reaction mixture was diluted with EtOAc and washed with 5 wt% LiCl_aq_, 1.0 M HCl_aq_, sat. NaHCO_3_ solution and brine. The organic layer was dried over MgSO_4_ and concentrated in vacuo. The crude was purified by column chromatography (silica, PE:EtOAc 9:1) to give compound **15a** (457 mg, 1.34 mmol, 81%) as a colorless oil. R_f_ (**15a**) = 0.50 (silica, PE:EtOAc 8:2);$\;{\left[\alpha \right]}_{D}^{20}$=  −31.7  (c  =  1.0, CHCl_3_); ^1^H NMR (500 MHz, CDCl_3_): δ = 5.86 (ddt, *J*  = 17.2, 10.2, 6.9 Hz, 1 H), 5.18–5.11 (m, 2 H), 4.45 (d, *J*  = 5.6 Hz, 1 H), 4.08 (dd, *J*  = 5.2, 5.2 Hz, 1 H), 3.97 (ddd, *J*  = 6.8, 5.2, 5.2 Hz, 1 H), 3.81 (s, 3 H), 3.80 (dd, *J*  = 5.5, 5.5 Hz, 1 H), 2.41 (dddt, *J*  = 14.8, 6.8, 5.3, 1.4 Hz, 1 H), 2.34 (dddt, *J*  = 14.3, 7.0, 7.0, 1.3 Hz, 1 H), 0.94 (s, 9 H), 0.15 (s, 3 H), 0.12 (s, 3 H) ppm; ^13^C NMR (125 MHz, CDCl_3_): δ = 170.9 (s), 133.6 (d), 118.2 (t), 84.2 (d), 78.8 (d), 76.2 (d), 64.9 (d), 52.7 (q), 37.4 (t), 25.9 (q), 18.2 (s), −4.4 (q), −4.7 (q) ppm; HRMS (CI) calcd for: C_15_H_28_NO_4_Si [M‐N_2 _+ H]^+^: 314.1782, found: 314.1802.

### Compound 16a

Triphenylphosphine (401 mg, 1.53 mmol) was added to a solution of compound 15a (435 mg, 1.27 mmol) in THF:H_2_O (10.4 mL, 25:1). The reaction mixture was stirred for 16 hours at room temperature before another portion of triphenylphosphine (16.7 mg, 64.0 µmol) was added. After another 2 hours at room temperature, the reaction mixture was acidified with 0.1 M HCl_aq_. The mixture was washed twice with Et_2_O (discard) before sat. NaHCO_3_ solution was added. The aqueous layer was extracted four times with Et_2_O. The combined organic layers were dried with MgSO_4_ and concentrated in vacuo to give amine 16a (360 mg, 1.14 mmol, 90%) as a colorless resin. R_f_ (16a) = 0.22 (silica, PE:EtOAc 7:3); ^1^H NMR (400 MHz, CDCl_3_): δ = 5.85 (ddt, *J*  = 17.2, 10.2, 7.1 Hz, 1 H), 5.18–5.11 (m, 2 H), 4.14 (d, *J*  = 8.3 Hz, 1 H), 4.01–3.93 (m, 2 H, 4‐H), 3.80 (s, 3 H), 3.36 (dd, *J*  = 8.2, 4.9 Hz, 1 H), 2.38 (m, 1 H), 2.30 (dddt, *J*  = 14.1, 6.8, 6.8, 1.2 Hz, 1 H, 6‐H), 0.93 (s, 9 H), 0.11 (s, 3 H), 0.10 (s, 3 H) ppm; ^13^C NMR (125 MHz, CDCl_3_): δ = 172.5 (s), 134.1 (d), 118.0 (t), 85.9 (d), 82.3 (d), 76.4 (d), 58.6 (d), 52.3 (q), 38.5 (t), 26.0 (q), 18.2 (s), −4.3 (q), −4.5 (q) ppm.

### Compound 17a

Diazoketone 8 (150 mg, 415 µmol) and amine 16a (87.0 mg, 277 µmol) were dissolved in anhydrous CH_2_Cl_2_ (2.8 mL). At room temperature, the resulting yellow solution was irradiated with a blue LED (365 nm, 18 W) for 1 hours. The colorless solution was concentrated in vacuo, and the residue was purified by column chromatography (silica, pentane:EtOAc 8:2) to give compound 17a (120 mg, 190 µmol, 69%) as a colorless resin. R_f_ (17a) = 0.44 (SiO_2_, PE:EtOAc 7:3); ${\left[\alpha \right]}_{D}^{20}$=  −7.4  (c  =  1.0, CHCl_3_); ^1^H NMR (400 MHz, CDCl_3_): δ = 6.46 (d, *J*  = 8.0 Hz, 1 H), 5.87 (ddt, *J*  = 17.2, 10.2, 7.1 Hz, 1 H), 5.20–5.11 (m, 2 H), 4.88 (m, 1 H), 4.51 (ddd, *J*  = 8.0, 8.0, 5.5 Hz, 1 H), 4.27 (d, *J*  = 7.9 Hz, 1 H), 4.19 (tt, *J*  = 5.4, 5.4 Hz, 1 H), 4.06 (dd, *J*  = 5.6, 2.8 Hz, 1 H), 3.99 (td, *J*  = 6.3, 2.9 Hz, 1 H), 3.75 (s, 3 H), 3.37 (m, 1 H), 3.07 (ddd, *J*  = 14.0, 5.4, 5.4 Hz, 1 H), 2.46–2.29 (m, 4 H), 1.45 (s, 9 H), 0.92 (s, 9 H), 0.90 (s, 9 H), 0.12 (s, 3 H), 0.09 (s, 6 H), 0.09 (s, 3 H) ppm; ^13^C NMR (100 MHz, CDCl_3_): δ = 171.5 (s), 170.0 (s), 156.4 (s), 133.8 (d), 118.2 (t), 86.0 (d), 79.9 (d), 79.5 (s), 75.0 (d), 68.8 (d), 55.0 (d), 52.5 (q), 45.3 (t), 42.5 (t), 38.1 (t), 28.6 (q), 26.0 (q), 25.9 (q), 18.2 (s,), 18.1 (s), −4.4 (q), −4.5 (q), −4.6 (q), −4.7 (q) ppm; HRMS (CI) calcd for: C_30_H_59_N_2_O_8_Si_2_ [M + H]^+^: 631.3804, found: 631.3802.

### Compound 22a

0.20 M LiOH_aq_ (741 µL, 148 µmol) was slowly added to a 0 °C cold solution of  17a (85.0 mg, 135 µmol) in THF (700 µL). The resulting mixture was stirred for 2 hours while slowly reaching room temperature. After full conversion, the mixture was acidified with 0.1 M HCl_aq_ and extracted with CH_2_Cl_2_. The combined organic layers were dried with MgSO_4_ and concentrated in vacuo to give the crude carboxylic acid as a colorless resin.

4.0 M HCl in dioxane (806 µL, 3.22 mmol) was added to a solution of dipeptide **4** (110 mg, 322 µmol) in CH_2_Cl_2_ (800 µL) at 0 °C. After stirring for 2 hours, the reaction mixture was concentrated in vacuo. The residue was partitioned between 1.0 M K_2_CO_3_ solution and CH_2_Cl_2_. The aqueous phase was extracted with CH_2_Cl_2_. The combined organic layers were dried with MgSO_4_ and concentrated in vacuo to give the crude amine (52.0 mg, 216 µmol, 67%) as a colorless resin.

The above‐prepared crude carboxylic acid and amine (48.7 mg, 202 µmol) were dissolved in anhydrous DMF (1.3 mL). After cooling to 0 °C, EDC⋅HCl (28.4 mg, 148 µmol, 1.1 eq.) and HOBt (4.1 mg, 27.0 µmol, 0.2 eq.) were added, and the resulting mixture was stirred for 16 hours while slowly reaching room temperature. The reaction mixture was diluted with EtOAc and washed with 5 wt% LiCl_aq_, 1.0 M HCl_aq_, sat. NaHCO_3_ solution and brine. The organic layer was dried over MgSO_4_ and concentrated in vacuo. The crude product was purified by automated reversed phase column chromatography (C18 spherical, H_2_O:MeCN 10% to 90% MeCN) to give tetrapeptide **22a** (108 mg, 129 µmol, 96%, dr 90:10) as a white foam.$\;{\left[\alpha \right]}_{D}^{20}$ =  −2.9 (c  =  1.0, CHCl_3_); ^1^H NMR (500 MHz, DMSO‐d_6_, 373 K): δ = 10.29 (s, 1 H), 8.11 (d, *J*  = 8.2 Hz, 1 H), 7.30 (d, *J*  = 8.2 Hz, 1 H), 6.09 (m, 1 H), 5.89 (ddt, *J*  = 17.1, 10.2, 6.9 Hz, 1 H), 5.37 (m, 1 H), 5.17–5.06 (m, 3 H), 4.38 (m, 1 H), 4.17–4.08 (m, 4 H), 4.02 (m, 1 H), 3.91 (td, *J*  = 6.4, 4.2 Hz, 1 H), 3.67 (s, 3 H), 3.10–2.96 (m, 2 H), 3.02 (s, 3 H), 2.75 (dd, *J*  = 17.5, 9.5 Hz, 1 H), 2.42–2.33 (m, 4 H), 2.24 (dd, *J*  = 14.6, 5.9 Hz, 1 H), 1.45 (s, 9 H), 0.91 (s, 9 H), 0.87 (s, 9 H), 0.08 (s, 6 H), 0.07 (s, 3 H), 0.06 (s, 3 H) ppm; ^13^C NMR (125 MHz, DMSO‐d_6_, 298 K): δ = 174.7 (s), 169.9 (s), 169.9 (s), 169.3 (s), 167.8 (s), 157.6 (s), 155.6 (s), 134.5 (d), 117.5 (t), 87.1 (d), 84.3 (d), 80.0 (s), 77.5 (d1), 74.2 (d), 68.5 (d), 54.2 (d), 51.7 (q), 48.9 (t), 46.7 (d), 45.4 (t), 41.8 (t), 37.4 (t), 36.3 (q), 34.4 (t), 28.3 (q), 25.8 (q), 25.7 (q), 17.8 (s), 17.7 (s), −4.75 (q), −4.83 (q), −4.90 (q), −4.92 (q) ppm; HRMS (ESI) calcd for: C_39_H_70_N_5_O_11_Si_2_ [M + H]^+^: 840.4605, found: 840.4603.

### Compound 23a

0.20 M LiOH_aq_ (570 µL, 114 µmol) was slowly added to a 0 °C cold solution of tetrapeptide 22a (87.0 mg, 104 µmol) in THF (500 µL). The resulting mixture was stirred for 2 hours while slowly reaching room temperature. After full conversion, the mixture was acidified with 0.1 M HCl_aq_ and extracted with CH_2_Cl_2_. The combined organic layers were dried with MgSO_4_ and concentrated in vacuo to give the crude carboxylic acid as a white foam.

4.0 M HCl in dioxane (531 µL, 2.13 mmol, 10 eq.) was added to a solution of Boc‐Trp‐GlyOMe (83.2 mg, 208 µmol) in CH_2_Cl_2_ (50 µL) at 0 °C. After stirring for 1 hour, the reaction mixture was concentrated in vacuo to give the crude amine as hydrochloride salt.

The above‐prepared crude carboxylic acid and amine hydrochloride (2.0 eq.) were dissolved in anhydrous DMF (1.0 mL). After cooling to 0 °C, NMM (47.0 µL, 427 µmol) and HBTU (43.4 mg, 115 µmol, 1.1 eq.) were subsequently added, and the resulting mixture was stirred for 16 hours while slowly warming to room temperature. The reaction mixture was diluted with EtOAc and washed with 5 wt% LiCl_aq_, 1.0 M HCl_aq_, sat. NaHCO_3_ solution and brine. The organic layer was dried over MgSO_4_ and concentrated in vacuo. The crude product was purified by automated reversed phase column chromatography (C18 spherical, H_2_O:MeCN 10% to 90% MeCN) to give hexapeptide **23a** (85.0 mg, 78.0 µmol, 75%, dr 97:3) as a white foam. The epimer from the previous coupling was mainly separated during the column chromatography (hence the improved diastereomeric ratio). ${\left[\alpha \right]}_{D}^{20}$ =  +45.0  (c  =  0.5, CHCl_3_); ^1^H NMR (500 MHz, DMSO‐d_6_): δ = 10.79 (m, 1 H), 10.39 (s, 1 H), 8.45–8.36 (m, 2 H), 8.09 (d, *J*  = 8.5 Hz, 1 H), 7.76 (d, *J*  = 8.3 Hz, 1 H), 7.57 (d, *J*  = 7.9 Hz, 1 H), 7.31 (d, *J*  = 8.1 Hz, 1 H), 7.13 (s, 1 H), 7.04 (ddd, *J*  = 8.1, 6.9, 1.2 Hz, 1 H), 6.97 (ddd, *J*  = 8.0, 6.9, 1.1 Hz, 1 H), 6.66 (m, 1 H), 5.85 (m, 1 H), 5.29 (s, 1 H), 5.15–5.02 (m, 3 H), 4.57 (m, 1 H), 4.33 (ddd, *J*  = 7.2, 7.2, 7.2 Hz, 1 H), 4.10 (d, *J*  = 6.9 Hz, 1 H), 4.03 (m, 1 H), 3.98–3.76 (m, 6 H), 3.64 (s, 3 H), 3.17 (dd, *J*  = 14.7, 4.8 Hz, 1 H), 3.03–2.88 (m, 3 H), 2.84 (s, 3 H), 2.73 (m, 1 H), 2.38–2.24 (m, 4 H), 2.16 (dd, *J*  = 14.7, 6.1 Hz, 1 H), 1.36 (s, 9 H), 0.86 (s, 9 H), 0.82 (s, 9 H), 0.03 (s, 6 H), 0.02 (s, 3 H), 0.01 (s, 3 H) ppm; ^13^C NMR (125 MHz, DMSO‐d_6_): δ = 174.7 (s), 171.9 (s), 170.2 (s), 169.9 (s), 169.3 (s), 168.1 (s), 167.6 (s), 157.0 (s), 155.6 (s), 136.0 (s), 134.5 (d), 127.3 (s), 123.6 (d), 120.8 (d), 118.3 (d), 118.2 (d), 117.6 (t), 111.2 (d), 109.9 (s), 87.6 (d), 84.3 (d), 79.9 (d), 77.5 (s), 74.1 (d), 68.5 (d), 54.2 (d), 53.2 (d), 51.7 (q), 50.0 (t), 46.6 (d), 45.3 (t), 41.8 (t), 40.7 (t), 37.4 (t8), 36.2 (q), 34.5 (t), 28.3 (q), 27.7 (t, C‐23), 25.8 (q), 25.7 (q), 17.8 (s), 17.7 (s), −4.75 (q), −4.82 (q), −4.90 (q), −4.92 (q) ppm; HRMS (ESI) calcd for: C_52_H_83_N_8_O_13_Si_2_ [M + H]^+^: 1083.5613, found: 1083.5622.

### Compound 25a

0.20 M LiOH_aq_ (359 µL, 72.0 µmol) was slowly added to a 0 °C cold solution of hexapeptide 23a (74.0 mg, 68.3 µmol) in THF (350 µL). The resulting mixture was stirred for 4 hours while slowly warming to room temperature. After full conversion, the mixture was acidified with 0.1 M HCl_aq_ and extracted with CH_2_Cl_2_. The combined organic layers were dried with MgSO_4_ and concentrated in vacuo to give the crude carboxylic acid as a white foam.

A preformed cleavage cocktail TFA:TIPS:H_2_O (260 µL, 185:10:5) was added to a solution of the crude carboxylic acid in anhydrous CH_2_Cl_2_ (300 µL) at 0 °C. After 110 minutes, reaction control via LC‐MS showed full conversion (60% Boc‐deprotection and 40% Boc‐ and monoTBS‐deprotection). The reaction mixture was concentrated in vacuo. The residue was dissolved in anhydrous DMF (70 mL). After cooling to 0 °C, DIPEA (119 µL, 683 µmol), HOAt (105 mg, 683 µmol), and PyAOP (356 mg, 683 µmol) were added. The reaction mixture was stirred for 72 hours while slowly reaching room temperature. After dilution with EtOAc, the mixture was washed with 5 wt% LiCl_aq_, 1.0 M HCl_aq_, sat. NaHCO_3_ solution and brine. The organic layer was dried over MgSO_4_ and concentrated in vacuo. The crude product was purified by automated reversed phase column chromatography (C18 spherical, H_2_O:MeCN 10% to 90% MeCN) to give the protected dehydrosocein precursor **25a** (25.0 mg, 26.5 µmol, 39%) as a white foam and its mono‐TBS‐deprotected variant **25a(OH)** (15.0 mg, 17.9 mmol, 26%) as a white foam.

Imidazole (2.6 mg, 38.0 mmol) and TBS‐Cl (3.0 mg, 19.8 mmol) were subsequently added to a 0 °C cold solution of the mono‐TBS‐deprotected variant **25a(OH)** (15.0 mg, 17.9 mmol) in anhydrous DMF (180 µL). The reaction mixture was stirred for 16 hours while slowly reaching room temperature. After dilution with EtOAc, the mixture was washed with 5 wt% LiCl_aq_, 1.0 M HCl_aq_, sat. NaHCO_3_ solution and brine. The organic layer was dried over MgSO_4_ and concentrated in vacuo. The crude product was purified by automated reversed phase column chromatography (C18 spherical, H_2_O:MeCN 10% to 90% MeCN) to give another portion of the protected dehydrosocein precursor **25a** (10.5 mg, 11.1 µmol, 62%) as a white foam.$\;{\left[\alpha \right]}_{D}^{20}$ =  −21.4 (c  =  1.0, CHCl_3_); ^1^H NMR (500 MHz, DMSO‐d_6_): δ = 10.86 (m, 1 H), 10.36 (s, 1 H), 8.67 (d, *J * =  8.2 Hz, 1 H), 8.26 (d, *J * = 8.3 Hz, 1 H), 8.17 (dd, *J * = 7.5, 5.0 Hz, 1 H), 7.52 (d, *J * = 7.9 Hz, 1 H), 7.35 (d, *J * = 8.1 Hz, 1 H), 7.26–7.20 (m, 2 H), 7.07 (dd, *J * = 7.6, 7.6 Hz, 1 H), 7.03 – 6.95 (m, 2 H), 5.86 (ddt, *J * = 17.2, 10.2, 7.0 Hz, 1 H), 5.24 (s, 1 H), 5.19 (m, 1 H), 5.13 (dd, *J * = 17.3, 2.0 Hz, 1 H), 5.07 (dd, *J *= 10.2, 2.1 Hz, 1 H), 4.50 (d, *J *= 15.6 Hz, 1 H), 4.37 (ddd, *J *= 9.2, 9.2, 5.2 Hz, 1 H), 4.25 (ddd, *J *= 9.9, 5.0, 5.0 Hz, 1 H), 4.18 (m, 1 H), 3.99 (d, *J *= 9.2 Hz, 1 H), 3.90 (dd, *J *= 5.3, 1.9 Hz, 1 H), 3.85 (td, *J *= 6.6, 1.9 Hz, 1 H), 3.77 (dd, *J *= 16.9, 7.6 Hz, 1 H), 3.46 (d, *J *= 16.0 Hz, 1 H), 3.42 (m, 1 H), 3.21 (dd, *J *= 14.7, 4.3 Hz, 1 H), 3.16 (s, 3 H), 3.14 (m, 1 H,), 3.00 (dd, *J *= 14.8, 9.8 Hz, 1 H), 2.82–2.73 (m, 2 H, 1‐H), 2.37–2.23 (m, 3 H, 8‐H), 2.13 (m, 2 H), 0.89 (s, 9 H), 0.78 (s, 9 H), 0.04 (s, 6 HH), 0.02 (s, 3 HH), 0.02 (s, 3 H) ppm; ^13^C NMR (125 MHz, DMSO‐d_6_): δ = 174.2 (s), 171.8 (s), 171.5 (s), 169.5 (s), 169.1 (s), 168.5 (s), 168.4 (s), 156.4 (s), 136.2 (s), 134.4 (d), 126.9 (s), 123.9 (d), 121.0 (d), 118.3 (d), 118.1 (d), 117.6 (t), 111.4 (d), 109.8 (s), 87.9 (d), 85.9 (d), 79.0 (d), 74.6 (d), 67.6 (d), 55.6 (d), 54.1 (d), 51.5 (t), 46.6 (d), 44.5 (t), 42.8 (t), 42.5 (t), 37.7 (t), 37.5 (q), 34.2 (t), 26.4 (t), 25.7 (q), 25.7 (q), 17.8 (s), 17.6 (s), −4.7 (q), −4.8 (q), −4.85 (q), −4.93 (q) ppm; HRMS (ESI) calcd for: C_46_H_71_N_8_O_10_Si_2_ [M + H]^+^: 951.4826, found: 951.4836.

### Compound 27a

1.0 M TBAF in THF (9.05 µL, 9.05 µmol, 2.05 eq.) was added to a 0 °C cold solution of protected dehydrosocein precursor 25a (4.2 mg, 4.4 µmol) in anhydrous THF (100 µL). The resulting solution was stirred for 4 hours while slowly warming to room temperature. After the addition of a droplet of H_2_O, the reaction mixture was adsorbed on isolute and purified by automated reversed phase column chromatography (C18 spherical, H_2_O:MeCN 10% to 90% MeCN) followed by preparative HPLC (H_2_O:MeCN 10% to 85% MeCN) to give dehydrosocein precursor 27a (2.3 mg, 3.2 µmol, 72%) as an amorphous solid after lyophilization. ${\left[\alpha \right]}_{D}^{20}$=  −39.3  (c  =  0.3, MeOH); ^1^H‐NMR (500 MHz, DMSO‐d_6_): δ = 10.86 (d, ^3^ *J*_NH,31_  = 2.4 Hz, 1 H), 10.39 (s, 1 H), 8.57 (d, ^3^ *J*_NH,22_  = 5.7 Hz, 1 H), 8.46 (d, ^3^ *J*_NH,13_  = 8.3 Hz, 1 H), 8.29 (t, ^3^ *J*_NH,33_  = 6.2 Hz, 1 H), 7.60 (d, ^3^ *J*_NH,5_  = 8.4 Hz, 1 H), 7.52 (d, ^3^ *J*_29,28_  = 7.9 Hz, 1 H), 7.34 (d, ^3^ *J*_26,27_  = 8.0 Hz, 1 H), 7.26 (t, ^3^ *J*_NH,1_  = 5.2 Hz, 1 H), 7.21 (d, ^3^ *J*_31,NH_  = 2.3 Hz, 1 H), 7.07 (ddd, ^3^ *J*_28,29_  = 8.1 Hz, ^3^ *J*_28,27_  = 7.0 Hz, ^4^ *J*_28,26_  = 1.2 Hz, 1 H), 6.99 (ddd, ^3^ *J*_27,26_  = 7.9 Hz, ^3^ *J*_27,28_  = 6.9 Hz, ^4^ *J*_27,29_  = 1.0 Hz, 1 H), 5.85 (ddt, ^3^ *J*_9,10_  = 17.1 Hz, ^3^ *J*_9,10‘_  = 10.3 Hz, ^3^ *J*_9,8_  = 6.8 Hz, 1 H), 5.48 (d, ^3^ *J*_OH,6_  = 4.2 Hz, 1 H), 5.38 (d, ^4^ *J*_17,13_  = 1.5 Hz, 1 H), 5.18 (m, 1 H), 5.12 (ddt, ^3^ *J*_10‘,9_  = 17.3 Hz, ^2^ *J*_10‘,10_  = 1.7 Hz, ^4^ *J*_10‘,8_  = 1.7 Hz, 1 H), 5.04 (ddt, ^3^ *J*_10,9_  = 10.2 Hz, ^2^ *J*_10,10‘_  = 2.2 Hz, ^4^ *J*_10,8_  = 1.2 Hz, 1 H), 4.67 (d, ^3^ *J*_OH,2_  = 4.9 Hz, 1 H), 4.56 (d, ^3^ *J*_20‘,20_  = 15.8 Hz, 1 H ‘), 4.39 (ddd, ^3^ *J*_5,11_  = 8.6 Hz, ^3^ *J*_5,NH_  = 8.6 Hz, ^3^ *J*_5,6_  = 5.5 Hz, 1 H), 4.18 (ddd, ^3^ *J*_22,23_  = 10.1 Hz, ^3^ *J*_22,NH_  = 5.2 Hz, ^3^ *J*_22,23‘_  = 5.2 Hz, 1 H), 3.97 (m, 1 H), 3.89 (d, ^3^ *J*_11,5_  = 8.8 Hz, 1 H), 3.86 – 3.77 (m, 2 H, 6‐H), 3.61 (m, 2 H), 3.44 (d, ^2^ *J*_20,20‘_  = 15.8 Hz, 1 H), 3.19 (dd, ^2^ *J*_23‘,23_  = 14.7 Hz, ^3^ *J*_23‘,22_  = 4.6 Hz, 1 H), 3.16 – 3.09 (m, 4 H), 3.01 (dd, ^2^ *J*_23,23‘_  = 14.7 Hz, ^3^ *J*_23,22_  = 9.4 Hz, 1 H), 2.80 (m, 1 H), 2.75 (dd, ^2^ *J*_14,14‘_  = 17.5 Hz, ^3^ *J*_14,13_  = 9.6 Hz, 1 H), 2.35 – 2.20 (m, 5 H); ^13^C‐NMR (125 MHz, DMSO‐d_6_): δ = 174.4 (s), 171.5 (s), 171.0 (s), 170.3 (s), 169.9 (s), 168.8 (s), 168.2 (s), 156.4 (s), 136.1 (s), 134.9 (d, C‐9), 127.0 (s), 124.0 (d), 121.0 (d), 118.4 (d), 118.1 (d9), 117.1 (t), 111.4 (d), 109.8 (s), 88.2 (d), 85.8 (d), 80.3 (d), 72.9 (d), 66.0 (d), 55.5 (d), 54.3 (d), 50.7 (t), 46.4 (d), 45.0 (t), 42.5 (t), 41.5 (t), 37.8 (t), 37.1 (q), 34.2 (t), 26.2 (t)); HRMS (ESI) calcd for: C_34_H_43_N_8_O_10_ [M + H]^+^: 723.3097, found: 723.3097.

### Compound 28a

In a 4 mL brown glass‐vial under an atmosphere of argon, protected dehydrosocein precursor 25a (8.0 mg, 8.4 µmol) was dissolved in argon‐degassed, anhydrous CH_2_Cl_2_ (200 µL). After the addition of styrene (9.73 µL, 84.1 µmol, 10 eq.) and Grubbs I catalyst in CH_2_Cl_2_ (42.0 µL, 0.841 µmol, 0.02 M, 10 mol%), the reaction mixture was stirred for 24 hours. Another portion of Grubbs I catalyst (42.0 µL, 0.841 µmol, 0.02 M, 10 mol%) was added, and the stirring was continued for 56 hours. After adsorption on isolute, the mixture was purified by automated reversed phase column chromatography (C18 spherical, H_2_O:MeCN 10% to 90% MeCN) to give the silylated olefination product (3.5 mg, 3.41 µmol, 41%, 93%brsm) as a white foam.

1.0 M TBAF in THF (6.98 µL, 6.98 µmol, 2.05 eq.) was added to a 0 °C cold solution of the above‐prepared olefination product (3.5 mg, 3.41 µmol) in anhydrous THF (70 µL). The resulting solution was stirred for 4 hours while slowly warming to room temperature. After the addition of a droplet of H_2_O, the reaction mixture was adsorbed on isolute and purified by automated reversed phase column chromatography (C18 spherical, H_2_O:MeCN 10% to 90% MeCN) followed by preparative HPLC (H_2_O:MeCN 10% to 100% MeCN) to give dehydrosocein derivative **28a** (2.6 mg, 3.25 µmol, 96%) as an amorphous solid after lyophilization.$\;{\left[\alpha \right]}_{D}^{20}$ =  −28.6 (c  =  0.3, MeOH); ^1^H NMR (500 MHz, DMSO‐d_6_): δ = 10.86 (d, *J*  = 2.4 Hz, 1 H), 10.39 (s, 1 H), 8.57 (d, *J*  = 5.7 Hz, 1 H), 8.48 (d, *J*  = 8.3 Hz, 1 H), 8.29 (t, *J*  = 6.0 Hz, 1 H), 7.60 (d, *J*  = 8.6 Hz, 1 H), 7.52 (d, *J*  = 7.9 Hz, 1 H), 7.39 (m, 2 H), 7.34 (d, *J*  = 8.0 Hz, 1 H), 7.30 (m, 2 H), 7.24 (m, 1 H), 7.23–7.18 (m, 2 H), 7.07 (ddd, *J*  = 8.2, 7.0, 1.2 Hz, 1 H), 6.99 (ddd, *J*  = 7.9, 7.0, 1.1 Hz, 1 H), 6.49 (d, *J*  = 16.0 Hz, 1 H), 6.37 (dt, *J*  = 15.9, 7.0 Hz, 1 H), 5.52 (d, *J*  = 4.3 Hz, 1 H), 5.39 (d, *J*  = 1.6 Hz, 1 H), 5.20 (m, 1 H), 4.66 (d, *J*  = 4.9 Hz, 1 H), 4.55 (d, *J*  = 15.8 Hz, 1 H), 4.45 (ddd, *J*  = 8.7, 8.7, 5.6 Hz, 1 H), 4.18 (ddd, *J*  = 10.0, 5.2, 5.2 Hz, 1 H), 3.98 (m, 1 H), 3.95–3.91 (m, 2 H), 3.88 (m, 1 H), 3.62 (m, 2 H), 3.44 (d, *J*  = 15.8 Hz, 1 H), 3.19 (dd, *J*  = 14.6, 4.6 Hz, 1 H), 3.16–3.08 (m, 4 H), 3.01 (dd, *J*  = 14.7, 9.5 Hz, 1 H), 2.79 (m, 1 H), 2.75 (dd, *J*  = 17.7, 9.8 Hz, 1 H), 2.46 (m, 2 H), 2.33 (dd, *J*  = 17.8, 6.3 Hz, 1 H), 2.22 (m, 2 H) ppm; ^13^C NMR (125 MHz, DMSO‐d_6_): δ = 174.4 (s), 171.5 (s), 171.0 (s), 170.2 (s), 170.0 (s), 168.8 (s), 168.3 (s), 156.4 (s), 137.2 (s, C‐35), 136.1 (s), 131.6 (d, C‐10), 128.5 (d, C‐37), 127.1 (d, C‐38), 127.0 (s, C‐25), 126.6 (d, C‐9), 125.9 (d), 124.0 (d), 121.0 (d), 118.4 (d), 118.1 (d), 111.4 (d), 109.8 (s), 88.2 (d), 86.1 (d), 80.3 (d), 73.0 (d), 66.0 (d2), 55.5 (d), 54.4 (d), 50.7 (t), 46.4 (d), 45.0 (t), 42.5 (t), 41.5 (t), 37.2 (q), 37.0 (t), 34.2 (t), 26.2 (t) ppm; HRMS (ESI) calcd for: C_40_H_47_N_8_O_10_ [M + H]^+^: 799.3410, found: 799.3420.

## Supporting Information

The remaining experimental procedures, spectroscopic data and copies of ^1^H and ^13^C spectra are available in the Supporting Information.

## Conflict of Interest

The authors declare no conflict of interest.

## Supporting information



Supporting Information The remaining experimental procedures, spectroscopic data and copies of ^1^H and ^13^C spectra are available in the Supporting Information.

## Data Availability

The data that support the findings of this study are available in the supplementary material of this article.
