# Supplementary material for: Stereoselective Syntheses of Cyclic Microsclerodermin Derivatives
Source: Chemistry. 2025 Sep 26;31(60):e02459. doi: 10.1002/chem.202502459 (PMC12559470; doi:10.1002/chem.202502459)
Supplement: Supplementary file 1 — Supporting Information The remaining experimental procedures, spectroscopic data and copies of 1H and 13C spectra are available in the Supporting Information. [file CHEM-31-e02459-s001.pdf]

List of Abbreviations

Ac	acetyl
Alloc	allyloxycarbonyl
aq.	aqueous
Asp	aspartic acid
Bn	benzyl
Boc	*tert*-butyloxycarbonyl
brine	saturated aqueous NaCl-solution
brsm	based on recovered starting material
Bz	benzoyl
CAN	ceric ammonium nitrate
cat.	catalytic
Cbz	benzyloxycarbonyl
CDI	*N*,*N*'-carbonyldiimidazole
CI	chemical ionization
conc	concentrated
COSY	correlated spectroscopy
Cy	cyclohexyl
CyH	cyclohexane
DCC	*N*,*N*'-dicyclohexylcarbodiimide
DEPT	distortionless enhancement by polarization transfer
DICHED	dicyclohexylethane-1,2-diol
DIPEA	*N*,*N*'-diisopropylethylamine
DMAP	4-dimethylaminopyridine
DMF	dimethylformamide
DMP	Dess-Martin-periodinane
DMS	dimethyl sulfide
DMSO	dimethylsulfoxide
dr	diastereomeric ratio
ECF	ethyl chloroformate
EDC	1-ethyl-3-(3-dimethylamino-propyl)carbodiimide
ee	enantiomeric excess
eq.	equivalents
ESI	electrospray ionization
Et	ethyl
et al.	*et alia*
GABOB	γ-amino-β-hydroxybutyric acid
GC-FID	gas chromatography with flame ionization detector
Gly	glycine
Grubbs I	benzylidene-bis(tricyclohexylphosphino)-dichlororuthenium
Grubbs II	(1,3-bis(2,4,6-trimethylphenyl)-2-imidazolidinylidene)dichloro-(phenylmethylene)(tricyclohexyl phosphine)-ruthenium
HATU	1-[bis(dimethylamino)-methylene]-1*H*-1,2,3-triazolo[4,5-b]pyridinium 3-oxide hexafluorophosphate
HBTU	2-(1*H*-benzotriazol-1-yl)-1,1,3,3-tetramethyluronium hexafluorophosphate
HMBC	heteronuclear multiple-bond correlation spectroscopy
HOAt	1-hydroxy-7-azabenzotriazole
HOBt	1*H*-1,2,3-benzotriazol-1-ol
HPLC	high-performance liquid chromatography
HRMS	high-resolution mass spectroscopy
HSQCED	heteronuclear single quantum correlation spectroscopy – DEPT edited
IBCF	isobutyl chloroformate
^{i}Pr	isopropyl
Ise	isoserine
KMM	potassium methyl malonate
LC-MS	liquid chromatography–mass spectrometry
LDA	lithium diisopropylamide
LED	light-emitting diode
Me	methyl
Met	methionine
*n*Bu	*n*-butyl

NMM	*N*-methylmorpholine
NMR	nuclear magnetic resonance
NOE	nuclear Overhauser effect
Orn	ornithine
PE	petroleum ether
Ph	phenyl
PMB	*p*-methoxybenzyl
prep.	preparative
pTsOH	*p*-toluene sulfonic acid
PyAOP	(7-azabenzotriazol-1-yloxy)-tripyrrolidinophosphonium hexafluorophosphate
PyBOP	(benzotriazol-1-yloxy)-tripyrrolidinophosphonium hexafluorophosphate
RP	reversed-phase
rt	room temperature
Sar	sarcosine
sat.	saturated
TBAF	tetra-*n*-butylammonium fluoride
TBDPS	*tert*-butyldiphenylsilyl
TBS	*tert*-butyldimethylsilyl
^{t}Bu	*tert*-butyl
Tf	trifluoromethane sulfonyl
TFA	trifluoroacetic acid
TFAA	trifluoroacetic anhydride
THF	tetrahydrofuran
TIPS	triisopropyl silane
TLC	thin-layer chromatography
TMS	trimethylsilyl
TOCSY	total correlation spectroscopy
Troc	2,2,2-trichloroethyloxy
Trp	tryptophan
UV	ultra violet

General Information

All reactions were carried out in oven-dried glassware under a nitrogen atmosphere if an anhydrous solvent is mentioned in the procedure. Anhydrous THF was prepared by distillation over sodium/benzophenone. Other anhydrous solvents were purchased from *Acros Organics* and *Thermo Scientific*. Petroleum ether (PE), pentane, and ethyl acetate (EtOAc) were distilled prior use.

NMR spectra were measured on a *Bruker* Avance II 400 (400 MHz, 5 mm BBO Probe, 298 K), a *Bruker* Avance I 500 (500 MHz, 5 mm TCI Probe, 298 K), or a *Bruker* Avance Neo 500 (500 MHz, 5 mm TCI Prodigy CryoProbe, 298 K). Spectra were calibrated on the solvent signals [$CDCl_3$ (^1H 7.27 ppm, ^{13}C 77.16 ppm) or DMSO-d6 (^1H 2.50 ppm, ^{13}C 39.52 ppm)]. The spectral data were analyzed with MestReNova 14.2 from *MestreLabReasearch S.L.* Chemical shifts (δ) are reported in ppm and coupling constants (*J*) in Hz. Multiplicities in ^1H-NMR spectra are reported as singlet (s), doublet (d), triplet (t), quartet (q), and multiplet (m). ^{13}C-NMR spectra were measured boadband decoupled, theoretical multiplicity of the carbon is given as s (quaternary C-atom), d (tertiary C-Atom, t (secondary C-Atom), and q (primary C-Atom). Assignments were done using two-dimensional measurements like H,H-COSY, HSQCED, HMBC, and TOCSY.

Reaction monitoring was done by thin-layer chromatography (**TLC**) on Polygram® SIL G/UV_{254} plates by *Macherey-Nagel* ($KMnO_4$, cerium-molybdate or ninhydrin stains) and **LC-MS** analysis on a *Shimadzu* Prominence-*i* LC-2030 (*Phenomenex* Onyx® C18, 50 x 4.6 mm) coupled with *Shimadzu* LCMS-2020 (ESI ionization). All runs were performed at a 4 mL/min flow rate with a column temperature of 40 °C and 0.1% $HCOOH_{aq}$/MeCN as the mobile phase.

0.1% $HCOOH_{aq}$: MeCN	short Method (time)	long Method (time)
**90:10 to 1:99**	1.5 min	6.0 min
**1:99**	1.0 min	1.5 min
**90:10**	0.7 min	1.0 min

For **column chromatography,** silica gel 60M 40 – 63 µm by *Macherey Nagel* was used. Automated flash column chromatography was done on a *Büchi* Pure C815 Flash with *Teledyne Isco* RediSep R_f cartridges. Automated reversed-phase column chromatography was done on a *Büchi* Reveleris® Prep with *Büchi* FlashPure Select C18 (spherical) cartridges or *Kinesis* Telos C18 cartridges. Preparative HPLC was done with a *Büchi* Reveleris® Prep with a *Phenomenex* Luna® (C18, 5 µm, 21.2 x 250 mm).

Specific optical rotation ($[\alpha]_D^{20}$) in [deg·mL/(g·dm)]· was measured on a P-8000-T polarimeter by *A. Krüss Optronic GmbH* with a PT80 thermostat by *A. Krüss Optronic GmbH* at 20 °C (λ = 589 nm). Concentrations are given in g/100 mL.

High-resolution mass spectra (**HRMS**) were recorded at Saarland University by Rudi Thomes on a *Finnigan* MAT 95 (CI, sector field) or at HIPS Saarland on a *Bruker* maXis 4G hr-ToF (ESI, ToF).

Melting points were measured in open glass capillaries on an M3000 from *Krüss Optronic GmbH*.

GC-FID analysis was performed on a GC-2010 System from *Shimadzu* (FID-detector, AOC-20i autoinjector) with a CP-Chirasil-Dex CB (Varian, 25 m x 0.25 mm, 0.25 µm diameter).

Photochemical reactions were conducted in a EvoluChem™ PhotoRedOx Box by *HepatoChem* using 18 W LED-lamps (365 nm, 405 nm and 450 nm).

Synthesis of the compounds

Benzyl (*R*)-4-amino-3-((*tert*-butoxycarbonyl)amino)-4-oxobutanoate[15] 1

According to Donohoe *et al.*[15], in a 250 mL Schlenk flask under an atmosphere of nitrogen, a solution of Boc-D-Asp(OBn)-OH (4.50 g, 13.9 mmol) and Triethylamine (2.33 mL, 16.7 mmol, 1.2 eq.) in anhydrous THF (90 mL) was cooled to 0 °C. Ethyl chloroformate (1.60 mL, 16.7 mmol, 1.2 eq.) was added dropwise, and the resulting suspension was stirred for 2 h at 0 °C. After the addition of aqueous ammonia solution (11.0 mL, 167 mmol, 35 wt%, 12 eq.), the reaction mixture was stirred for 30 min at 0 °C. The resulting solution was diluted with EtOAc and water. The aqueous layer was extracted twice with EtOAc, and the combined organic layers were washed with 0.5 M HCl_{aq}, sat. $NaHCO_3$ solution and brine. Drying over $MgSO_4$ and concentration in vacuo gave the primary amide **1** (4.05 g, 12.6 mmol, 90%) as a white solid. The (*S*)-enantiomer was prepared via the same procedure in 76% yield.

TLC: R_f (1) = 0.11 (silica, PE:EtOAc 1:1)

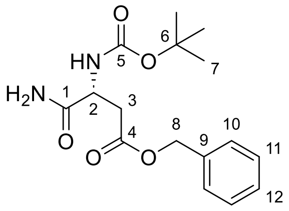

1

^{1}H-NMR (400 MHz, $CDCl_3$): δ = 7.31 – 7.42 (m, 5 H, 10-H, 11-H, 12-H), 6.46 (s, 1 H, 1-NH_2), 5.71 (d, $^3J_{NH,2}$ = 12.4 Hz, 1 H, 5-NH), 5.55 (s, 1 H, 1-NH_2), 5.17 (d, $^2J_{8',8}$ = 12.4 Hz, 1 H, 8-H‘), 5.14 (d, $^2J_{8,8'}$ = 12.4 Hz, 1 H, 8-H), 4.56 (m, 1 H, 2-H), 3.07 (d, $^2J_{3',3}$ = 17.2 Hz, $^3J_{3',2}$ = 4.5 Hz, 1 H, 3-H‘), 2.73 (d, $^2J_{3,3'}$ = 17.2 Hz, $^3J_{3,2}$ = 6.2 Hz, 1 H, 3-H), 1.46 (s, 9 H, 7-H).

^{13}C-NMR (100 MHz, $CDCl_3$): δ = 171.9 (s, C-4), 173.1 (s, C-1), 155.5 (s, C-5), 135.3 (s, C-9), 128.6 (d, C-10), 128.5 (d, C-12), 128.3 (d, C-11), 80.6 (s, C-6), 66.9 (t, C-8), 50.3 (d, C-2), 36.0 (t, C-3), 28.3 (q, C-7).

**Optical rotation:**	**(*R*)- 1:** $[\alpha]_D^{20}$= −12.5 (c = 1.0, $CHCl_3$)	
	**(*S*)- 1:** $[\alpha]_D^{20}$= +10.2 (c = 0.5, $CHCl_3$)	
**HRMS (CI):**	calculated	found
$C_{16}H_{22}O_5N_2$ $[M+H]^+$:	323.1601	323.1627
**Melting point:**	162 – 163 °C	

Benzyl (*R*)-3-((*tert*-butoxycarbonyl)amino)-3-cyanopropanoate[15] 2

According to Donohoe *et al.*[15], TFAA (3.31 mL, 23.4 mmol, 2.0 eq.) was added dropwise to a solution of the primary amide **1** (3.77 g, 11.7 mmol) and pyridine (4.73 mL, 58.5 mmol, 5.0 eq.) in anhydrous THF (80 mL) at 0 °C. The reaction mixture was stirred for 2 h before being diluted with EtOAc and washed with 1.0 M HCl_{aq}, sat. $NaHCO_3$ solution and brine. The organic layer was dried

with $MgSO_4$ and concentrated in vacuo to give the nitrile **2** (3.37 g, 11.1 mmol, 95%) as a white solid. The (*S*)-enantiomer was prepared via the same procedure in 88% yield.

TLC: R_f (2) = 0.66 (silica, PE:EtOAc 1:1)

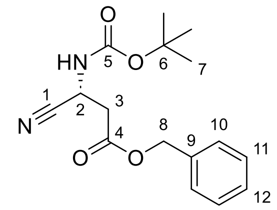

2

^{1}H-NMR (400 MHz, $CDCl_3$): δ = 7.34 – 7.44 (m, 5 H, 10-H, 11-H, 12-H), 5.57 (m, 1 H, 5-NH), 5.22 (s, 2 H, 8-H), 4.95 (m, 1 H, 2-H), 2.94 (d, $^{2}J_{3',3}$ = 17.3 Hz, $^{3}J_{3',2}$ = 5.0 Hz, 1 H, 3-H‘), 2.86 (d, $^{2}J_{3,3'}$ = 17.3 Hz, $^{3}J_{3,2}$ = 5.3 Hz, 1 H, 3-H), 1.47 (s, 9 H, 7-H).
^{13}C-NMR (100 MHz, $CDCl_3$): δ = 169.2 (s, C-4), 154.2 (s, C-5), 134.9 (s, C-9), 128.9 (d, C-10), 128.8 (d, C-12), 128.6 (d, C-11), 117.8 (s, C-1), 81.6 (s, C-6), 67.7 (t, C-8), 37.6 (t, C-3), 38.5 (d, C-2), 28.3 (q, C-7).

**Optical rotation:**	**(*R*)-2:** $[\alpha]_D^{20}$= +25.3 (c = 1.0, $CHCl_3$)	
	**(*S*)-2:** $[\alpha]_D^{20}$= −26.4 (c = 1.0, $CHCl_3$)	
**HRMS (CI):**	calculated	found
$C_{16}H_{21}O_4N_2$ $[M+H]^+$:	305.1496	305.1485
**Melting point:**	70 – 72 °C	

***tert*-Butyl (*R,Z*)-2-(3-((*tert*-butoxycarbonyl)amino)-5-oxopyrrolidin-2-ylidene)acetate[15] 3**
Preparation according to Donohoe *et al*.[15]

TLC: R_f (3) = 0.25 (silica, PE:EtOAc 7:3)

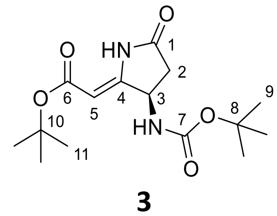

3

^{1}H-NMR (400 MHz, $CDCl_3$): δ = 9.75 (s, 1 H, 1-NH), 5.12 (m, 1 H, 5-H), 4.88 – 4.99 (m, 2 H, 3-H, 7-NH), 2.92 (dd, $^{2}J_{2,2'}$ = 16.6 Hz, $^{3}J_{2,3}$ = 7.5 Hz, 1 H, 2-H‘), 2.37 (dd, $^{2}J_{2,2'}$ = 17.0 Hz, $^{3}J_{2,3}$ = 4.7 Hz, 1 H, 2-H), 1.48 (s, 9 H, 11-H), 1.46 (s, 9 H, 9-H).
^{13}C-NMR (100 MHz, $CDCl_3$): δ = 173.9 (s, C-1), 167.4 (s, C-6), 156.5 (s, C-4), 155.0 (s, C-7), 92.5 (d, C-5), 80.8 (s, s, C-8, C-10), 48.7 (d, C-3), 36.5 (t, C-2), 28.3 (q, C-9), 28.2 (q, C-11).

**Optical rotation:**	**(*R*)-3:** $[\alpha]_D^{20}$= +74.4 (c = 1.0, $CHCl_3$)	
	**(*S*)-3:** $[\alpha]_D^{20}$= −70.1 (c = 1.0, $CHCl_3$)	
**HRMS (ESI):**	calculated	found

$C_{15}H_{25}N_2O_5$ $[M+H]^+$: 313.1758 313.1761

Melting point: 127 – 131 °C

(*R*,*Z*)-2-(3-((*tert*-Butoxycarbonyl)amino)-5-oxopyrrolidin-2-ylidene)acetic acid[15] SI-1

According to Donohoe *et al.*[15], TFA (903 µL, 11.7 mmol, 20 eq.) was added to a solution of amino pyrrolidone **3** (183 mg, 586 µmol) in CH_2Cl_2 (5.0 mL) at room temperature. The reaction mixture was stirred for 4 h at room temperature before being concentrated in vacuo by redissolving in toluene twice. The residue was dissolved in THF:H_2O (1:1, 6.0 mL) and cooled to 0 °C. After addition of $NaHCO_3$ (172 mg, 2.05 mmol) and Boc_2O (136 µL, 586 µmol, 1.0 eq.), the resulting suspension was stirred for 48 h while slowly reaching room temperature. The reaction mixture was acidified with HCl_{aq} (pH = 2 – 3) and extracted thrice with EtOAc. The combined organic extracts were dried with $MgSO_4$ and concentrated in vacuo. The crude product was purified by automated reversed phase column chromatography (C18 spherical, H_2O:MeCN 10% to 90% MeCN) to give carboxylic acid **SI-1** (46.0 mg, 180 µmol, 31%) as a light-yellow resin. The (*S*)-enantiomer was prepared via the same procedure in 30% yield. Analytical samples (for GC-FID) of the corresponding methyl esters were prepared using TMS-Diazomethane.

TLC: R_f (SI-1) = 0.20 (silica, PE:EtOAc 1:1)

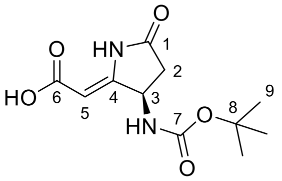

SI-1

^{1}H-NMR (500 MHz, DMSO-d$_6$): δ = 12.04 (s, 1 H, 6-OH), 9.98 (s, 1 H, 1-NH), 7.50 (d, $^3J_{NH,3}$ = 8.6 Hz, 1 H, 7-NH), 4.87 (s, 1 H, 5-H), 4.78 (ddd, $^3J_{3,2}$ = 9.6 Hz, $^3J_{3,NH}$ = 8.6 Hz, $^3J_{3,2'}$ = 6.0 Hz, 1 H, 3-H), 2.74 (dd, $^2J_{2,2'}$ = 17.6 Hz, $^3J_{2,3}$ = 9.6 Hz, 1 H, 2-H'), 2.32 (dd, $^2J_{2,2'}$ = 17.5 Hz, $^3J_{2,3}$ = 6.0 Hz, 1 H, 2-H), 1.40 (s, 9 H, 9-H).

^{13}C-NMR (125 MHz, DMSO-d$_6$): δ = 174.6 (s, C-1), 168.6 (s, C-6), 160.0 (s, C-4), 155.2 (s, C-7), 89.0 (d, C-5), 78.7 (s, C-8), 48.2 (d, C-3), 34.3 (t, C-2), 28.1 (q, C-9).

Chiral GC-FID of SI-1(OMe):

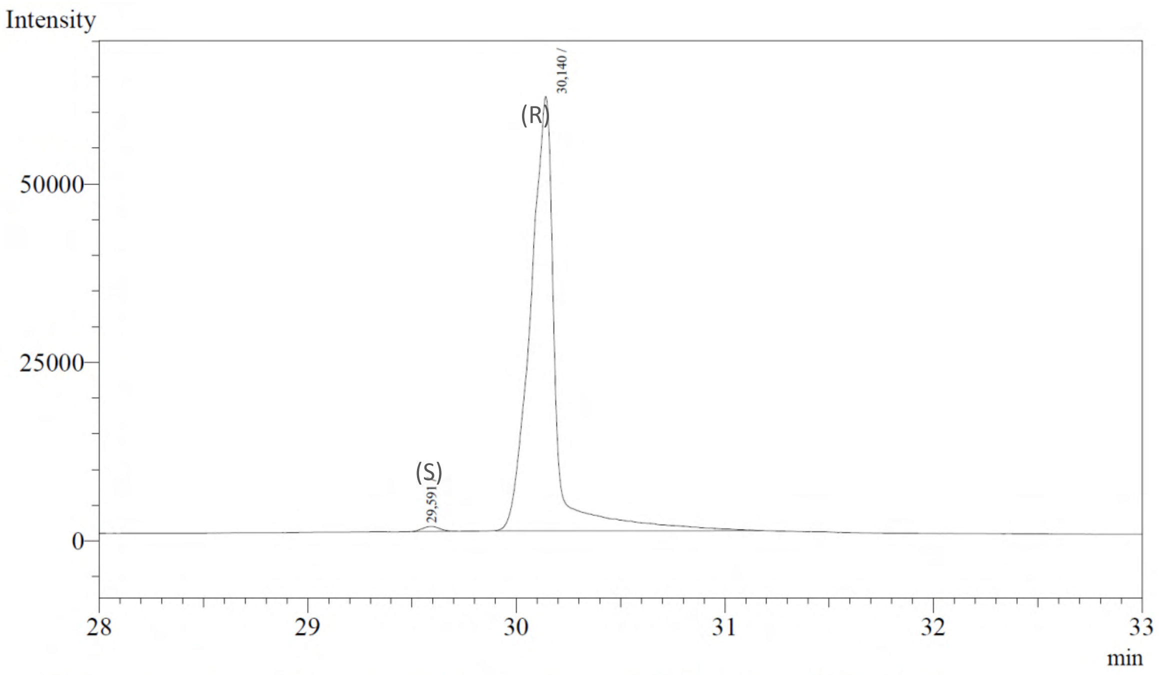

Peak#	Ret.Time	Area	Height	Conc.	Unit	Mark	ID#	Cmpd Name
1	29,591	3991	670	0,781				
2	30,140	506967	60258	99,219				
Total		510958	60928					

Chiral GC-FID of ent-SI-1(OMe):

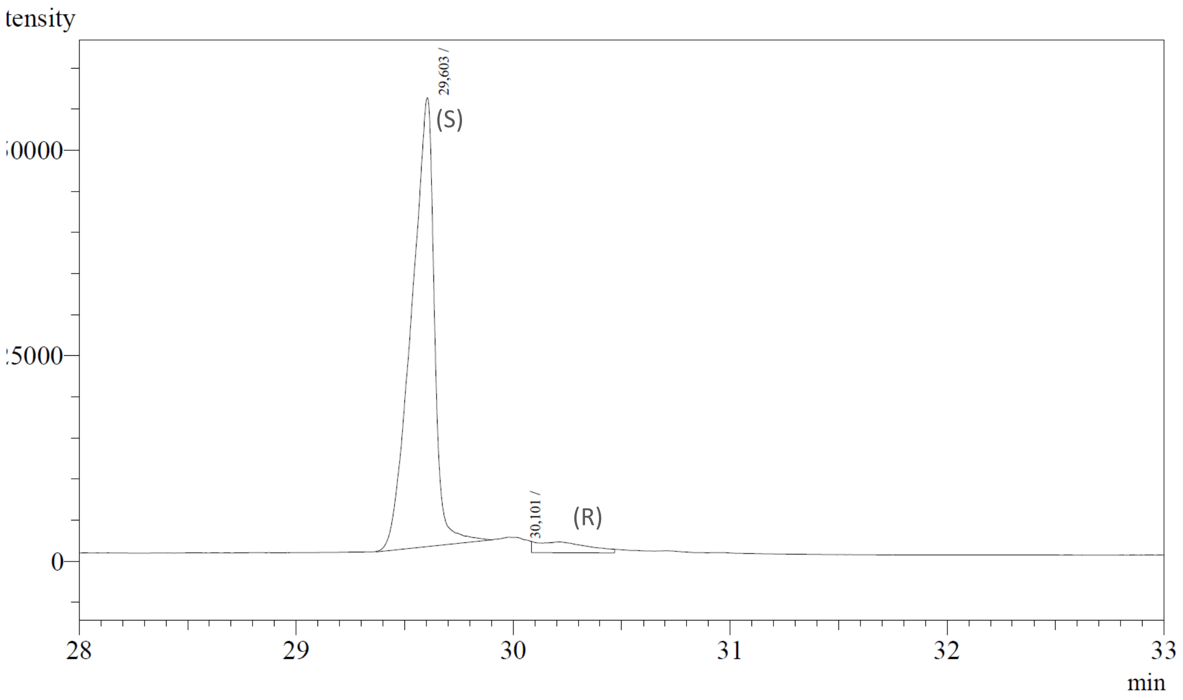

Peak#	Ret.Time	Area	Height	Conc.	Unit	Mark	ID#	Cmpd Name
1	29,603	399659	53603	94,724				
2	30,101	22260	1205	5,276				
Total		421919	54808					

Methyl (*R,Z*)-*N*-(2-(3-((*tert*-butoxycarbonyl)amino)-5-oxopyrrolidin-2-ylidene)acetyl)-*N*-methylglycinate 4

TFA (6.76 mL, 88.0 mmol, 20 eq.) was added to a solution of amino pyrrolidone **3** (1.37 g, 4.39 mmol) in CH_2Cl_2 (28 mL) at room temperature. The reaction mixture was stirred for 4 h at room temperature before being concentrated in vacuo. The residue was azeotropically dried with toluene twice. The residue was dissolved in THF:H_2O (1:1, 30 mL) and cooled to 0 °C. After addition of $NaHCO_3$ (1.29 g, 15.4 mmol, 3.5 eq.) and Boc_2O (1.32 mL, 5.70 mmol, 1.3 eq.), the resulting suspension was stirred for 72 h while slowly reaching room temperature. The reaction mixture was diluted with 5 wt% $NaHCO_3$ solution (3.0 mL) and washed twice with Et_2O. The aqueous layer was acidified with HCl_{aq} (pH = 2 – 3) and extracted thrice with Et_2O. The combined organic extracts of the last extraction step were dried with $MgSO_4$ and concentrated in vacuo to give the crude carboxylic acid (873 mg).

The above-prepared carboxylic acid (873 mg) and sarcosine methyl ester hydrochloride (951 mg, 6.81 mmol, 2.0 eq.) were dissolved in anhydrous CH_2Cl_2 (28 mL). After cooling to 0 °C, NMM (1.54 mL, 14.0 mmol, 4.1 eq.) and HBTU (1.42 g, 3.75 mmol, 1.1 eq.) were added subsequentially. The reaction mixture was stirred for 16 h while slowly reaching room temperature before being diluted with EtOAc. The mixture was washed with 1.0 M HCl_{aq}, sat. $NaHCO_3$ solution and brine. The organic layer was dried with $MgSO_4$ and concentrated in vacuo to give the dipeptide **4** (700 mg, 2.05 mmol, 47% over 2 steps) as a light-yellow foam.

TLC: R_f (4) = 0.10 (silica, CH_2Cl_2:MeOH 97:3)

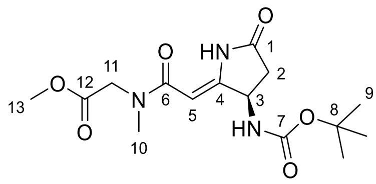

4

^{1}H-NMR (500 MHz, DMSO-d$_6$, 373 K): δ = 10.25 (s, 1 H, 1-NH), 7.14 (d, $^{3}J_{NH,3}$ = 5.7 Hz, 1 H, 7-NH), 5.42 (s, 1 H, 5-H), 4.77 (ddd, $^{3}J_{3,2}$ = 9.5 Hz, $^{3}J_{3,NH}$ = 5.7 Hz, $^{3}J_{3,2'}$ = 5.6 Hz, 1 H, 3-H), 4.16 (m, 2 H, 11-H), 3.68 (s, 3 H, 13-H), 3.00 (s, 3 H, 10-H), 2.75 (dd, $^{2}J_{2',2}$ = 17.6 Hz, $^{3}J_{2',3}$ = 9.5 Hz, 1 H, 2-H‘), 2.37 (dd, $^{2}J_{2,2'}$ = 17.6 Hz, $^{3}J_{2,3}$ = 5.6 Hz, 1 H, 2-H), 1.44 (s, 9 H, 9-H).

^{13}C-NMR (125 MHz, $CDCl_3$, 298 K): δ = 174.3 (s, C-1), 168.3 (s, C-6), 170.1 (s, C-12), 155.5 (s, C-7), 157.0 (s, C-4), 88.7 (d, C-5), 80.8 (s, C-8), 52.4 (s, C-13), 49.5 (t, C-11), 49.0 (d, C-3), 36.9 (q, C-10), 36.3 (t, C-2), 28.4 (q, C-9).

**Optical rotation:**	$[\alpha]_D^{20}$= +75.8 (c = 1.0, $CHCl_3$)	
**HRMS (ESI):**	calculated	found
$C_{15}H_{24}N_3O_6$ $[M+H]^+$:	342.1660	342.1656

Methyl (*S*)-3-((*tert*-butoxycarbonyl)amino)-2-hydroxypropanoate[204] 5

L-Isoserine (1.00 g, 9.52 mmol) was dissolved in MeOH (9.5 mL) and cooled to 0 °C. Thionyl chloride (764 µL, 10.5 mmol, 1.1 eq.) was added dropwise, and the mixture was stirred for 16 h while slowly reaching room temperature. The reaction mixture was concentrated in vacuo to give the crude H-Ise-OMe·HCl.

The above-prepared H-Ise-OMe·HCl and $NaHCO_3$ (1.76 g, 20.9 mmol, 2.2 eq.) were dissolved in THF:H_2O (1:1). After cooling to 0 °C, Boc_2O (2.04 g, 9.32 mmol, 0.98 eq.) was added, and the reaction mixture was stirred for 16 h while slowly reaching room temperature. After dilution with EtOAc, the organic layer was washed with 1.0 M HCl_{aq}, sat. $NaHCO_3$ solution and brine. The organic layer was dried with $MgSO_4$ and concentrated in vacuo to give Boc-Ise-OMe (2.04 g, 9.30 mmol, 98%) as a colorless resin.

TLC: R_f (5) = 0.42 (silica, CH_2Cl_2:MeOH 95:5)

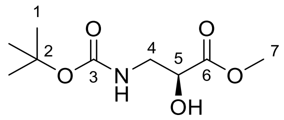

5

^{1}H-NMR (400 MHz, $CDCl_3$): δ = 4.95 (m, 1 H, 3-NH), 4.28 (t, $^3J_{5,4}$ = 4.5 Hz, 1 H, 5-H), 3.81 (s, 3 H, 7-H), 3.50 (m, 2 H, 4-H), 1.44 (s, 9 H, 1-H).

^{13}C-NMR (100 MHz, $CDCl_3$): δ = 173.6 (s, C-6), 156.2 (s, C-3), 79.9 (s, C-2), 70.3 (d, C-5), 52.8 (q, C-7), 44.0 (t, C-4), 28.3 (q, C-1).

**Optical rotation:**	$[\alpha]_D^{20}$= +23.0 (c = 1.0, $CHCl_3$)	
**HRMS (CI):**	calculated	found
$C_9H_{17}NO_5$ $[M]^+$:	219.1101	219.1107
**Melting point:**	40 – 42 °C	

Methyl (*S*)-3-((*tert*-butoxycarbonyl)amino)-2-((*tert*-butyldimethylsilyl)oxy)propanoate 6

Imidazole (559 mg, 8.21 mmol, 1.2 eq.) and TBS-Cl (1.08 g, 7.18 mmol, 1.05 eq.) were subsequently added to a 0 °C cold solution of Boc-Ise-OMe **5** (1.50 g, 6.84 mmol) in anhydrous CH_2Cl_2 (30 mL). The resulting suspension was stirred for 16 h while slowly reaching rt. After dilution with EtOAc, the reaction mixture was washed with 1.0 M HCl_{aq}, sat. $NaHCO_3$ solution and brine. The organic layer was dried with $MgSO_4$ and concentrated in vacuo. The crude was purified by automated flash chromatography (silica, CyH:EtOAc 0% to 40% EtOAc) to give the protected isoserine **6** (2.02 g, 6.06 mmol, 89%) as a colorless resin.

TLC: R_f (6) = 0.24 (silica, PE:EtOAc 8:2)

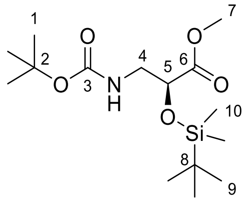

6

^{1}H-NMR (400 MHz, $CDCl_3$): δ = 4.86 (m, 1 H, 3-NH), 4.32 (dd, $^{3}J_{5,4}$ = 5.9 Hz, $^{3}J_{5,4'}$ = 5.9 Hz , 1 H, 5-H), 3.74 (s, 3 H, 7-H), 3.50 (m, 1 H, 4-H), 3.35 (ddd, $^{2}J_{4',4}$ = 13.4 Hz, $^{3}J_{4',5}$ = 6.4 Hz, $^{3}J_{4',NH}$ = 6.4 Hz, 1 H, 4-H'), 1.44 (s, 9 H, 1-H), 0.92 (s, 9 H, 9-H), 0.12 (s, 3 H, 10-H), 0.09 (s, 3 H, 10'-H).

^{13}C-NMR (100 MHz, $CDCl_3$): δ = 172.4 (s, C-6), 155.9 (s, C-3), 79.7 (s, C-2), 71.8 (d, C-5), 52.2 (q, C-7), 44.6 (t, C-4), 28.5 (q, C-1), 25.8 (q, C-9), 19.4 (s, C-8), –4.9 (q, C-10), –5.2 (q, C-10').

Optical rotation: $[\alpha]_D^{20}$= –18.8 (c = 1.0, $CHCl_3$)

**HRMS (CI):**	calculated	found
$C_{15}H_{32}O_5NSi$ $[M+H]^+$:	334.2044	334.2035

***tert*-Butyl (*S*)-(2-((*tert*-butyldimethylsilyl)oxy)-4-diazo-3-oxobutyl)carbamate 8**

Protected isoserine **6** (940 mg, 2.82 mmol) was dissolved in THF (15 mL) and cooled to 0 °C. After the addition of 0.20 M $LiOH_{aq}$ (15.5 mL, 3.10 mmol, 1.1 eq.), the solution was stirred for 1 h while slowly reaching room temperature. After full conversion, the reaction mixture was acidified with 0.2 M $KHSO_4$ solution and extracted thrice with Et_2O. The combined organic layers were dried with $MgSO_4$ and concentrated in vacuo to give the crude carboxylic acid **7**, which was immediately used in the following reaction.

Diazomethane preparation: 1-Methyl-1-nitrosourea (871 mg, 8.45 mmol, 3.0 eq.) was slowly dissolved in a mixture of Et_2O (18 mL) and KOH_{aq} (6.0 mL, 40wt%) at –15 to –10 °C. After complete solvation, H_2O (3.0 mL) was added, and the mixture was cooled to –78 °C. The ether layer was decanted in a separation funnel and washed with H_2O.

The above-prepared carboxylic acid **7** was dissolved in anhydrous THF (15 mL) and cooled to –20 °C. Triethylamine (412 µL, 2.96 mmol, 1.05 eq.) and ethyl chloroformate (284 µL, 2.96 mmol, 1.05 eq.) were added subsequently. The reaction mixture was stirred for 30 min before being cooled to –40 °C. The above-prepared diazomethane in Et_2O was added dropwise to the active ester suspension. The resulting mixture was stirred for 18 h while slowly reaching room temperature. The reaction mixture was diluted with H_2O, and the aqueous layer was extracted with Et_2O. The combined organic layers were washed with sat. $NaHCO_3$ solution, dried with $MgSO_4$ and concentrated in vacuo. The crude product was purified by column chromatography (silica, pentane:EtOAc 9:1) to give the diazoketone **8** (845 mg, 2.34 mmol, 83%) as a light-yellow solid.

TLC: R_f (8) = 0.33 (silica, pentane:EtOAc 8:2)

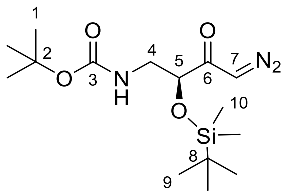

8

^{1}H-NMR (400 MHz, $CDCl_3$): δ = 5.75 (s, 1 H, 7-H), 4.82 (m, 1 H, 3-NH), 4.15 (t, $^3J_{5,4}$ = 5.7 Hz, 1 H, 5-H), 3.38 (m, 2 H, 4-H), 1.44 (s, 9 H, 1-H), 0.94 (s, 9 H, 9-H), 0.13 (s, 3 H, 10-H), 0.11 (s, 3 H, 10'-H).
^{13}C-NMR (100 MHz, $CDCl_3$): δ = 196.4 (s, C-6), 155.6 (s, C-3), 79.5 (s, C-2), 75.9 (d, C-5), 53.2 (d, C-7), 44.7 (t, C-4), 28.4 (q, C-1), 25.8 (q, C-9), 18.1 (s, C-8), –5.0 (q, C-10), –5.1 (q, C-10').

Optical rotation: $[\alpha]_D^{20}$= –66.8 (c = 1.0, $CHCl_3$)

**HRMS (ESI):**	calculated	found
$C_{15}H_{30}N_3O_4Si$ $[M+H]^+$:	344.2000	344.2000

Methyl (*R*)-4-((*tert*-butoxycarbonyl)amino)-3-((*tert*-butyldimethylsilyl)oxy)butanoate 9

Diazoketone **8** (23.5 mg, 68.0 µmol) was dissolved in anhydrous MeOH (1.4 mL). The resulting yellow solution was irradiated with a blue LED (405 nm, 18 W) for 2 h at room temperature. The colorless solution was concentrated in vacuo, and the residue was purified by column chromatography (silica, pentane:EtOAc 8:2) to give GABOB-derivative **9** (22.0 mg, 63.3 µmol, 93%) as a colorless resin.

TLC: R_f (9) = 0.53 (silica, PE:EtOAc 6:4)

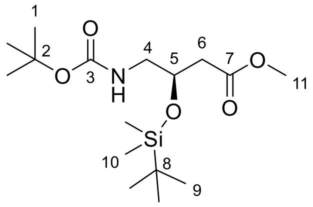

9

^{1}H-NMR (400 MHz, $CDCl_3$): δ = 4.76 (m, 1 H, 3-NH), 4.23 (tt, $^3J_{5,4}$ = 5.6 Hz, $^3J_{5,6}$ = 5.6 Hz, 1 H, 5-H), 3.68 (s, 3 H, 11-H), 3.22 (m, 2 H, 4-H), 2.47 (m, 2 H, 6-H), 1.44 (s, 9 H, 1-H), 0.87 (s, 9 H, 9-H), 0.09 (s, 3 H, 10-H), 0.05 (s, 3 H, 10'-H).
^{13}C-NMR (100 MHz, $CDCl_3$): δ = 171.8 (s, C-7), 156.1 (s, C-3), 79.5 (s, C-2), 68.5 (d, C-5), 51.8 (q, C-11), 46.2 (t, C-4), 40.3 (t, C-6), 28.5 (q, C-1), 25.8 (q, C-9), 18.1 (s, C-8), –4.6 (q, C-10), –4.9 (q, C-10').

Optical rotation: $[\alpha]_D^{20}$= +17.6 (c = 1.0, $CHCl_3$)

**HRMS (CI):**	calculated	found
$C_{16}H_{34}NO_5Si$ $[M+H]^+$:	348.2201	348.2200

(((3a*S*,5*R*,6*S*,6a*S*)-6-Azido-2,2-dimethyltetrahydrofuro[2,3-d][1,3]dioxol-5-yl)methoxy)(*tert*-butyl)dimethylsilane 11a

Triflic anhydride (4.51 mL, 26.7 mmol, 1.5 eq.) was added dropwise to a –15 °C cold solution of monoacetonide **10** (5.42 g, 17.8 mmol), and pyridine (4.32 mL, 53.4 mmol, 3.0 eq.) in anhydrous CH_2Cl_2 (60 mL). The reaction mixture was stirred for 1 h at –10 °C. After full conversion, the mixture was diluted with CH_2Cl_2 and washed with 1.0 M HCl_{aq} and brine. The organic layer was dried with $MgSO_4$ and concentrated in vacuo to give the crude triflate.

The above-prepared crude triflate and tetrabutylammonium hydrogen sulfate (30.0 mg, 89.0 µmol, 0.5 mol%) were dissolved in anhydrous DMF (90 mL). After the addition of NaN_3 (5.79 g, 89.0 mmol, 5.0 eq.), the suspension was stirred for 24 h at room temperature. The reaction mixture was diluted with EtOAc and washed with 5wt% $LiCl_{aq}$, 1.0 M HCl_{aq}, sat. $NaHCO_3$ solution and brine. The organic layer was dried over $MgSO_4$ and concentrated in vacuo. The crude was purified by column chromatography (silica, PE:EtOAc 97:3) to give the azido-sugar **11a** (2.80 g, 8.50 mmol, 48%) as a colorless oil.

TLC: R_f (11a) = 0.53 (silica, PE:EtOAc 8:2)

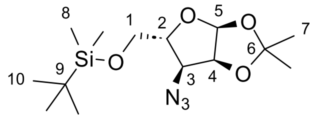

11a

^{1}H-NMR (400 MHz, $CDCl_3$): δ = 5.79 (d, $^3J_{5,4}$ = 3.8 Hz, 1 H, 5-H), 4.73 (dd, $^3J_{4,3}$ = 4.2 Hz, $^3J_{4,5}$ = 4.2 Hz, 1 H, 4-H), 4.11 (ddd, $^3J_{2,3}$ = 9.4 Hz, $^3J_{2,1}$ = 2.8 Hz, $^3J_{2,1'}$ = 2.8 Hz, 1 H, 2-H), 3.95 (dd, $^2J_{1',1}$ = 11.9 Hz, $^3J_{1',2}$ = 2.7 Hz, 1 H, 1-H'), 3.82 (dd, $^2J_{1,1'}$ = 12.0 Hz, $^3J_{1,2}$ = 2.8 Hz, 1 H, 1-H), 3.61 (dd, $^3J_{3,2}$ = 9.3 Hz, $^3J_{3,4}$ = 4.6 Hz, 1 H, 3-H), 1.58 (s, 3 H, 7'-H), 1.37 (s, 3 H, 7-H), 0.91 (s, 9 H, 10-H), 0.09 (s, 3 H, 8'-H), 0.08 (s, 3 H, 8-H).

^{13}C-NMR (100 MHz, $CDCl_3$): δ = 113.2 (s, C-6), 104.2 (d, C-5), 80.3 (d, C-4), 78.6 (d, C-2), 61.2 (d, C-3), 60.2 (t, C-1), 26.6 (q, C-7), 26.0 (q, C-10), 18.5 (s, C-9), –5.3 (q, C-8), –5.2 (q, C-8').

Optical rotation: $[\alpha]_D^{20}$= –88.7 (c = 1.0, $CHCl_3$)

**HRMS (CI):**	calculated	found
$C_{14}H_{28}NO_4Si$ $[M-N_2+H]^+$:	302.1782	302.1811

(((3a*S*,5*S*,6*S*,6a*S*)-6-Azido-2,2-dimethyltetrahydrofuro[2,3-d][1,3]dioxol-5-yl)methoxy)(*tert*-butyl)dimethylsilane 11b

Triflic anhydride (8.32 mL, 49.3 mmol, 1.5 eq.) was added dropwise to a –15 °C cold solution of monoacetonide **10b** (10.0 g, 32.8 mmol), and pyridine (7.97 mL, 99.0 mmol, 3.0 eq.) in anhydrous CH_2Cl_2 (164 mL). The reaction mixture was stirred for 1 h at –10 °C. After full conversion, the mixture was poured into ice water, and the aqueous layer was extracted twice with EtOAc. The combined organic layers were washed with 1.0 M HCl_{aq} and brine. The organic layer was dried with $MgSO_4$ and concentrated in vacuo to give the crude triflate.

The above-prepared crude triflate was dissolved in anhydrous DMF (120 mL). After the addition of NaN_3 (10.7 g, 164 mmol, 5.0 eq.), the suspension was stirred for 2 h at 100 °C. The reaction mixture was diluted with EtOAc and washed with 5wt% $LiCl_{aq}$, 1.0 M HCl_{aq}, sat. $NaHCO_3$ solution and brine. The organic layer was dried over $MgSO_4$ and concentrated in vacuo. The crude was purified by automated flash chromatography (silica, CyH:EtOAc 0% to 20%) to give the azido-sugar **11b** (7.79 g, 23.6 mmol, 72%) as a colorless oil.

TLC: R_f (11b) = 0.36 (silica, PE:EtOAc 8:2)

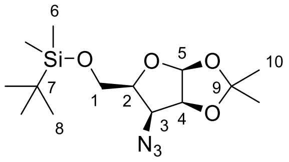

11b

^{1}H-NMR (500 MHz, $CDCl_3$): δ = 5.76 (d, $^3J_{5,4}$ = 4.2 Hz, 1 H, 5-H), 4.77 (dd, $^3J_{4,3}$ = 5.6 Hz, $^3J_{4,5}$ = 4.2 Hz, 1 H, 4-H), 4.17 (ddd, $^3J_{2,3}$ = 6.6 Hz, $^3J_{2,1'}$ = 6.6 Hz, $^3J_{2,1}$ = 5.5 Hz, 1 H, 2-H), 4.12 (dd, $^3J_{3,2}$ = 6.7 Hz, $^3J_{3,4}$ = 5.5 Hz, 1 H, 3-H), 4.05 (dd, $^2J_{1',1}$ = 10.4 Hz, $^3J_{1',2}$ = 6.5 Hz, 1 H, 1-H'), 3.83 (dd, $^2J_{1,1'}$ = 10.4 Hz, $^3J_{1,2}$ = 5.5 Hz, 1 H, 1-H), 1.62 (s, 3 H, 10'-H), 1.37 (s, 3 H, 10-H), 0.91 (s, 9 H, 8-H), 0.10 (s, 3 H, 6'-H), 0.10 (s, 3 H, 6-H).

^{13}C-NMR (125 MHz, $CDCl_3$): δ = 114.5 (s, C-9), 105.0 (d, C-5), 81.8 (d, C-2), 80.4 (d, C-4), 62.8 (t, C-1), 61.7 (d, C-3), 26.6 (q, C-10'), 26.6 (q, C-10), 26.0 (q, C-8), 18.5 (s, C-7), –5.19 (q, C-6), –5.24 (q, C-6').

**Optical rotation:**	$[\alpha]_D^{20}$= –53.2 (c = 1.0, $CHCl_3$)	
**HRMS (ESI):**	calculated	found
$C_{14}H_{27}N_3O_4SiNa$ $[M+Na]^+$:	352.1663	352.1660

(2*R*,3*S*,4*R*,5*R*)-2-Allyl-4-azido-5-(((*tert*-butyldimethylsilyl)oxy)methyl)tetrahydrofuran-3-ol 12a

In a 100 mL Schlenk flask under an atmosphere of nitrogen, azido-sugar **11a** (1.17 g, 3.46 mmol) and allyltrimethylsilane (2.20 mL, 13.9 mmol, 4.0 eq.) were dissolved in anhydrous CH_2Cl_2 (35 mL). After cooling to –20 °C, $TiCl_4$ (759 µL, 6.92 mmol, 2.0 eq.) was added dropwise, and the stirring continued for 20 min. The reaction mixture was quenched by the addition of sat. $NaHCO_3$ solution (3.5 mL). After pouring into more sat. $NaHCO_3$ solution, the aqueous phase was extracted once with CH_2Cl_2. The combined organic layers were washed with brine, dried with $MgSO_4$, and concentrated in vacuo. The crude was purified by column chromatography (silica, PE:EtOAc 9:1) to give C-furanoside **12a** (866 mg, 2.76 mmol, 80%, dr 97:3) as a colorless oil.

TLC: R_f (12a) = 0.23 (silica, PE:EtOAc 9:1)

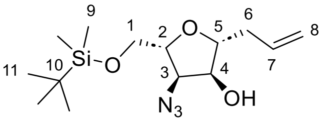

12a

^{1}H-NMR (500 MHz, DMSO-d$_6$): δ = 5.80 (ddt, $^3J_{7,8'}$ = 17.1 Hz, $^3J_{7,8}$ = 10.2 Hz, $^3J_{7,6}$ = 6.9 Hz, 1 H, 7-H), 5.65 (d, $^3J_{OH,4}$ = 5.6 Hz, 1 H, 4-OH), 5.08 (ddt, $^3J_{8',7}$ = 17.2 Hz, $^2J_{8',8}$ = 2.2 Hz, $^4J_{8',6}$ = 1.4 Hz, 1 H, 8-H′), 5.01 (ddt, $^2J_{8,8'}$ = 2.3 Hz, $^3J_{8,7}$ = 10.2 Hz, $^4J_{8,6}$ = 1.2 Hz, 1 H, 8-H), 3.93 (ddd, $^3J_{4,3}$ = 5.6 Hz, $^3J_{4,5}$ = 5.6 Hz, $^3J_{4,OH}$ = 5.6 Hz, 1 H, 4-H), 3.76 – 3.70 (m, 2 H, 2-H, 3-H), 3.66 (ddd, $^3J_{5,6}$ = 7.2 Hz, $^3J_{5,4}$ = 5.9 Hz, $^3J_{5,6'}$ = 4.9 Hz, 1 H, 5-H), 3.62 (d, $^3J_{1,2}$ = 3.4 Hz, 2 H, 1-H), 2.31 (dddt, $^2J_{6',6}$ = 14.4 Hz, $^3J_{6',7}$ = 6.5 Hz, $^3J_{6',5}$ = 4.9 Hz, $^4J_{6',8}$ = 1.4 Hz, 1 H, 6-H′), 2.14 (dddt, $^2J_{6,6'}$ = 14.4 Hz, $^3J_{6,7}$ = 7.1 Hz, $^3J_{6,5}$ = 7.1 Hz, $^4J_{6,8}$ = 1.3 Hz, 1 H, 6-H), 0.87 (s, 9 H, 11-H), 0.09 (s, 3 H, 9′-H), 0.08 (s, 3 H, 9-H).

^{13}C-NMR (100 MHz, DMSO-d$_6$): δ = 134.8 (d, C-7), 116.9 (t, C-8), 81.9 (d, C-5), 80.8 (d, C-2), 74.8 (d, C-4), 63.2 (t, C-1), 62.5 (d, C-3), 37.2 (t, C-6), 25.8 (q, C-11), 18.0 (s, C-10), –5.4 (q, C-9′), –5.5 (q, C-9).

**Optical rotation:**	$[\alpha]_D^{20}$= –8.0 (c = 1.0, $CHCl_3$)	
**HRMS (CI):**	calculated	found
$C_{14}H_{28}NO_3Si$ [M-N$_2$+H]$^+$:	286.1833	286.1838

(2*S*,3*S*,4*R*,5*R*)-2-Allyl-4-azido-5-(((*tert*-butyldimethylsilyl)oxy)methyl)tetrahydrofuran-3-ol 12b

In a 25 mL Schlenk tube under an atmosphere of nitrogen, allyltrimethylsilane (965 µL, 6.07 mmol, 4.0 eq.) was added to a 0 °C cold solution of compound **11a** (500 mg, 1.52 mmol) in anhydrous CH_2Cl_2 (10 mL). After stirring for 10 min, $BF_3{\cdot}OEt_2$ (801 µL, 3.04 mmol, 2.0 eq.) was added, and the stirring continued for 30 min. The cooling bath was removed, and the stirring continued for 3 h at room temperature. Another portion of allyltrimethylsilane (965 µL, 6.07 mmol, 4.0 eq.) and $BF_3{\cdot}OEt_2$ (801 µL, 3.04 mmol, 2.0 eq.) were added, and the mixture was stirred for another 2 h at room temperature. The reaction mixture was quenched with sat. $NaHCO_3$ solution (20 mL) and brine (2.0 mL) before being extracted three times with CH_2Cl_2. The combined organic layers were washed with brine, dried with $MgSO_4$, and concentrated in vacuo. The residue was dissolved in anhydrous DMF (1.5 mL) before adding imidazole (124 mg, 1.82 mmol, 1.2 eq.) and TBS-Cl (137 mg, 911 µmol, 0.6 eq.) at room temperature. After stirring for 24 h, the reaction mixture was diluted with EtOAc and washed with 5wt% $LiCl_{aq}$, 1.0 M HCl_{aq}, sat. $NaHCO_3$ solution and brine. The organic layer was dried over $MgSO_4$ and concentrated in vacuo. The crude product was purified by automated flash chromatography (silica, CyH:EtOAc 0% to 20% EtOAc) to give C-furanoside **12b** (220 mg, 702 µmol, 46%, dr 99:1) and compound **13a** (70.0 mg, 164 µmol, 11%, dr 96:4) as a colorless oil.

TLC: R$_f$ (12b) = 0.35 (silica, pentane:EtOAc 8:2)

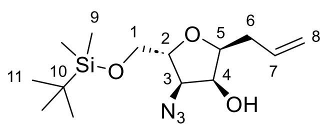

12b

^{1}H-NMR (500 MHz, DMSO-d$_6$): δ = 5.77 (ddt, $^{3}J_{7,8'}$ = 17.2 Hz, $^{3}J_{7,8}$ = 10.2 Hz, $^{3}J_{7,6}$ = 6.9 Hz, 1 H, 7-H), 5.61 (d, $^{3}J_{OH,4}$ = 5.8 Hz, 1 H, 4-OH), 5.09 (ddt, $^{3}J_{8',7}$ = 17.2 Hz, $^{2}J_{8',8}$ = 2.2 Hz, $^{4}J_{8',6}$ = 1.5 Hz, 1 H, 8-H'), 5.00 (ddt, $^{3}J_{8,7}$ = 10.2 Hz, $^{2}J_{8,8'}$ = 2.2 Hz, $^{4}J_{8,6}$ = 1.1 Hz, 1 H, 8-H), 4.15 (ddd, $^{3}J_{4,OH}$ = 5.8 Hz, $^{3}J_{4,3}$ = 4.3 Hz, $^{3}J_{4,5}$ = 2.9 Hz, 1 H, 4-H), 4.04 (dd, $^{3}J_{3,4}$ = 4.6 Hz, $^{3}J_{3,2}$ = 3.6 Hz, 1 H, 3-H), 3.89 (ddd, $^{3}J_{2,1'}$ = 8.1 Hz, $^{3}J_{2,1}$ = 3.9 Hz, $^{3}J_{2,3}$ = 3.9 Hz, 1 H, 2-H), 3.69 (dd, $^{2}J_{1,1'}$ = 11.2 Hz, $^{3}J_{1,2}$ = 3.9 H, 1 H, 1-H), 3.67 – 3.62 (m, 2 H, 5-H, 1-H'), 2.27 (m, 2 H, 6-H), 0.86 (s, 9 H, 11-H), 0.05 (s, 3 H, 9'-H), 0.04 (s, 3 H, 9-H).

^{13}C-NMR (125 MHz, DMSO-d$_6$): δ = 135.2 (d, C-7), 116.7 (t, C-8), 80.8 (d, C-5), 78.2 (d, C-2), 72.6 (d, C-4), 63.3 (t, C-1), 62.5 (d, C-3), 33.9 (t, C-6), 25.8 (q, C-11), 18.0 (s, C-10), –5.35 (q, C-9'), –5.40 (q, C-9).

**Optical rotation:**	$[\alpha]_D^{20}$= +18.4 (c = 1.0, CHCl$_3$)	
**HRMS (CI):**	calculated	found
$C_{14}H_{28}N_3O_3Si$ [M+H]$^+$:	314.1894	314.1893

(2*R*,3*S*,4*R*,5*S*)-2-Allyl-4-azido-5-(((*tert*-butyldimethylsilyl)oxy)methyl)tetrahydrofuran-3-ol 12c

In a 100 mL Schlenk flask under an atmosphere of nitrogen, azido-sugar **11b** (2.81 g, 8.19 mmol) and allyltrimethylsilane (5.22 mL, 32.8 mmol, 4.0 eq.) were dissolved in anhydrous CH_2Cl_2 (55 mL). After cooling to –20 °C, $TiCl_4$ (1.81 mL, 16.4 mmol, 2.0 eq.) was added dropwise, and the stirring continued for 15 min. The reaction mixture was quenched by the addition of sat. $NaHCO_3$ solution (10 mL). After pouring into more sat. $NaHCO_3$ solution, the aqueous layer was extracted twice with CH_2Cl_2. The combined organic layers were washed with brine, dried with $MgSO_4$, and concentrated in vacuo. The crude was purified by column chromatography (silica, PE:EtOAc 9:1) to give C-furanoside **12c** (2.05 g, 6.54 mmol, 80%, dr > 99:1) as a colorless oil.

TLC: R$_f$ (12c) = 0.25 (silica, PE:EtOAc 9:1)

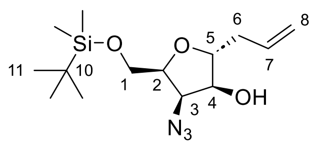

12c

^{1}H-NMR (500 MHz, DMSO-d$_6$): δ = 5.80 (ddt, $^{3}J_{7,8'}$ = 17.2 Hz, $^{3}J_{7,8}$ = 10.2 Hz, $^{3}J_{7,6}$ = 6.9 Hz, 1 H, 7-H), 5.65 (d, $^{3}J_{OH,4}$ = 5.6 Hz, 1 H, 4-OH), 5.08 (ddt, $^{3}J_{8',7}$ = 17.3 Hz, $^{2}J_{8',8}$ = 2.3 Hz, $^{4}J_{8',6}$ = 1.5 Hz, 1 H, 8-H'), 5.03 (ddt, $^{3}J_{8,7}$ = 10.2 Hz, $^{2}J_{8,8'}$ = 2.3 Hz, $^{4}J_{8,6}$ = 1.1 Hz, 1 H, 8-H), 4.10 (ddd, $^{3}J_{4,5}$ = 8.2 Hz, $^{3}J_{4,3}$ = 5.2 Hz, $^{3}J_{4,OH}$ = 5.2 Hz, 1 H, 4-H), 4.04 (dd, $^{3}J_{3,4}$ = 4.7 Hz, $^{3}J_{3,2}$ = 3.6 Hz, 1 H, 3-H), 3.98 (ddd, $^{3}J_{2,1}$ = 6.4 Hz, $^{3}J_{2,1'}$ = 6.4 Hz, $^{3}J_{2,3}$ = 3.8 Hz, 1 H, 2-H), 3.68 (dd, $^{2}J_{1,1'}$ = 10.3 Hz, $^{3}J_{1,2}$ = 6.5 H, 1 H, 1-H), 3.65 (m, 1 H,

5-H), 3.59 (dd, ${}^2J_{1',1}$ = 10.3 Hz, ${}^3J_{1',2}$ = 6.2 H, 1 H, 1-H‘), 2.34 (m, 1 H, 6-H‘), 2.16 (dddt, ${}^2J_{6,6'}$ = 14.5 Hz, ${}^3J_{6,7}$ = 7.3 Hz, ${}^3J_{6,5}$ = 7.3 Hz, ${}^4J_{6,8}$ = 1.3 Hz, 1 H, 6-H), 0.86 (s, 9 H, 11-H), 0.05 (s, 3 H, 9'-H), 0.04 (s, 3 H, 9-H).

^{13}C-NMR (125 MHz, DMSO-d$_6$): δ = 134.9 (d, C-7), 116.9 (t, C-8), 79.5 (d, C-5), 78.1 (d, C-2), 76.0 (d, C-4), 64.9 (d, C-3), 62.2 (t, C-1), 37.1 (t, C-6), 25.7 (q, C-11), 17.9 (s, C-10), –5.3 (q, C-9‘), –5.5 (q, C-9).

Optical rotation: $[\alpha]_D^{20}$= –3.4 (c = 2.0, $CHCl_3$)

**HRMS (CI):**	calculated	found
$C_{14}H_{28}O_3N_3Si$ [M+H]$^+$:	314.1894	314.1900

(2*S*,3*S*,4*R*,5*S*)-2-Allyl-4-azido-5-(((*tert*-butyldimethylsilyl)oxy)methyl)tetrahydrofuran-3-ol 12d

In a 250 mL Schlenk-tube under an atmosphere of nitrogen, allyltrimethylsilane (9.65 mL, 60.7 mmol, 4.0 eq.) was added to a –10 °C cold solution of compound **11b** (5.00 g, 15.2 mmol) in anhydrous CH_2Cl_2 (150 mL). After stirring for 10 min, $BF_3{\cdot}OEt_2$ (8.01 mL, 30.4 mmol, 2.0 eq.) was added, and the stirring continued for 30 min. The cooling bath was removed, and the stirring continued for 2 h at room temperature. The reaction mixture was quenched with sat. $NaHCO_3$ solution (200 mL) and brine (20 mL) before being extracted three times with CH_2Cl_2. The combined organic layers were washed with brine, dried with $MgSO_4$, and concentrated in vacuo. The residue was dissolved in anhydrous DMF (15 mL) before adding imidazole (2.07 g, 30.4 mmol, 2.0 eq.) and TBS-Cl (2.29 g, 15.2 mmol, 1.0 eq.) at room temperature. After stirring for 24 h, the reaction mixture was diluted with EtOAc and washed with 5wt% $LiCl_{aq}$, 1.0 M HCl_{aq}, sat. $NaHCO_3$ solution and brine. The organic layer was dried over $MgSO_4$ and concentrated in vacuo. The crude product was purified by automated flash chromatography (silica, CyH:EtOAc 0% to 30% EtOAc) to give C-furanoside **12d** (1.05 g, 3.35 mmol, 22%, dr 95:5) and compound **13c** (1.43 g, 3.34 mmol, 22%, dr 95:5) as a colorless oil.

TLC: R$_f$ (12d) = 0.37 (silica, pentane:EtOAc 85:15)

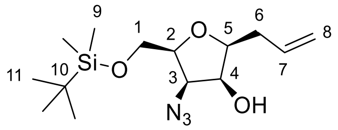

12d

^{1}H-NMR (500 MHz, $CDCl_3$): δ = 5.85 (ddt, ${}^3J_{7,8'}$ = 17.1 Hz, ${}^3J_{7,8}$ = 10.1 Hz, ${}^3J_{7,6}$ = 7.0 Hz, 1 H, 7-H), 5.16 (ddt, ${}^3J_{8',7}$ = 17.1 Hz, ${}^2J_{8',8}$ = 1.7 Hz, ${}^4J_{8',6}$ = 1.6 Hz, 1 H, 8-H'), 5.09 (ddt, ${}^3J_{8,7}$ = 10.2 Hz, ${}^2J_{8,8'}$ = 2.1 Hz, ${}^4J_{8,6}$ = 1.1 Hz, 1 H, 8-H), 4.52 (d, ${}^3J_{OH,4}$ = 11.7 Hz, 1 H, 4-OH), 4.30 (dd, ${}^3J_{3,2}$ = 9.0 Hz, ${}^3J_{3,4}$ = 4.7 Hz, 1 H, 3-H), 4.14 (ddd, ${}^3J_{2,3}$ = 9.0 Hz, ${}^3J_{2,1}$ = 3.1 Hz, ${}^3J_{2,1'}$ = 1.4 Hz, 1 H, 2-H), 4.11 (ddd, ${}^3J_{4,OH}$ = 11.6 Hz, ${}^3J_{4,3}$ = 4.7 Hz, ${}^3J_{4,5}$ = 2.8 Hz, 1 H, 4-H), 3.82 (ddd, ${}^3J_{5,6'}$ = 7.4 Hz, ${}^3J_{5,6}$ = 6.5 Hz, ${}^3J_{5,4}$ = 2.8 Hz, 1 H, 5-H), 3.74 (dd, ${}^2J_{1,1'}$ = 11.2 Hz, ${}^3J_{1,2}$ = 3.1 H, 1 H, 1-H), 3.69 (dd, ${}^2J_{1',1}$ = 11.2 Hz, ${}^3J_{1',2}$ = 1.4 H, 1 H, 1-H‘), 2.47 (dddt, ${}^2J_{6,6'}$ = 14.5 Hz, ${}^3J_{6,7}$ = 6.6 Hz, ${}^3J_{6,5}$ = 6.6 Hz, ${}^4J_{6,8}$ = 1.4 Hz, 1 H, 6-H), 2.39 (dddt, ${}^2J_{6',6}$ = 14.7 Hz, ${}^3J_{6',7}$ = 7.4 Hz, ${}^3J_{6',5}$ = 7.4 Hz, ${}^4J_{6',8}$ = 1.3 Hz, 1 H, 6-H‘), 0.95 (s, 9 H, 11-H), 0.15 (s, 6 H, 9-H).

^{13}C-NMR (125 MHz, $CDCl_3$): δ = 134.4 (d, C-7), 117.5 (t, C-8), 81.2 (d, C-5), 78.1 (d, C-2), 71.4 (d, C-4), 64.2 (d, C-3), 61.9 (t, C-1), 34.0 (t, C-6), 25.9 (q, C-11), 18.6 (s, C-10), –5.5 (q, C-9‘), –5.6 (q, C-9).

Optical rotation: $[\alpha]_D^{20}$= +56.6 (c = 1.0, $CHCl_3$)

**HRMS (ESI):**	calculated	found
$C_{14}H_{28}N_3O_3Si$ $[M+H]^+$:	314.1894	314.1895

2-((2*R*,3*S*,4*R*,5*R*)-4-Azido-5-(((*tert*-butyldimethylsilyl)oxy)methyl)-3-hydroxytetrahydrofuran-2-yl)acetaldehyde SI-2

In a 25 mL 2-neck flask, a solution of C-furanoside **12a** (90.0 mg, 287 µmol) in anhydrous CH_2Cl_2 (8.0 mL) was cooled to –78 °C. The solution was saturated with ozone until the reaction mixture turned blue (10 s). After stirring for another 30 s, the solution was degassed with N_2. Triphenylphosphine (75.0 mg, 287 µmol, 1.0 eq.) was added at –78 °C before the cooling bath was removed, and the stirring continued for 20 min. The reaction mixture was concentrated in vacuo, and the residue was purified by column chromatography (silica, PE:EtOAc 75:25) to give the aldehyde **SI-2** (42.0 mg, 133 µmol, 46%) as a colorless resin.

TLC: R_f (SI-2) = 0.45 (silica, pentane:EtOAc 6:4)

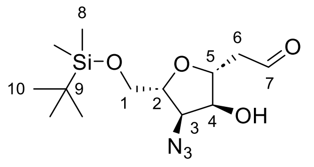

SI-2

^{1}H-NMR (400 MHz, DMSO-d_6): δ = 9.65 (dd, $^3J_{7,6}$ = 3.0 Hz, $^3J_{7,6'}$ = 1.5 Hz, 1 H, 7-H), 5.78 (d, $^3J_{OH,4}$ = 5.5 Hz, 1 H, 4-OH), 4.11 (ddd, $^3J_{5,6}$ = 8.4 Hz, $^3J_{5,4}$ = 6.5 Hz, $^3J_{5,6'}$ = 4.0 Hz, 1 H, 5-H), 4.02 (m, 1 H, 4-H), 3.83 (dd, $^3J_{3,4}$ = 5.6 Hz, $^3J_{3,2}$ = 4.3 Hz, 1 H, 3-H), 3.77 (dt, $^3J_{2,3}$ = 4.0 Hz, $^3J_{2,1}$ = 4.0 Hz, 1 H, 2-H), 3.61 (m, 2 H, 1-H), 2.70 (ddd, $^2J_{6',6}$ = 16.2 Hz, $^3J_{6',5}$ = 4.0 Hz, $^3J_{6',7}$ = 1.5 Hz, 1 H, 6-H‘), 2.44 (ddd, $^2J_{6,6'}$ = 16.0 Hz, $^3J_{6,5}$ = 8.7 Hz, $^3J_{6,7}$ = 2.4 Hz, 1 H, 6-H), 0.87 (s, 9 H, 10-H), 0.04 (s, 3 H, 8‘-H), 0.03 (s, 3 H, 8-H).

^{13}C-NMR (100 MHz, DMSO-d_6): δ = 201.5 (d, C-7), 81.5 (d, C-2), 77.2 (d, C-5), 75.2 (d, C-4), 63.2 (t, C-1), 62.5 (d, C-3), 46.7 (t, C-6), 25.8 (q, C-10), 17.9 (s, C-9), –5.4 (q, C-8‘), –5.5 (q, C-8).

Optical rotation: $[\alpha]_D^{20}$= –80.3 (c = 0.5, $CHCl_3$)

**HRMS (CI):**	calculated	found
$C_{13}H_{26}NO_4Si$ $[M-N_2+H]^+$:	288.1626	288.1631

(3a*S*,5*R*,6*S*,6a*S*)-6-Azido-5-(((*tert*-butyldimethylsilyl)oxy)methyl)tetrahydrofuro[3,2-b]furan-2(3*H*)-one SI-3

In a 25 mL 2-neck flask, a solution of C-furanoside **12b** (85.0 mg, 271 µmol) in anhydrous CH_2Cl_2 (6.0 mL) was cooled to –78 °C. The solution was saturated with ozone until the reaction mixture

turned blue (10 s). After stirring for another 30 s, the solution was degassed with N_2. Dimethylsulfide (201 µL, 2.71 mmol, 10 eq.) was added at −70 °C and the reaction mixture was stirred for 18 h while slowly reaching room temperature. The reaction mixture was concentrated in vacuo, and the residue was purified by automated flash chromatography (silica, CyH:EtOAc 0% to 40% EtOAc) to give the hemiacetal (68.0 mg, 216 µmol, 79%) as a colorless resin.

The above-prepared hemiacetal (50.0 mg, 159 µmol) was dissolved in MeCN (1.2 mL). A solution of NaH_2PO_4 (3.8 mg, 31.7 µmol, 0.2 eq.) in H_2O (100 µL) and H_2O_2 (16.0 µL, 159 µmol, 30wt%, 1.0 eq.) were subsequently added. After cooling to 0 °C, a solution of $NaClO_2$ (28.7 mg, 254 µmol, 1.6 eq.) in H_2O (100 µL) was added dropwise. The resulting solution was stirred for 16 h at room temperature. After the addition of Na_2SO_4 (15 mg) and brine (2.0 mL), the mixture was extracted thrice with EtOAc. The combined organic layers were dried with $MgSO_4$ and concentrated in vacuo. The residue was dissolved in anhydrous CH_2Cl_2 (1.0 mL) before pTsOH (3.0 mg, 15.8 µmol, 10mol%) was added. The resulting solution was stirred for 2 h at room temperature. After concentration in vacuo, the crude was purified by automated reversed phase column chromatography (C18 spherical, H_2O:MeCN 10% to 90% MeCN) to give the lactone **SI-3** (32.3 mg, 103 µmol, 65%) as a colorless resin.

TLC: R_f (SI-3) = 0.17 (silica, pentane:EtOAc 8:2)

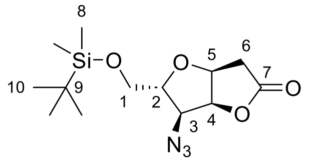

SI-3

^{1}H-NMR (500 MHz, $CDCl_3$): δ = 5.09 (dd, $^3J_{4,5}$ = 4.6 Hz, $^3J_{4,3}$ = 4.6 Hz, 1 H, 4-H), 4.86 (ddd, $^3J_{5,6}$ = 6.1 Hz, $^3J_{5,4}$ = 4.5 Hz, $^3J_{5,6'}$ = 1.4 Hz, 1 H, 5-H), 4.04 (dd, $^3J_{3,2}$ = 8.0 Hz, $^3J_{3,4}$ = 4.7 Hz, 1 H, 3-H), 4.04 (dt, $^3J_{2,3}$ = 8.0 Hz, $^3J_{2,1}$ = 2.6 Hz, 1 H, 2-H), 3.89 (dd, $^2J_{1,1'}$ = 11.8 Hz, $^3J_{1,2}$ = 2.5 Hz, 1 H, 1-H), 3.75 (dd, $^2J_{1',1}$ = 11.8 Hz, $^3J_{1',2}$ = 2.7 Hz, 1 H, 1-H'), 2.80 (dd, $^2J_{6,6'}$ = 18.8 Hz, $^3J_{6,5}$ = 6.2 Hz, 1 H, 6-H), 2.71 (dd, $^2J_{6',6}$ = 18.6 Hz, $^3J_{6',5}$ = 1.2 Hz, 1 H, 6-H'), 0.91 (s, 9 H, 10-H), 0.09 (s, 3 H, 8'-H), 0.08 (s, 3 H, 8-H).
^{13}C-NMR (125 MHz, $CDCl_3$): δ = 174.7 (s, C-7), 83.3 (d, C-4), 80.8 (d, C-2), 77.2 (d, C-5), 62.0 (t, C-1), 61.6 (d, C-3), 36.8 (t, C-6), 26.0 (q, C-10), 18.5 (s, C-9), −5.2 (q, C-8'), −5.4 (q, C-8).

**Optical rotation:**	$[\alpha]_D^{20}$= −156.3 (c = 1.0, $CHCl_3$)	
**HRMS (CI):**	calculated	found
$C_{13}H_{24}O_4N_3Si$ $[M+H]^+$:	314.1531	314.1511

2-((2*R*,3*S*,4*R*,5*S*)-4-Azido-5-(((*tert*-butyldimethylsilyl)oxy)methyl)-3-hydroxytetrahydrofuran-2-yl)acetaldehyde SI-4

In a 25 mL 2-neck flask, a solution of C-furanoside **12c** (90.0 mg, 287 µmol) in anhydrous CH_2Cl_2 (6.0 mL) was cooled to −78 °C. The solution was saturated with ozone until the reaction mixture turned blue (10 s). After stirring for another 30 s, the solution was degassed with N_2.

Dimethylsulfide (212 µL, 2.87 mmol, 10 eq.) was added at −70 °C and the reaction mixture was stirred for 18 h while slowly reaching room temperature. The reaction mixture was concentrated in vacuo, and the residue was purified by automated flash chromatography (silica, CyH:EtOAc 0% to 40% EtOAc) to give the aldehyde **SI-4** (57.0 mg, 181 µmol, 63%) as a colorless resin.

TLC: R_f (SI-4) = 0.35 (silica, pentane:EtOAc 6:4)

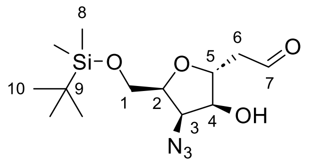

SI-4

^{1}H-NMR (500 MHz, $CDCl_3$): δ = 9.77 (dd, $^3J_{7,6}$ = 2.0 Hz, $^3J_{7,6'}$ = 1.5 Hz, 1 H, 7-H), 4.28 – 4.23 (m, 2 H, 3-H, 5-H), 4.20 (ddd, $^3J_{2,1}$ = 6.5 Hz, $^3J_{2,3}$ = 5.4 Hz, $^3J_{2,1'}$ = 4.1 Hz, 1 H, 2-H), 4.12 (ddd, $^3J_{4,OH}$ = 9.4 Hz, $^3J_{4,5}$ = 5.7 Hz, $^3J_{4,3}$ = 5.7 Hz, 1 H, 4-H), 3.81 (dd, $^2J_{1,1'}$ = 10.5 Hz, $^3J_{1,2}$ = 6.5 Hz, 1 H, 1-H), 3.75 (dd, $^2J_{1',1}$ = 10.5 Hz, $^3J_{1',2}$ = 4.1 Hz, 1 H, 1-H'), 3.56 (d, $^3J_{OH,4}$ = 9.7 Hz, 1 H, 4-OH), 2.74 (m, 1 H, 6-H'), 2.70 (m, 1 H, 6-H), 0.92 (s, 9 H, 10-H), 0.12 (s, 3 H, 8'-H), 0.11 (s, 3 H, 8-H).
^{13}C-NMR (125 MHz, $CDCl_3$): δ = 200.5 (d, C-7), 79.3 (d, C-2), 78.5 (d, C-5), 76.1 (d, C-4), 64.6 (d, C-3), 62.1 (t, C-1), 47.8 (t, C-6), 26.0 (q, C-10), 18.5 (s, C-9), −5.35 (q, C-8'), −5.42 (q, C-8).

**Optical rotation:**	$[\alpha]_D^{20}$= −29.5 (c = 1.0, $CHCl_3$)	
**HRMS (CI):**	calculated	found
$C_{13}H_{26}O_4NSi$ $[M-N_2+H]^+$:	288.1626	288.1622

(3a*S*,5*S*,6*S*,6a*S*)-6-Azido-5-(((*tert*-butyldimethylsilyl)oxy)methyl)tetrahydrofuro[3,2-b]furan-2(3*H*)-one SI-5

In a 25 mL 2-neck flask, a solution of C-furanoside **12d** (100 mg, 319 µmol) in anhydrous CH_2Cl_2 (6.0 mL) was cooled to −78 °C. The solution was saturated with ozone until the reaction mixture turned blue (10 s). After stirring for another 30 s, the solution was degassed with N_2. Dimethylsulfide (236 µL, 3.19 mmol, 10 eq.) was added at −70 °C and the reaction mixture was stirred for 18 h while slowly reaching room temperature. The reaction mixture was concentrated in vacuo, and the residue was purified by automated flash chromatography (silica, CyH:EtOAc 0% to 40% EtOAc) to give the hemiacetal (94.0 mg, 298 µmol, 93%) as a colorless resin.

The above-prepared hemiacetal (50.0 mg, 159 µmol) was dissolved in MeCN (1.2 mL). A solution of NaH_2PO_4 (3.8 mg, 31.7 µmol, 0.2 eq.) in H_2O (100 µL) and H_2O_2 (16.0 µL, 159 µmol, 30wt%, 1.0 eq.) were subsequently added. After cooling to 0 °C, a solution of $NaClO_2$ (28.7 mg, 254 µmol, 1.6 eq.) in H_2O (100 µL) was added dropwise. The resulting solution was stirred for 16 h at room temperature. After the addition of Na_2SO_4 (15 mg) and brine (2.0 mL), the mixture was extracted thrice with EtOAc. The combined organic layers were dried with $MgSO_4$ and concentrated in vacuo. The residue was dissolved in anhydrous CH_2Cl_2 (1.0 mL) before pTsOH (3.0 mg, 15.8 µmol, 10mol%) was added. The resulting solution was stirred for 2 h at room temperature. After

concentration in vacuo, the crude was purified by automated flash chromatography (silica, CyH:EtOAc 0% to 50% EtOAc) to give the lactone **SI-5** (30.0 mg, 96.1 µmol, 60%) as a colorless resin.

TLC: R_f (SI-5) = 0.31 (silica, pentane:EtOAc 6:4)

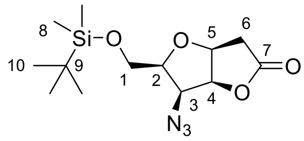

SI-5

^{1}H-NMR (500 MHz, $CDCl_3$): δ = 5.17 (dd, $^3J_{4,5}$ = 6.8 Hz, $^3J_{4,3}$ = 5.8 Hz, 1 H, 4-H), 4.70 (ddd, $^3J_{5,6}$ = 7.8 Hz, $^3J_{5,4}$ = 6.8 Hz, $^3J_{5,6'}$ = 3.4 Hz, 1 H, 5-H), 4.29 (dd, $^3J_{3,4}$ = 5.8 Hz, $^3J_{3,2}$ = 3.9 Hz, 1 H, 3-H), 3.95 (ddd, $^3J_{2,1}$ = 6.8 Hz, $^3J_{2,1'}$ = 5.8 Hz, $^3J_{2,3}$ = 3.9 Hz, 1 H, 2-H), 3.88 (dd, $^2J_{1,1'}$ = 10.3 Hz, $^3J_{1,2}$ = 6.8 Hz, 1 H, 1-H), 3.80 (dd, $^2J_{1',1}$ = 10.3 Hz, $^3J_{1',2}$ = 5.9 Hz, 1 H, 1-H'), 2.79 (dd, $^2J_{6,6'}$ = 18.8 Hz, $^3J_{6,5}$ = 7.8 Hz, 1 H, 6-H), 2.69 (dd, $^2J_{6',6}$ = 18.8 Hz, $^3J_{6',5}$ = 3.5 Hz, 1 H, 6-H'), 0.91 (s, 9 H, 10-H), 0.10 (s, 3 H, 8'-H), 0.09 (s, 3 H, 8-H).

^{13}C-NMR (125 MHz, $CDCl_3$): δ = 174.6 (s, C-7), 82.3 (d, C-4), 81.8 (d, C-2), 76.1 (d, C-5), 63.3 (t, C-1), 61.3 (d, C-3), 35.7 (t, C-6), 26.0 (q, C-10), 18.4 (s, C-9), –5.3 (q, C-8'), –5.4 (q, C-8).

Optical rotation: $[\alpha]_D^{20}$= –160.9 (c = 1.0, $CHCl_3$)

**HRMS (ESI):**	calculated	found
$C_{13}H_{24}N_3O_4Si$ $[M+H]^+$:	314.1531	314.1532

(((2*R*,3*S*,4*S*,5*R*)-5-Allyl-3-azido-4-((*tert*-butyldimethylsilyl)oxy)tetrahydrofuran-2-yl)methoxy)-(*tert*-butyl)dimethylsilane 13a

Imidazole (541 mg, 7.94 mmol, 3.0 eq.) and TBS-Cl (599 mg, 3.97 mmol, 1.5 eq.) were subsequently added to a 0 °C cold solution of C-furanoside **12a** (830 mg, 2.65 mmol) in anhydrous DMF (12 mL). The reaction mixture was stirred for 16 h while slowly reaching room temperature. After dilution with EtOAc, the mixture was washed with 5wt% $LiCl_{aq}$, 1.0 M HCl_{aq}, sat. $NaHCO_3$ solution and brine. The organic layer was dried over $MgSO_4$ and concentrated in vacuo. The crude was purified by column chromatography (silica, pentane:EtOAc 97:3) to give compound **13a** (1.03 g, 2.40 mmol, 91%) as a colorless oil.

TLC: R_f (13a) = 0.72 (silica, PE:EtOAc 9:1)

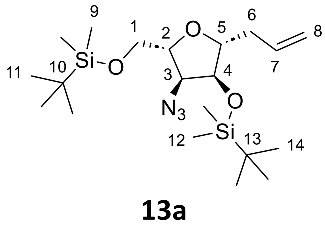

13a

^{1}H-NMR (400 MHz, $CDCl_3$): δ = 5.85 (ddt, $^3J_{7,8'}$ = 17.1 Hz, $^3J_{7,8}$ = 10.2 Hz, $^3J_{7,6}$ = 6.9 Hz, 1 H, 7-H), 5.11 – 5.06 (m, 2 H, 8-H), 4.02 – 3.96 (m, 2 H, 2-H, 4-H), 3.84 (ddd, $^3J_{5,6}$ = 7.2 Hz, $^3J_{5,4}$ = 5.7 Hz, $^3J_{5,6'}$ = 4.7 Hz, 1 H, 5-H), 3.75 (dd, $^3J_{1',1}$ = 11.3 Hz, $^3J_{1',2}$ = 3.3 Hz, 1 H, 1-H'), 3.70 (dd, $^2J_{1,1'}$ = 11.3 Hz, $^3J_{1,2}$ = 3.1 Hz, 1 H, 1-H), 3.66 (dd, $^3J_{3,4}$ = 5.3 Hz, $^3J_{3,2}$ = 5.3 Hz, 1 H, 3-H), 2.38 (dddt, $^2J_{6',6}$ = 14.2 Hz, $^3J_{6',7}$ = 6.3 Hz, $^3J_{6',5}$ = 4.7 Hz, $^4J_{6',8}$ = 1.5 Hz, 1 H, 6-H'), 2.20 (dddt, $^2J_{6,6'}$ = 14.4 Hz, $^3J_{6,7}$ = 7.4 Hz, $^3J_{6,5}$ = 7.4 Hz, $^4J_{6,8}$ = 1.4 Hz, 1 H, 6-H), 0.94 (s, 9 H, 14-H), 0.92 (s, 9 H, 11-H), 0.15 (s, 3 H, 12'-H), 0.12 (s, 3 H, 12-H), 0.09 (s, 3 H, 9'-H), 0.08 (s, 3 H, 9-H).

^{13}C-NMR (100 MHz, $CDCl_3$): δ = 134.4 (d, C-7), 117.5 (t, C-8), 82.7 (d, C-5), 81.6 (d, C-2), 76.6 (d, C-4), 63.3 (t, C-1), 62.9 (d, C-3), 37.6 (t, C-6), 26.1 (q, C-14), 25.9 (q, C-11), 18.5 (s, C-13), 18.2 (s, C-10), –4.4 (q, C-12'), –4.7 (q, C-12), –5.2 (q, C-9'), –5.4 (q, C-9).

**Optical rotation:**	$[\alpha]_D^{20}$= –7.0 (c = 1.0, $CHCl_3$)	
**HRMS (CI):**	calculated	found
$C_{20}H_{42}N_3O_3Si_2$ $[M+H]^+$:	428.2759	428.2758

(((2*S*,3*S*,4*S*,5*R*)-5-Allyl-3-azido-4-((*tert*-butyldimethylsilyl)oxy)tetrahydrofuran-2-yl)methoxy) (*tert*-butyl)dimethylsilane 13c

Imidazole (562 mg, 8.26 mmol, 2.8 eq.) and TBS-Cl (623 mg, 4.13 mmol, 1.4 eq.) were subsequently added to a 0 °C cold solution of C-furanoside **12c** (925 mg, 2.95 mmol) in anhydrous DMF (15 mL). The reaction mixture was stirred for 16 h while slowly reaching room temperature. Another portion of TBS-Cl (311 mg, 2.06 mmol, 0.7 eq.) was added, and the stirring continued for 4 h. After dilution with EtOAc, the mixture was washed with 5wt% $LiCl_{aq}$, 1.0 M HCl_{aq}, sat. $NaHCO_3$ solution and brine. The organic layer was dried over $MgSO_4$ and concentrated in vacuo. The crude was purified by column chromatography (silica, pentane:EtOAc 5% to 20% EtOAc) to give compound **13c** (887 mg, 2.07 mmol, 70%, 97%brsm) as a colorless oil.

TLC: R_f (13c) = 0.71 (silica, PE:EtOAc 8:2)

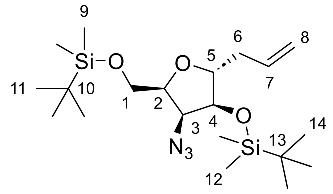

13c

^{1}H-NMR (500 MHz, $CDCl_3$): δ = 5.85 (dddd, $^3J_{7,8'}$ = 17.8 Hz, $^3J_{7,8}$ = 10.3 Hz, $^3J_{7,6}$ = 7.6 Hz, $^3J_{7,6'}$ = 6.1 Hz, 1 H, 7-H), 5.14 (m, 1 H, 8-H), 5.10 (m, 1 H, 8-H'), 4.13 (dd, $^3J_{4,5}$ = 8.1 Hz, $^3J_{4,3}$ = 4.7 Hz, 1 H, 4-H), 4.05 (ddd, $^3J_{2,1'}$ = 8.0 Hz, $^3J_{2,1}$ = 5.6 Hz, $^3J_{2,3}$ = 3.7 Hz, 1 H, 2-H), 3.90 – 3.85 (m, 2 H, 3-H, 5-H), 3.81 (dd, $^2J_{1',1}$ = 9.9 Hz, $^3J_{1',2}$ = 8.0 Hz, 1 H, 1-H'), 3.73 (dd, $^2J_{1,1'}$ = 9.8 Hz, $^3J_{1,2}$ = 5.6 Hz, 1 H, 1-H), 2.46 (m, 1 H, 6-H'), 2.19 (dddt, $^2J_{6,6'}$ = 14.5 Hz, $^3J_{6,7}$ = 7.8 Hz, $^3J_{6,5}$ = 6.8 Hz, $^4J_{6,8}$ = 1.2 Hz, 1 H, 6-H), 0.95 (s, 9 H, 14-H), 0.90 (s, 9 H, 11-H), 0.16 (s, 3 H, 12'-H), 0.13 (s, 3 H, 12-H), 0.09 (s, 3 H, 9'-H), 0.08 (s, 3 H, 9-H).

^{13}C-NMR (125 MHz, $CDCl_3$): δ = 134.2 (d, C-7), 117.8 (t, C-8), 80.3 (d, C-5), 79.0 (d, C-2), 77.2 (d, C-4), 65.5 (d, C-3), 62.1 (t, C-1), 37.1 (t, C-6), 26.0 (q, C-14), 25.8 (q, C-11), 18.5 (s, C-13), 18.1 (s, C-10), –4.3 (q, C-12'), –4.7 (q, C-12), –5.26 (q, C-9'), –5.32 (q, C-9).

**Optical rotation:**	$[\alpha]_D^{20}$= –12.3 (c = 1.0, $CHCl_3$)	
**HRMS (CI):**	calculated	found
$C_{20}H_{42}N_3O_3Si_2$ $[M+H]^+$:	428.2759	428.2767

((2*R*,3*S*,4*S*,5*R*)-Allyl-3-azido-4-((*tert*-butyldimethylsilyl)oxy)tetrahydrofuran-2-yl) methanol 14a

A solution of compound **13a** (1.02 g, 2.26 mmol) in anhydrous MeOH (16 mL) was cooled to 0 °C. After the addition of $PyBr_3$ (36.1 mg, 113 µmol, 5mol%), the reaction mixture was stirred for 8 h while slowly reaching room temperature. The solution was diluted with EtOAc and washed with 1.0 M HCl_{aq}, sat. $NaHCO_3$ solution, and brine. The organic layer was dried with $MgSO_4$ and concentrated in vacuo. The crude was purified by column chromatography (silica, PE:EtOAc 8:2) to give the primary alcohol **14a** (580 mg, 1.85 mmol, 82%) as a colorless oil.

TLC: R_f (14a) = 0.10 (silica, PE:EtOAc 9:1)

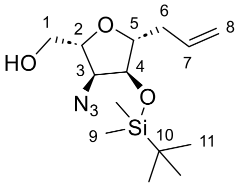

14a

^{1}H-NMR (500 MHz, $CDCl_3$): δ = 5.82 (ddt, $^3J_{7,8'}$ = 17.2 Hz, $^3J_{7,8}$ = 10.3 Hz, $^3J_{7,6}$ = 7.0 Hz, 1 H, 7-H), 5.17 – 5.11 (m, 2 H, 8-H), 4.05 – 3.99 (m, 2 H, 2-H, 4-H), 3.92 – 3.85 (m, 2 H, 1-H', 5-H), 3.64 (ddd, $^2J_{1,1'}$ = 11.9 Hz, $^3J_{1,OH}$ = 8.4 Hz, $^3J_{1,2}$ = 3.1 Hz, 1 H, 1-H), 3.58 (dd, $^3J_{3,2}$ = 6.8 Hz, $^3J_{3,4}$ = 5.7 Hz, 1 H, 3-H), 2.39 (dddt, $^2J_{6',6}$ = 14.6 Hz, $^3J_{6',7}$ = 6.6 Hz, $^3J_{6',5}$ = 5.0 Hz, $^4J_{6',8}$ = 1.4 Hz, 1 H, 6-H'), 2.24 (dddt, $^2J_{6,6'}$ = 14.3 Hz, $^3J_{6,7}$ = 7.0 Hz, $^3J_{6,5}$ = 7.0 Hz, $^4J_{6,8}$ = 1.3 Hz, 1 H, 6-H), 1.87 (m, 1 H, 1-OH), 0.95 (s, 9 H, 11-H), 0.16 (s, 3 H, 9'-H), 0.13 (s, 3 H, 9-H).

^{13}C-NMR (125 MHz, $CDCl_3$): δ = 133.7 (d, C-7), 118.3 (t, C-8), 83.9 (d, C-5), 80.8 (d, C-2), 76.5 (d, C-4), 62.1 (t, C-1), 61.6 (d, C-3), 37.6 (t, C-6), 25.9 (q, C-11), 18.2 (s, C-10), –4.5 (q, C-9'), –4.7 (q, C-9).

**Optical rotation:**	$[\alpha]_D^{20}$= –3.4 (c = 1.0, $CHCl_3$)	
**HRMS (CI):**	calculated	found
$C_{14}H_{30}NO_3Si$ $[M-N_2+3H]^+$:	288.1989	288.1995

((2*S*,3*S*,4*S*,5*R*)-Allyl-3-azido-4-((*tert*-butyldimethylsilyl)oxy)tetrahydrofuran-2-yl)methanol 14c

A solution of compound **13c** (750 mg, 1.75 mmol) in anhydrous MeOH (12 mL) was cooled to 0 °C. After the addition of $PyBr_3$ (33.0 mg, 88.0 µmol, 5mol%), the reaction mixture was stirred for 2 h at 0 °C and 3 h at room temperature. The solution was diluted with EtOAc and washed with 1.0 M HCl_{aq}, sat. $NaHCO_3$ solution and brine. The organic layer was dried with $MgSO_4$ and concentrated

in vacuo. The crude was purified by column chromatography (silica, PE:EtOAc 7:3) to give the primary alcohol **14c** (435 mg, 1.39 mmol, 79%) as a colorless oil.

TLC: R_f (14c) = 0.23 (silica, PE:EtOAc 7:3)

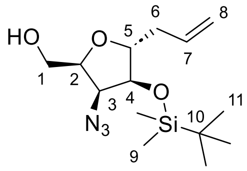

14c

^{1}H-NMR (500 MHz, $CDCl_3$): δ = 5.84 (dddd, $^3J_{7,8'}$ = 17.6 Hz, $^3J_{7,8}$ = 10.2 Hz, $^3J_{7,6}$ = 7.5 Hz, $^3J_{7,6'}$ = 6.3 Hz, 1 H, 7-H), 5.15 (m, 1 H, 8-H), 5.12 (m, 1 H, 8-H'), 4.16 – 4.11 (m, 2 H, 2-H, 4-H), 3.98 – 3.94 (m, 2 H, 3-H, 5-H), 3.81 (ddd, $^2J_{1,1'}$ = 11.7 Hz, $^3J_{1,2}$ = 6.1 Hz, $^3J_{1,OH}$ = 4.2 Hz, 1 H, 1-H), 3.75 (ddd, $^2J_{1',1}$ = 11.7 Hz, $^3J_{1',OH}$ = 8.1 Hz, $^3J_{1',2}$ = 5.2 Hz, 1 H, 1-H'), 2.45 (dddt, $^2J_{6',6}$ = 14.6 Hz, $^3J_{6',7}$ = 6.1 Hz, $^3J_{6',5}$ = 4.5 Hz, $^4J_{6',8}$ = 1.6 Hz, 1 H, 6-H'), 2.21 (dddt, $^2J_{6,6'}$ = 15.0 Hz, $^3J_{6,7}$ = 7.2 Hz, $^3J_{6,5}$ = 7.2 Hz, $^4J_{6,8}$ = 1.2 Hz, 1 H, 6-H), 2.04 (dd, $^3J_{OH,1'}$ = 8.1 Hz, $^3J_{OH,1}$ = 5.2 Hz, 1 H, 1-OH), 0.95 (s, 9 H, 11-H), 0.16 (s, 3 H, 9'-H), 0.13 (s, 3 H, 9-H).

^{13}C-NMR (125 MHz, $CDCl_3$): δ = 133.9 (d, C-7), 118.0 (t, C-8), 81.0 (d, C-5), 78.6 (d, C-2), 77.2 (d, C-4), 64.6 (d, C-3), 62.4 (t, C-1), 37.2 (t, C-6), 25.8 (q, C-11), 18.1 (s, C-10), –4.3 (q, C-9'), –4.7 (q, C-9).

**Optical rotation:**	$[\alpha]_D^{20}$= –14.2 (c = 1.0, $CHCl_3$)	
**HRMS (CI):**	calculated	found
$C_{14}H_{28}O_3N_3Si$ [M+H]$^+$:	314.1894	314.1900

Methyl (2*R*,3*S*,4*S*,5*R*)-5-allyl-3-azido-4-((*tert*-butyldimethylsilyl)oxy)tetrahydrofuran-2-carboxylate 15a

A solution of DMSO (380 µL, 5.36 mmol, 3.0 eq.) in anhydrous CH_2Cl_2 (1.2 mL) was added dropwise to a –78 °C cold solution of oxalyl dichloride (235 µL, 2.68 mmol, 1.5 eq.) in anhydrous CH_2Cl_2 (4.8 mL) keeping the temperature below –70 °C. After complete addition, the mixture was stirred at –60 to –70 °C for 30 min. A solution of primary alcohol **14a** (560 mg, 1.79 mmol) in anhydrous CH_2Cl_2 (4.0 mL) was added dropwise while keeping the temperature below –60 °C. After stirring for another 45 min, a solution of DIPEA (1.56 mL, 8.93 mmol, 5.0 eq.) in anhydrous CH_2Cl_2 (1.2 mL) was added dropwise while keeping the temperature around –60 °C. The stirring was continued for 30 min before warming to 0 °C. After the addition of 1.0 M HCl_{aq} (20 mL), the aqueous layer was extracted thrice with CH_2Cl_2. The combined organic layers were washed with phosphate buffer (pH 7), dried with $MgSO_4$, and concentrated in vacuo to give the crude aldehyde.

The above-prepared crude aldehyde was dissolved in MeCN (12 mL). A solution of NaH_2PO_4 (55.7 mg, 357 µmol, 0.2 eq.) in H_2O (1.2 mL) and H_2O_2 (182 µL, 1.79 mmol, 30wt%, 1.0 eq.) were subsequently added. After cooling to 0 °C, a solution of $NaClO_2$ (323 mg, 2.86 mmol, 1.6 eq.) in

H_2O (1.2 mL) was added dropwise. The resulting solution was stirred for 16 h while slowly reaching room temperature. After the addition of Na_2SO_4 (50 mg) and brine (2.0 mL), the mixture was extracted thrice with EtOAc. The combined organic layers were dried with $MgSO_4$ and concentrated in vacuo to give the carboxylic acid (589 mg, 1.69 mmol, 95%) as a colorless resin.

K_2CO_3 (367 mg, 2.65 mmol, 1.6 eq.) and MeI (332 µL, 5.30 mmol, 3.2 eq.) were added to a solution of the above prepared carboxylic acid (579 mg, 1.66 mmol, 94wt%) in anhydrous DMF (12 mL). The resulting suspension was stirred for 18 h at room temperature. After full conversion, the reaction mixture was diluted with EtOAc and washed with 5wt% $LiCl_{aq}$, 1.0 M HCl_{aq}, sat. $NaHCO_3$ solution and brine. The organic layer was dried over $MgSO_4$ and concentrated in vacuo. The crude was purified by column chromatography (silica, PE:EtOAc 9:1) to give compound **15a** (457 mg, 1.34 mmol, 81%) as a colorless oil.

TLC: R_f (15a) = 0.50 (silica, PE:EtOAc 8:2)

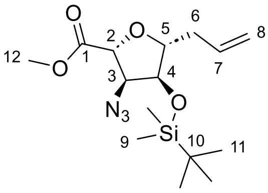

15a

^{1}H-NMR (500 MHz, $CDCl_3$): δ = 5.86 (ddt, $^3J_{7,8'}$ = 17.2 Hz, $^3J_{7,8}$ = 10.2 Hz, $^3J_{7,6}$ = 6.9 Hz, 1 H, 7-H), 5.18 – 5.11 (m, 2 H, 8-H), 4.45 (d, $^3J_{2,3}$ = 5.6 Hz, 1 H, 2-H), 4.08 (dd, $^3J_{4,3}$ = 5.2 Hz, $^3J_{4,5}$ = 5.2 Hz, 1 H, 4-H), 3.97 (ddd, $^3J_{5,6}$ = 6.8 Hz, $^3J_{5,4}$ = 5.2 Hz, $^3J_{5,6'}$ = 5.2 Hz, 1 H, 5-H), 3.81 (s, 3 H, 12-H), 3.80 (dd, $^3J_{3,2}$ = 5.5 Hz, $^3J_{3,4}$ = 5.5 Hz, 1 H, 3-H), 2.41 (dddt, $^2J_{6',6}$ = 14.8 Hz, $^3J_{6',7}$ = 6.8 Hz, $^3J_{6',5}$ = 5.3 Hz, $^4J_{6',8}$ = 1.4 Hz, 1 H, 6-H'), 2.34 (dddt, $^2J_{6,6'}$ = 14.3 Hz, $^3J_{6,7}$ = 7.0 Hz, $^3J_{6,5}$ = 7.0 Hz, $^4J_{6,8}$ = 1.3 Hz, 1 H, 6-H), 0.94 (s, 9 H, 11-H), 0.15 (s, 3 H, 9'-H), 0.12 (s, 3 H, 9-H).
^{13}C-NMR (125 MHz, $CDCl_3$): δ = 170.9 (s, C-1), 133.6 (d, C-7), 118.2 (t, C-8), 84.2 (d, C-5), 78.8 (d, C-2), 76.2 (d, C-4), 64.9 (d, C-3), 52.7 (q, C-12), 37.4 (t, C-6), 25.9 (q, C-11), 18.2 (s, C-10), –4.4 (q, C-9'), –4.7 (q, C-9).

**Optical rotation:**	$[\alpha]_D^{20}$ = –31.7 (c = 1.0, $CHCl_3$)	
**HRMS (CI):**	calculated	found
$C_{15}H_{28}NO_4Si$ $[M-N_2+H]^+$:	314.1782	314.1802

Methyl (2*S*,3*S*,4*S*,5*R*)-5-allyl-3-azido-4-((*tert*-butyldimethylsilyl)oxy)tetrahydrofuran-2-carboxylate 15c

A solution of DMSO (283 µL, 3.99 mmol, 3.0 eq.) in anhydrous CH_2Cl_2 (800 µL) was added dropwise to a –78 °C cold solution of oxalyl dichloride (174 µL, 2.00 mmol, 1.5 eq.) in anhydrous CH_2Cl_2 (3.0 mL) keeping the temperature below –70 °C. After complete addition, the mixture was stirred at –60 to –70 °C for 30 min. A solution of primary alcohol **14c** (417 mg, 1.33 mmol) in anhydrous CH_2Cl_2 (2.5 mL) was added dropwise while keeping the temperature below –60 °C. After stirring for another 45 min, a solution of DIPEA (1.16 mL, 6.65 mmol, 5.0 eq.) in anhydrous CH_2Cl_2 (800 µL)

was added dropwise while keeping the temperature around –60 °C. The stirring was continued for 30 min before warming to 0 °C. After the addition of 1.0 M HCl_{aq} (12 mL), the aqueous layer was extracted thrice with CH_2Cl_2. The combined organic layers were washed with phosphate buffer (pH 7), dried with $MgSO_4$, and concentrated in vacuo to give the crude aldehyde.

The above-prepared crude aldehyde was dissolved in MeCN (7.5 mL). A solution of NaH_2PO_4 (41.5 mg, 266 µmol, 0.2 eq.) in H_2O (750 µL) and H_2O_2 (136 µL, 1.33 mmol, 30wt%, 1.0 eq.) were subsequently added. After cooling to 0 °C, a solution of $NaClO_2$ (240 mg, 2.13 mmol, 80wt% 1.6 eq.) in H_2O (750 µL) was added dropwise. The resulting solution was stirred for 16 h while slowly reaching room temperature. After the addition of Na_2SO_4 (176 mg) and brine (8.0 mL), the mixture was extracted thrice with EtOAc. The combined organic layers were dried with $MgSO_4$ and concentrated in vacuo to give the carboxylic acid (439 mg, 1.23 mmol, 93wt%, 93%) as a colorless resin.

K_2CO_3 (241 mg, 1.75 mmol, 1.5 eq.) and MeI (218 µL, 3.49 mmol, 3.0 eq.) were added to a solution of the above-prepared carboxylic acid (410 mg, 1.16 mmol, 93wt%) in anhydrous DMF (9.0 mL). The resulting suspension was stirred for 18 h at room temperature. After full conversion, the reaction mixture was diluted with EtOAc and washed with 5wt% $LiCl_{aq}$, 1.0 M HCl_{aq}, sat. $NaHCO_3$ solution and brine. The organic layer was dried over $MgSO_4$ and concentrated in vacuo. The crude was purified by column chromatography (silica, PE:EtOAc 9:1) to give compound **15c** (291 mg, 852 µmol, 73%) as a colorless oil.

TLC: R_f (15c) = 0.25 (silica, PE:EtOAc 9:1)

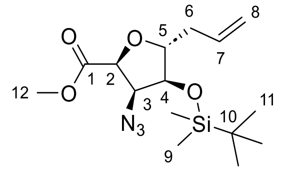

15c

^{1}H-NMR (500 MHz, $CDCl_3$): δ = 5.86 (dddd, $^3J_{7,8'}$ = 17.7 Hz, $^3J_{7,8}$ = 10.2 Hz, $^3J_{7,6}$ = 7.7 Hz, $^3J_{7,6}$ = 5.9 Hz, 1 H, 7-H), 5.18 (ddt, $^3J_{8',7}$ = 17.2 Hz, $^2J_{8',8}$ = 1.5 Hz, $^4J_{8',6}$ = 1.5 Hz, 1 H, 8-H'), 5.14 (m, 1 H, 8-H), 4.62 (d, $^3J_{2,3}$ = 4.6 Hz, 1 H, 2-H), 4.15 – 4.10 (m, 3 H, 3-H, 4-H, 5-H), 3.82 (s, 3 H, 12-H), 2.57 (m, 1 H, 6-H), 2.26 (m, 1 H, 6-H'), 0.94 (s, 9 H, 11-H), 0.15 (s, 3 H, 9'-H), 0.14 (s, 3 H, 9-H).

^{13}C-NMR (125 MHz, $CDCl_3$): δ = 169.5 (s, C-1), 133.2 (d, C-7), 118.4 (t, C-8), 81.1 (d, C-5), 77.9 (d, C-2), 76.2 (d, C-4), 65.2 (d, C-3), 52.5 (q, C-12), 36.1 (t, C-6), 25.8 (q, C-11), 18.1 (s, C-10), –4.2 (q, C-9'), –4.8 (q, C-9).

**Optical rotation:**	$[\alpha]_D^{20}$= +17.5 (c = 0.5, $CHCl_3$)	
**HRMS (CI):**	calculated	found
$C_{15}H_{28}O_4N_3Si$ $[M+H]^+$:	342.1844	342.1823

Methyl (2*R*,3*S*,4*S*,5*R*)-5-allyl-3-amino-4-((*tert*-butyldimethylsilyl)oxy)tetrahydrofuran-2-carboxylate 16a

Triphenylphosphine (401 mg, 1.53 mmol, 1.2 eq.) was added to a solution of compound **15a** (435 mg, 1.27 mmol) in THF:H_2O (10.4 mL, 25:1). The reaction mixture was stirred for 16 h at room temperature before another portion of triphenylphosphine (16.7 mg, 64.0 µmol, 0.05 eq.) was added. After another 2 h at room temperature, the reaction mixture was acidified with 0.1 M HCl_{aq}. The mixture was washed twice with Et_2O (discard) before sat. $NaHCO_3$ solution was added. The aqueous layer was extracted four times with Et_2O. The combined organic layers were dried with $MgSO_4$ and concentrated in vacuo to give amine **16a** (360 mg, 1.14 mmol, 90%) as a colorless resin.

TLC: R_f (16a) = 0.22 (silica, PE:EtOAc 7:3)

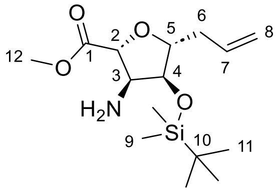

16a

^{1}H-NMR (400 MHz, $CDCl_3$): δ = 5.85 (ddt, $^3J_{7,8'}$ = 17.2 Hz, $^3J_{7,8}$ = 10.2 Hz, $^3J_{7,6}$ = 7.1 Hz, 1 H, 7-H), 5.18 – 5.11 (m, 2 H, 8-H), 4.14 (d, $^3J_{2,3}$ = 8.3 Hz, 1 H, 2-H), 4.01 – 3.93 (m, 2 H, 4-H, 5-H), 3.80 (s, 3 H, 12-H), 3.36 (dd, $^3J_{3,2}$ = 8.2 Hz, $^3J_{3,4}$ = 4.9 Hz, 1 H, 3-H), 2.38 (m, 1 H, 6-H‘), 2.30 (dddt, $^2J_{6,6'}$ = 14.1 Hz, $^3J_{6,7}$ = 6.8 Hz, $^3J_{6,5}$ = 6.8 Hz, $^4J_{6,8}$ = 1.2 Hz, 1 H, 6-H), 0.93 (s, 9 H, 11-H), 0.11 (s, 3 H, 9‘-H), 0.10 (s, 3 H, 9-H).

^{13}C-NMR (125 MHz, $CDCl_3$): δ = 172.5 (s, C-1), 134.1 (d, C-7), 118.0 (t, C-8), 85.9 (d, C-5), 82.3 (d, C-2), 76.4 (d, C-4), 58.6 (d, C-3), 52.3 (q, C-12), 38.5 (t, C-6), 26.0 (q, C-11), 18.2 (s, C-10), –4.3 (q, C-9‘), –4.5 (q, C-9).

Methyl (2*S*,3*S*,4*S*,5*R*)-5-allyl-3-amino-4-((*tert*-butyldimethylsilyl)oxy)tetrahydrofuran-2-carboxylate 16c

Triphenylphosphine (249 mg, 949 µmol, 1.2 eq.) was added to a solution of compound **15c** (270 mg, 791 µmol) in THF:H_2O (6.2 mL, 25:1). The reaction mixture was stirred for 16 h at room temperature before another portion of triphenylphosphine (10.4 mg, 40.0 µmol, 0.05 eq.) was added. After another 2 h at room temperature, the reaction mixture was acidified with 0.1 M HCl_{aq}. The mixture was washed twice with Et_2O (discard) before sat. $NaHCO_3$ solution was added. The aqueous layer was extracted four times with Et_2O. The combined organic layers were dried with $MgSO_4$ and concentrated in vacuo to give amine **16c** (233 mg, 739 µmol, 93%) as a colorless resin.

TLC: R_f (16c) = 0.16 (silica, PE:EtOAc 6:4)

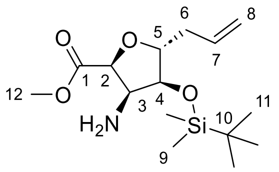

16c

^{1}H-NMR (400 MHz, $CDCl_3$): δ = 5.86 (ddt, $^3J_{7,8'}$ = 17.2 Hz, $^3J_{7,8}$ = 10.2 Hz, $^3J_{7,6}$ = 7.0 Hz, 1 H, 7-H), 5.19 – 5.09 (m, 2 H, 8-H), 4.52 (d, $^3J_{2,3}$ = 6.1 Hz, 1 H, 2-H), 4.13 (dt, $^3J_{5,4}$ = 5.6 Hz, $^3J_{5,6}$ = 5.6 Hz, 1 H, 5-H), 3.93 (dd, $^3J_{4,5}$ = 5.3 Hz, $^3J_{4,3}$ = 5.3 Hz, 1 H, 4-H), 3.80 (s, 3 H, 12-H), 3.63 (dd, $^3J_{3,2}$ = 6.1 Hz, $^3J_{3,4}$ = 5.1 Hz, 1 H, 3-H), 2.44 (dddt, $^2J_{6',6}$ = 14.8 Hz, $^3J_{6',7}$ = 6.8 Hz, $^3J_{6',5}$ = 5.5 Hz, $^4J_{6',8}$ = 1.4 Hz, 1 H, 6-H‘), 2.29 (dddt, $^2J_{6,6'}$ = 14.6 Hz, $^3J_{6,7}$ = 7.2 Hz, $^3J_{6,5}$ = 5.8 Hz, $^4J_{6,8}$ = 1.2 Hz, 1 H, 6-H), 0.91 (s, 9 H, 11-H), 0.10 (s, 6 H, 9-H).

Methyl (2*R*,3*S*,4*S*,5*R*)-5-allyl-3-((*R*)-4-((*tert*-butoxycarbonyl)amino)-3-((*tert*-butyldimethylsilyl)-oxy)butanamido)-4-((*tert*-butyldimethylsilyl)oxy)tetrahydrofuran-2-carboxylate 17a

Diazoketone **8** (150 mg, 415 µmol, 1.5 eq.) and amine **16a** (87.0 mg, 277 µmol) were dissolved in anhydrous CH_2Cl_2 (2.8 mL). At room temperature, the resulting yellow solution was irradiated with a blue LED (365 nm, 18 W) for 1 h. The colorless solution was concentrated in vacuo, and the residue was purified by column chromatography (silica, pentane:EtOAc 8:2) to give compound **17a** (120 mg, 190 µmol, 69%) as a colorless resin.

TLC: R_f (17a) = 0.44 (SiO_2, PE:EtOAc 7:3)

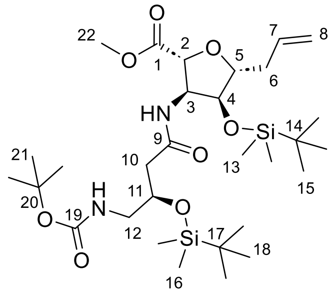

17a

^{1}H-NMR (400 MHz, $CDCl_3$): δ = 6.46 (d, $^3J_{NH,3}$ = 8.0 Hz, 1 H, 9-NH), 5.87 (ddt, $^3J_{7,8'}$ = 17.2 Hz, $^3J_{7,8}$ = 10.2 Hz, $^3J_{7,6}$ = 7.1 Hz, 1 H, 7-H), 5.20 – 5.11 (m, 2 H, 8-H), 4.88 (m, 1 H, 19-NH), 4.51 (ddd, $^3J_{3,2}$ = 8.0 Hz, $^3J_{3,NH}$ = 8.0 Hz, $^3J_{3,4}$ = 5.5 Hz, 1 H, 3-H), 4.27 (d, $^3J_{2,3}$ = 7.9 Hz, 1 H, 2-H), 4.19 (tt, $^3J_{11,10}$ = 5.4 Hz, $^3J_{11,12}$ = 5.4 Hz, 1 H, 11-H), 4.06 (dd, $^3J_{4,3}$ = 5.6 Hz, $^3J_{4,5}$ = 2.8 Hz, 1 H, 4-H), 3.99 (td, $^3J_{5,6}$ = 6.3 Hz, $^3J_{5,4}$ = 2.9 Hz, 1 H, 5-H), 3.75 (s, 3 H, 22-H), 3.37 (m, 1 H, 12-H‘), 3.07 (ddd, $^2J_{12,12'}$ = 14.0 Hz, $^3J_{12,11}$ = 5.4 Hz, $^3J_{12,NH}$ = 5.4 Hz, 1 H, 12-H), 2.46 – 2.29 (m, 4 H, 6-H, 10-H), 1.45 (s, 9 H, 21-H), 0.92 (s, 9 H, 15-H), 0.90 (s, 9 H, 18-H), 0.12 (s, 3 H, 13‘-H), 0.09 (s, 6 H, 16-H), 0.09 (s, 3 H, 13-H).

^{13}C-NMR (100 MHz, $CDCl_3$): δ = 171.5 (s, C-1), 170.0 (s, C-9), 156.4 (s, C-19), 133.8 (d, C-7), 118.2 (t, C-8), 86.0 (d, C-5), 79.9 (d, C-2), 79.5 (s, C-20), 75.0 (d, C-4), 68.8 (d, C-11), 55.0 (d, C-3), 52.5 (q, C-22), 45.3 (t, C-12), 42.5 (t, C-10), 38.1 (t, C-6), 28.6 (q, C-21), 26.0 (q, C-15), 25.9 (q, C-18), 18.2 (s, C-14), 18.1 (s, C-17), –4.4 (q, C-13‘), –4.5 (q, C-16‘), –4.6 (q, C-13), –4.7 (q, C-16).

Optical rotation: $[\alpha]_D^{20}$= –7.4 (c = 1.0, $CHCl_3$)

**HRMS (CI):**	calculated	found
$C_{30}H_{59}N_2O_8Si_2$ [M+H]$^+$:	631.3804	631.3802

Methyl (2*R*,3*R*,4*S*,5*S*)-5-allyl-3-((*R*)-4-((*tert*-butoxycarbonyl)amino)-3-((*tert*-butyldimethylsilyl)-oxy)butanamido)-4-hydroxytetrahydrofuran-2-carboxylate 17b

In a 10 mL Schlenk tube under an atmosphere of nitrogen, DMP (138 mg, 315 µmol, 1.15 eq.) was added to a 0 °C cold solution of compound **20b** (134 mg, 274 µmol) in CH_2Cl_2 (2.7 mL). The resulting solution was stirred for 100 min before another portion of DMP (18.0 mg, 41.0 µmol, 0.15 eq.) was added. After stirring for another 20 min, the reaction mixture was quenched by adding a preformed mixture of sat. $NaHCO_3$ solution and sat. NaS_2O_3 solution (4 mL, 7:3). The aqueous layer was extracted twice with CH_2Cl_2. The combined organic layers were dried with $MgSO_4$ and concentrated in vacuo to give the crude aldehyde.

The above-prepared crude aldehyde was dissolved in MeCN (2.7 mL). A solution of NaH_2PO_4 (7.5 mg, 55.0 µmol, 0.2 eq.) in H_2O (100 µL) and H_2O_2 (27.9 µL, 273 µmol, 30wt%, 1.0 eq.) were subsequently added. After cooling to 0 °C, a solution of $NaClO_2$ (49.4 mg, 437 µmol, 1.6 eq.) in H_2O (100 µL) was added dropwise. The resulting solution was stirred for 2 h at room temperature. After the addition of Na_2SO_4 (80 mg) and brine (7.0 mL), the mixture was extracted thrice with EtOAc. The combined organic layers were dried with $MgSO_4$ and concentrated in vacuo to give the crude carboxylic acid.

K_2CO_3 (56.5 mg, 409 µmol, 1.5 eq.) and MeI (85.0 µL, 1.36 mmol, 5.0 eq.) were added to a solution of the above-prepared carboxylic acid in anhydrous DMF (2.7 mL). The resulting suspension was stirred for 1 h at room temperature. After full conversion, the reaction mixture was diluted with EtOAc and washed with 5wt% $LiCl_{aq}$, 1.0 M HCl_{aq}, sat. $NaHCO_3$ solution and brine. The organic layer was dried over $MgSO_4$ and concentrated in vacuo. The crude was purified by automated reversed phase column chromatography (C18 spherical, H_2O:MeCN 10% to 90% MeCN) to give compound **17b** (70.0 mg, 135 µmol, 50%) as a colorless resin.

LC-MS: t_R (17b) = 1.34 min (short method)

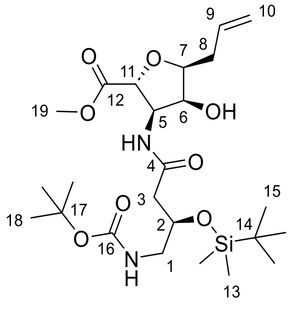

17b

^{1}H-NMR (500 MHz, $CDCl_3$): δ = 7.00 (d, $^3J_{NH,5}$ = 8.3 Hz, 1 H, 4-NH), 5.84 (ddt, $^3J_{9,10'}$ = 17.1 Hz, $^3J_{9,10}$ = 10.2 Hz, $^3J_{9,8}$ = 6.9 Hz, 1 H, 9-H), 5.21 (ddt, $^3J_{10',9}$ = 17.2 Hz, $^2J_{10',10}$ = 1.6 Hz, $^4J_{10',8}$ = 1.6 Hz, 1 H, 10-H‘), 5.11 (ddt, $^3J_{10,9}$ = 10.1 Hz, $^2J_{10,10'}$ = 1.2 Hz, $^4J_{10,8}$ = 1.2 Hz, 1 H, 10-H), 4.92 (t, $^3J_{NH,1}$ = 6.5 Hz,

1 H, 16-NH), 4.68 (ddd, $^3J_{5,NH}$ = 8.5 Hz, $^3J_{5,11}$ = 8.5 Hz, $^3J_{5,6}$ = 4.4 Hz, 1 H, 5-H), 4.33 (d, $^3J_{11,5}$ = 8.7 Hz, 1 H, 11-H), 4.27 (ddd, $^3J_{7,8}$ = 7.8 Hz, $^3J_{7,8'}$ = 6.5 Hz, $^3J_{7,6}$ = 2.7 Hz, 1 H, 7-H), 4.21 (m, 1 H, 6-H), 4.12 (tt, $^3J_{2,1}$ = 5.4 Hz, $^3J_{2,3}$ = 5.4 Hz, 1 H, 2-H), 3.76 (s, 3 H, 19-H), 3.22 (m, 2 H, 1-H), 2.87 (m, 1 H, 6-OH), 2.53 (m, 2 H, 8-H), 2.43 (m, 2 H, 3-H), 1.45 (s, 9 H, 18-H), 0.90 (s, 9 H, 15-H), 0.12 (s, 3 H, 13-H), 0.10 (s, 3 H, 13-H').

^{13}C-NMR (125 MHz, $CDCl_3$): δ = 172.5 (s, C-12), 170.4 (s, C-4), 156.7 (s, C-16), 133.7 (d, C-9), 118.0 (t, C-10), 82.3 (d, C-7), 80.1 (s, C-17), 78.5 (d, C-11), 72.0 (d, C-6), 69.1 (d, C-2), 57.1 (d, C-5), 52.6 (q, C-19), 45.3 (t, C-1), 42.3 (t, C-3), 33.6 (t, C-8), 28.5 (q, C-18), 25.9 (q, C-15), 18.1 (s, C-14), –4.69 (q, C-13), –4.70 (q, C-13').

Optical rotation: $[\alpha]_D^{20}$ = –14.4 (c = 1.0, $CHCl_3$)

**HRMS (ESI):**	calculated	found
$C_{24}H_{45}N_2O_8Si$ [M+H]$^+$:	517.2940	517.2946

Methyl (2*S*,3*S*,4*S*,5*R*)-5-allyl-3-((*R*)-4-((*tert*-butoxycarbonyl)amino)-3-((*tert*-butyldimethylsilyl)-oxy)butanamido)-4-((*tert*-butyldimethylsilyl)oxy)tetrahydrofuran-2-carboxylate 17c

Diazoketone **8** (361 mg, 998 µmol, 1.5 eq.) and amine **16c** (210 mg, 666 µmol) were dissolved in anhydrous CH_2Cl_2 (6.7 mL). The resulting yellow solution was irradiated with a blue LED (365 nm, 18 W) for 2 h at room temperature. The colorless solution was concentrated in vacuo, and the residue was purified by automated reversed phase column chromatography (C18 spherical, H_2O:MeCN 10% to 90% MeCN) to give compound **17c** (294 mg, 466 µmol, 70%) as a colorless resin.

TLC: R$_f$ (17c) = 0.51 (silica, PE:EtOAc 7:3)

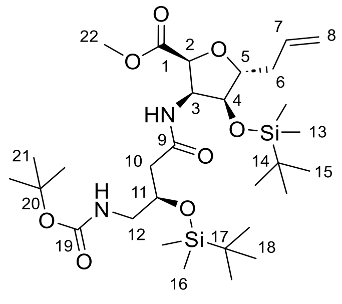

17c

^{1}H-NMR (500 MHz, $CDCl_3$): δ = 6.58 (d, $^3J_{NH,3}$ = 7.6 Hz, 1 H, 9-NH), 5.81 (ddt, $^3J_{7,8'}$ = 17.3 Hz, $^3J_{7,8}$ = 10.2 Hz, $^3J_{7,6}$ = 7.1 Hz, 1 H, 7-H), 5.20 – 5.11 (m, 2 H, 8-H), 4.90 (t, $^3J_{NH,12}$ = 5.8 Hz, 1 H, 19-NH), 4.63 – 4.56 (m, 2 H, 2-H, 3-H), 4.24 (tt, $^3J_{11,10}$ = 6.0 Hz, $^3J_{11,12}$ = 5.4 Hz, 1 H, 11-H), 4.18 (td, $^3J_{5,6}$ = 6.3 Hz, $^3J_{5,4}$ = 2.2 Hz, 1 H, 5-H), 4.02 (dd, $^3J_{4,3}$ = 4.8 Hz, $^3J_{4,5}$ = 2.3 Hz, 1 H, 4-H), 3.73 (s, 3 H, 22-H), 3.32 (m, 1 H, 12-H'), 3.17 (ddd, $^2J_{12,12'}$ = 13.7 Hz, $^3J_{12,11}$ = 5.3 Hz, $^3J_{12,NH}$ = 5.8 Hz, 1 H, 12-H), 2.41 (dd, $^2J_{10,12'}$ = 14.4 Hz, $^3J_{10,11}$ = 6.1 Hz, 1 H, 10-H), 2.37 – 2.29 (m, 3 H, 6-H, 10-H'), 1.44 (s, 9 H, 21-H), 0.89 (s, 9 H, 15-H), 0.87 (s, 9 H, 18-H), 0.11 (s, 3 H, 16'-H), 0.09 (s, 3 H, 16-H), 0.05 (s, 3 H, 13'-H), 0.04 (s, 3 H, 13-H).

^{13}C-NMR (100 MHz, $CDCl_3$): δ = 171.8 (s, C-1), 170.1 (s, C-9), 156.2 (s, C-19), 133.4 (d, C-7), 118.6 (t, C-8), 85.8 (d, C-5), 79.4 (s, C-20), 75.7 (d, C-2), 74.1 (d, C-4), 68.9 (d, C-11), 53.7 (d, C-3), 52.2 (q,

C-22), 45.8 (t, C-12), 42.5 (t, C-10), 38.3 (t, C-6), 28.6 (q, C-21), 26.0 (q, C-18), 25.7 (q, C-15), 18.2 (s, C-17), 18.0 (s, C-14), –4.4 (q, C-16), –4.68 (q, C-16′, C-13), –4.73 (q, C-13′).

Optical rotation: $[\alpha]_D^{20}$= +24.5 (c = 1.0, $CHCl_3$)

**HRMS (CI):**	calculated	found
$C_{30}H_{59}N_2O_8Si_2$ $[M+H]^+$:	631.3804	631.3801

(2*S*,3*S*,4*R*,5*R*)-2-Allyl-4-amino-5-(((*tert*-butyldimethylsilyl)oxy)methyl)tetrahydrofuran-3-ol 18b

Ammonium sulfide in H_2O (11.2 mL, 40wt%) was added to a 0 °C cold solution of C-furanoside **12b** (700 mg, 2.23 mmol) in MeCN (11.2 mL). The biphasic mixture was stirred for 16 h while slowly reaching room temperature. After the addition of sat. $NaHCO_3$ solution (30.0 mL), the mixture was extracted thrice with EtOAc. The combined organic layers were dried with $MgSO_4$ and concentrated in vacuo to give the crude amine **18b** (642 mg, 2.21 mmol, 99%) as a colorless resin.

TLC: R_f (18b) = 0.10 (silica, pentane:EtOAc 2:8)

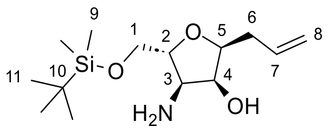

18b

^{1}H-NMR (500 MHz, $CDCl_3$): δ = 5.85 (ddt, $^3J_{7,8'}$ = 17.1 Hz, $^3J_{7,8}$ = 10.2 Hz, $^3J_{7,6}$ = 6.9 Hz, 1 H, 7-H), 5.15 (ddt, $^3J_{8',7}$ = 17.2 Hz, $^2J_{8',8}$ = 1.7 Hz, $^4J_{8',6}$ = 1.7 Hz, 1 H, 8-H′), 5.06 (ddt, $^3J_{8,7}$ = 10.2 Hz, $^2J_{8,8'}$ = 2.0 Hz, $^4J_{8,6}$ = 1.2 Hz, 1 H, 8-H), 3.97 (td, $^3J_{5,6}$ = 7.0 Hz, $^3J_{5,4}$ = 2.8 Hz, 1 H, 5-H), 3.94 (dd, $^3J_{4,3}$ = 4.4 Hz, $^3J_{4,5}$ = 2.9 Hz, 1 H, 4-H), 3.82 (dd, $^2J_{1,1'}$ = 9.8 Hz, $^3J_{1,2}$ = 3.5 Hz, 1 H, 1-H), 3.69 (ddd, $^3J_{2,3}$ = 7.7 Hz, $^3J_{2,1'}$ = 6.4 Hz, $^3J_{2,1}$ = 3.6 Hz, 1 H, 2-H), 3.64 (dd, $^2J_{1',1}$ = 9.8 Hz, $^3J_{1',2}$ = 6.4 Hz, 1 H, 1-H′), 3.47 (dd, $^3J_{3,2}$ = 7.9 Hz, $^3J_{3,4}$ = 4.7 Hz, 1 H, 3-H), 2.46 (m, 2 H, 6-H), 0.89 (s, 9 H, 11-H), 0.06 (s, 6 H, 9-H).

***tert*-Butyl ((*R*)-4-(((2*R*,3*R*,4*S*,5*S*)-5-allyl-2-(((*tert*-butyldimethylsilyl)oxy)methyl)-4-hydroxy-tetrahydrofuran-3-yl)amino)-2-((*tert*-butyldimethylsilyl)oxy)-4-oxobutyl)carbamate 19b**

Diazoketone **77** (49.3 mg, 143 µmol, 1.5 eq.) and amine **18b** (27.5 mg, 96.0 µmol) were dissolved in anhydrous CH_2Cl_2 (1.0 mL). At room temperature, the resulting yellow solution was irradiated with a blue LED (405 nm, 18 W) for 1.5 h. The colorless solution was concentrated in vacuo, and the residue was purified by automated reversed phase column chromatography (C18 spherical, H_2O:MeCN 10% to 90% MeCN) to give compound **19b** (38.0 mg, 63.0 µmol, 66%) as a colorless resin.

LC-MS: t_R (19b) = 1.55 min (short method)

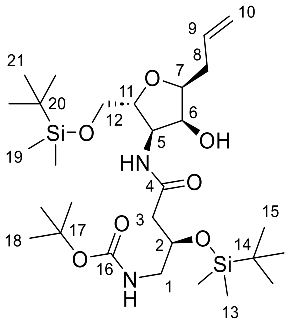

19b

^{1}H-NMR (500 MHz, $CDCl_3$): δ = 6.62 (d, $^3J_{NH,5}$ = 7.9 Hz, 1 H, 4-NH), 5.83 (ddt, $^3J_{9,10'}$ = 17.1 Hz, $^3J_{9,10}$ = 10.2 Hz, $^3J_{9,8}$ = 6.9 Hz, 1 H, 9-H), 5.17 (ddt, $^3J_{10',9}$ = 17.2 Hz, $^2J_{10',10}$ = 1.6 Hz, $^4J_{10',8}$ = 1.6 Hz, 1 H, 10-H'), 5.07 (m, 1 H, 10-H), 4.89 (t, $^3J_{NH,1}$ = 6.2 Hz, 1 H, 16-NH), 4.50 (ddd, $^3J_{5,NH}$ = 8.0 Hz, $^3J_{5,11}$ = 8.0 Hz, $^3J_{5,6}$ = 4.5 Hz, 1 H, 5-H), 4.17 – 4.10 (m, 2 H, 2-H, 6-H), 4.05 (td, $^3J_{7,8}$ = 7.2 Hz, $^3J_{7,6}$ = 2.6 Hz, 1 H, 7-H), 3.88 (dt, $^3J_{11,5}$ = 4.8 Hz, $^3J_{11,12}$ = 3.5 Hz, 1 H, 11-H), 3.82 (dd, $^2J_{12,12'}$ = 11.1 Hz, $^3J_{12,11}$ = 3.0 Hz, 1 H, 12-H), 3.74 (dd, $^2J_{12',12}$ = 11.0 Hz, $^3J_{12',11}$ = 4.0 Hz, 1 H, 12-H'), 3.17 (dd, $^3J_{1,2}$ = 5.8 Hz, $^3J_{1,NH}$ = 5.8 Hz, 2 H, 1-H), 2.81 (d, $^3J_{OH,6}$ = 5.8 Hz, 1 H, 6-OH), 2.45 (dd, $^3J_{8,7}$ = 6.7 Hz, $^3J_{8,9}$ = 6.7 Hz, 2 H, 8-H), 2.38 (d, $^3J_{3,2}$ = 6.1 Hz, 2 H, 3-H), 1.43 (s, 9 H, 18-H), 0.89 (s, 9 H, 15-H/21-H), 0.88 (s, 9 H, 21-H/15-H), 0.10 (s, 3 H, 13-H/19-H), 0.08 (s, 3 H, 13-H/19-H), 0.07 (s, 3 H, 13-H/19-H), 0.06 (s, 3 H, 13-H/19-H).

^{13}C-NMR (125 MHz, $CDCl_3$): δ = 170.3 (s, C-4), 156.5 (s, C-16), 134.4 (d, C-9), 117.4 (t, C-10), 81.7 (d, C-11), 81.4 (d, C-7), 80.0 (s, C-17), 72.6 (d, C-6), 69.1 (d, C-2), 64.5 (t, C-12), 54.2 (d, C-5), 45.6 (t, C-1), 42.5 (t, C-3), 33.9 (t, C-8), 28.5 (q, C-18), 26.1 (q, C-15/C-21), 25.9 (q, C-21/C-15), 18.5 (s, C-14/C-20), 18.1 (s, C-20/C-14), –4.69 (q, C-13/C-19), –4.72 (q, C-13/C-19), –5.20 (q, C-13/C-19), –5.26 (q, C-13/C-19).

**Optical rotation:**	$[\alpha]_D^{20}$= –14.7 (c = 1.0, $CHCl_3$)	
**HRMS (CI):**	calculated	found
$C_{29}H_{59}N_2O_7Si_2$ $[M+H]^+$:	603.3855	603.3856

***tert*-Butyl ((*R*)-4-(((2*R*,3*R*,4*S*,5*S*)-5-allyl-4-hydroxy-2-(hydroxymethyl)tetrahydrofuran-3-yl)amino)-2-((*tert*-butyldimethylsilyl)oxy)-4-oxobutyl)carbamate 20b**

1.0 M TBAF in THF (621 µL, 621 µmol, 1.05 eq.) was added to a 0 °C cold solution of the crude amine **18b** (170 mg, 591 µmol) in anhydrous THF (5.9 mL). The resulting solution was stirred for 2 h while slowly reaching room temperature. After adding 1.0 M HCl_{aq} (621 µL, 621 µmol, 1.05 eq.), the reaction mixture was concentrated in vacuo. The residue was dissolved in anhydrous CH_2Cl_2 (5.9 mL) before diazoketone **8** (323 mg, 883 µmol, 1.5 eq.) was added. The resulting yellow solution was irradiated with a blue LED (405 nm, 18 W) at room temperature for 30 min. The reaction mixture was concentrated in vacuo, and the residue was purified by automated reversed phase column chromatography (C18 spherical, H_2O:MeCN 10% to 90% MeCN) to give compound **20b** (151 mg, 309 µmol, 52%) as a colorless resin.

LC-MS: t_R (20b) = 1.23 min (short method)

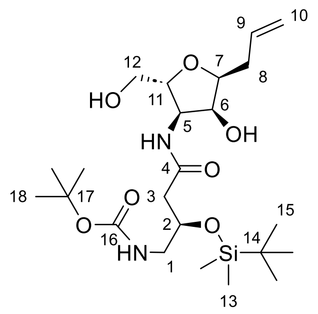

20b

^{1}H-NMR (500 MHz, $CDCl_3$): δ = 6.99 (d, $^3J_{NH,5}$ = 8.0 Hz , 1 H, 4-NH), 5.84 (ddt, $^3J_{9,10'}$ = 17.0 Hz, $^3J_{9,10}$ = 10.2 Hz, $^3J_{9,8}$ = 6.9 Hz, 1 H, 9-H), 5.18 (ddt, $^3J_{10',9}$ = 17.0 Hz, $^2J_{10',10}$ = 1.6 Hz, $^4J_{10',8}$ = 1.6 Hz, 1 H, 10-H'), 5.10 (ddt, $^3J_{10,9}$ = 10.2 Hz, $^2J_{10,10'}$ = 2.0 Hz, $^4J_{10,8}$ = 1.1 Hz, 1 H, 10-H), 4.92 (t, $^3J_{NH,1}$ = 6.4 Hz, 1 H, 16-NH), 4.33 (ddd, $^3J_{5,NH}$ = 8.2 Hz, $^3J_{5,11}$ = 8.2 Hz, $^3J_{5,6}$ = 4.4 Hz, 1 H, 5-H), 4.19 – 4.12 (m, 2 H, 2-H, 6-H), 4.08 (td, $^3J_{7,8}$ = 7.1 Hz, $^3J_{7,6}$ = 2.8 Hz, 1 H, 7-H), 3.89 (dt, $^3J_{11,5}$ = 8.6 Hz, $^3J_{11,12}$ = 4.3 Hz, 1 H, 11-H), 3.75 (dd, $^2J_{12,12'}$ = 12.1 Hz, $^3J_{12,11}$ = 4.7 Hz, 1 H, 12-H), 3.67 (dd, $^2J_{12',12}$ = 12.2 Hz, $^3J_{12',11}$ = 4.0 Hz, 1 H, 12-H'), 3.55 (m, 1 H, 12-OH), 3.23 (ddd, $^2J_{1,1'}$ = 14.1 Hz, $^3J_{1,2}$ = 7.2 Hz, $^3J_{1,NH}$ = 5.1 Hz, 1 H, 1-H), 3.13 (ddd, $^2J_{1',1}$ = 14.2 Hz, $^3J_{1',2}$ = 5.4 Hz, $^3J_{1',NH}$ = 5.4 Hz, 1 H, 1-H'), 2.99 (m, 1 H, 6-OH), 2.46 (m, 2 H, 8-H), 2.42 (m, 2 H, 3-H), 1.44 (s, 9 H, 18-H), 0.89 (s, 9 H, 15-H), 0.10 (s, 3 H, 13-H), 0.08 (s, 3 H, 13-H').

^{13}C-NMR (125 MHz, $CDCl_3$): δ = 171.5 (s, C-4), 156.5 (s, C-16), 134.2 (d, C-9), 117.7 (t, C-10), 81.6 (d, C-11), 80.9 (d, C-7), 80.0 (s, C-17), 72.8 (d, C-6), 68.8 (d, C-2), 62.9 (t, C-12), 54.7 (d, C-5), 45.5 (t, C-1), 42.1 (t, C-3), 34.0 (t, C-8), 28.5 (q, C-18), 25.9 (q, C-15), 18.1 (s, C-14), –4.7 (q, C-13), –4.8 (q, C-13').

**Optical rotation:**	$[\alpha]_D^{20}$= –20.1 (c = 1.0, $CHCl_3$)	
**HRMS (CI):**	calculated	found
$C_{17}H_{31}N_2O_7$ $[M\text{-}TBS+2H]^+$:	375.2126	375.2128

***tert*-Butyl ((*R*)-4-(((2*S*,3*R*,4*S*,5*S*)-5-allyl-4-hydroxy-2-(hydroxymethyl)tetrahydrofuran-3-yl)amino)-2-((*tert*-butyldimethylsilyl)oxy)-4-oxobutyl)carbamate 20d**

Ammonium sulfide in H_2O (7.5 mL, 40wt%) was added to a 0 °C cold solution of C-furanoside **12d** (470 mg, 1.50 mmol) in MeCN (7.5 mL). The biphasic mixture was stirred for 16 h while slowly reaching room temperature. After the addition of sat. $NaHCO_3$ solution (20.0 mL), the mixture was extracted thrice with EtOAc. The combined organic layers were dried with $MgSO_4$ and concentrated in vacuo to give the crude amine (300 mg, 939 µmol, 63%, 90wt% purity) as a colorless resin.

1.0 M TBAF in THF (1.07 mL, 1.07 mmol, 1.15 eq.) was added to a 0 °C cold solution of the above-prepared crude amine (298 mg, 933 µmol) in anhydrous THF (4.7 mL). The resulting solution was stirred for 2 h while slowly reaching room temperature. After adding 1.0 M HCl_{aq} (1.07 mL,

1.07 mmol, 1.15 eq.), the reaction mixture was concentrated in vacuo. The residue was dissolved in anhydrous CH_2Cl_2 (9.4 mL) before diazoketone **8** (444 mg, 1.22 mmol, 1.3 eq.) was added. The resulting yellow solution was irradiated with a blue LED (405 nm, 18 W) at room temperature for 3 h. After dilution with EtOAc, the mixture was washed with 1.0 M HCl_{aq}, sat. $NaHCO_3$ solution, and brine. The organic layer was dried with $MgSO_4$ and concentrated in vacuo. The residue was purified by automated reversed phase column chromatography (C18 spherical, H_2O:MeCN 10% to 90% MeCN) to give compound **20d** (55 mg, 113 µmol, 12%) as a colorless resin.

LC-MS: t_R (20d) = 1.29 min (short method)

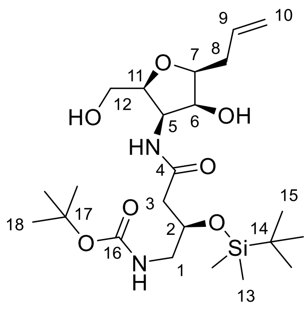

20d

^{1}H-NMR (500 MHz, $CDCl_3$): δ = 6.71 (d, $^3J_{NH,5}$ = 7.3 Hz , 1 H, 4-NH), 5.85 (ddt, $^3J_{9,10'}$ = 17.2 Hz, $^3J_{9,10}$ = 10.2 Hz, $^3J_{9,8}$ = 7.0 Hz, 1 H, 9-H), 5.17 (ddt, $^3J_{10',9}$ = 17.1 Hz, $^2J_{10',10}$ = 1.7 Hz, $^4J_{10',8}$ = 1.5 Hz, 1 H, 10-H'), 5.08 (ddt, $^3J_{10,9}$ = 10.2 Hz, $^2J_{10,10'}$ = 2.1 Hz, $^4J_{10,8}$ = 1.1 Hz, 1 H, 10-H), 4.93 (dd, $^3J_{NH,1}$ = 9.4 Hz, $^3J_{NH,1'}$ = 3.4 Hz, 1 H, 16-NH), 4.63 (ddd, $^3J_{5,11}$ = 8.9 Hz, $^3J_{5,NH}$ = 7.3 Hz, $^3J_{5,6}$ = 4.6 Hz, 1 H, 5-H), 4.38 (d, $^3J_{11,5}$ = 8.8 Hz, 1 H, 11-H), 4.23 (m, 1 H, 2-H), 3.85 (dd, $^3J_{6,5}$ = 4.7 Hz, $^3J_{6,7}$ = 2.5 Hz, 1 H, 12-H), 3.81 (td, $^3J_{7,8}$ = 6.9 Hz, $^3J_{7,6}$ = 2.4 Hz, 1 H, 7-H), 3.75 (dd, $^2J_{12,12'}$ = 12.2 Hz, $^3J_{12,11}$ = 2.0 Hz, 1 H, 12-H), 3.55 (d, $^2J_{12',12}$ = 12.2 Hz, 1 H, 12-H'), 3.40 (ddd, $^2J_{1,1'}$ = 13.8 Hz, $^3J_{1,NH}$ = 9.4 Hz, $^3J_{1,2}$ = 3.8 Hz, 1 H, 1-H), 3.03 (ddd, $^2J_{1',1}$ = 14.3 Hz, $^3J_{1',2}$ = 3.6 Hz, $^3J_{1',NH}$ = 3.6 Hz, 1 H, 1-H'), 2.52 – 2.39 (m, 3 H, 3-H, 8-H), 2.35 (dd, $^2J_{3',3}$ = 14.3 Hz, $^3J_{3',2}$ = 3.6 Hz, 1 H, 3-H'), 1.44 (s, 9 H, 18-H), 0.90 (s, 9 H, 15-H), 0.11 (s, 3 H, 13-H), 0.10 (s, 3 H, 13-H').

^{13}C-NMR (125 MHz, $CDCl_3$): δ = 170.1 (s, C-4), 157.1 (s, C-16), 134.5 (d, C-9), 117.4 (t, C-10), 80.7 (s, C-17), 80.5 (d, C-7), 78.5 (d, C-11), 71.5 (d, C-6), 69.1 (d, C-2), 60.4 (t, C-12), 54.9 (d, C-5), 44.8 (t, C-1), 41.4 (t, C-3), 33.6 (t, C-8), 28.5 (q, C-18), 25.9 (q, C-15), 18.2 (s, C-14), –4.7 (q, C-13), –4.8 (q, C-13').

**Optical rotation:**	$[\alpha]_D^{20}$= +48.5 (c = 1.0, $CHCl_3$)	
**HRMS (ESI):**	calculated	found
$C_{23}H_{45}N_2O_7Si$ $[M+H]^+$:	489.2991	489.2997

Methyl (2*S*,3*S*,4*S*,5*R*)-5-allyl-3-((tert-butoxycarbonyl)amino)-4-((*tert*-butyldimethylsilyl)oxy)-tetrahydrofuran-2-carboxylate 21

Triphenylphosphine (77.0 mg, 293 µmol, 1.0 eq.) was added to a solution of compound **15c** (100 mg, 293 µmol) in THF:H_2O (1.56 mL, 25:1). The reaction mixture was stirred for 16 h at room

temperature before another portion of triphenylphosphine (7.7 mg, 29.3 µmol, 0.1 eq.) was added. After another 2 h at room temperature, H_2O (600 µL), $NaHCO_3$ (49.0 mg, 583 µmol, 2.0 eq.), and Boc_2O (81.0 µL, 350 µmol, 1.2 eq.) were added at 0 °C. The reaction mixture was stirred for 16 h while slowly reaching room temperature. After dilution with EtOAc, the mixture was washed three times with brine, dried with $MgSO_4$, and concentrated in vacuo. The crude product was purified by column chromatography (silica, PE:EtOAc 9:1) to give Boc-protected amine **21** (119 mg, 286 µmol, 98%) as a colorless oil.

TLC: R_f (21) = 0.44 (silica, PE:EtOAc 8:2)

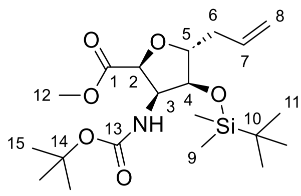

21

^{1}H-NMR (400 MHz, $CDCl_3$): δ = 5.82 (ddt, $^3J_{7,8'}$ = 17.7 Hz, $^3J_{7,8}$ = 10.2 Hz, $^3J_{7,6}$ = 7.1 Hz, 1 H, 7-H), 5.39 (d, $^3J_{NH,3}$ = 8.8 Hz, 1 H, 13-NH), 5.17 (ddt, $^3J_{8',7}$ = 17.2 Hz, $^2J_{8',8}$ = 1.6 Hz, $^4J_{8',6}$ = 1.6 Hz, 1 H, 8-H'), 5.14 (m, 1 H, 8-H), 4.57 (d, $^3J_{2,3}$ = 7.5 Hz, 1 H, 2-H), 4.36 (ddd, $^3J_{3,NH}$ = 8.8 Hz, $^3J_{3,2}$ = 7.5 Hz, $^3J_{3,4}$ = 5.2 Hz, 1 H, 3-H), 4.14 (td, $^3J_{5,6}$ = 6.4 Hz, $^3J_{5,4}$ = 2.6 Hz, 1 H, 5-H), 3.99 (dd, $^3J_{4,3}$ = 5.2 Hz, $^3J_{4,5}$ = 2.6 Hz, 1 H, 4-H), 3.75 (s, 3 H, 12-H), 2.35 (m, 2 H, 6-H), 1.45 (s, 9 H, 15-H), 0.88 (s, 9 H, 11-H), 0.05 (s, 6 H, 9-H).
^{13}C-NMR (100 MHz, $CDCl_3$): δ = 171.9 (s, C-1), 155.4 (s, C-13), 133.5 (d, C-7), 118.5 (t, C-8), 85.8 (d, C-5), 79.8 (s, C-14), 76.1 (d, C-2), 73.8 (d, C-4), 55.1 (d, C-3), 52.2 (q, C-12), 38.2 (t, C-6), 28.5 (q, C-15), 25.7 (q, C-11), 18.1 (s, C-10), –4.7 (q, C-9'), –4.9 (q, C-9).

**Optical rotation:**	$[\alpha]_D^{20}$= +23.7 (c = 1.0, $CHCl_3$)	
**HRMS (ESI):**	calculated	found
$C_{20}H_{38}NO_6Si$ $[M+H]^+$:	416.2463	416.2476

Methyl *N*-((*Z*)-2-((*R*)-3-((2*R*,3*S*,4*S*,5*R*)-5-allyl-3-((*R*)-4-((*tert*-butoxycarbonyl)amino)-3-((*tert*-butyldimethylsilyl)oxy)butanamido)-4-((*tert*-butyldimethylsilyl)oxy)tetrahydrofuran-2-carboxamido)-5-oxopyrrolidin-2-ylidene)acetyl)-*N*-methylglycinate 22a

0.20 M $LiOH_{aq}$ (741 µL, 148 µmol, 1.1 eq.) was slowly added to a 0 °C cold solution of **17a** (85.0 mg, 135 µmol) in THF (700 µL). The resulting mixture was stirred for 2 h while slowly reaching room temperature. After full conversion, the mixture was acidified with 0.1 M HCl_{aq} and extracted with CH_2Cl_2. The combined organic layers were dried with $MgSO_4$ and concentrated in vacuo to give the crude carboxylic acid as a colorless resin.

4.0 M HCl in dioxane (806 µL, 3.22 mmol, 10 eq.) was added to a solution of dipeptide **4** (110 mg, 322 µmol) in CH_2Cl_2 (800 µL) at 0 °C. After stirring for 2 h, the reaction mixture was concentrated in vacuo. The residue was partitioned between 1.0 M K_2CO_3 solution and CH_2Cl_2. The aqueous

phase was extracted with CH_2Cl_2. The combined organic layers were dried with $MgSO_4$ and concentrated in vacuo to give the crude amine (52.0 mg, 216 µmol, 67%) as a colorless resin.

The above-prepared crude carboxylic acid and amine (48.7 mg, 202 µmol, 1.5 eq.) were dissolved in anhydrous DMF (1.3 mL). After cooling to 0 °C, EDC·HCl (28.4 mg, 148 µmol, 1.1 eq.) and HOBt (4.1 mg, 27.0 µmol, 0.2 eq.) were added, and the resulting mixture was stirred for 16 h while slowly reaching room temperature. The reaction mixture was diluted with EtOAc and washed with 5wt% $LiCl_{aq}$, 1.0 M HCl_{aq}, sat. $NaHCO_3$ solution and brine. The organic layer was dried over $MgSO_4$ and concentrated in vacuo. The crude product was purified by automated reversed phase column chromatography (C18 spherical, H_2O:MeCN 10% to 90% MeCN) to give tetrapeptide **22a** (108 mg, 129 µmol, 96%, dr 90:10) as a white foam.

LC-MS: t_R (22a) = 5.00 min (long method)

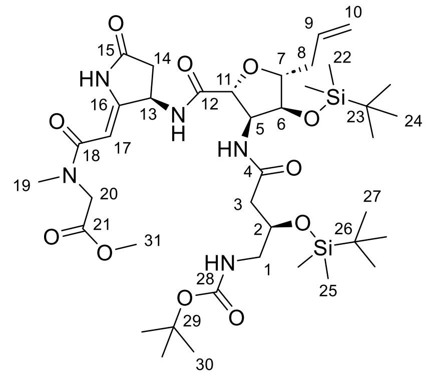

22a

^{1}H-NMR (500 MHz, DMSO-d$_6$, 373 K): δ = 10.29 (s, 1 H, 15-NH), 8.11 (d, $^3J_{NH,13}$ = 8.2 Hz, 1 H, 12-NH), 7.30 (d, $^3J_{NH,5}$ = 8.2 Hz, 1 H, 4-NH), 6.09 (m, 1 H, 28-NH), 5.89 (ddt, $^3J_{9,10}$ = 17.1 Hz, $^3J_{9,10'}$ = 10.2 Hz, $^3J_{9,8}$ = 6.9 Hz, 1 H, 9-H), 5.37 (m, 1 H, 17-H), 5.17 – 5.06 (m, 3 H, 10-H, 13-H), 4.38 (m, 1 H, 5-H), 4.17 – 4.08 (m, 4 H, 2-H, 11-H, 20-H), 4.02 (m, 1 H, 6-H), 3.91 (td, $^3J_{7,8}$ = 6.4 Hz, $^3J_{7,6}$ = 4.2 Hz, 1 H, 7-H), 3.67 (s, 3 H, 31-H), 3.10 – 2.96 (m, 2 H, 1-H), 3.02 (s, 3 H, 19-H), 2.75 (dd, $^2J_{14,14'}$ = 17.5 Hz, $^3J_{14,13}$ = 9.5 Hz, 1 H, 14-H‘), 2.42 – 2.33 (m, 4 H, 3-H‘, 8-H, 14-H), 2.24 (dd, $^2J_{3,3'}$ = 14.6 Hz, $^3J_{3,2}$ = 5.9 Hz, 1 H, 3-H), 1.45 (s, 9 H, 30-H), 0.91 (s, 9 H, 24-H), 0.87 (s, 9 H, 27-H), 0.08 (s, 6 H, 25-H), 0.07 (s, 3 H, 22‘-H), 0.06 (s, 3 H, 22-H).

^{13}C-NMR (125 MHz, DMSO-d$_6$, 298 K): δ = 174.7 (s, C-15), 169.9 (s, C-21), 169.9 (s, C-12), 169.3 (s, C-4), 167.8 (s, C-18), 157.6 (s, C-16), 155.6 (s, C-28), 134.5 (d, C-9), 117.5 (t, C-10), 87.1 (d, C-17), 84.3 (d, C-7), 80.0 (s, C-29), 77.5 (d, C-11), 74.2 (d, C-6), 68.5 (d, C-2), 54.2 (d, C-5), 51.7 (q, C-31), 48.9 (t, C-20), 46.7 (d, C-13), 45.4 (t, C-1), 41.8 (t, C-3), 37.4 (t, C-8), 36.3 (q, C-19), 34.4 (t, C-14), 28.3 (q, C-30), 25.8 (q, C-27), 25.7 (q, C-24), 17.8 (s, C-26), 17.7 (s, C-23), –4.75 (q, C-25‘), –4.83 (q, C-22‘), –4.90 (q, C-25), –4.92 (q, C-22).

Optical rotation: $[\alpha]_D^{20}$= –2.9 (c = 1.0, $CHCl_3$)

**HRMS (ESI):**	calculated	found
$C_{39}H_{70}N_5O_{11}Si_2$ [M+H]$^+$:	840.4605	840.4603

Methyl *N*-((*Z*)-2-((*R*)-3-((2*R*,3*R*,4*S*,5*S*)-5-allyl-3-((*R*)-4-((*tert*-butoxycarbonyl)amino)-3-((*tert*-butyldimethylsilyl)oxy)butanamido)-4-hydroxytetrahydrofuran-2-carboxamido)-5-oxopyrrolidin-2-ylidene)acetyl)-*N*-methylglycinate 22b

0.40 M $LiOH_{aq}$ (351 µL, 141 µmol, 1.1 eq.) was slowly added to a 0 °C cold solution of compound **17b** (66.0 mg, 128 µmol) in MeCN (640 µL). The resulting mixture was stirred for 16 h while slowly reaching room temperature. After full conversion, the mixture was acidified with 0.1 M HCl_{aq} and extracted with CH_2Cl_2. The combined organic layers were dried with $MgSO_4$ and concentrated in vacuo to give the crude carboxylic acid as a colorless resin.

4.0 M HCl in dioxane (586 µL, 2.34 mmol, 10 eq.) was added to a solution of dipeptide **4** (80 mg, 234 µmol) in CH_2Cl_2 (400 µL) at 0 °C. After stirring for 3 h, the reaction mixture was concentrated in vacuo. The residue was partitioned between 1.0 M K_2CO_3 solution and CH_2Cl_2. The aqueous phase was extracted with CH_2Cl_2. The combined organic layers were dried with $MgSO_4$ and concentrated in vacuo to give the crude amine (37.0 mg, 153 µmol, 65%) as a colorless resin.

The above-prepared crude carboxylic acid and amine (37.0 mg, 153 µmol, 1.2 eq.) were dissolved in anhydrous DMF (1.1 mL). After cooling to 0 °C, EDC·HCl (26.8 mg, 140 µmol, 1.1 eq.) and HOBt (3.9 mg, 25.0 µmol, 0.2 eq.) were added, and the resulting mixture was stirred for 16 h while slowly reaching room temperature. The reaction mixture was diluted with EtOAc and washed with 5wt% $LiCl_{aq}$, 1.0 M HCl_{aq}, sat. $NaHCO_3$ solution and brine. The organic layer was dried over $MgSO_4$ and concentrated in vacuo. The crude product was purified by automated reversed phase column chromatography (C18 spherical, H_2O:MeCN 10% to 90% MeCN) followed by preparative HPLC (H_2O:MeCN 10% to 100% MeCN) to give tetrapeptide **22b** (64.0 mg, 88.0 µmol, 69%, dr >99:1) as a colorless resin. An epimer/isomer (6.1 mg, 8.4 µmol, 7%) was separated during the preparative HPLC step.

LC-MS: t_R (22b) = 3.09 min (long method)

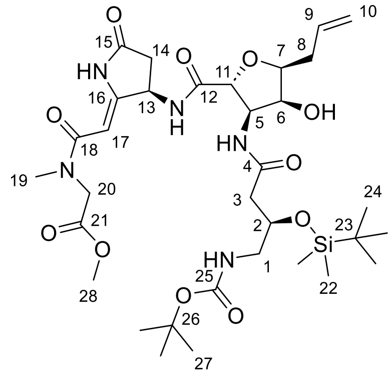

22b

^{1}H-NMR (500 MHz, DMSO-d$_6$, 373 K): δ = 10.30 (s, 1 H, 15-NH), 8.21 (d, $^3J_{NH,13}$ = 8.6 Hz, 1 H, 12-NH), 7.42 (d, $^3J_{NH,5}$ = 8.1 Hz, 1 H, 4-NH), 6.16 (m, 1 H, 25-NH), 5.86 (ddt, $^3J_{9,10}$ = 17.1 Hz, $^3J_{9,10'}$ = 10.3 Hz, $^3J_{9,8}$ = 6.9 Hz, 1 H, 9-H), 5.40 (s, 1 H, 17-H), 5.13 (ddt, $^3J_{10,9}$ = 17.5 Hz, $^2J_{10,10'}$ = 1.8 Hz, $^4J_{10,8}$ = 1.8 Hz, 1 H, 10-H), 5.09 (m, 1 H, 13-H), 5.03 (ddt, $^3J_{10',9}$ = 10.3 Hz, $^2J_{10',10}$ = 2.3 Hz, $^4J_{10',8}$ = 1.3 Hz, 1 H, 10-H'),

4.89 (d, $^3J_{OH,6}$ = 5.3 Hz, 1 H, 6-OH), 4.47 (m, 1 H, 5-H), 4.15 (m, 2 H, 20-H), 4.13 – 4.08 (m, 2 H, 2-H, 7-H), 4.06 (d, $^3J_{11,5}$ = 8.3 Hz, 1 H, 11-H), 4.00 (ddd, $^3J_{6,OH}$ = 5.2 Hz, $^3J_{6,7}$ = 5.2 Hz, $^3J_{6,5}$ = 2.9 Hz, 1 H, 6-H), 3.67 (s, 3 H, 28-H), 3.07 – 3.00 (m, 5 H, 1-H, 19-H), 2.73 (dd, $^2J_{14,14'}$ = 17.5 Hz, $^3J_{14,13}$ = 9.6 Hz, 1 H, 14-H), 2.46 – 2.37 (m, 2 H, 8-H, 14-H'), 2.37 – 2.25 (m, 3 H, 3-H, 8-H'), 1.40 (s, 9 H, 27-H), 0.88 (s, 9 H, 24-H), 0.06 (s, 3 H, 22-H), 0.05 (s, 3 H, 22'-H).

^{13}C-NMR (125 MHz, DMSO-d$_6$, 373 K): δ = 173.8 (s, C-15), 170.9 (s, C-12), 169.3 (s, C-4), 169.1 (s, C-21), 167.6 (s, C-18), 156.6 (s, C-16), 155.1 (s, C-25), 134.5 (d, C-9), 115.9 (t, C-10), 87.2 (d, C-17), 81.6 (d, C-7), 78.9 (d, C-11), 77.3 (s, C-26), 70.5 (d, C-6), 68.4 (d, C-2), 56.2 (d, C-5), 51.0 (q, C-28), 46.5 (d, C-13), 45.5 (t, C-1), 41.5 (t, C-3), 34.2 (t, C-14), 32.7 (t, C-8), 27.8 (q, C-27), 25.3 (q, C-24), 17.2 (s, C-23), –5.3 (q, C-22), –5.4 (q, C-22'), C-20 and C-19 were not observed.

Optical rotation: $[\alpha]_D^{20}$= +2.4 (c = 1.0, $CHCl_3$)

**HRMS (ESI):**	calculated	found
$C_{33}H_{56}N_5O_{11}Si$ [M+H]$^+$:	726.3740	726.3750

Methyl *N*-((*Z*)-2-((*R*)-3-((2*S*,3*S*,4*S*,5*R*)-5-allyl-3-((*R*)-4-((*tert*-butoxycarbonyl)amino)-3-((*tert*-butyldimethylsilyl)oxy)butanamido)-4-((*tert*-butyldimethylsilyl)oxy)tetrahydrofuran-2-carboxamido)-5-oxopyrrolidin-2-ylidene)acetyl)-*N*-methylglycinate 22c

0.30 M $LiOH_{aq}$ (277 µL, 83.0 µmol, 1.05 eq.) was slowly added to a 0 °C cold solution of compound **17c** (49.7 mg, 78.8 µmol) in THF (500 µL). The resulting mixture was stirred for 2 h while slowly reaching room temperature. After full conversion, the mixture was acidified with 0.1 M HCl_{aq} and extracted with CH_2Cl_2. The combined organic layers were dried with $MgSO_4$ and concentrated in vacuo to give the crude carboxylic acid as a colorless resin.

4.0 M HCl in dioxane (732 µL, 2.93 mmol, 10 eq.) was added to a solution of dipeptide **4** (100 mg, 293 µmol) in CH_2Cl_2 (500 µL) at 0 °C. After stirring for 2 h, the reaction mixture was concentrated in vacuo. The residue was partitioned between sat. $NaHCO_3$ solution and CH_2Cl_2. The aqueous phase was extracted with CH_2Cl_2. The combined organic layers were dried with $MgSO_4$ and concentrated in vacuo to give the crude amine (44.0 mg, 182 µmol, 62%) as a colorless resin.

The above-prepared crude carboxylic acid and amine (37.5 mg, 156 µmol, 2.0 eq.) were dissolved in anhydrous DMF (800 µL). After cooling to 0 °C, EDC·HCl (16.4 mg, 86.0 µmol, 1.1 eq.) and HOBt (2.4 mg, 16.0 µmol, 0.2 eq.) were added, and the resulting mixture was stirred for 16 h while slowly reaching room temperature. The reaction mixture was diluted with EtOAc and washed with 5wt% $LiCl_{aq}$, 1.0 M HCl_{aq}, sat. $NaHCO_3$ solution and brine. The organic layer was dried over $MgSO_4$ and concentrated in vacuo. The crude product was purified by automated reversed phase column chromatography (C18 spherical, H_2O:MeCN 10% to 90% MeCN) to give tetrapeptide **22c** (47.0 mg, 56.0 µmol, 71%, dr 80:20) as a white foam.

LC-MS: t_R (22c) = 1.73 min (short method)

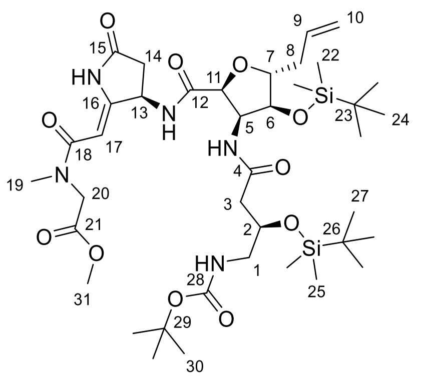

22c

^{1}H-NMR (500 MHz, DMSO-d$_6$, 373 K): δ = 10.30 (s, 1 H, 15-NH), 8.16 (d, $^3J_{NH,13}$ = 7.5 Hz, 1 H, 12-NH), 7.15 (d, $^3J_{NH,5}$ = 8.7 Hz, 1 H, 4-NH), 6.12 (m, 1 H, 28-NH), 5.87 (m, 1 H, 9-H), 5.38 (m, 1 H, 17-H), 5.16 (ddt, $^3J_{10,9}$ = 17.1 Hz, $^2J_{10,10'}$ = 1.8 Hz, $^4J_{10,8}$ = 1.8 Hz, 1 H, 10-H), 5.11 (ddt, $^3J_{10',9}$ = 10.2 Hz, $^2J_{10',10}$ = 2.0 Hz, $^4J_{10',8}$ = 1.1 Hz, 1 H, 10-H'), 4.88 (m, 1 H, 13-H), 4.59 (m, 1 H, 5-H), 4.37 (d, $^3J_{11,5}$ = 6.8 Hz, 1 H, 11-H), 4.14 (m, 2 H, 20-H), 4.12 – 4.06 (m, 3 H, 2-H, 6-H, 7-H), 3.67 (s, 3 H, 31-H), 3.09 – 2.99 (m, 2 H, 1-H), 3.02 (s, 3 H, 19-H), 2.69 (dd, $^2J_{14,14'}$ = 17.5 Hz, $^3J_{14,13}$ = 9.4 Hz, 1 H, 14-H), 2.45 (dd, $^2J_{14',14}$ = 17.5 Hz, $^3J_{14',13}$ = 5.2 Hz, 1 H, 14-H'), 2.41 – 2.20 (m, 4 H, 3-H, 8-H), 1.39 (s, 9 H, 30-H), 0.88 (s, 9 H, 24-H), 0.87 (s, 9 H, 27-H), 0.08 (s, 3 H, 25-H/22-H), 0.07 (s, 3 H, 25-H/22-H), 0.06 (s, 3 H, 25-H/22-H), 0.05 (s, 3 H, 25-H/22-H).

^{13}C-NMR (125 MHz, DMSO-d$_6$, 373 K): δ = 173.7 (s, C-15), 169.8 (s, C-12), 169.1 (s, C-21), 168.4 (s, C-4), 167.5 (s, C-18), 156.3 (s, C-16), 155.0 (s, C-28), 133.6 (d, C-9), 116.9 (t, C-10), 87.1 (d, C-17), 83.3 (d, C-7), 77.2 (s, C-29), 76.1 (d, C-11), 73.9 (d, C-6), 67.9 (d, C-2), 52.8 (d, C-5), 51.0 (q, C-31), 47.3 (d, C-13), 45.6 (t, C-1), 41.7 (t, C-3), 36.7 (t, C-8), 34.0 (t, C-14), 27.8 (q, C-30), 25.3 (q, C-27), 25.1 (q, C-24), 17.2 (s, C-26), 17.1 (s, C-23), –5.28 (q, C-25/C-22), –5.33 (q, C-25/C-22), –5.40 (q, C-25/C-22), –5.47 (q, C-25/C-22), C-20 and C-19 were not observed.

Selected epimer/isomer signals:

^{1}H-NMR (500 MHz, DMSO-d$_6$, 373 K): δ = 8.21 (d, $^3J_{NH,13}$ = 8.6 Hz, 1 H, 12-NH), 7.21 (d, $^3J_{NH,5}$ = 8.6 Hz, 1 H, 4-NH), 5.19 (m, 1 H, 13-H), 4.44 (d, $^3J_{11,5}$ = 7.1 Hz, 1 H, 11-H), 1.40 (s, 9 H, 30-H), 0.88 (s, 9 H, 24-H/27-H), 0.86 (s, 9 H, 24-H/27-H), 0.09 (s, 3 H, 25-H/22-H), 0.07 (s, 3 H, 25-H/22-H), 0.06 (s, 3 H, 25-H/22-H), 0.03 (s, 3 H, 25-H/22-H).

**Optical rotation:**	$[\alpha]_D^{20}$= +21.2 (c = 1.0, DMSO)	
**HRMS (ESI):**	calculated	found
$C_{39}H_{70}N_5O_{11}Si_2$ $[M+H]^+$:	840.4605	840.4604

Methyl (*tert*-butoxycarbonyl)-D-tryptophylglycinate SI-6

Boc-D-Trp-OH (1.00 g, 3.29 mmol) and H-Gly-OMe·HCl (454 mg, 3.61 mmol, 1.1 eq.) were dissolved in anhydrous CH_2Cl_2 (33 ml) and cooled to 0 °C. EDC·HCl (630 mg, 3.29 mmol, 1.0 eq.), HOBt (503 mg, 3.29 mmol, 1.0 eq.), and NMM (369 µl, 3.29 mmol, 1.0 eq.) were added subsequentially, and the reaction mixture was stirred for 16 h while slowly reaching room temperature. The reaction mixture was diluted with EtOAc and washed with 1.0 M HCl_{aq}, sat.

$NaHCO_3$ solution and brine. The organic layer was dried with $MgSO_4$ and concentrated in vacuo. The crude product was purified by automated column chromatography (silica, pentane:EtOAc 0% to 60% EtOAc) to give the dipeptide **SI-6** (1.10 g, 2.78 mmol, 85%) as a white foam.

TLC: R_f (SI-6) = 0.15 (silica, pentane:EtOAc 1:1)

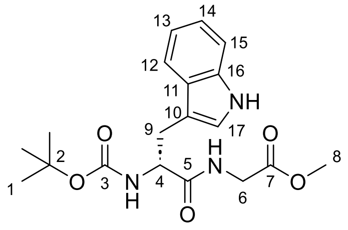

SI-6

^{1}H-NMR (500 MHz, $CDCl_3$): δ = 8.43 (m, 1 H, 17-NH), 7.62 (d, $^3J_{15,14}$ = 7.9 Hz, 1 H, 15-H), 7.34 (d, $^3J_{12,13}$ = 8.2 Hz, 1 H, 12-H), 7.18 (ddd, $^3J_{13,12}$ = 8.0 Hz, $^3J_{13,14}$ = 6.9 Hz, $^4J_{13,15}$ = 1.2 Hz, 1 H, 13-H), 7.11 (ddd, $^3J_{14,15}$ = 8.0 Hz, $^3J_{14,13}$ = 7.0 Hz, $^4J_{14,12}$ = 1.0 Hz, 1 H, 14-H), 7.07 (m, 1 H, 17-H), 6.47 (t, $^3J_{NH,6}$ = 5.4 Hz, 1 H, 5-NH), 5.23 (d, $^3J_{NH,4}$ = 8.6 Hz, 1 H, 3-NH), 4.51 (m, 1 H, 4-H), 3.90 (m, 2 H, 6-H), 3.67 (s, 3 H, 8-H), 3.31 (m, 1 H, 9-H'), 3.21 (m, 1 H, 9-H), 1.42 (s, 9 H, 1-H).

^{13}C-NMR (125 MHz, $CDCl_3$): δ = 172.2 (s, C-5), 170.0 (s, C-7), 155.7 (s, C-3), 136.3 (s, C-16), 127.6 (s, C-11), 123.5 (d, C-17), 122.3 (d, C-13), 119.7 (d, C-14), 118.8 (d, C-15), 111.4 (d, C-12), 110.4 (s, C-10), 80.3 (s, C-2), 55.2 (d, C-4), 52.4 (q, C-8), 41.3 (t, C-6), 28.4 (q, C-1), 28.3 (t, C-9).

**Optical rotation:**	$[\alpha]_D^{20}$= +7,6 (c = 1.0, $CHCl_3$)	
**HRMS (CI):**	calculated	found
$C_{19}H_{27}N_3O_5$ $[M+2H]^+$:	377.1945	377.1934

Methyl *N*-((*Z*)-2-((*R*)-3-((2*R*,3*S*,4*S*,5*R*)-5-allyl-3-((*R*)-4-((*tert*-butoxycarbonyl)amino)-3-((*tert*-butyldimethylsilyl)oxy)butanamido)-4-((*tert*-butyldimethylsilyl)oxy)tetrahydrofuran-2-carboxamido)-5-oxopyrrolidin-2-ylidene)acetyl)-*N*-methylglycyl-D-tryptophylglycinate 23a

0.20 M $LiOH_{aq}$ (570 µL, 114 µmol, 1.1 eq.) was slowly added to a 0 °C cold solution of tetrapeptide **22a** (87.0 mg, 104 µmol) in THF (500 µL). The resulting mixture was stirred for 2 h while slowly reaching room temperature. After full conversion, the mixture was acidified with 0.1 M HCl_{aq} and extracted with CH_2Cl_2. The combined organic layers were dried with $MgSO_4$ and concentrated in vacuo to give the crude carboxylic acid as a white foam.

4.0 M HCl in dioxane (531 µL, 2.13 mmol, 10 eq.) was added to a solution of dipeptide **SI-6** (83.2 mg, 208 µmol) in CH_2Cl_2 (50 µL) at 0 °C. After stirring for 1 h, the reaction mixture was concentrated in vacuo to give the crude amine as hydrochloride salt.

The above-prepared crude carboxylic acid and amine hydrochloride (2.0 eq.) were dissolved in anhydrous DMF (1.0 mL). After cooling to 0 °C, NMM (47.0 µL, 427 µmol, 4.1 eq.) and HBTU (43.4 mg, 115 µmol, 1.1 eq.) were subsequently added, and the resulting mixture was stirred for 16 h while slowly reaching room temperature. The reaction mixture was diluted with EtOAc and

washed with 5wt% $LiCl_{aq}$, 1.0 M HCl_{aq}, sat. $NaHCO_3$ solution and brine. The organic layer was dried over $MgSO_4$ and concentrated in vacuo. The crude product was purified by automated reversed phase column chromatography (C18 spherical, H_2O:MeCN 10% to 90% MeCN) to give hexapeptide **23a** (85.0 mg, 78.0 µmol, 75%, dr 97:3) as a white foam. The epimer from the previous coupling was mainly separated during the column chromatography (hence the improved diastereomeric ratio).

LC-MS: t_R (23a) = 1.73 min (short method)

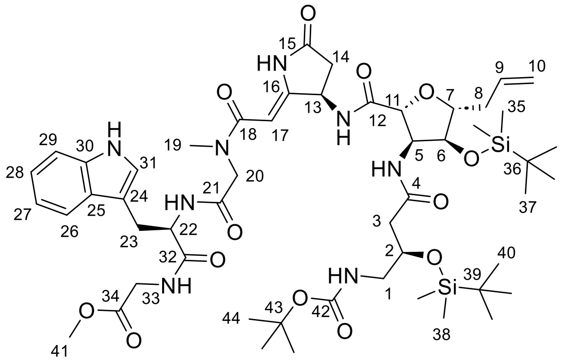

23a

^{1}H-NMR (500 MHz, DMSO-d$_6$): δ = 10.79 (m, 1 H, 31-NH), 10.39 (s, 1 H, 15-NH), 8.45 – 8.36 (m, 2 H, 12-NH, 32-NH), 8.09 (d, $^3J_{NH,22}$ = 8.5 Hz, 1 H, 21-NH), 7.76 (d, $^3J_{NH,5}$ = 8.3 Hz, 1 H, 4-NH), 7.57 (d, $^3J_{29,28}$ = 7.9 Hz, 1 H, 29-H), 7.31 (d, $^3J_{26,27}$ = 8.1 Hz, 1 H, 26-H), 7.13 (s, 1 H, 31-H), 7.04 (ddd, $^3J_{27,26}$ = 8.1 Hz, $^3J_{27,28}$ = 6.9 Hz, $^4J_{27,29}$ = 1.2 Hz, 1 H, 27-H), 6.97 (ddd, $^3J_{28,29}$ = 8.0 Hz, $^3J_{28,27}$ = 6.9 Hz, $^4J_{28,26}$ = 1.1 Hz, 1 H, 28-H), 6.66 (m, 1 H, 42-NH), 5.85 (m, 1 H, 9-H), 5.29 (s, 1 H, 17-H), 5.15 – 5.02 (m, 3 H, 10-H, 13-H), 4.57 (m, 1 H, 22-H), 4.33 (ddd, $^3J_{5,11}$ = 7.2 Hz, $^3J_{5,NH}$ = 7.2 Hz, $^3J_{5,6}$ = 7.2 Hz, 1 H, 5-H), 4.10 (d, $^3J_{11,5}$ = 6.9 Hz, 1 H, 11-H), 4.03 (m, 1 H, 2-H), 3.98 – 3.76 (m, 6 H, 6-H, 7-H, 20-H, 33-H), 3.64 (s, 3 H, 41-H), 3.17 (dd, $^2J_{23',23}$ = 14.7 Hz, $^3J_{23',22}$ = 4.8 Hz, 1 H, 23-H‘), 3.03 – 2.88 (m, 3 H, 1-H, 23-H), 2.84 (s, 3 H, 19-H), 2.73 (m, 1 H, 14-H‘), 2.38 – 2.24 (m, 4 H, 3-H‘, 8-H, 14-H), 2.16 (dd, $^2J_{3,3'}$ = 14.7 Hz, $^3J_{3,2}$ = 6.1 Hz, 1 H, 3-H), 1.36 (s, 9 H, 44-H), 0.86 (s, 9 H, 37-H), 0.82 (s, 9 H, 40-H), 0.03 (s, 6 H, 38-H), 0.02 (s, 3 H, 35‘-H), 0.01 (s, 3 H, 35-H).

^{13}C-NMR (125 MHz, DMSO-d$_6$): δ = 174.7 (s, C-15), 171.9 (s, C-32), 170.2 (s, C-34), 169.9 (s, C-12), 169.3 (s, C-4), 168.1 (s, C-21), 167.6 (s, C-18), 157.0 (s, C-16), 155.6 (s, C-42), 136.0 (s, C-30), 134.5 (d, C-9), 127.3 (s, C-25), 123.6 (d, C-31), 120.8 (d, C-27), 118.3 (d, C-28), 118.2 (d, C-29), 117.6 (t, C-10), 111.2 (d, C-26), 109.9 (s, C-24), 87.6 (d, C-17), 84.3 (d, C-7), 79.9 (d, C-11), 77.5 (s, C-43), 74.1 (d, C-6), 68.5 (d, C-2), 54.2 (d, C-5), 53.2 (d, C-22), 51.7 (q, C-41), 50.0 (t, C-20), 46.6 (d, C-13), 45.3 (t, C-1), 41.8 (t, C-3), 40.7 (t, C-33), 37.4 (t, C-8), 36.2 (q, C-19), 34.5 (t, C-14), 28.3 (q, C-44), 27.7 (t, C-23), 25.8 (q, C-40), 25.7 (q, C-37), 17.8 (s, C-39), 17.7 (s, C-36), –4.75 (q, C-38‘), –4.82 (q, C-35‘), –4.90 (q, C-38), –4.92 (q, C-35).

**Optical rotation:**	$[\alpha]_D^{20}$= +45.0 (c = 0.5, $CHCl_3$)	
**HRMS (ESI):**	calculated	found
$C_{52}H_{83}N_8O_{13}Si_2$ $[M+H]^+$:	1083.5613	1083.5622

Methyl *N*-((*Z*)-2-((*R*)-3-((2*R*,3*R*,4*S*,5*S*)-5-allyl-3-((*R*)-4-((*tert*-butoxycarbonyl)amino)-3-((*tert*-butyldimethylsilyl)oxy)butanamido)-4-hydroxytetrahydrofuran-2-carboxamido)-5-oxopyrrolidin-2-ylidene)acetyl)-*N*-methylglycyl-D-tryptophylglycinate 23b

0.40 M $LiOH_{aq}$ (201 µL, 80.0 µmol, 1.1 eq.) was slowly added to a 0 °C cold solution of tetrapeptide **22b** (53.0 mg, 73.0 µmol) in THF (700 µL). The resulting mixture was stirred for 2 h while slowly reaching room temperature. After full conversion, the mixture was acidified with 0.1 M HCl_{aq} and extracted with CH_2Cl_2. The combined organic layers were dried with $MgSO_4$ and concentrated in vacuo to give the crude carboxylic acid as a white foam.

4.0 M HCl in dioxane (367 µL, 1.47 mmol, 10 eq.) was added to a solution of dipeptide **SI-6** (58.0 mg, 147 µmol) in CH_2Cl_2 (50 µL) at 0 °C. After stirring for 3 h, the reaction mixture was concentrated in vacuo to give the crude amine as hydrochloride salt.

The above-prepared crude carboxylic acid and amine hydrochloride (2.0 eq.) were dissolved in anhydrous DMF (1.0 mL). After cooling to 0 °C, NMM (33.0 µL, 299 µmol, 4.1 eq.) and HATU (30.6 mg, 80.0 µmol, 1.1 eq.) were subsequently added, and the resulting mixture was stirred for 16 h while slowly reaching room temperature. The reaction mixture was diluted with EtOAc and washed with 5wt% $LiCl_{aq}$, 1.0 M HCl_{aq}, sat. $NaHCO_3$ solution and brine. The organic layer was dried over $MgSO_4$ and concentrated in vacuo. The crude product was purified by automated reversed phase column chromatography (C18 spherical, H_2O:MeCN 10% to 90% MeCN) to give hexapeptide **23b** (42.2 mg, 43.5 µmol, 60%, dr > 99:1) as a white foam.

LC-MS: t_R (23b) = 1.25 min (short method)

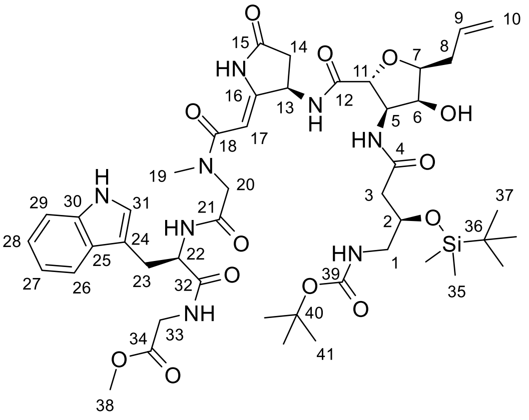

23b

^{1}H-NMR (500 MHz, DMSO-d$_6$): δ = 10.80 (s, 1 H, 31-NH), 10.40 (s, 1 H, 15-NH), 8.48 (d, $^3J_{NH,13}$ = 8.6 Hz, 1 H, 12-NH), 8.42 (t, $^3J_{NH,33}$ = 5.5 Hz, 1 H, 32-NH), 8.09 (d, $^3J_{NH,22}$ = 8.2 Hz, 1 H, 21-NH), 7.87 (d, $^3J_{NH,5}$ = 8.4 Hz, 1 H, 4-NH), 7.57 (d, $^3J_{29,28}$ = 7.9 Hz, 1 H, 29-H), 7.31 (d, $^3J_{26,27}$ = 8.0 Hz , 1 H, 26-H), 7.13 (s, 1 H, 31-H), 7.05 (ddd, $^3J_{27,26}$ = 8.2 Hz, $^3J_{27,28}$ = 7.0 Hz, $^4J_{27,29}$ = 1.2 Hz, 1 H, 27-H), 6.97 (ddd, $^3J_{28,29}$ = 7.9 Hz, $^3J_{28,27}$ = 7.0 Hz, $^4J_{28,26}$ = 1.1 Hz, 1 H, 28-H), 6.70 (m, 1 H, 39-NH), 5.81 (m, 1 H, 9-H), 5.34 (s, 1 H, 17-H), 5.30 (d, $^3J_{OH,6}$ = 5.1 Hz, 1 H, 6-OH), 5.16 – 5.06 (m, 2 H, 10-H, 13-H), 5.02 (m, 1 H, 10-H‘), 4.57 (m, 1 H, 22-H), 4.44 (ddd, $^3J_{5,11}$ = 8.7 Hz, $^3J_{5,NH}$ = 8.7 Hz, $^3J_{5,6}$ = 4.2 Hz, 1 H, 5-H),

4.07 (td, $^3J_{7,8}$ = 7.1 Hz, $^3J_{7,6}$ = 2.5 Hz, 1 H, 7-H), 4.39 (d, $^3J_{11,5}$ = 6.1 Hz, 1 H, 11-H), 4.04 – 3.88 (m, 5 H, 2-H, 6-H, 11-H, 20-H), 3.86 (d, $^3J_{33,NH}$ = 6.2 Hz, 1 H, 33-H), 3.64 (s, 3 H, 38-H), 3.17 (dd, $^2J_{23',23}$ = 14.7 Hz, $^3J_{23',22}$ = 4.9 Hz, 1 H, 23-H'), 2.98 – 2.90 (m, 3 H, 1-H, 23-H), 2.87 (s, 3 H, 19-H), 2.73 (m, 1 H, 14-H), 2.40 – 2.19 (m, 5 H, 3-H, 8-H, 14-H'), 1.37 (s, 9 H, 41-H), 0.82 (s, 9 H, 37-H), 0.03 (s, 3 H, 35-H), 0.00 (s, 3 H, 35'-H).

^{13}C-NMR (125 MHz, DMSO-d$_6$): δ = 174.8 (s, C-15), 171.9 (s, C-32), 171.4 (s, C-12), 170.2 (s, C-34), 169.8 (s, C-4), 168.2 (s, C-21), 167.7 (s, C-18), 157.0 (s, C-16), 155.6 (s, C-39), 136.0 (s, C-30), 135.0 (d, C-9), 127.3 (s, C-25), 123.6 (d, C-31), 120.8 (d, C-27), 118.3 (d, C-29), 118.2 (d, C-28), 116.9 (t, C-10), 111.2 (d, C-26), 109.9 (s, C-24), 87.9 (d, C-17), 82.2 (d, C-7), 78.9 (d, C-11), 77.5 (s, C-40), 70.8 (d, C-6), 68.9 (d, C-2), 56.5 (d, C-5), 53.2 (d, C-22), 51.7 (q, C-38), 50.2 (t, C-20), 46.4 (d, C-13), 45.4 (t, C-1), 41.8 (t, C-3), 40.7 (t, C-33), 36.3 (q, C-19), 34.7 (t, C-14), 33.5 (t, C-8), 28.3 (q, C-41), 27.7 (t, C-23), 25.8 (q, C-37), 17.8 (s, C-36), –4.9 (q, C-35'), –5.0 (q, C-35).

Selected rotamer signals:

^{1}H-NMR (500 MHz, DMSO-d$_6$): δ = 10.76 (s, 1 H, 31-NH), 10.43 (s, 1 H, 15-NH), 8.17 (m, 1 H, 32-NH/21-NH), 7.92 (m, 1 H, 32-NH/21-NH), 5.21 (s, 1 H, 17-H), 4.97 (m, 1 H, 13-H), 4.62 (m, 1 H, 22-H), 1.38 (s, 9 H, 41-H), 0.83 (s, 9 H, 37-H), 0.04 (s, 3 H, 35-H), 0.01 (s, 3 H, 35'-H).

Optical rotation: $[\alpha]_D^{20}$ = +27.6 (c = 1.0, CHCl$_3$)

**HRMS (ESI):**	calculated	found
$C_{46}H_{69}N_8O_{13}Si$ [M+H]$^+$:	969.4748	969.4733

Methyl *N*-((*Z*)-2-((*R*)-3-((2*S*,3*S*,4*S*,5*R*)-5-allyl-3-((*R*)-4-((*tert*-butoxycarbonyl)amino)-3-((*tert*-butyldimethylsilyl)oxy)butanamido)-4-((*tert*-butyldimethylsilyl)oxy)tetrahydrofuran-2-carboxamido)-5-oxopyrrolidin-2-ylidene)acetyl)-*N*-methylglycyl-D-tryptophylglycinate 23c

0.30 M LiOH$_{aq}$ (137 µL, 40.8 µmol, 1.05 eq.) was slowly added to a 0 °C cold solution of tetrapeptide **22c** (33.0 mg, 39.3 µmol) in THF (200 µL). The resulting mixture was stirred for 2 h while slowly reaching room temperature. Another portion of 0.30 M LiOH$_{aq}$ (13.0 µL, 3.93 µmol, 0.1 eq.) was added, and the stirring continued for 1 h. After full conversion, the mixture was acidified with 0.1 M HCl$_{aq}$ and extracted with CH_2Cl_2. The combined organic layers were dried with $MgSO_4$ and concentrated in vacuo to give the crude carboxylic acid as a white foam.

4.0 M HCl in dioxane (196 µL, 784 µmol, 10 eq.) was added to a solution of dipeptide **SI-6** (31.0 mg, 78.4 µmol) in CH_2Cl_2 (100 µL) at 0 °C. After stirring for 1 h, the reaction mixture was concentrated in vacuo to give the crude amine as hydrochloride salt.

The above-prepared crude carboxylic acid and amine hydrochloride (2.0 eq.) were dissolved in anhydrous DMF (500 µL). After cooling to 0 °C, NMM (17.0 µL, 159 µmol, 4.1 eq.) and HATU (15.5 mg, 40.8 µmol, 1.05 eq.) were subsequently added, and the resulting mixture was stirred for 16 h while slowly reaching room temperature. The reaction mixture was diluted with EtOAc and washed with 5wt% LiCl$_{aq}$, 1.0 M HCl$_{aq}$, sat. $NaHCO_3$ solution and brine. The organic layer was dried over $MgSO_4$ and concentrated in vacuo. The crude product was purified by automated reversed

phase column chromatography (C18 spherical, H_2O:MeCN 10% to 90% MeCN) to give hexapeptide **23c** (33.0 mg, 30.5 µmol, 78%, dr 80:20) as a white foam. The analytical characterizations were done with a diastereomerically pure fraction.

LC-MS: t_R (23c) = 4.68 min (long method)

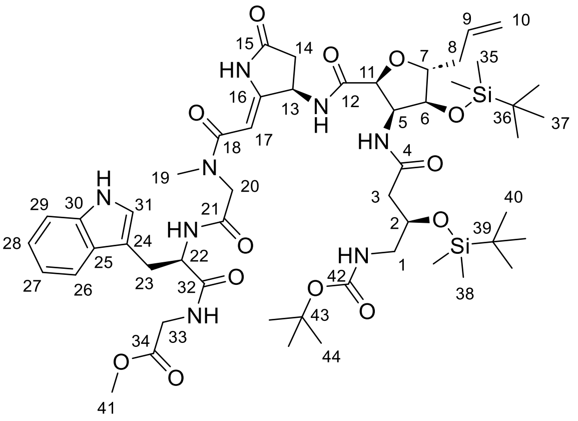

23c

^{1}H-NMR (500 MHz, DMSO-d$_6$): δ = 10.80 (s, 1 H, 31-NH), 10.40 (s, 1 H, 15-NH), 8.44 (t, $^3J_{NH,33}$ = 5.8 Hz, 1 H, 32-NH), 8.40 (d, $^3J_{NH,13}$ = 7.8 Hz, 1 H, 12-NH), 8.13 (d, $^3J_{NH,22}$ = 8.5 Hz, 1 H, 21-NH), 7.58 (d, $^3J_{29,28}$ = 8.0 Hz, 1 H, 29-H), 7.50 (d, $^3J_{NH,5}$ = 8.5 Hz, 1 H, 4-NH), 7.31 (d, $^3J_{26,27}$ = 8.0 Hz , 1 H, 26-H), 7.13 (s, 1 H, 31-H), 7.05 (ddd, $^3J_{27,26}$ = 8.1 Hz, $^3J_{27,28}$ = 6.9 Hz, $^4J_{27,29}$ = 1.2 Hz, 1 H, 27-H), 6.97 (ddd, $^3J_{28,29}$ = 7.9 Hz, $^3J_{28,27}$ = 7.0 Hz, $^4J_{28,26}$ = 1.1 Hz, 1 H, 28-H), 6.67 (t, $^3J_{NH,1}$ = 6.1 Hz, 1 H, 42-NH), 5.84 (m, 1 H, 9-H), 5.32 (s, 1 H, 17-H), 5.18 – 5.06 (m, 2 H, 10-H), 4.87 (m, 1 H, 13-H), 4.64 – 4.49 (m, 2 H, 5-H, 22-H), 4.39 (d, $^3J_{11,5}$ = 6.1 Hz, 1 H, 11-H), 4.09 – 3.87 (m, 5 H, 2-H, 6-H, 7-H, 20-H), 3.86 (m, 2 H, 33-H), 3.64 (s, 3 H, 41-H), 3.17 (dd, $^2J_{23',23}$ = 14.6 Hz, $^3J_{23',22}$ = 4.7 Hz, 1 H, 23-H‘), 3.02 – 2.86 (m, 3 H, 1-H, 23-H), 2.83 (s, 3 H, 19-H), 2.64 (m, 1 H, 14-H‘), 2.40 – 2.21 (m, 4 H, 3-H‘, 8-H, 14-H), 2.16 (m, 1 H, 3-H), 1.36 (s, 9 H, 44-H), 0.83 (s, 9 H, 37-H/40-H), 0.82 (s, 9 H, 40-H/37-H), 0.03 (s, 6 H, 35-H/38-H), 0.02 (s, 3 H, 38-H/35-H), 0.01 (s, 3 H, 38-H/35-H).

^{13}C-NMR (125 MHz, DMSO-d$_6$): δ = 174.7 (s, C-15), 171.9 (s, C-32), 170.2 (s, C-34), 169.8 (s, C-12), 169.2 (s, C-4), 168.2 (s, C-21), 167.7 (s, C-18), 156.8 (s, C-16), 155.6 (s, C-42), 136.0 (s, C-30), 134.3 (d, C-9), 127.3 (s, C-25), 123.7 (d, C-31), 120.8 (d, C-27), 118.4 (d, C-29), 118.2 (d, C-28), 117.7 (t, C-10), 111.3 (d, C-26), 110.0 (s, C-24), 87.7 (d, C-17), 82.7 (d, C-7), 77.5 (s, C-43), 77.1 (d, C-11), 74.1 (d, C-6), 68.3 (d, C-2), 53.2 (d, C-22), 53.1 (d, C-5), 51.7 (q, C-41), 49.9 (t, C-20), 47.3 (d, C-13), 45.5 (t, C-1), 41.9 (t, C-3), 40.7 (t, C-33), 37.2 (t, C-8), 36.3 (q, C-19), 34.4 (t, C-14), 28.3 (q, C-44), 27.8 (t, C-23), 25.8 (q, C-40/C-37), 25.7 (q, C-37/C-40), 17.8 (s, C-39/C-36), 17.7 (s, C-39/C-36), –4.77 (q, C-38/C-35), –4.90 (q, C-35, C-38), –5.05 (q, C-35/C-38).

Selected rotamer signals:

^{1}H-NMR (500 MHz, DMSO-d$_6$): δ = 10.44 (s, 1 H, 15-NH), 8.53 (m, 1 H, 32-NH), 8.23 (m, 1 H, 21-NH), 7.42 (m, 1 H, 4-NH), 5.22 (s, 17-H), 4.70 (m, 1 H, 13-H), 4.33 (d, $^3J_{11,5}$ = 6.1 Hz, 1 H, 11-H), 2.61 (s, 3 H, 19-H).

Optical rotation: $[\alpha]_D^{20}$= +37.5 (c = 1.0, $CHCl_3$)

**HRMS (ESI):**	calculated	found
$C_{52}H_{83}N_8O_{13}Si_2$ [M+H]$^+$:	1083.5613	1083.5616

(2*R*,3*S*,3a*S*,7*R*,14*R*,23a*R*,25a*R*,*Z*)-14-((1*H*-Indol-3-yl)methyl)-2-allyl-3,7-bis((*tert*-butyldimethyl-silyl)oxy)-18-methyl-2,3,3a,6,7,8,9,11,12,14,15,17,18,23a,24,25a-hexadecahydrofuro[2,3-q]-pyrrolo[2,3-m][1,4,7,10,15,19]hexaazacyclotricosine-5,10,13,16,19,22,25(4*H*,21*H*,23*H*)-heptaone 25a

0.20 M $LiOH_{aq}$ (359 µL, 72.0 µmol, 1.05 eq.) was slowly added to a 0 °C cold solution of hexapeptide **23a** (74.0 mg, 68.3 µmol) in THF (350 µL). The resulting mixture was stirred for 4 h while slowly reaching room temperature. After full conversion, the mixture was acidified with 0.1 M HCl_{aq} and extracted with CH_2Cl_2. The combined organic layers were dried with $MgSO_4$ and concentrated in vacuo to give the crude carboxylic acid as a white foam.

A preformed cleavage cocktail TFA:TIPS:H_2O (260 µL, 185:10:5) was added to a solution of the crude carboxylic acid in anhydrous CH_2Cl_2 (300 µL) at 0 °C. After 110 min, reaction control via LC-MS showed full conversion (60% Boc-deprotection and 40% Boc- and monoTBS-deprotection). The reaction mixture was concentrated in vacuo. The residue was dissolved in anhydrous DMF (70 mL). After cooling to 0 °C, DIPEA (119 µL, 683 µmol, 10 eq.), HOAt (105 mg, 683 µmol, 10 eq.), and PyAOP (356 mg, 683 µmol, 10 eq.) were added. The reaction mixture was stirred for 72 h while slowly reaching room temperature. After dilution with EtOAc, the mixture was washed with 5wt% $LiCl_{aq}$, 1.0 M HCl_{aq}, sat. $NaHCO_3$ solution and brine. The organic layer was dried over $MgSO_4$ and concentrated in vacuo. The crude product was purified by automated reversed phase column chromatography (C18 spherical, H_2O:MeCN 10% to 90% MeCN) to give the protected dehydrosocein precursor **25a** (25.0 mg, 26.5 µmol, 39%) as a white foam and its mono-TBS-deprotected variant **25a(OH)** (15.0 mg, 17.9 mmol, 26%) as a white foam.

Imidazole (2.6 mg, 38.0 mmol, 2.1 eq.) and TBS-Cl (3.0 mg, 19.8 mmol, 1.1 eq.) were subsequently added to a 0 °C cold solution of the mono-TBS-deprotected variant **25a(OH)** (15.0 mg, 17.9 mmol) in anhydrous DMF (180 µL). The reaction mixture was stirred for 16 h while slowly reaching room temperature. After dilution with EtOAc, the mixture was washed with 5wt% $LiCl_{aq}$, 1.0 M HCl_{aq}, sat. $NaHCO_3$ solution and brine. The organic layer was dried over $MgSO_4$ and concentrated in vacuo. The crude product was purified by automated reversed phase column chromatography (C18 spherical, H_2O:MeCN 10% to 90% MeCN) to give another portion of the protected dehydrosocein precursor **25a** (10.5 mg, 11.1 µmol, 62%) as a white foam.

LC-MS: t_R (25a) = 1.57 min (short method)

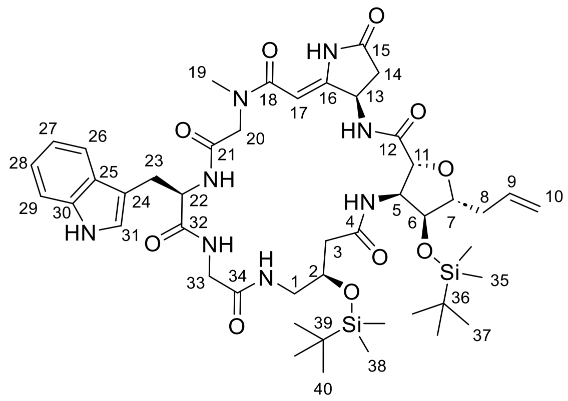

25a

^{1}H-NMR (500 MHz, DMSO-d$_6$): δ = 10.86 (m, 1 H, 31-NH), 10.36 (s, 1 H, 15-NH), 8.67 (d, $^{3}J_{NH,22}$ = 8.2 Hz, 1 H, 21-NH), 8.26 (d, $^{3}J_{NH,13}$ = 8.3 Hz, 1 H, 12-NH), 8.17 (dd, $^{3}J_{NH,33'}$ = 7.5 Hz, $^{3}J_{NH,33}$ = 5.0 Hz, 1 H, 32-NH), 7.52 (d, $^{3}J_{29,28}$ = 7.9 Hz, 1 H, 29-H), 7.35 (d, $^{3}J_{26,27}$ = 8.1 Hz, 1 H, 26-H), 7.26 – 7.20 (m, 2 H, 31-H, 34-NH), 7.07 (dd, $^{3}J_{28,29}$ = 7.6 Hz, $^{3}J_{28,27}$ = 7.6 Hz, 1 H, 28-H), 7.03 – 6.95 (m, 2 H, 27-H, 4-NH), 5.86 (ddt, $^{3}J_{9,10}$ = 17.2 Hz, $^{3}J_{9,10'}$ = 10.2 Hz, $^{3}J_{9,8}$ = 7.0 Hz, 1 H, 9-H), 5.24 (s, 1 H, 17-H), 5.19 (m, 1 H, 13-H), 5.13 (dd, $^{2}J_{10',10}$ = 2.0 Hz, $^{3}J_{10',9}$ = 17.3 Hz, 1 H, 10-H‘), 5.07 (dd, $^{2}J_{10,10'}$ = 2.1 Hz, $^{3}J_{10,9}$ = 10.2 Hz, 1 H, 10-H), 4.50 (d, $^{2}J_{20',20}$ = 15.6 Hz, 1 H, 20-H‘), 4.37 (ddd, $^{3}J_{5,11}$ = 9.2 Hz, $^{3}J_{5,NH}$ = 9.2 Hz, $^{3}J_{5,6}$ = 5.2 Hz, 1 H, 5-H), 4.25 (ddd, $^{3}J_{22,23}$ = 9.9 Hz, $^{3}J_{22,NH}$ = 5.0 Hz, $^{3}J_{22,23'}$ = 5.0 Hz, 1 H, 22-H), 4.18 (m, 1 H, 2-H), 3.99 (d, $^{3}J_{11,5}$ = 9.2 Hz, 1 H, 11-H), 3.90 (dd, $^{3}J_{6,5}$ = 5.3 Hz, $^{3}J_{6,7}$ = 1.9 Hz, 1 H, 6-H), 3.85 (td, $^{3}J_{7,8}$ = 6.6 Hz, $^{3}J_{7,6}$ = 1.9 Hz, 1 H, 7-H), 3.77 (dd, $^{2}J_{33',33}$ = 16.9 Hz, $^{3}J_{33',NH}$ = 7.6 Hz, 1 H, 33-H‘), 3.46 (d, $^{2}J_{20,20'}$ = 16.0 Hz, 1 H, 20-H), 3.42 (m, 1 H, 33-H), 3.21 (dd, $^{2}J_{23',23}$ = 14.7 Hz, $^{3}J_{23',22}$ = 4.3 Hz, 1 H, 23-H‘), 3.16 (s, 3 H, 19-H), 3.14 (m, 1 H, 1-H‘), 3.00 (dd, $^{2}J_{23,23'}$ = 14.8 Hz, $^{3}J_{23,22}$ = 9.8 Hz, 1 H, 23-H), 2.82 – 2.73 (m, 2 H, 1-H, 14-H‘), 2.37 – 2.23 (m, 3 H, 8-H, 14-H), 2.13 (m, 2 H, 3-H), 0.89 (s, 9 H, 40-H), 0.78 (s, 9 H, 37-H), 0.04 (s, 6 H, 35-H), 0.02 (s, 3 H, 38-H), 0.02 (s, 3 H, 38‘-H).

^{13}C-NMR (125 MHz, DMSO-d$_6$): δ = 174.2 (s, C-15), 171.8 (s, C-21), 171.5 (s, C-32), 169.5 (s, C-12), 169.1 (s, C-4), 168.5 (s, C-34), 168.4 (s, C-18), 156.4 (s, C-16), 136.2 (s, C-30), 134.4 (d, C-9), 126.9 (s, C-25), 123.9 (d, C-31), 121.0 (d, C-28), 118.3 (d, C-27), 118.1 (d, C-29), 117.6 (t, C-10), 111.4 (d, C-26), 109.8 (s, C-24), 87.9 (d, C-17), 85.9 (d, C-7), 79.0 (d, C-11), 74.6 (d, C-6), 67.6 (d, C-2), 55.6 (d, C-22), 54.1 (d, C-5), 51.5 (t, C-20), 46.6 (d, C-13), 44.5 (t, C-1), 42.8 (t, C-3), 42.5 (t, C-33), 37.7 (t, C-8), 37.5 (q, C-19), 34.2 (t, C-14), 26.4 (t, C-23), 25.7 (q, C-40), 25.7 (q, C-37), 17.8 (s, C-39), 17.6 (s, C-36), –4.7 (q, C-35‘), –4.8 (q, C-38‘), –4.85 (q, C-35), –4.93 (q, C-38).

**Optical rotation:**	$[\alpha]_D^{20}$= –21.4 (c = 1.0, CHCl$_3$)	
**HRMS (ESI):**	calculated	found
$C_{46}H_{71}N_8O_{10}Si_2$ [M+H]$^+$:	951.4826	951.4836

(2*R*,3*S*,3a*S*,7*R*,14*R*,23a*R*,25a*S*,*Z*)-14-((1*H*-Indol-3-yl)methyl)-2-allyl-3,7-bis((*tert*-butyldimethyl-silyl)oxy)-18-methyl-2,3,3a,6,7,8,9,11,12,14,15,17,18,23a,24,25a-hexadecahydrofuro[2,3-q]-pyrrolo[2,3-m][1,4,7,10,15,19]hexaazacyclotricosine-5,10,13,16,19,22,25(4*H*,21*H*,23*H*)-heptaone 25c

0.30 M $LiOH_{aq}$ (92.0 µL, 28.0 µmol, 1.0 eq.) was slowly added to a 0 °C cold solution of hexapeptide **23c** (30.0 mg, 27.7 µmol) in THF (200 µL). The resulting mixture was stirred for 6 h while slowly reaching room temperature. After full conversion, the mixture was acidified with 0.1 M HCl_{aq} and extracted with CH_2Cl_2. The combined organic layers were dried with $MgSO_4$ and concentrated in vacuo to give the crude carboxylic acid as a white foam.

A preformed cleavage cocktail TFA:TIPS:H_2O (107 µL, 185:10:5) was added to a solution of the crude carboxylic acid in anhydrous CH_2Cl_2 (30 µL) at –10 °C. After 15 min, reaction control via LC-MS showed full conversion (70% Boc-deprotection and 30% Boc- and monoTBS-deprotection). The reaction mixture was concentrated in vacuo. The residue was dissolved in anhydrous DMF (25 mL). After cooling to 0 °C, DIPEA (48.0 µL, 277 µmol, 10 eq.), HOAt (42.7 mg, 277 µmol, 10 eq.), and PyAOP (144 mg, 277 µmol, 10 eq.) were added. The reaction mixture was stirred for 72 h while slowly reaching room temperature. After dilution with EtOAc, the mixture was washed with 5wt% $LiCl_{aq}$, 1.0 M HCl_{aq}, sat. $NaHCO_3$ solution and brine. The organic layer was dried over $MgSO_4$ and concentrated in vacuo. The crude product was purified by automated reversed phase column chromatography (C18 spherical, H_2O:MeCN 10% to 90% MeCN) followed by preparative HPLC (H_2O:MeCN 10% to 100% MeCN) to give the protected dehydrosocein precursor **25c** (8.7 mg, 9.15 µmol, 33%, dr > 99:1) as a white foam and its mono-TBS-deprotected variant **25c(OH)** (3.9 mg, 4.66 µmol, 17%) as a white foam. The epimer from the tetrapeptide coupling was separated during the preparative HPLC (hence the improved diastereomeric ratio).

LC-MS: t_R (25c) = 1.52 min (short method)

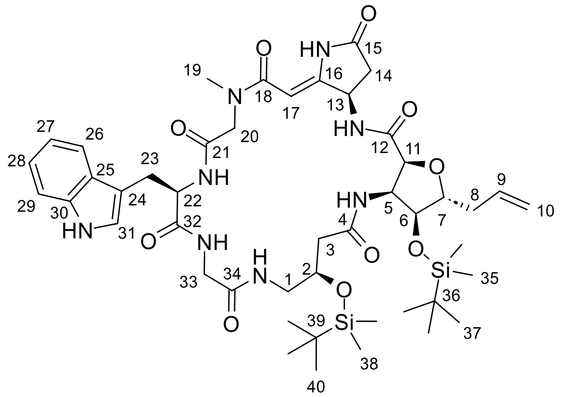

25c

^{1}H-NMR (500 MHz, $CDCl_3$): δ = 9.95 (s, 1 H, 15-NH), 8.61 (m, 1 H, 31-NH), 7.73 (dd, $^3J_{NH,33}$ = 8.2 Hz, $^3J_{NH,33'}$ = 3.5 Hz , 1 H, 32-NH), 7.64 (d, $^3J_{29,28}$ = 7.9 Hz, 1 H, 29-H), 7.41 (d, $^3J_{26,27}$ = 8.1 Hz, 1 H, 26-H), 7.24 (ddd, $^3J_{27,26}$ = 8.1 Hz, $^3J_{27,28}$ = 7.0 Hz, $^4J_{27,29}$ = 1.2 Hz, 1 H, 27-H), 7.19 – 7.13 (m, 2 H, 28-H, 34-NH), 7.12 (d, $^3J_{31,NH}$ = 2.4 Hz, 1 H, 31-H), 7.01 (m, 1 H, 12-NH), 6.71 (m, 1 H, 21-NH), 6.36 (m, 1 H, 4-NH), 5.83 (m, 1 H, 9-H), 5.70 (d, $^4J_{17,13}$ = 1.5 Hz, 1 H, 17-H), 5.22 – 5.15 (m, 2 H, 10-H), 5.04 (m, 1 H, 13-H), 4.65 (m, 1 H, 22-H), 4.60 (ddd, $^3J_{5,6}$ = 5.9 Hz, $^3J_{5,NH}$ = 5.9 Hz, $^3J_{5,11}$ = 3.5 Hz, 1 H, 5-H), 4.45 (dd, $^2J_{33,33'}$ = 17.1 Hz, $^3J_{33,NH}$ = 8.4 Hz, 1 H, 33-H), 4.37 (d, $^3J_{11,5}$ = 3.4 Hz, 1 H, 11-H), 4.23 (dd, $^3J_{6,7}$ = 6.9 Hz, $^3J_{6,5}$ = 5.5 Hz, 1 H, 6-H), 4.08 (m, 1 H, 2-H), 4.05 – 3.98 (m, 2 H, 7-H, 20-H), 3.45 – 3.33

(m, 3 H, 1-H, 23-H), 3.35 (s, 3 H, 19-H), 3.27 (d, $^2J_{20',20}$ = 14.2 Hz, 1 H, 20-H'), 3.23 (dd, $^2J_{33',33}$ = 17.2 Hz, $^3J_{33',NH}$ = 3.6 Hz, 1 H, 33-H'), 3.05 (ddd, $^2J_{1',1}$ = 13.4 Hz, $^3J_{1',NH}$ = 4.5 Hz, $^3J_{1',2}$ = 4.5 Hz, 1 H, 1-H'), 2.63 (dd, $^2J_{14,14'}$ = 17.6 Hz, $^3J_{14,13}$ = 9.9 Hz, 1 H, 14-H'), 2.53 (m, 1 H, 8-H), 2.35 – 2.23 (m, 3 H, 3-H, 8-H', 14-H'), 2.19 (dd, $^2J_{3',3}$ = 15.2 Hz, $^3J_{3,2}$ = 4.3 Hz, 2 H, 3-H'), 0.91 (s, 9 H, 40-H), 0.85 (s, 9 H, 37-H), 0.13 (s, 3 H, 38-H/35-H), 0.10 (s, 3 H, 38-H/35-H), 0.08 (s, 3 H, 35-H/38-H), 0.04 (s, 3 H, 35-H/38-H).

^{13}C-NMR (125 MHz, $CDCl_3$): δ = 173.9 (s, C-15), 172.3 (s, C-21), 171.7 (s, C-32), 171.1 (s, C-4), 170.1 (s, s, C-12, C-34), 170.0 (s, C-18), 155.6 (s, C-16), 136.4 (s, C-30), 133.1 (d, C-9), 127.7 (s, C-25), 123.4 (d, C-31), 122.6 (d, C-27), 120.1 (d, C-28), 118.9 (t, C-10), 118.6 (d, C-29), 111.7 (d, C-26), 109.6 (s, C-24), 90.9 (d, C-17), 82.6 (d, C-7), 81.1 (d, C-11), 74.9 (d, C-6), 68.3 (d, C-2), 55.7 (d, C-22), 54.8 (t, C-20), 54.7 (d, C-5), 47.6 (d, C-13), 45.0 (t, C-1), 42.9 (t, C-33), 41.1 (t, C-3), 39.4 (q, C-19), 37.2 (t, C-8), 34.3 (t, C-14), 26.1 (q, t, C-40, C-23), 26.0 (q, C-37), 18.1 (s, s, C-36, C-39), –4.1 (q, C-38), –4.2 (q, C-38'), –4.9 (q, C-35), –5.0 (q, C-35').

Optical rotation: $[\alpha]_D^{20}$= +7.4 (c = 1.0, $CHCl_3$)

**HRMS (ESI):**	calculated	found
$C_{46}H_{71}N_8O_{10}Si_2$ $[M+H]^+$:	951.4826	951.4832

(2*R*,3*S*,3a*R*,7*R*,14*R*,23a*R*,25a*R*,*Z*)-14-((1*H*-Indol-3-yl)methyl)-2-allyl-3,7-dihydroxy-18-methyl-2,3,3a,6,7,8,9,11,12,14,15,17,18,23a,24,25a-hexadecahydrofuro[2,3-q]pyrrolo[2,3-m][1,4,7,10,15,19]hexaazacyclotricosine-5,10,13,16,19,22,25(4*H*,21*H*,23*H*)-heptaone 27a

1.0 M TBAF in THF (9.05 µL, 9.05 µmol, 2.05 eq.) was added to a 0 °C cold solution of protected dehydrosocein precursor **25a** (4.2 mg, 4.4 µmol) in anhydrous THF (100 µL). The resulting solution was stirred for 4 h while slowly reaching room temperature. After the addition of a droplet of H_2O, the reaction mixture was adsorbed on isolute® and purified by automated reversed phase column chromatography (C18 spherical, H_2O:MeCN 10% to 90% MeCN) followed by preparative HPLC (H_2O:MeCN 10% to 85% MeCN) to give dehydrosocein precursor **27a** (2.3 mg, 3.2 µmol, 72%) as an amorphous solid after lyophilization.

LC-MS: t_R (27a) = 0.71 min (short method)

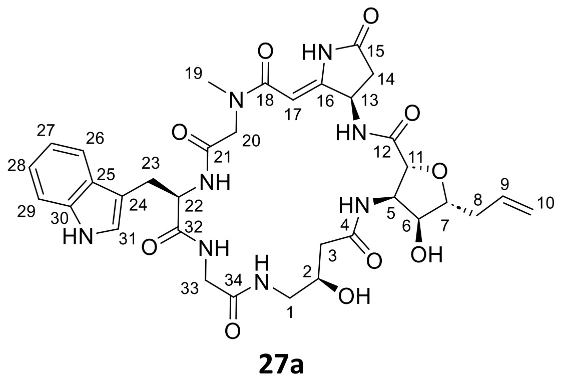

27a

^{1}H-NMR (500 MHz, DMSO-d$_6$): δ = 10.86 (d, $^{3}J_{NH,31}$ = 2.4 Hz, 1 H, 31-NH), 10.39 (s, 1 H, 15-NH), 8.57 (d, $^{3}J_{NH,22}$ = 5.7 Hz, 1 H, 21-NH), 8.46 (d, $^{3}J_{NH,13}$ = 8.3 Hz, 1 H, 12-NH), 8.29 (t, $^{3}J_{NH,33}$ = 6.2 Hz, 1 H, 32-NH), 7.60 (d, $^{3}J_{NH,5}$ = 8.4 Hz, 1 H, 4-NH), 7.52 (d, $^{3}J_{29,28}$ = 7.9 Hz, 1 H, 29-H), 7.34 (d, $^{3}J_{26,27}$ = 8.0 Hz, 1 H, 26-H), 7.26 (t, $^{3}J_{NH,1}$ = 5.2 Hz, 1 H, 34-NH), 7.21 (d, $^{3}J_{31,NH}$ = 2.3 Hz, 1 H, 31-H), 7.07 (ddd, $^{3}J_{28,29}$ = 8.1 Hz, $^{3}J_{28,27}$ = 7.0 Hz, $^{4}J_{28,26}$ = 1.2 Hz, 1 H, 28-H), 6.99 (ddd, $^{3}J_{27,26}$ = 7.9 Hz, $^{3}J_{27,28}$ = 6.9 Hz, $^{4}J_{27,29}$ = 1.0 Hz, 1 H, 27-H), 5.85 (ddt, $^{3}J_{9,10}$ = 17.1 Hz, $^{3}J_{9,10'}$ = 10.3 Hz, $^{3}J_{9,8}$ = 6.8 Hz, 1 H, 9-H), 5.48 (d, $^{3}J_{OH,6}$ = 4.2 Hz, 1 H, 6-OH), 5.38 (d, $^{4}J_{17,13}$ = 1.5 Hz, 1 H, 17-H), 5.18 (m, 1 H, 13-H), 5.12 (ddt, $^{3}J_{10',9}$ = 17.3 Hz, $^{2}J_{10',10}$ = 1.7 Hz, $^{4}J_{10',8}$ = 1.7 Hz, 1 H, 10-H‘), 5.04 (ddt, $^{3}J_{10,9}$ = 10.2 Hz, $^{2}J_{10,10'}$ = 2.2 Hz, $^{4}J_{10,8}$ = 1.2 Hz, 1 H, 10-H), 4.67 (d, $^{3}J_{OH,2}$ = 4.9 Hz, 1 H, 2-OH), 4.56 (d, $^{3}J_{20',20}$ = 15.8 Hz, 1 H, 20-H‘), 4.39 (ddd, $^{3}J_{5,11}$ = 8.6 Hz, $^{3}J_{5,NH}$ = 8.6 Hz, $^{3}J_{5,6}$ = 5.5 Hz, 1 H, 5-H), 4.18 (ddd, $^{3}J_{22,23}$ = 10.1 Hz, $^{3}J_{22,NH}$ = 5.2 Hz, $^{3}J_{22,23'}$ = 5.2 Hz, 1 H, 22-H), 3.97 (m, 1 H, 2-H), 3.89 (d, $^{3}J_{11,5}$ = 8.8 Hz, 1 H, 11-H), 3.86 – 3.77 (m, 2 H, 6-H, 7-H), 3.61 (m, 2 H, 33-H), 3.44 (d, $^{2}J_{20,20'}$ = 15.8 Hz, 1 H, 20-H), 3.19 (dd, $^{2}J_{23',23}$ = 14.7 Hz, $^{3}J_{23',22}$ = 4.6 Hz, 1 H, 23-H‘), 3.16 – 3.09 (m, 4 H, 1-H, 19-H), 3.01 (dd, $^{2}J_{23,23'}$ = 14.7 Hz, $^{3}J_{23,22}$ = 9.4 Hz, 1 H, 23-H), 2.80 (m, 1 H, 1-H‘), 2.75 (dd, $^{2}J_{14,14'}$ = 17.5 Hz, $^{3}J_{14,13}$ = 9.6 Hz, 1 H, 14-H), 2.35 – 2.20 (m, 5 H, 3-H, 8-H, 14-H‘).

^{13}C-NMR (125 MHz, DMSO-d$_6$): δ = 174.4 (s, C-15), 171.5 (s, C-32), 171.0 (s, C-21), 170.3 (s, C-4), 169.9 (s, C-12), 168.8 (s, C-34), 168.2 (s, C-18), 156.4 (s, C-16), 136.1 (s, C-30), 134.9 (d, C-9), 127.0 (s, C-25), 124.0 (d, C-31), 121.0 (d, C-28), 118.4 (d, C-27), 118.1 (d, C-29), 117.1 (t, C-10), 111.4 (d, C-26), 109.8 (s, C-24), 88.2 (d, C-17), 85.8 (d, C-7), 80.3 (d, C-11), 72.9 (d, C-6), 66.0 (d, C-2), 55.5 (d, C-22), 54.3 (d, C-5), 50.7 (t, C-20), 46.4 (d, C-13), 45.0 (t, C-1), 42.5 (t, C-33), 41.5 (t, C-3), 37.8 (t, C-8), 37.1 (q, C-19), 34.2 (t, C-14), 26.2 (t, C-23).

Optical rotation: $[\alpha]_D^{20}$= –39.3 (c = 0.3, MeOH)

**HRMS (ESI):**	calculated	found
$C_{34}H_{43}N_8O_{10}$ [M+H]$^+$:	723.3097	723.3097

(2*S*,3*S*,3a*R*,7*R*,14*R*,23a*R*,25a*R*,*Z*)-14-((1*H*-Indol-3-yl)methyl)-2-allyl-3,7-dihydroxy-18-methyl-2,3,3a,6,7,8,9,11,12,14,15,17,18,23a,24,25a-hexadecahydrofuro[2,3-q]pyrrolo[2,3-m][1,4,7,10,15,19]hexaazacyclotricosine-5,10,13,16,19,22,25(4*H*,21*H*,23*H*)-heptaone 27b

0.40 M LiOH$_{aq}$ (95.0 µL, 38.0 µmol, 1.05 eq.) was slowly added to a 0 °C cold solution of hexapeptide **23b** (35.0 mg, 36.0 µmol) in THF (250 µL). The resulting mixture was stirred for 5 h while slowly reaching room temperature. After full conversion, the mixture was acidified with 0.1 M HCl$_{aq}$ and extracted with CH_2Cl_2 and $CHCl_3$. The combined organic layers were dried with $MgSO_4$ and concentrated in vacuo to give the crude carboxylic acid as a white foam.

A preformed cleavage cocktail TFA:TIPS:H_2O (1.0 mL, 185:10:5) was added to a solution of the crude carboxylic acid in anhydrous CH_2Cl_2 (1.0 mL) at 0 °C. After 30 min, reaction control via LC-MS showed full conversion. The reaction mixture was concentrated in vacuo. The residue was dissolved in anhydrous DMF (18 mL). After cooling to 0 °C, DIPEA (63.0 µL, 361 µmol, 10 eq.), HOAt (55.7 mg, 361 µmol, 10 eq.), and PyAOP (188 mg, 361 µmol, 10 eq.) were added. The reaction

mixture was stirred for 16 h while slowly reaching room temperature. After dilution with EtOAc, the mixture was washed with 5wt% $LiCl_{aq}$, 1.0 M HCl_{aq}, sat. $NaHCO_3$ solution and brine. The organic layer was dried over $MgSO_4$ and concentrated in vacuo. The crude product was purified by automated reversed phase column chromatography (C18 spherical, H_2O:MeCN 10% to 90% MeCN) followed by preparative HPLC (H_2O:MeCN 20% to 90% MeCN) and preparative HPLC (0.1% $HCOOH_{aq}$:MeCN 20% to 90% MeCN) to give the cyclized dehydrosocein precursor (7.0 mg, unknown purity).

1.0 M TBAF in THF (10.0 µL, 10.0 µmol) was added to a 0 °C cold solution of cyclized dehydrosocein precursor (7.0 mg, unknown purity) in anhydrous THF (80 µL). The resulting solution was stirred for 4 h while slowly reaching room temperature. After the addition of a droplet of H_2O, the reaction mixture was adsorbed on isolute® and purified by automated reversed phase column chromatography (C18 spherical, 0.1% $HCOOH_{aq}$:MeCN 0% to 50% MeCN) followed by preparative HPLC (0.1% $HCOOH_{aq}$:MeCN 0% to 70% MeCN) to give dehydrosocein precursor **27b** (4.1 mg, 5.7 µmol, 16% from **23b**) as an amorphous solid after lyophilization.

LC-MS: t_R (27b) = 1.16 min (long method)

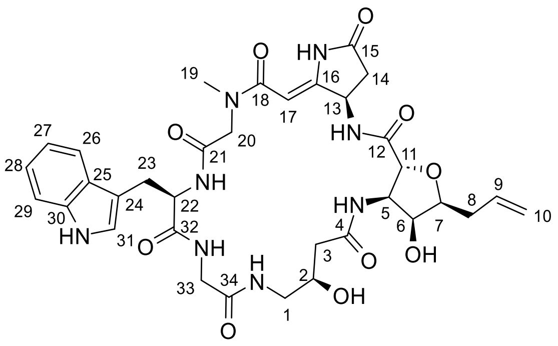

27b

^{1}H-NMR (500 MHz, DMSO-d$_6$): δ = 10.87 (d, $^{3}J_{NH,31}$ = 2.4 Hz, 1 H, 31-NH), 10.39 (s, 1 H, 15-NH), 8.54 – 8.46 (m, 2 H, 21-NH, 12-H), 8.31 (t, $^{3}J_{NH,33}$ = 6.2 Hz, 1 H, 32-NH), 7.73 (d, $^{3}J_{NH,5}$ = 8.4 Hz, 1 H, 4-NH), 7.52 (d, $^{3}J_{29,28}$ = 7.9 Hz, 1 H, 29-H), 7.34 (d, $^{3}J_{26,27}$ = 8.0 Hz, 1 H, 26-H), 7.28 (t, $^{3}J_{NH,1}$ = 5.5 Hz, 1 H, 34-NH), 7.21 (d, $^{3}J_{31,NH}$ = 2.3 Hz, 1 H, 31-H), 7.07 (ddd, $^{3}J_{28,29}$ = 8.1 Hz, $^{3}J_{28,27}$ = 6.8 Hz, $^{4}J_{28,26}$ = 1.2 Hz, 1 H, 28-H), 6.99 (ddd, $^{3}J_{27,26}$ = 7.9 Hz, $^{3}J_{27,28}$ = 7.0 Hz, $^{4}J_{27,29}$ = 1.1 Hz, 1 H, 27-H), 5.80 (ddt, $^{3}J_{9,10}$ = 17.0 Hz, $^{3}J_{9,10'}$ = 10.1 Hz, $^{3}J_{9,8}$ = 6.8 Hz, 1 H, 9-H), 5.41 (m, 1 H, 6-OH), 5.38 (d, $^{4}J_{17,13}$ = 1.6 Hz, 1 H, 17-H), 5.16 (m, 1 H, 13-H), 5.11 (m, 1 H, 10-H‘), 5.01 (m, 1 H, 10-H), 4.67 (d, $^{3}J_{OH,2}$ = 4.9 Hz, 1 H, 2-OH), 4.57 (d, $^{3}J_{20',20}$ = 15.9 Hz, 1 H, 20-H‘), 4.53 (ddd, $^{3}J_{5,11}$ = 8.6 Hz, $^{3}J_{5,NH}$ = 8.6 Hz, $^{3}J_{5,6}$ = 4.4 Hz, 1 H, 5-H), 4.19 (ddd, $^{3}J_{22,23}$ = 10.0 Hz, $^{3}J_{22,NH}$ = 5.2 Hz, $^{3}J_{22,23'}$ = 5.2 Hz, 1 H, 22-H), 4.08 (td, $^{3}J_{7,8}$ = 6.9 Hz, $^{3}J_{7,6}$ = 2.6 Hz, 1 H, 7-H), 4.01 – 3.93 (m, 2 H, 2-H, 6-H), 3.91 (d, $^{3}J_{11,5}$ = 8.6 Hz, 1 H, 11-H), 3.65 (dd, $^{2}J_{33,33'}$ = 16.9 Hz, $^{3}J_{33,NH}$ = 5.9 Hz, 1 H, 33-H), 3.58 (dd, $^{2}J_{33',33}$ = 16.9 Hz, $^{3}J_{33',NH}$ = 6.2 Hz, 1 H, 33-H‘), 3.43 (d, $^{2}J_{20,20'}$ = 15.9 Hz, 1 H, 20-H), 3.18 (dd, $^{2}J_{23',23}$ = 14.7 Hz, $^{3}J_{23',22}$ = 4.8 Hz, 1 H, 23-H‘), 3.15 – 3.09 (m, 4 H, 1-H, 19-H), 3.01 (dd, $^{2}J_{23,23'}$ = 14.8 Hz, $^{3}J_{23,22}$ = 9.5 Hz, 1 H, 23-H), 2.79 (m, 1 H, 1-H‘), 2.74 (dd, $^{2}J_{14,14'}$ = 17.6 Hz, $^{3}J_{14,13}$ = 9.7 Hz, 1 H, 14-H), 2.38 – 2.17 (m, 5 H, 3-H, 8-H, 14-H‘).

^{13}C-NMR (125 MHz, DMSO-d$_6$): δ = 174.4 (s, C-15), 171.5 (s, C-32), 171.4 (s, C-12), 170.9 (s, C-21), 170.3 (s, C-4), 168.8 (s, C-34), 168.2 (s, C-18), 156.5 (s, C-16), 136.1 (s, C-30), 135.2 (d, C-9), 127.0 (s, C-25), 124.0 (d, C-31), 121.0 (d, C-28), 118.4 (d, C-27), 118.1 (d, C-29), 116.8 (t, C-10), 111.4 (d, C-26), 109.8 (s, C-24), 88.1 (d, C-17), 82.6 (d, C-7), 79.3 (d, C-11), 71.0 (d, C-6), 66.0 (d, C-2), 56.5 (d, C-5), 55.4 (d, C-22), 50.6 (t, C-20), 46.3 (d, C-13), 45.0 (t, C-1), 42.5 (t, C-33), 41.4 (t, C-3), 37.1 (q, C-19), 34.3 (t, C-14), 33.6 (t, C-8), 26.3 (t, C-23).
Selected rotamer signals:
^{1}H-NMR (500 MHz, DMSO-d$_6$): δ = 10.84 (m, 1 H, 31-NH), 10.43 (s, 1 H, 15-NH), 7.56 (m, 1 H, 29-H), 7.14 (m, 1 H, 31-H), 4.89 (m, 1 H, 13-H), 4.42 (m, 1 H, 5-H), 2.63 (s, 3 H, 19-H).

**Optical rotation:**	$[\alpha]_D^{20}$= -15.4 (c = 0.5, DMSO)	
**HRMS (ESI):**	calculated	found
$C_{34}H_{43}N_8O_{10}$ [M+H]$^+$:	723.3097	723.3100

(2*R*,3*S*,3a*R*,7*R*,14*R*,23a*R*,25a*S*,*Z*)-14-((1*H*-Indol-3-yl)methyl)-2-allyl-3,7-dihydroxy-18-methyl-2,3,3a,6,7,8,9,11,12,14,15,17,18,23a,24,25a-hexadecahydrofuro[2,3-q]pyrrolo[2,3-m][1,4,7,10,15,19]hexaazacyclotricosine-5,10,13,16,19,22,25(4*H*,21*H*,23*H*)-heptaone 27c

1.0 M TBAF in THF (4.47 µL, 4.47 µmol, 1.1 eq.) was added to a 0 °C cold solution of mono-TBS-deprotected variant **25c(OH)** (3.4 mg, 4.06 µmol) in anhydrous THF (300 µL). The resulting solution was stirred for 3 h while slowly reaching room temperature. After the addition of a droplet of H_2O, the reaction mixture was adsorbed on isolute® and purified by automated reversed phase column chromatography (C18 spherical, H_2O:MeCN 10% to 90% MeCN) to give dehydrosocein precursor **27c** (2.4 mg, 3.32 µmol, 82%) as an amorphous solid after lyophilization.

LC-MS: t$_R$ (27c) = 0.70 min (short method)

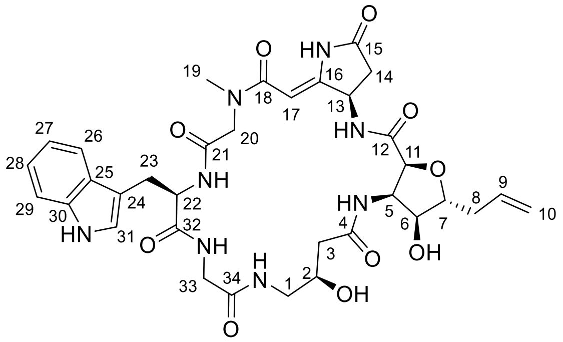

27c

^{1}H-NMR (500 MHz, MeOD-d$_4$): δ = 7.69 (d, $^3J_{29,28}$ = 7.9 Hz, 1 H, 29-H), 7.27 (d, $^3J_{26,27}$ = 8.0 Hz, 1 H, 26-H), 7.16 (s, 1 H, 31-H), 7.11 (ddd, $^3J_{28,29}$ = 8.0 Hz, $^3J_{28,27}$ = 7.0 Hz, $^4J_{28,26}$ = 1.2 Hz, 1 H, 28-H), 7.05 (ddd, $^3J_{27,26}$ = 8.0 Hz, $^3J_{27,28}$ = 7.0 Hz, $^4J_{27,29}$ = 1.1 Hz, 1 H, 27-H), 5.81 (ddt, $^3J_{9,10}$ = 17.2 Hz, $^3J_{9,10'}$ = 10.3 Hz, $^3J_{9,8}$ = 6.9 Hz, 1 H, 9-H), 5.12 (m, 1 H, 10-H'), 5.07 (m, 1 H, 10-H), 4.79 (d, $^4J_{17,13}$ = 1.6 Hz, 1 H, 17-H), 4.62 (dd, $^3J_{5,11}$ = 8.5 Hz, $^3J_{5,6}$ = 4.5 Hz, 1 H, 5-H), 4.49 (dd, $^3J_{22,23}$ = 10.7 Hz, $^3J_{22,23'}$ = 4.7 Hz, 1 H, 22-H), 4.30 (d, $^3J_{11,5}$ = 8.5 Hz, 1 H, 11-H), 4.25 (d, $^2J_{20',20}$ = 17.9 Hz, 1 H, 20-H'), 4.24 (m, 1 H, 13-H), 4.17 (m, 1 H, 2-H), 4.11 (t, $^3J_{7,8}$ = 6.8 Hz, 1 H, 7-H), 4.02 (d, $^2J_{33',33}$ = 16.6 Hz, 1 H, 33-H'), 3.98

(d, $^3J_{6,5}$ = 4.5 Hz, 1 H, 6-H), 3.77 (d, $^2J_{33,33'}$ = 16.3 Hz, 1 H, 33-H), 3.73 (d, $^2J_{20,20'}$ = 17.6 Hz, 1 H, 20-H), 3.47 (m, 1 H, 1-H‘), 3.38 (dd, $^2J_{23',23}$ = 14.5 Hz, $^3J_{23',22}$ = 4.6 Hz, 1 H, 23-H‘), 3.19 (m, 1 H, 1-H), 3.03 (dd, $^2J_{23,23'}$ = 14.5 Hz, $^3J_{23,22}$ = 10.9 Hz, 1 H, 23-H), 2.95 (s, 3 H, 19-H), 2.70 (dd, $^2J_{3,3'}$ = 14.5 Hz, $^3J_{3,2}$ = 10.1 Hz, 1 H, 3-H), 2.53 – 2.18 (m, 5 H, 3-H‘, 8-H, 14-H).

^{13}C-NMR (125 MHz, MeOD-d$_4$): δ = 177.2 (s, C-15), 174.8 (s, C-32), 174.0 (s, C-12), 173.5 (s, C-4), 173.1 (s, C-21), 172.4 (s, C-34), 170.1 (s, C-18), 157.3 (s, C-16), 138.5 (s, C-30), 135.0 (d, C-9), 128.1 (s, C-25), 125.2 (d, C-31), 122.6 (d, C-28), 120.1 (d, C-27), 119.6 (d, C-29), 118.2 (t, C-10), 112.6 (d, C-26), 110.0 (s, C-24), 90.1 (d, C-17), 87.6 (d, C-7), 75.6 (d, C-11), 73.5 (d, C-6), 68.7 (d, C-2), 56.3 (d, C-22), 55.0 (d, C-5), 54.4 (t, C-20), 46.8 (d, C-13), 46.4 (t, C-1), 44.2 (t, C-33), 41.1 (t, C-3), 39.6 (t, C-8), 35.6 (q, C-19), 35.2 (t, C-14), 28.3 (t, C-23).

Selected rotamer signals:

^{1}H-NMR (500 MHz, MeOD-d$_4$): δ = 7.58 (d, $^3J_{29,28}$ = 7.9 Hz, 1 H, 29-H), 7.34 (d, $^3J_{26,27}$ = 8.2 Hz , 1 H, 26-H), 7.18 (s, 1 H, 31-H), 7.09 (m, 1 H, 28-H), 7.03 (m, 1 H, 27-H), 5.89 (ddt, $^3J_{9,10}$ = 17.2 Hz, $^3J_{9,10'}$ = 10.1 Hz, $^3J_{9,8}$ = 7.0 Hz, 1 H, 9-H), 5.67 (d, $^4J_{17,13}$ = 1.5 Hz, 1 H, 17-H), 5.27 (ddd, $^3J_{13,14}$ = 9.7 Hz, $^3J_{13,14'}$ = 5.4 Hz, $^4J_{13,17}$ = 1.5 Hz, 1 H, 13-H), 5.16 (m, 1 H, 10-H‘), 4.70 (m, 1 H, 5-H), 4.41 (d, $^2J_{20',20}$ = 15.7 Hz, 1 H, 20-H‘), 4.34 (dd, $^3J_{22,23}$ = 8.7 Hz, $^3J_{22,23'}$ = 5.7 Hz, 1 H, 22-H), 3.68 (d, $^2J_{20,20'}$ = 16.0 Hz, 1 H, 20-H), 3.12 (s, 3 H, 19-H), 2.86 (dd, $^2J_{14,14'}$ = 17.9 Hz, $^3J_{14,13}$ = 9.9 Hz, 1 H, 14-H), 2.58 – 2.14 (m, 5 H, 3-H, 8-H, 14-H‘).

**Optical rotation:**	$[\alpha]_D^{20}$= +38.0 (c = 0.3, MeOH)	
**HRMS (ESI):**	calculated	found
$C_{34}H_{43}N_8O_{10}$ [M+H]$^+$:	723.3097	723.3103

(2*R*,3*S*,3a*R*,7*R*,14*R*,23a*R*,25a*R*,*Z*)-14-((1*H*-Indol-3-yl)methyl)-2-cinnamyl-3,7-dihydroxy-18-methyl-2,3,3a,6,7,8,9,11,12,14,15,17,18,23a,24,25a-hexadecahydrofuro[2,3-q]pyrrolo[2,3-m][1,4,7,10,15,19]hexaazacyclotricosine-5,10,13,16,19,22,25(4*H*,21*H*,23*H*)-heptaone 28a

In a 4 mL brown glass-vial under an atmosphere of argon, protected dehydrosocein precursor **25a** (8.0 mg, 8.4 µmol) was dissolved in argon-degassed, anhydrous CH_2Cl_2 (200 µL). After the addition of styrene (9.73 µL, 84.1 µmol, 10 eq.) and Grubbs I catalyst in CH_2Cl_2 (42.0 µL, 0.841 µmol, 0.02 M, 10mol%), the reaction mixture was stirred for 24 h. Another portion of Grubbs I catalyst (42.0 µL, 0.841 µmol, 0.02 M, 10mol%) was added, and the stirring was continued for 56 h. After adsorption on isolute®, the mixture was purified by automated reversed phase column chromatography (C18 spherical, H_2O:MeCN 10% to 90% MeCN) to give the olefination product (3.5 mg, 3.41 µmol, 41%, 93%brsm) as a white foam.

1.0 M TBAF in THF (6.98 µL, 6.98 µmol, 2.05 eq.) was added to a 0 °C cold solution of the above-prepared olefination product (3.5 mg, 3.41 µmol) in anhydrous THF (70 µL). The resulting solution was stirred for 4 h while slowly reaching room temperature. After the addition of a droplet of H_2O, the reaction mixture was adsorbed on isolute® and purified by automated reversed phase column chromatography (C18 spherical, H_2O:MeCN 10% to 90% MeCN) followed by preparative HPLC

(H_2O:MeCN 10% to 100% MeCN) to give dehydrosocein derivative **28a** (2.6 mg, 3.25 µmol, 96%) as an amorphous solid after lyophilization.

LC-MS: t_R (28a) = 0.89 min (short method)

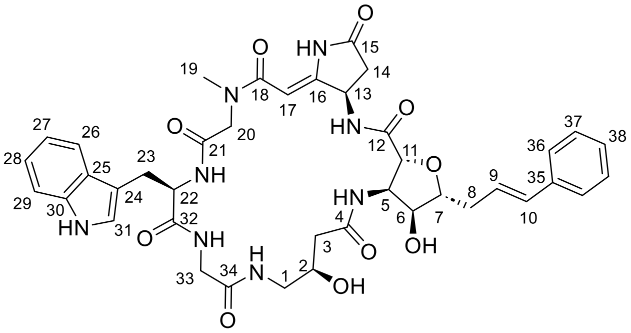

28a

^{1}H-NMR (500 MHz, DMSO-d$_6$): δ = 10.86 (d, $^3J_{NH,31}$ = 2.4 Hz, 1 H, 31-NH), 10.39 (s, 1 H, 15-NH), 8.57 (d, $^3J_{NH,22}$ = 5.7 Hz, 1 H, 21-NH), 8.48 (d, $^3J_{NH,13}$ = 8.3 Hz, 1 H, 12-NH), 8.29 (t, $^3J_{NH,33}$ = 6.0 Hz, 1 H, 32-NH), 7.60 (d, $^3J_{NH,5}$ = 8.6 Hz, 1 H, 4-NH), 7.52 (d, $^3J_{29,28}$ = 7.9 Hz, 1 H, 29-H), 7.39 (m, 2 H, 36-H), 7.34 (d, $^3J_{26,27}$ = 8.0 Hz, 1 H, 26-H), 7.30 (m, 2 H, 37-H), 7.24 (m, 1 H, 34-NH), 7.23 – 7.18 (m, 2 H, 31-H, 38-H), 7.07 (ddd, $^3J_{28,29}$ = 8.2 Hz, $^3J_{28,27}$ = 7.0 Hz, $^4J_{28,26}$ = 1.2 Hz, 1 H, 28-H), 6.99 (ddd, $^3J_{27,26}$ = 7.9 Hz, $^3J_{27,28}$ = 7.0 Hz, $^4J_{27,29}$ = 1.1 Hz, 1 H, 27-H), 6.49 (d, $^3J_{10,9}$ = 16.0 Hz, 1 H, 10-H), 6.37 (dt, $^3J_{9,10}$ = 15.9 Hz, $^3J_{9,8}$ = 7.0 Hz, 1 H, 9-H), 5.52 (d, $^3J_{OH,6}$ = 4.3 Hz, 1 H, 6-OH), 5.39 (d, $^4J_{17,13}$ = 1.6 Hz, 1 H, 17-H), 5.20 (m, 1 H, 13-H), 4.66 (d, $^3J_{OH,2}$ = 4.9 Hz, 1 H, 2-OH), 4.55 (d, $^3J_{20',20}$ = 15.8 Hz, 1 H, 20-H‘), 4.45 (ddd, $^3J_{5,11}$ = 8.7 Hz, $^3J_{5,NH}$ = 8.7 Hz, $^3J_{5,6}$ = 5.6 Hz, 1 H, 5-H), 4.18 (ddd, $^3J_{22,23}$ = 10.0 Hz, $^3J_{22,NH}$ = 5.2 Hz, $^3J_{22,23'}$ = 5.2 Hz, 1 H, 22-H), 3.98 (m, 1 H, 2-H), 3.95 – 3.91 (m, 2 H, 11-H, 7-H), 3.88 (m, 1 H, 6-H), 3.62 (m, 2 H, 33-H), 3.44 (d, $^2J_{20,20'}$ = 15.8 Hz, 1 H, 20-H), 3.19 (dd, $^2J_{23',23}$ = 14.6 Hz, $^3J_{23',22}$ = 4.6 Hz, 1 H, 23-H‘), 3.16 – 3.08 (m, 4 H, 1-H, 19-H), 3.01 (dd, $^2J_{23,23'}$ = 14.7 Hz, $^3J_{23,22}$ = 9.5 Hz, 1 H, 23-H), 2.79 (m, 1 H, 1-H‘), 2.75 (dd, $^2J_{14,14'}$ = 17.7 Hz, $^3J_{14,13}$ = 9.8 Hz, 1 H, 14-H), 2.46 (m, 2 H, 8-H), 2.33 (dd, $^2J_{14',14}$ = 17.8 Hz, $^3J_{14',13}$ = 6.3 Hz 1 H, 14-H‘), 2.22 (m, 2 H, 3-H).

^{13}C-NMR (125 MHz, DMSO-d$_6$): δ = 174.4 (s, C-15), 171.5 (s, C-32), 171.0 (s, C-21), 170.2 (s, C-4), 170.0 (s, C-12), 168.8 (s, C-34), 168.3 (s, C-18), 156.4 (s, C-16), 137.2 (s, C-35), 136.1 (s, C-30), 131.6 (d, C-10), 128.5 (d, C-37), 127.1 (d, C-38), 127.0 (s, C-25), 126.6 (d, C-9), 125.9 (d, C-36), 124.0 (d, C-31), 121.0 (d, C-28), 118.4 (d, C-27), 118.1 (d, C-29), 111.4 (d, C-26), 109.8 (s, C-24), 88.2 (d, C-17), 86.1 (d, C-7), 80.3 (d, C-11), 73.0 (d, C-6), 66.0 (d, C-2), 55.5 (d, C-22), 54.4 (d, C-5), 50.7 (t, C-20), 46.4 (d, C-13), 45.0 (t, C-1), 42.5 (t, C-33), 41.5 (t, C-3), 37.2 (q, C-19), 37.0 (t, C-8), 34.2 (t, C-14), 26.2 (t, C-23).

**Optical rotation:**	$[\alpha]_D^{20} = -28.6$ (c = 0.3, MeOH)	
**HRMS (ESI):**	calculated	found
$C_{40}H_{47}N_8O_{10}$ $[M+H]^+$:	799.3410	799.3420

(2*S*,3*S*,3a*R*,7*R*,14*R*,23a*R*,25a*R*,*Z*)-14-((1*H*-Indol-3-yl)methyl)-2-cinnamyl-3,7-dihydroxy-18-methyl-2,3,3a,6,7,8,9,11,12,14,15,17,18,23a,24,25a-hexadecahydrofuro[2,3-q]pyrrolo[2,3-m][1,4,7,10,15,19]hexaazacyclotricosine-5,10,13,16,19,22,25(4*H*,21*H*,23*H*)-heptaone 28b

In a 4 mL brown-glass vial under an atmosphere of argon, dehydrosocein precursor **27b** (3.5 mg, 4.84 µmol) was dissolved in argon-degassed AcOH (150 µL). After the addition of styrene (8.41 µL, 73.0 µmol, 15 eq.) and Grubbs II catalyst in AcOH (40.5 µL, 0.485 µmol, 0.012 M, 10mol%), the reaction mixture was stirred for 24 h. Another portion of Grubbs II catalyst (40.5 µL, 0.485 µmol, 0.012 M, 10mol%) was added, and the stirring was continued for 28 h at 40 °C. After adsorption on isolute®, the mixture was purified by automated reversed phase column chromatography (C18 spherical, H_2O:MeCN 10% to 90% MeCN) followed by preparative HPLC (0.1% $HCOOH_{aq}$:MeCN 0% to 90% MeCN) to give the dehydrosocein derivative **28b** (0.4 mg, 0.5 µmol, 10%) as an amorphous solid after lyophilization.

LC-MS: t_R (28b) = 0.87 min (short method)

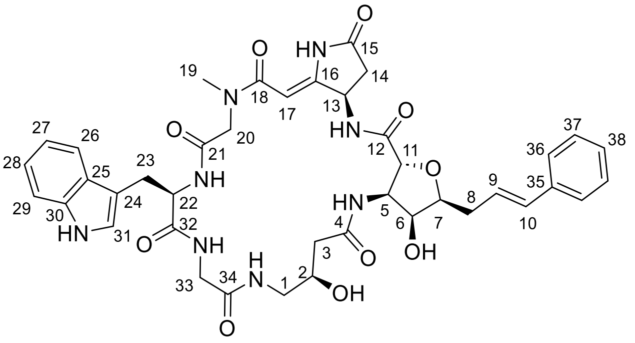

28b

^{1}H-NMR (500 MHz, DMSO-d$_6$): δ = 10.87 (d, $^3J_{NH,31}$ = 2.4 Hz, 1 H, 31-NH), 10.39 (s, 1 H, 15-NH), 8.58 – 8.48 (m, 2 H, 21-NH, 12-NH), 8.31 (t, $^3J_{NH,33}$ = 5.6 Hz, 1 H, 32-NH), 7.81 (d, $^3J_{NH,5}$ = 8.4 Hz, 1 H, 4-NH), 7.52 (d, $^3J_{29,28}$ = 7.9 Hz, 1 H, 29-H), 7.38 (m, 2 H, 36-H), 7.33 (d, $^3J_{26,27}$ = 8.1 Hz, 1 H, 26-H), 7.32 – 7.27 (m, 3 H, 34-NH, 37-H), 7.22 – 7.17 (m, 2 H, 31-H, 38-H), 7.07 (m, 1 H, 28-H), 6.99 (m, 1 H, 27-H), 6.48 (d, $^3J_{10,9}$ = 15.9 Hz, 1 H, 10-H), 6.28 (dt, $^3J_{9,10}$ = 15.9 Hz, $^3J_{9,8}$ = 7.1 Hz, 1 H, 9-H), 5.51 (m, 1 H, 6-OH), 5.39 (s, 1 H, 17-H), 5.16 (m, 1 H, 13-H), 4.71 (m, 1 H, 2-OH), 4.57 (d, $^2J_{20',20}$ = 15.8 Hz, 1 H, 20-H'), 4.54 (m, 1 H, 5-H), 4.22 – 4.13 (m, 2 H, 7-H, 22-H), 4.05 – 3.94 (m, 2 H, 2-H, 6-H), 3.96 (d, $^3J_{11,5}$ = 8.8 Hz, 1 H, 11-H), 3.65 (dd, $^2J_{33,33'}$ = 16.9 Hz, $^3J_{33,NH}$ = 5.6 Hz, 1 H, 33-H), 3.57 (m, 1 H, 33-H'), 3.42 (d, $^2J_{20,20'}$ = 15.8 Hz, 1 H, 20-H), 3.18 (dd, $^2J_{23',23}$ = 14.9 Hz, $^3J_{23',22}$ = 4.7 Hz, 1 H, 23-H'), 3.15 – 3.06 (m, 4 H, 1-H, 19-H), 3.01 (dd, $^2J_{23,23'}$ = 14.7 Hz, $^3J_{23,22}$ = 9.4 Hz, 1 H, 23-H), 2.79 (m, 1 H, 1-H'), 2.74 (dd, $^2J_{14,14'}$ = 17.6 Hz, $^3J_{14,13}$ = 9.6 Hz, 1 H, 14-H), 2.32 (dd, $^2J_{14',14}$ = 17.6 Hz, $^3J_{14,13}$ = 6.1 Hz, 1 H, 14-H'), 2.44 (m, 2 H, 8-H, covered by the DMSO-d$_5$ signal), 2.24 (m, 2 H, 3-H).

^{13}C-NMR-shifts extracted from 2D-NMR data (HMBC, HSQCED) due to insufficient material for a 1D ^{13}C-NMR (125 MHz, DMSO-d$_6$): δ = 174.3 (s, C-15), 170.8 (s, C-12), 170.2 (s, C-4), 168.2 (s, C-18), 156.3 (s, C-16), 136.2 (s, C-30), 131.1 (d, C-10), 128.3 (d, C-37), 127.0 (s, C-25), 126.7 (d, d, C-9, C-38), 125.9 (s, C-35), 125.6 (d, C-36), 123.7 (d, C-31), 120.8 (d, C-28), 118.0 (d, C-27), 117.9 (d, C-29), 111.1 (d, C-26), 109.8 (s, C-24), 87.7 (d, C-17), 82.6 (d, C-7), 79.0 (d, C-11), 70.8 (d, C-6), 65.8

(d, C-2), 56.2 (d, C-5), 55.0 (d, C-22), 50.3 (t, C-20), 46.0 (d, C-13), 44.7 (t, C-1), 42.1 (t, C-33), 41.4 (t, C-3), 36.8 (q, C-19), 35.0 (t, C-8), 34.3 (t, C-14), 26.0 (t, C-23), C-21, C-32 and C-34 were not observed.

Optical rotation: insufficient amount of material

**HRMS (ESI):**	calculated	found
$C_{40}H_{47}N_8O_{10}$ [M+H]$^+$:	799.3410	799.3417

(2*R*,3*S*,3a*R*,7*R*,14*R*,23a*R*,25a*S*,*Z*)-14-((1*H*-Indol-3-yl)methyl)-2-cinnamyl-3,7-dihydroxy-18-methyl-2,3,3a,6,7,8,9,11,12,14,15,17,18,23a,24,25a-hexadecahydrofuro[2,3-q]pyrrolo[2,3-m][1,4,7,10,15,19]hexaazacyclotricosine-5,10,13,16,19,22,25(4*H*,21*H*,23*H*)-heptaone 28c

In a 4 mL brown-glass vial under an atmosphere of argon, protected dehydrosocein precursor **25c** (8.4 mg, 8.8 µmol) was dissolved in argon-degassed, anhydrous CH_2Cl_2 (150 µL). After the addition of styrene (15.3 µL, 132 µmol, 15 eq.) and Grubbs II catalyst in CH_2Cl_2 (73.6 µL, 0.883 µmol, 0.012 M, 10mol%), the reaction mixture was stirred for 48 h. Another portion of Grubbs II catalyst (73.6 µL, 0.883 µmol, 0.012 M, 10mol%) was added, and the stirring was continued for 24 h. After adsorption on isolute®, the mixture was purified by automated reversed phase column chromatography (C18 spherical, H_2O:MeCN 10% to 90% MeCN) to give the olefination product **26c** (4.5 mg, 4.4 µmol, 50%) as a white foam.

1.0 M TBAF in THF (5.84 µL, 5.84 µmol, 1.5 eq.) was added to a 0 °C cold solution of the above-prepared olefination product **26c** (4.0 mg, 3.9 µmol) in anhydrous THF (70 µL). The resulting solution was stirred for 4 h while slowly reaching room temperature. After the addition of a droplet of H_2O, the reaction mixture was adsorbed on isolute® and purified by automated reversed phase column chromatography (C18 spherical, H_2O:MeCN 10% to 90% MeCN) followed by preparative HPLC (H_2O:MeCN 10% to 100% MeCN) to give dehydrosocein derivative **28c** (2.1 mg, 2.6 µmol, 68%) as an amorphous solid after lyophilization.

LC-MS: t_R (28c) = 0.90 min (short method)

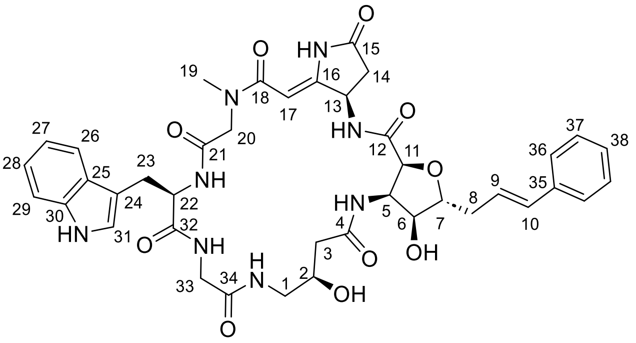

28c

^{1}H-NMR (500 MHz, MeOD-d$_4$): δ = 7.68 (d, $^3J_{29,28}$ = 7.9 Hz, 1 H, 29-H), 7.36 (m, 2 H, 36-H), 7.30 – 7.23 (m, 3 H, 26-H, 37-H), 7.18 (m, 1 H, 38-H), 7.16 (s, 1 H, 31-H), 7.11 (m, 1 H, 28-H), 7.04 (m, 1 H, 27-H), 6.49 (d, $^3J_{10,9}$ = 16.2 Hz , 1 H, 10-H), 6.22 (dt, $^3J_{9,10}$ = 15.8 Hz, $^3J_{9,8}$ = 7.1 Hz, 1 H, 9-H),

4.80 (s, 1 H, 17-H), 4.67 (dd, $^3J_{5,11}$ = 8.5 Hz, $^3J_{5,6}$ = 4.5 Hz, 1 H, 5-H), 4.49 (dd, $^3J_{22,23}$ = 10.9 Hz, $^3J_{22,23'}$ = 4.4 Hz, 1 H, 22-H), 4.34 (d, $^3J_{11,5}$ = 8.5 Hz, 1 H, 11-H), 4.27 (m, 1 H, 13-H), 4.24 (d, $^2J_{20',20}$ = 17.8 Hz, 1 H, 20-H'), 4.21 – 4.13 (m, 2 H, 2-H, 7-H), 4.05 (d, $^3J_{6,5}$ = 4.5 Hz, 1 H, 6-H), 4.02 (d, $^2J_{33',33}$ = 16.6 Hz, 1 H, 33-H'), 3.76 (d, $^2J_{33,33'}$ = 16.5 Hz, 1 H, 33-H), 3.72 (d, $^2J_{20,20'}$ = 17.9 Hz, 1 H, 20-H), 3.46 (m, 1 H, 1-H'), 3.37 (dd, $^2J_{23',23}$ = 14.4 Hz, $^3J_{23',22}$ = 4.5 Hz, 1 H, 23-H'), 3.17 (m, 1 H, 1-H), 3.03 (dd, $^2J_{23,23'}$ = 14.5 Hz, $^3J_{23,22}$ = 10.9 Hz, 1 H, 23-H), 2.93 (s, 3 H, 19-H), 2.70 (dd, $^2J_{3,3'}$ = 14.3 Hz, $^3J_{3,2}$ = 9.9 Hz, 1 H, 3-H), 2.63 – 2.33 (m, 5 H, 3-H', 8-H, 14-H).

^{13}C-NMR (125 MHz, MeOD-d$_4$): δ = 177.3 (s, C-15), 174.8 (s, C-32), 174.0 (s, C-12), 173.5 (s, C-4), 173.1 (s, C-21), 172.4 (s, C-34), 170.1 (s, C-18), 157.3 (s, C-16), 138.8 (s, C-35), 138.5 (s, C-30), 134.2 (d, C-10), 129.5 (d, C-37), 128.3 (d, C-38), 128.1 (s, C-25), 127.1 (d, C-36), 126.2 (d, C-9), 125.2 (d, C-31), 122.6 (d, C-28), 120.1 (d, C-27), 119.6 (d, C-29), 112.6 (d, C-26), 110.0 (s, C-24), 90.2 (d, C-17), 87.9 (d, C-7), 75.7 (d, C-11), 73.6 (d, C-6), 68.7 (d, C-2), 56.3 (d, C-22), 55.1 (d, C-5), 54.4 (t, C-20), 46.8 (d, C-13), 46.4 (t, C-1), 44.2 (t, C-33), 41.1 (t, C-3), 38.8 (t, C-8), 35.6 (q, C-19), 35.2 (t, C-14), 28.3 (t, C-23).

Selected rotamer signals:

^{1}H-NMR (500 MHz, MeOD-d$_4$): δ = 7.57 (d, $^3J_{29,28}$ = 7.9 Hz, 1 H, 29-H), 7.02 (m, 1 H, 27-H), 6.53 (d, $^3J_{10,9}$ = 15.6 Hz, 1 H, 10-H), 6.30 (dt, $^3J_{9,10}$ = 15.2 Hz, $^3J_{9,8}$ = 7.1 Hz, 1 H, 9-H), 5.67 (s, 1 H, 17-H), 5.27 (m, 1 H, 13-H), 4.73 (dd, $^3J_{5,11}$ = 4.9 Hz, $^3J_{5,6}$ = 4.9 Hz, 1 H, 5-H), 4.53 (d, $^3J_{11,5}$ = 4.6 Hz, 1 H, 11-H), 4.41 (d, $^2J_{20',20}$ = 15.7 Hz, 1 H, 20-H'), 4.09 (m, 1 H, 6-H), 3.11 (s, 3 H, 19-H), 2.84 (m, 1 H, 14-H).

**Optical rotation:**	$[\alpha]_D^{20}$= –39.3 (c = 0.3, MeOH)	
**HRMS (ESI):**	calculated	found
$C_{40}H_{47}N_8O_{10}$ [M+H]$^+$:	799.3410	799.3406

benzyl (*R*)-4-amino-3-((*tert*-butoxycarbonyl)amino)-4-oxobutanoate 1

^{1}H-NMR (400 MHz, $CDCl_3$):

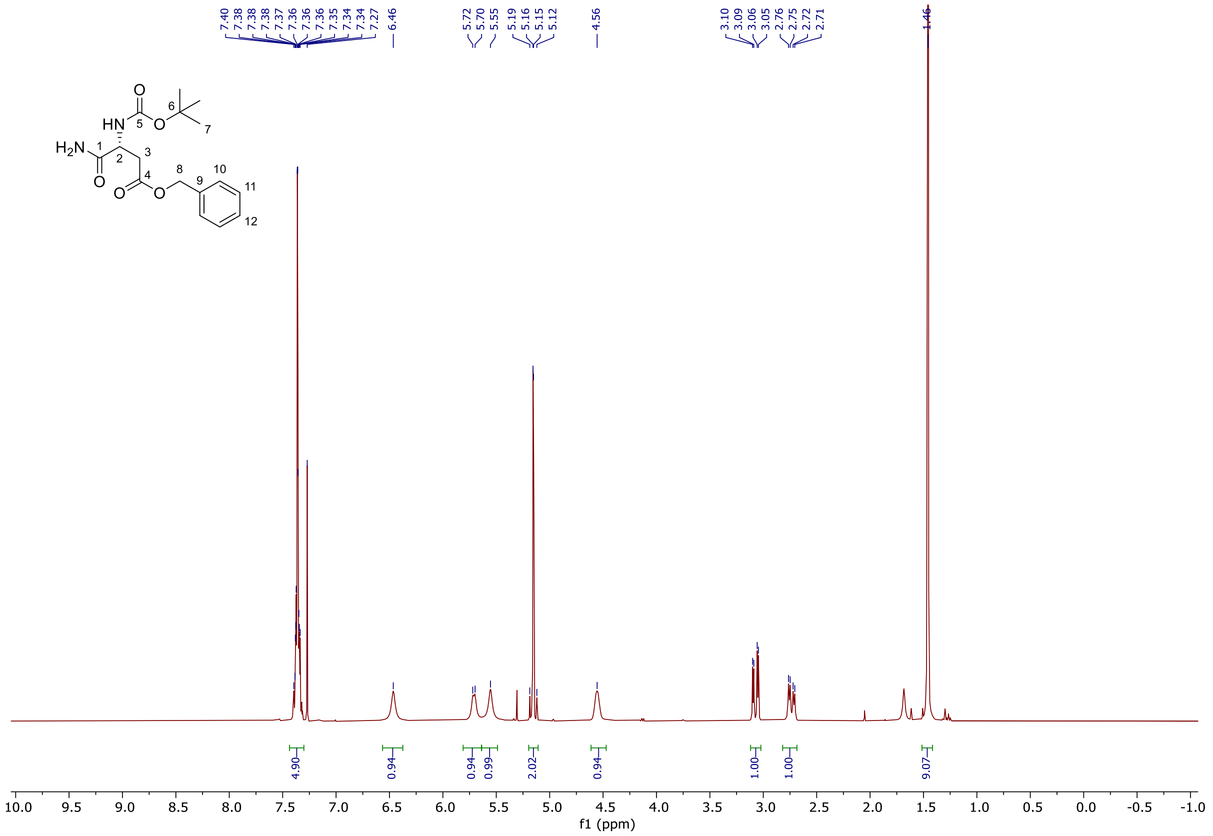

^{13}C-NMR (100 MHz, $CDCl_3$):

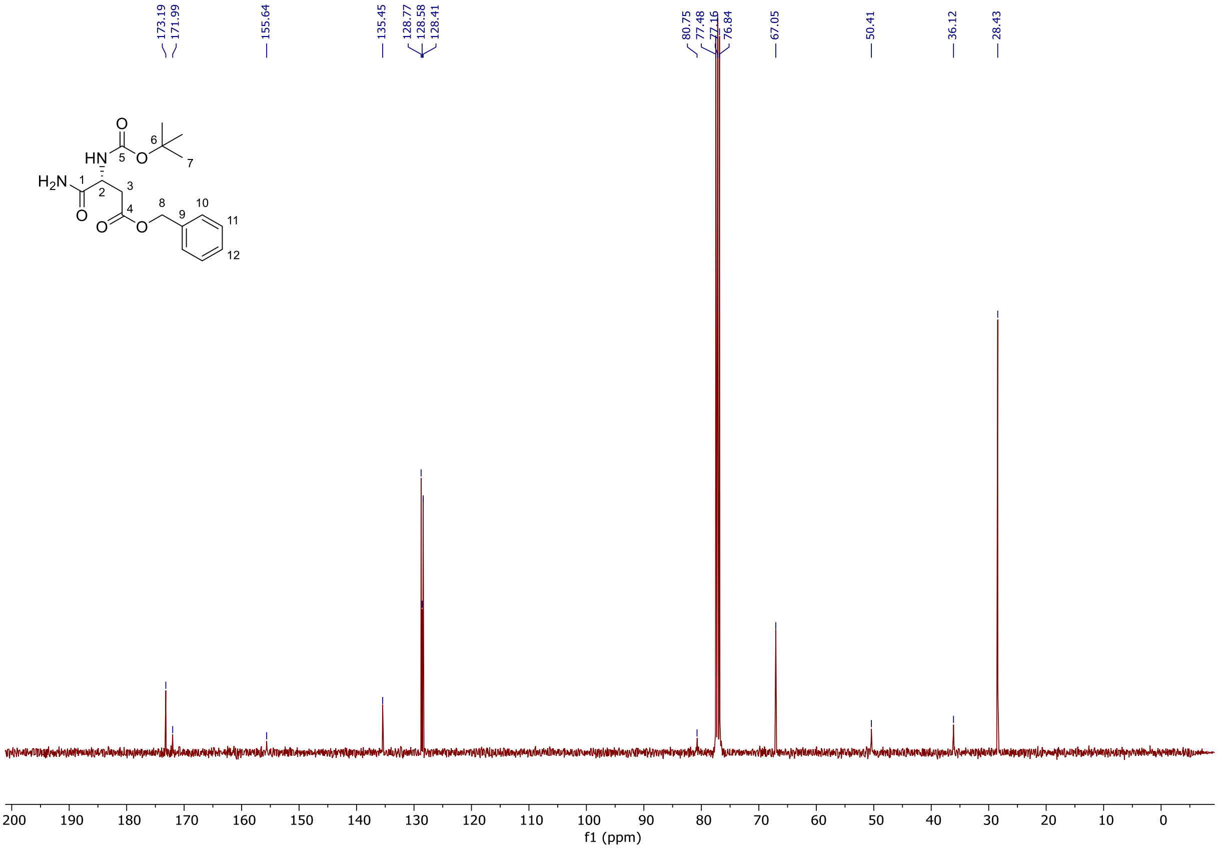

benzyl (*R*)-3-((*tert*-butoxycarbonyl)amino)-3-cyanopropanoate 2

^{1}H-NMR (400 MHz, $CDCl_3$):

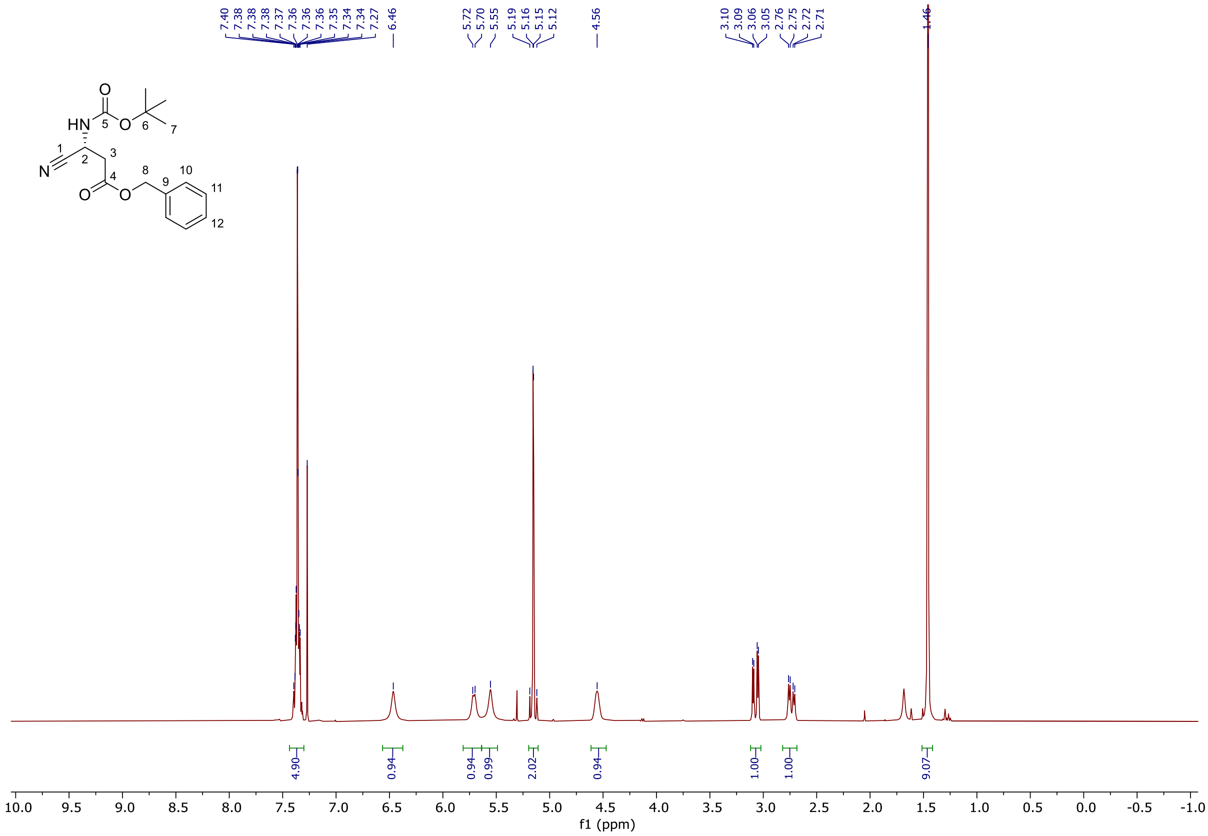

^{13}C-NMR (100 MHz, $CDCl_3$):

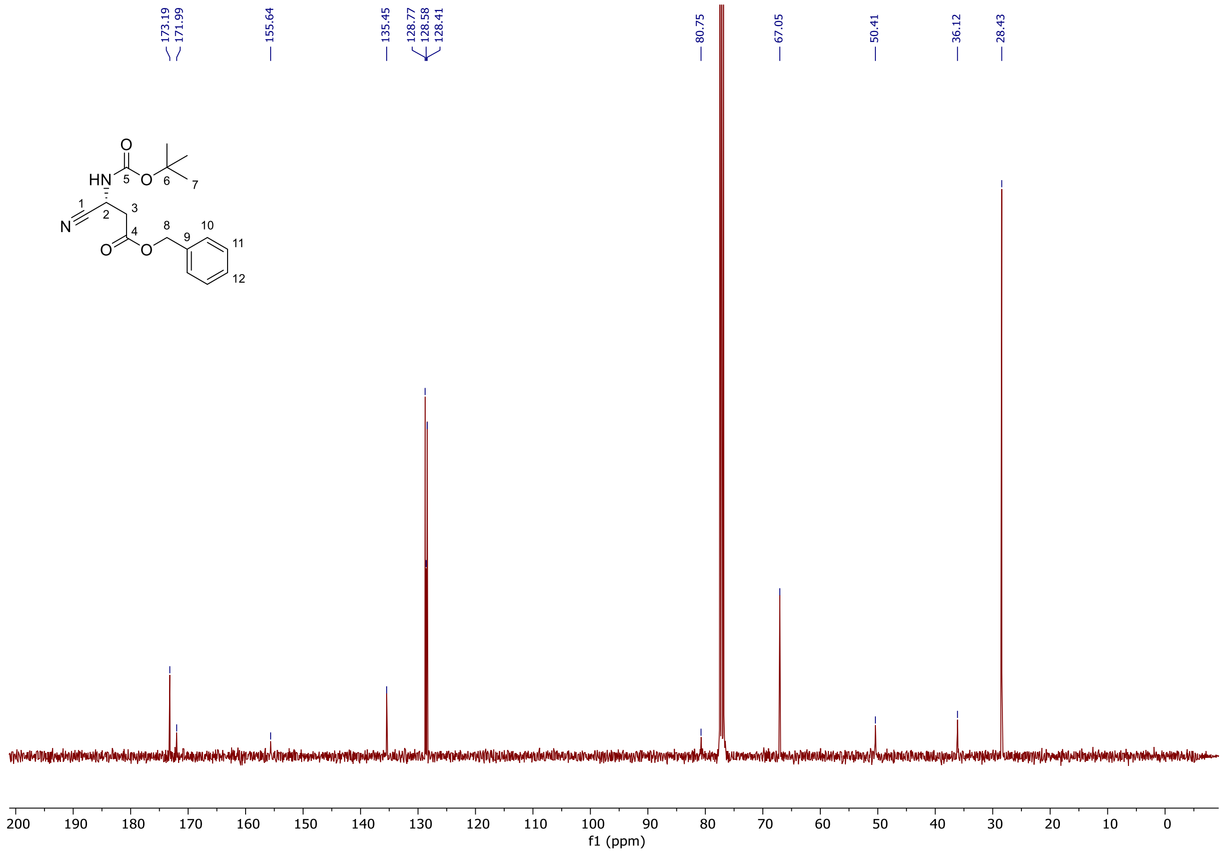

***tert*-Butyl (*R*,*Z*)-2-(3-((*tert*-butoxycarbonyl)amino)-5-oxopyrrolidin-2-ylidene)acetate 3**

^{1}H-NMR (400 MHz, $CDCl_3$):

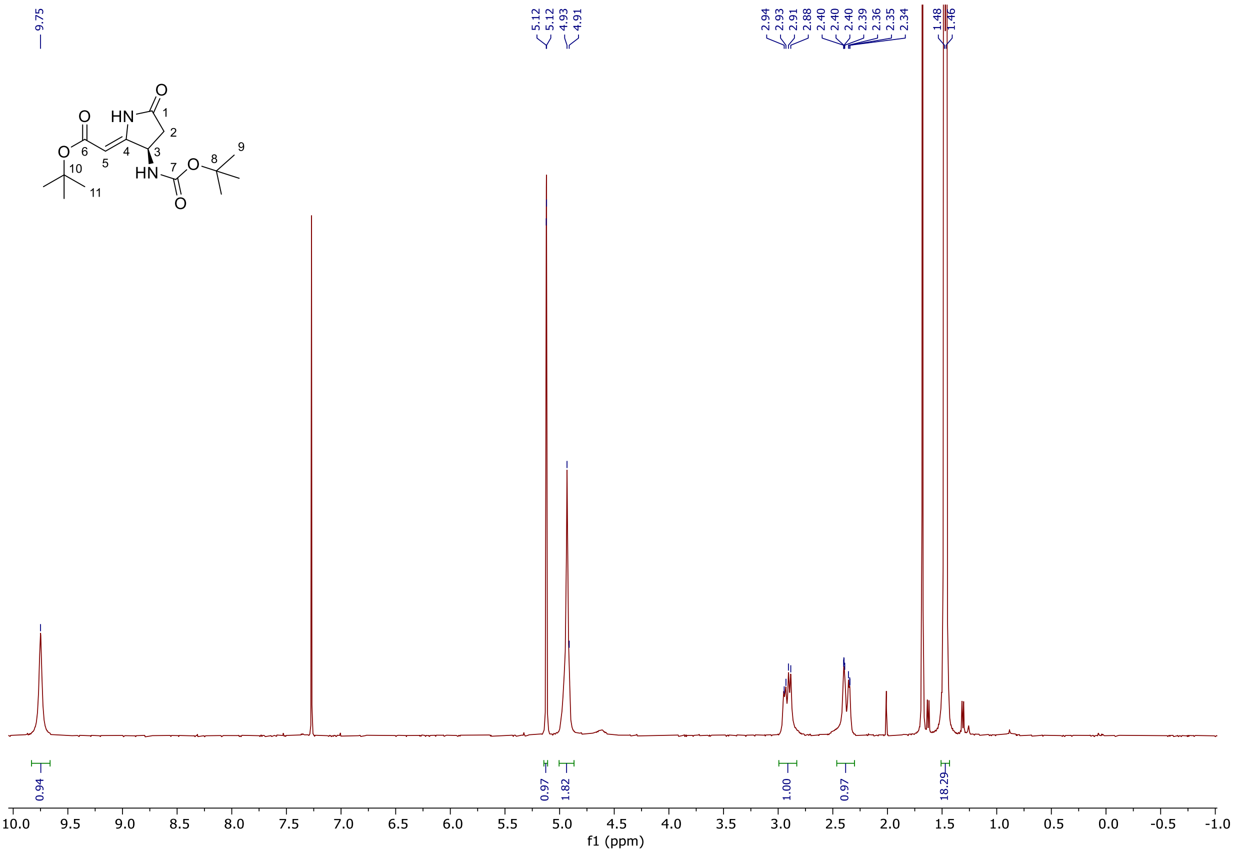

^{13}C-NMR (100 MHz, $CDCl_3$):

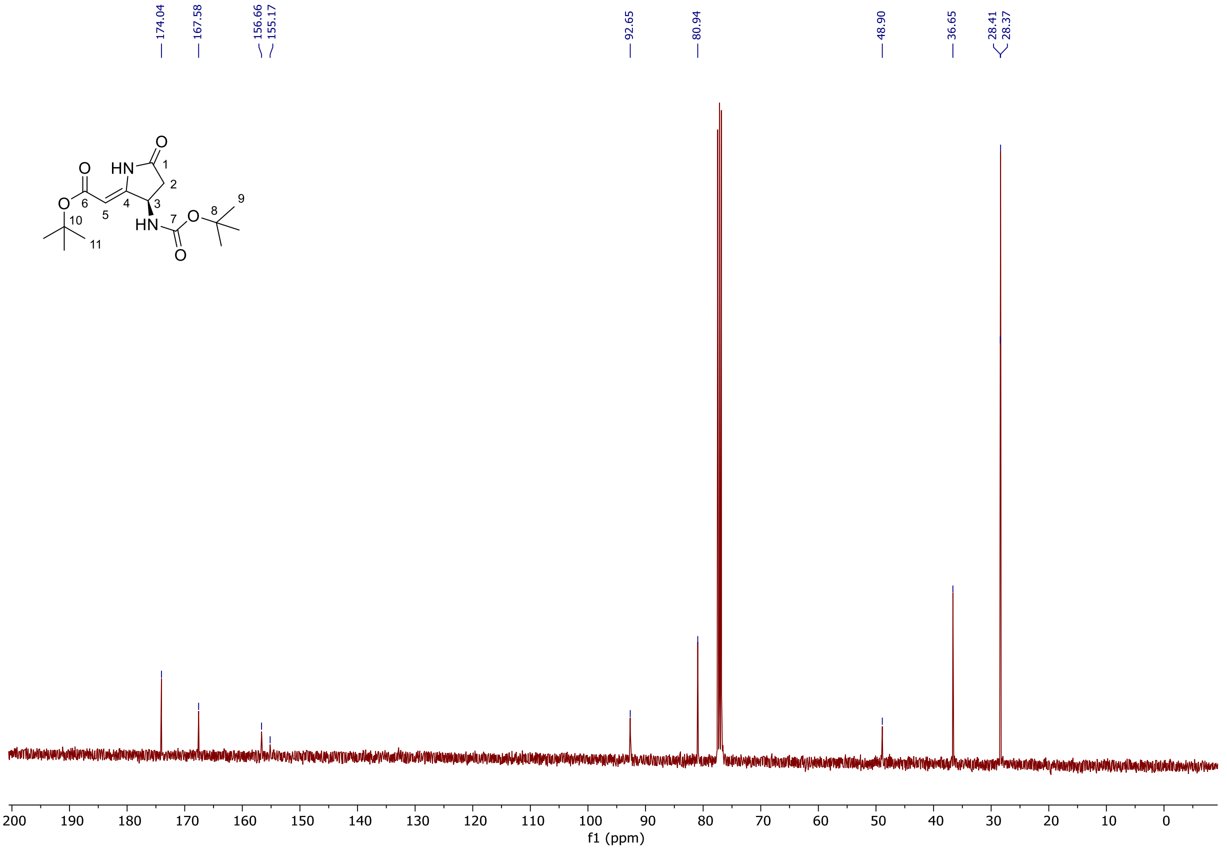

(*R*,*Z*)-2-(3-((*tert*-Butoxycarbonyl)amino)-5-oxopyrrolidin-2-ylidene)acetic acid SI-1

^{1}H-NMR (500 MHz, DMSO-d$_6$):

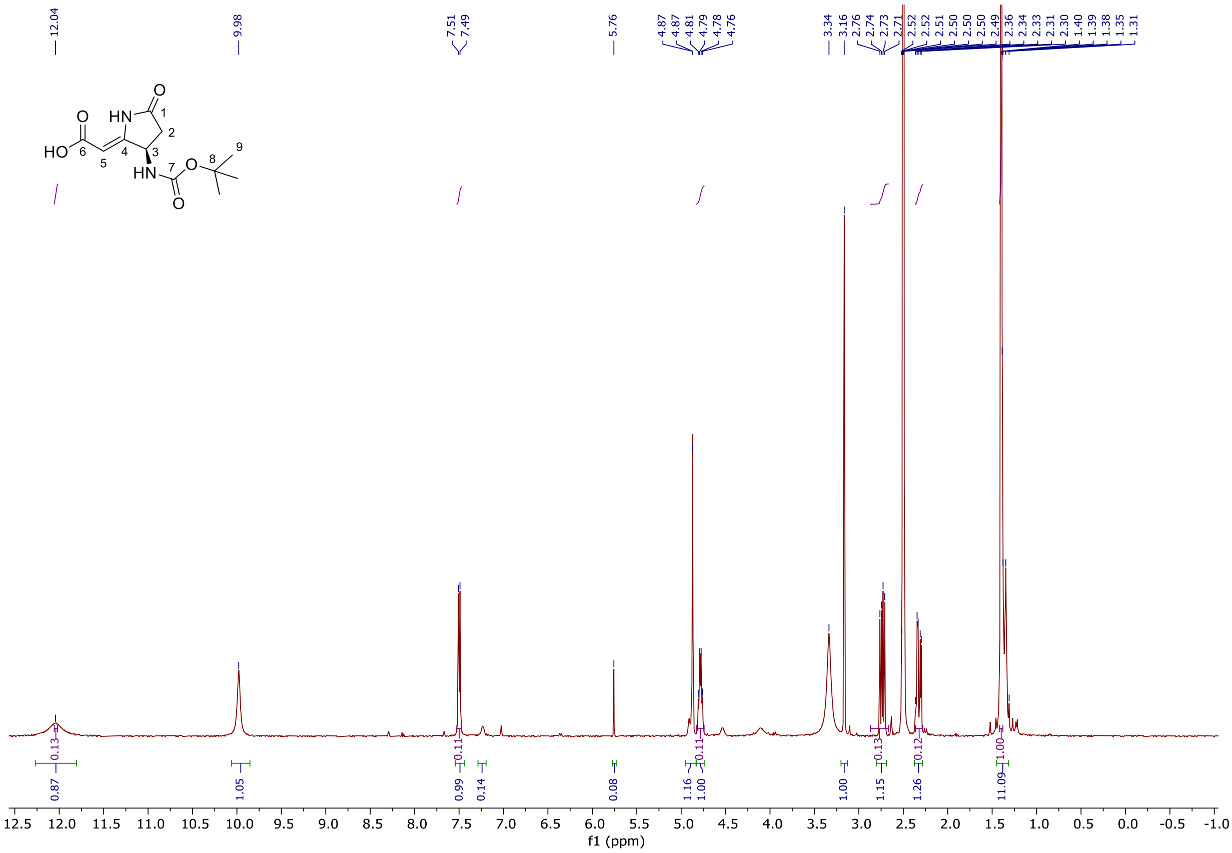

^{13}C-NMR (125 MHz, DMSO-d$_6$):

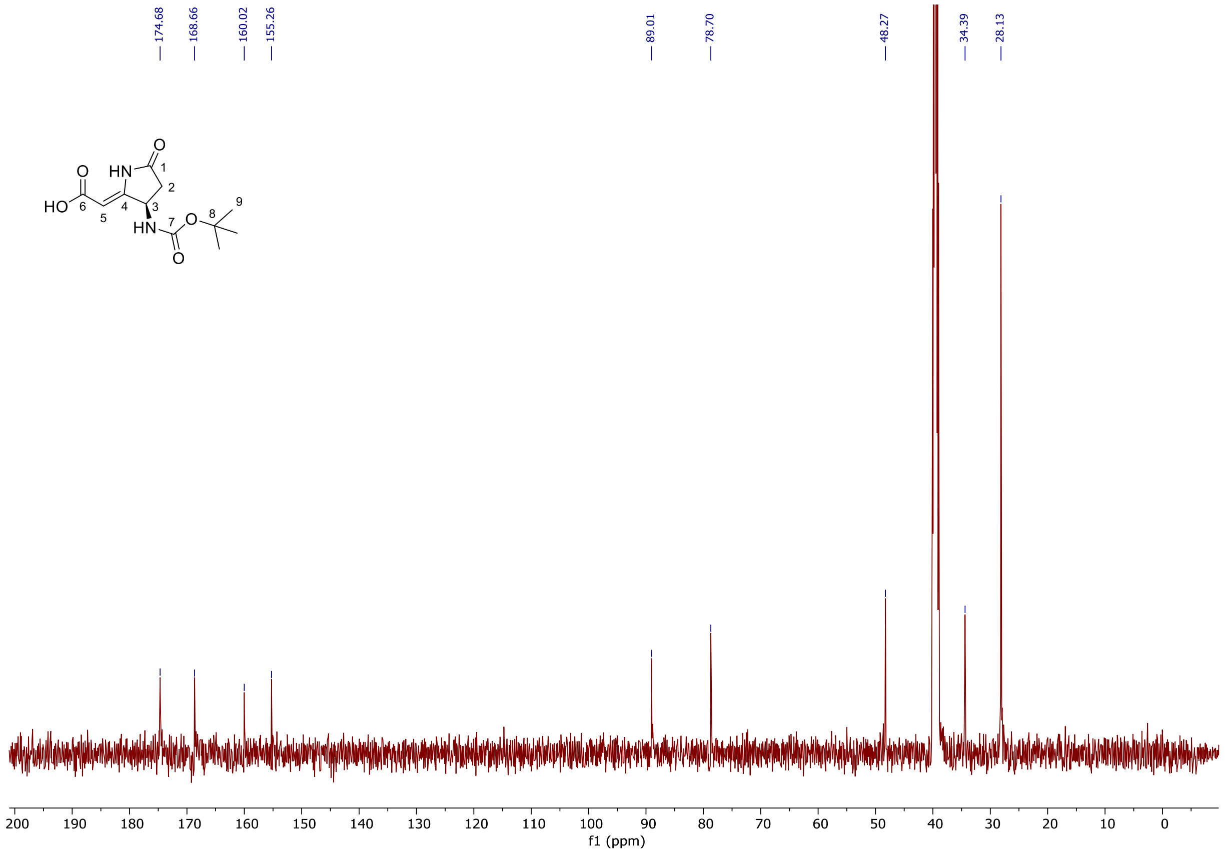

Methyl (*R*,*Z*)-*N*-(2-(3-((*tert*-butoxycarbonyl)amino)-5-oxopyrrolidin-2-ylidene)acetyl)-*N*-methylglycinate 4

^{1}H-NMR (500 MHz, DMSO-d$_6$, 373 K):

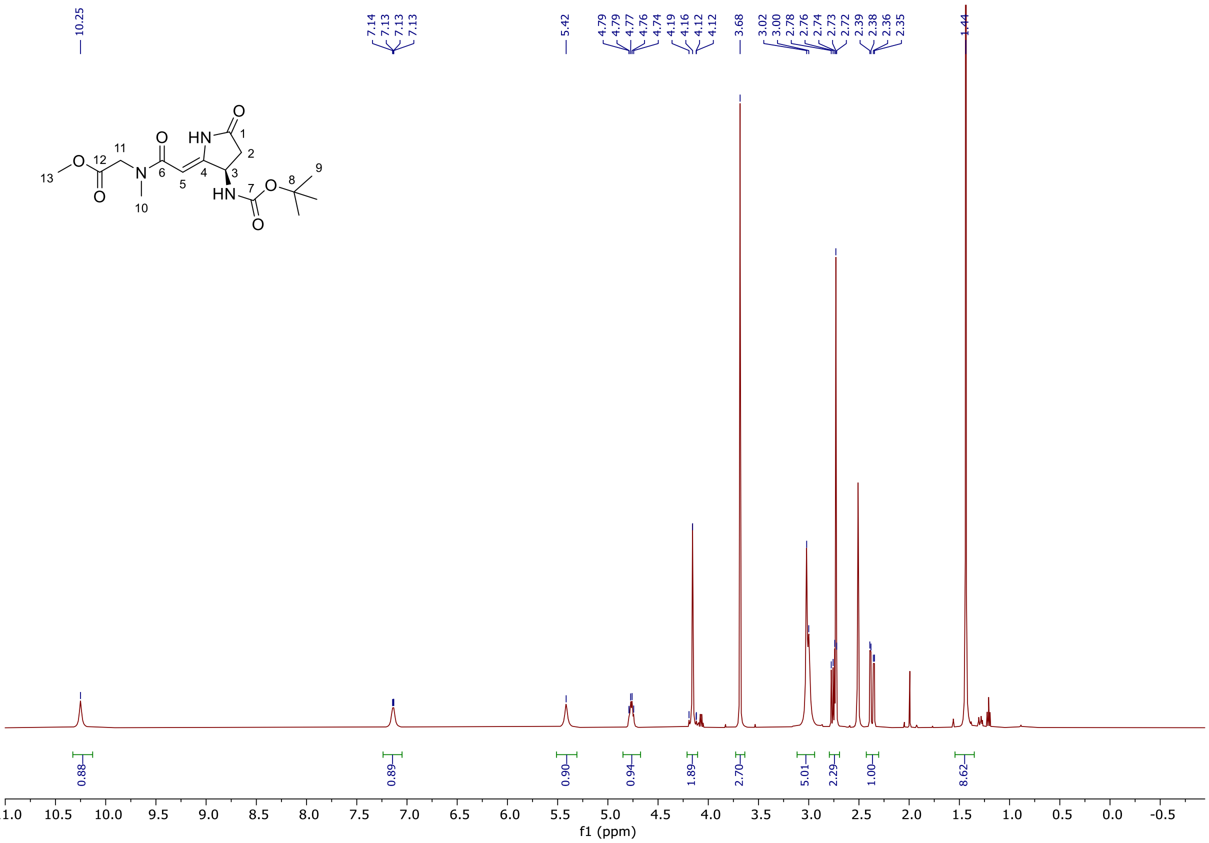

^{13}C-NMR (125 MHz, DMSO-d$_6$): impurity tetramethylurea (38.7 ppm)

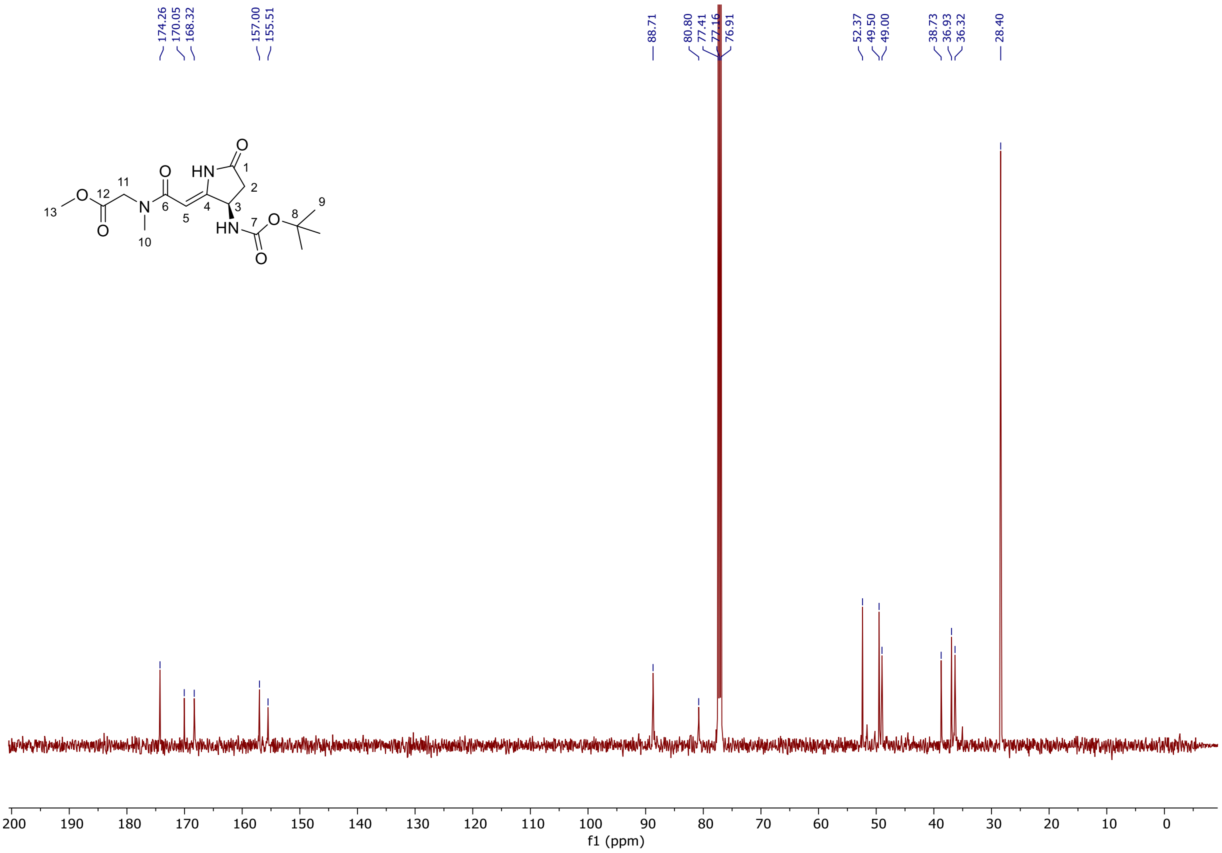

Methyl (*S*)-3-((*tert*-butoxycarbonyl)amino)-2-hydroxypropanoate 5

^{1}H-NMR (400 MHz, $CDCl_3$):

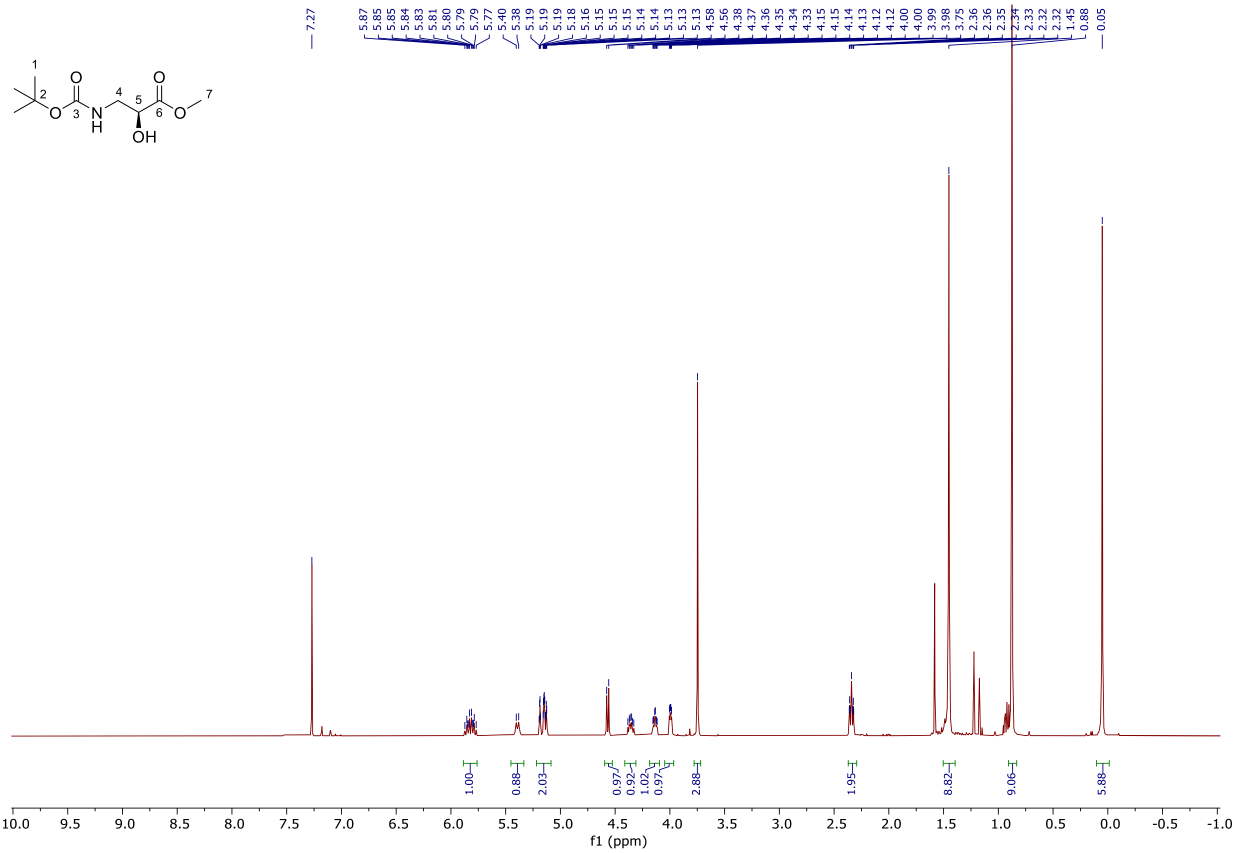

^{13}C-NMR (100 MHz, $CDCl_3$):

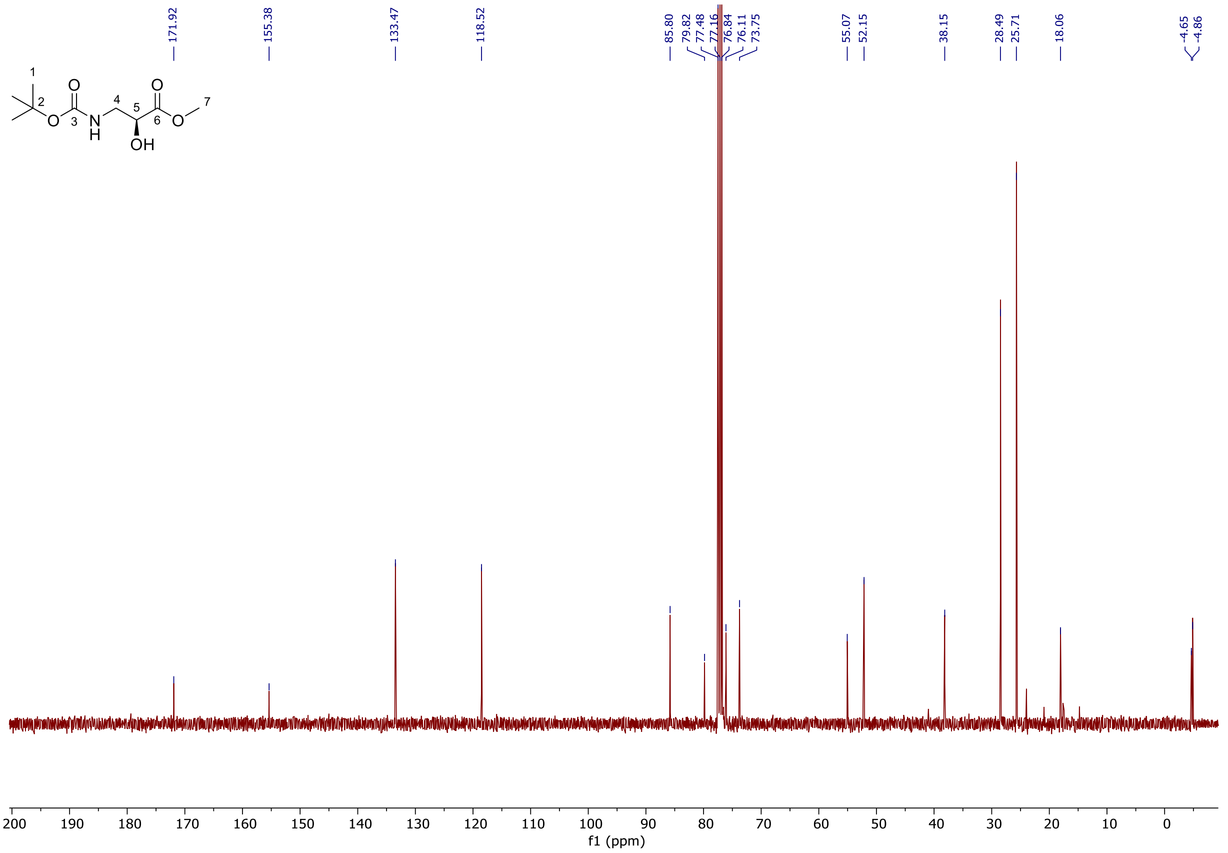

Methyl (*S*)-3-((*tert*-butoxycarbonyl)amino)-2-((*tert*-butyldimethylsilyl)oxy)propanoate 6

^{1}H-NMR (400 MHz, $CDCl_3$):

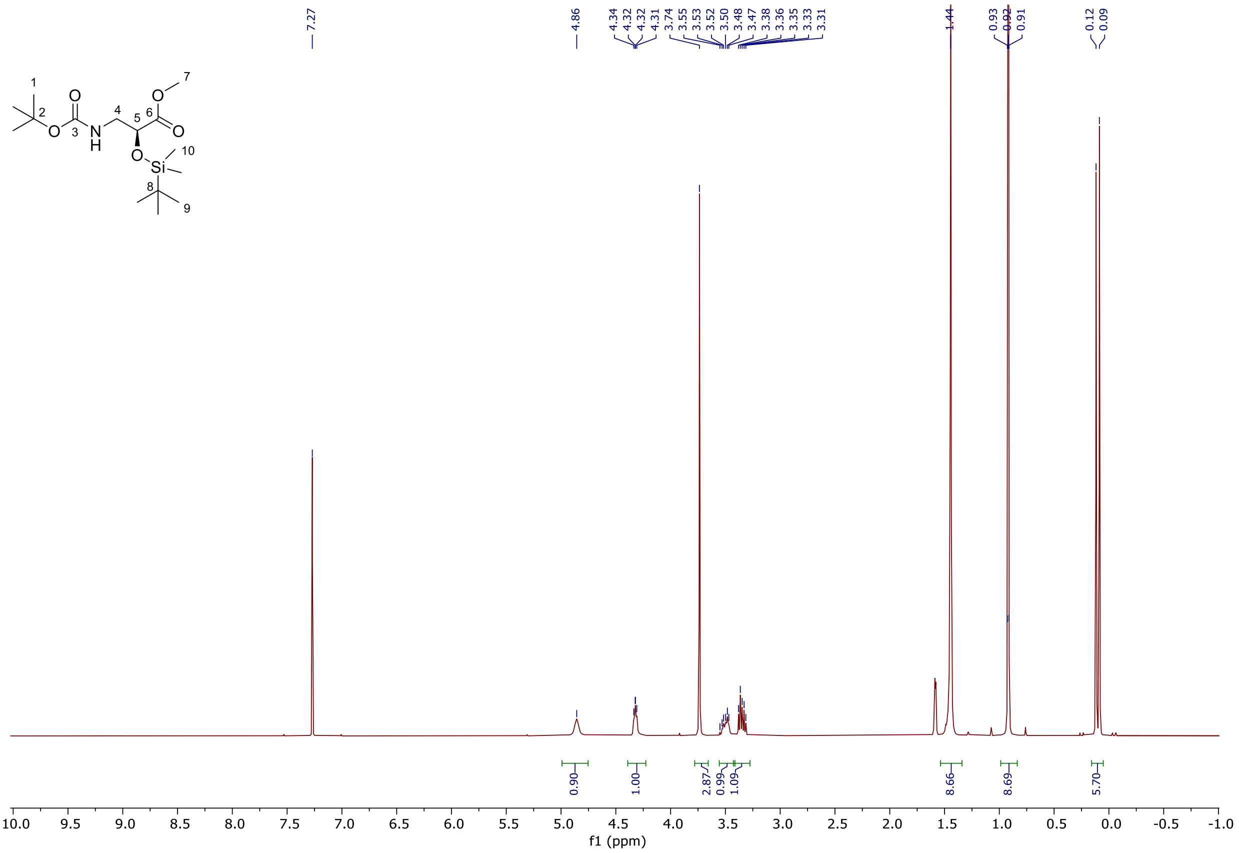

^{13}C-NMR (100 MHz, $CDCl_3$):

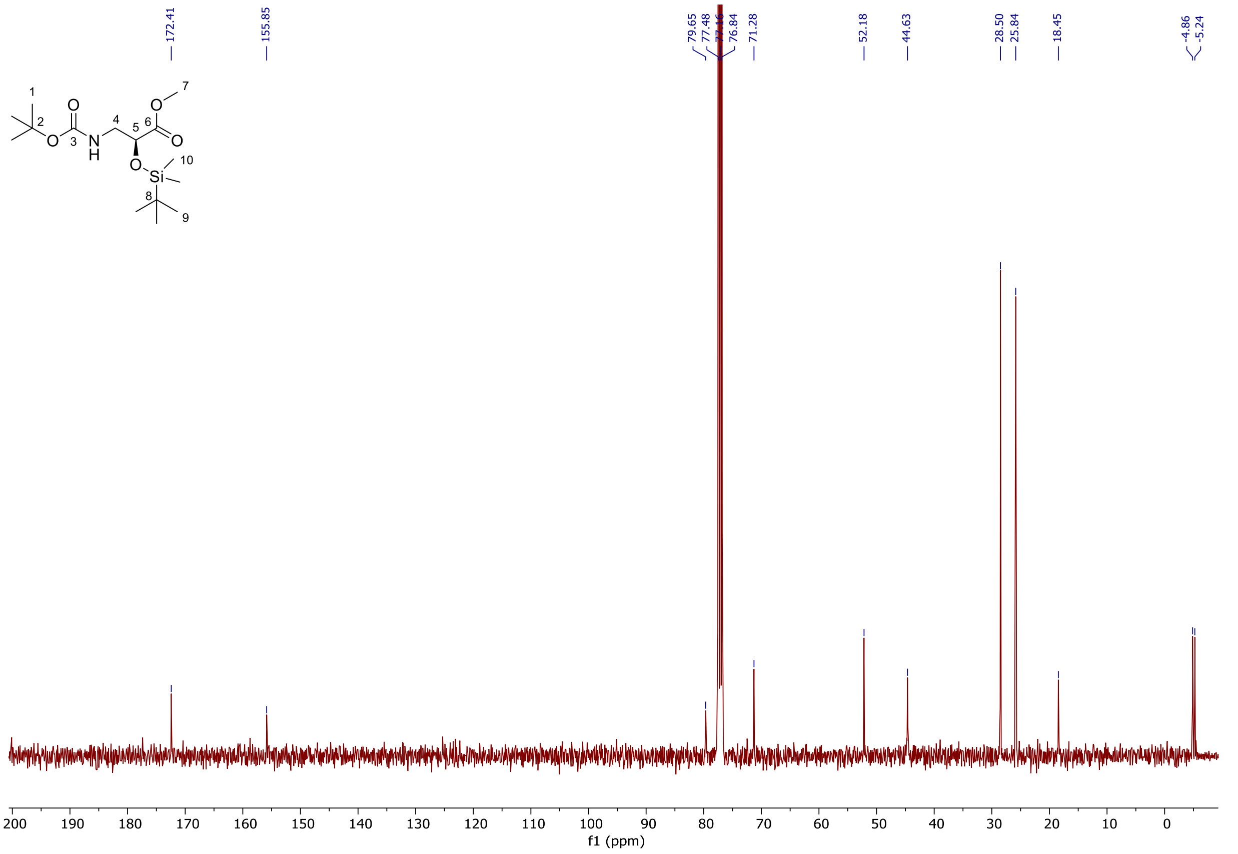

tert-Butyl (_S_)-(2-((_tert_-butyldimethylsilyl)oxy)-4-diazo-3-oxobutyl)carbamate 8

^{1}H-NMR (400 MHz, $CDCl_3$):

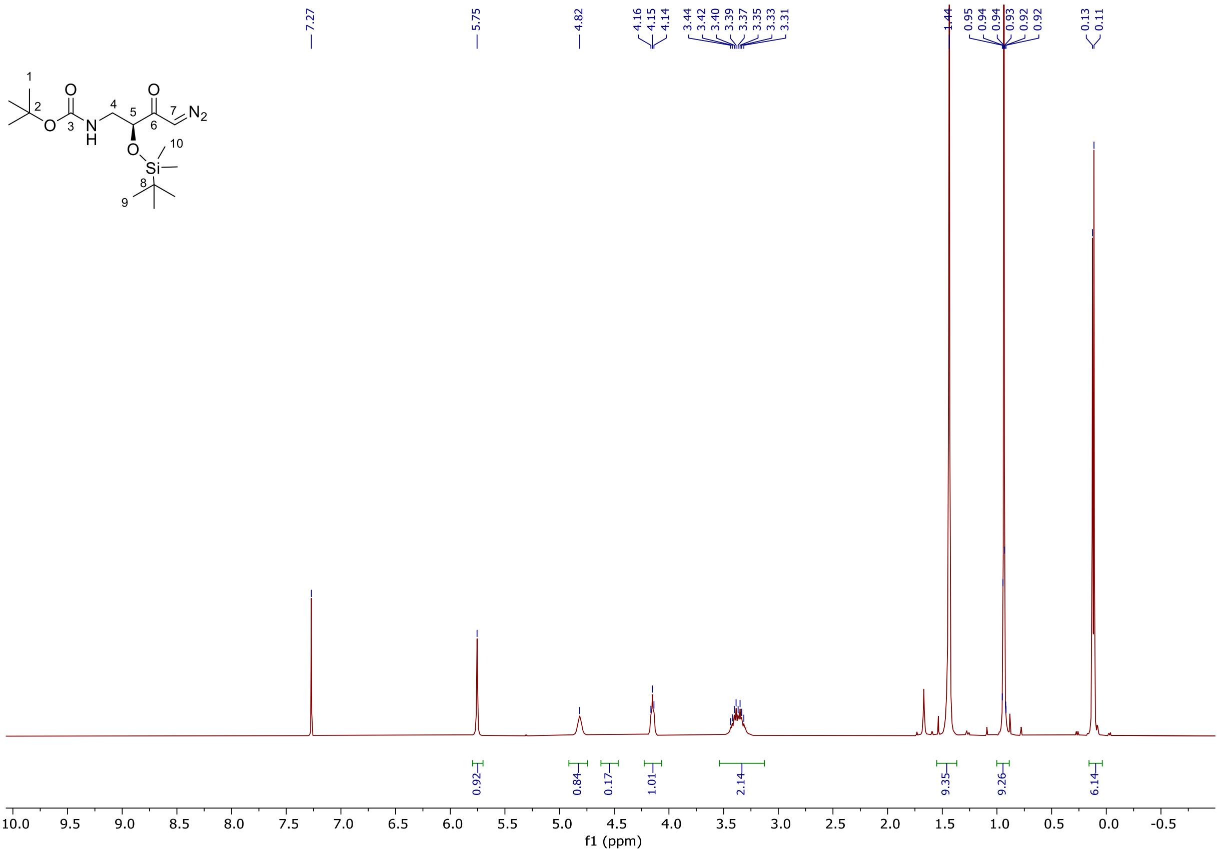

^{13}C-NMR (100 MHz, $CDCl_3$):

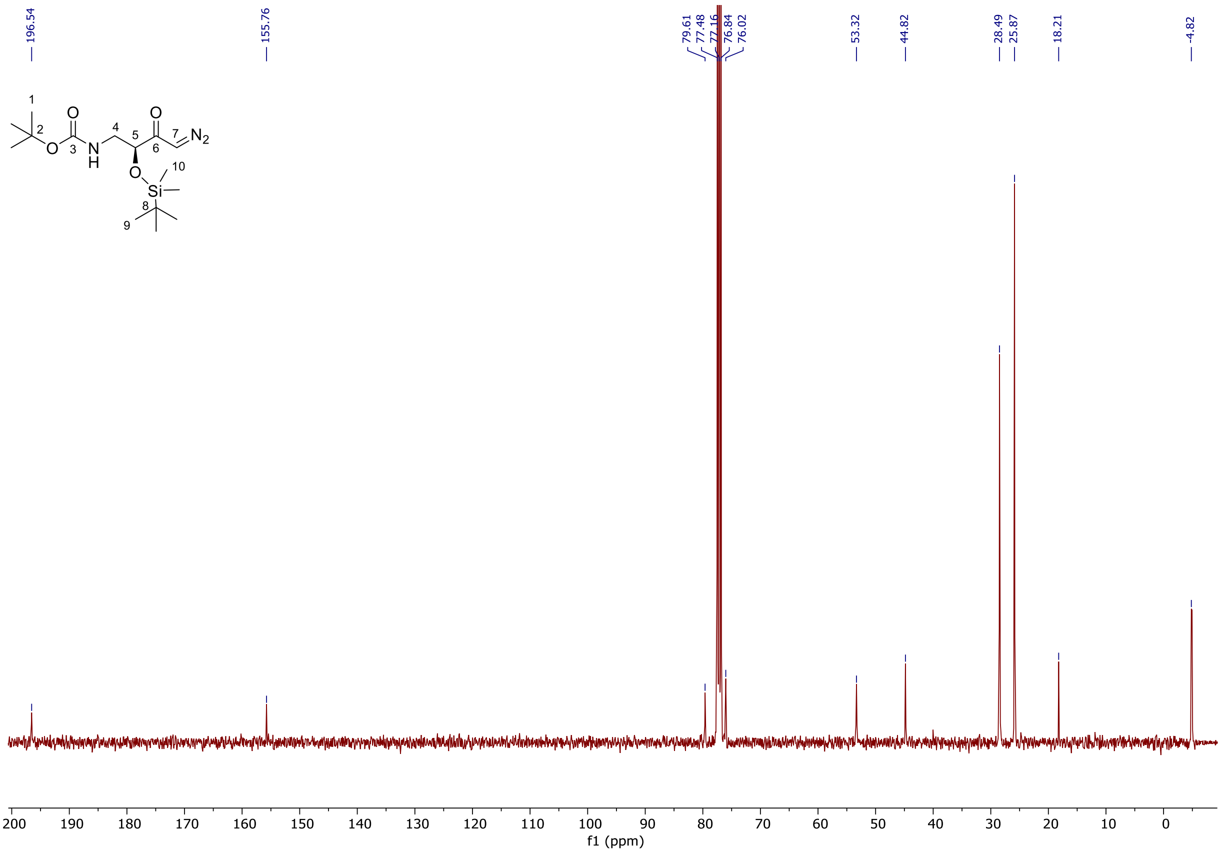

Methyl (*R*)-4-((*tert*-butoxycarbonyl)amino)-3-((*tert*-butyldimethylsilyl)oxy)butanoate 9

^{1}H-NMR (500 MHz, $CDCl_3$):

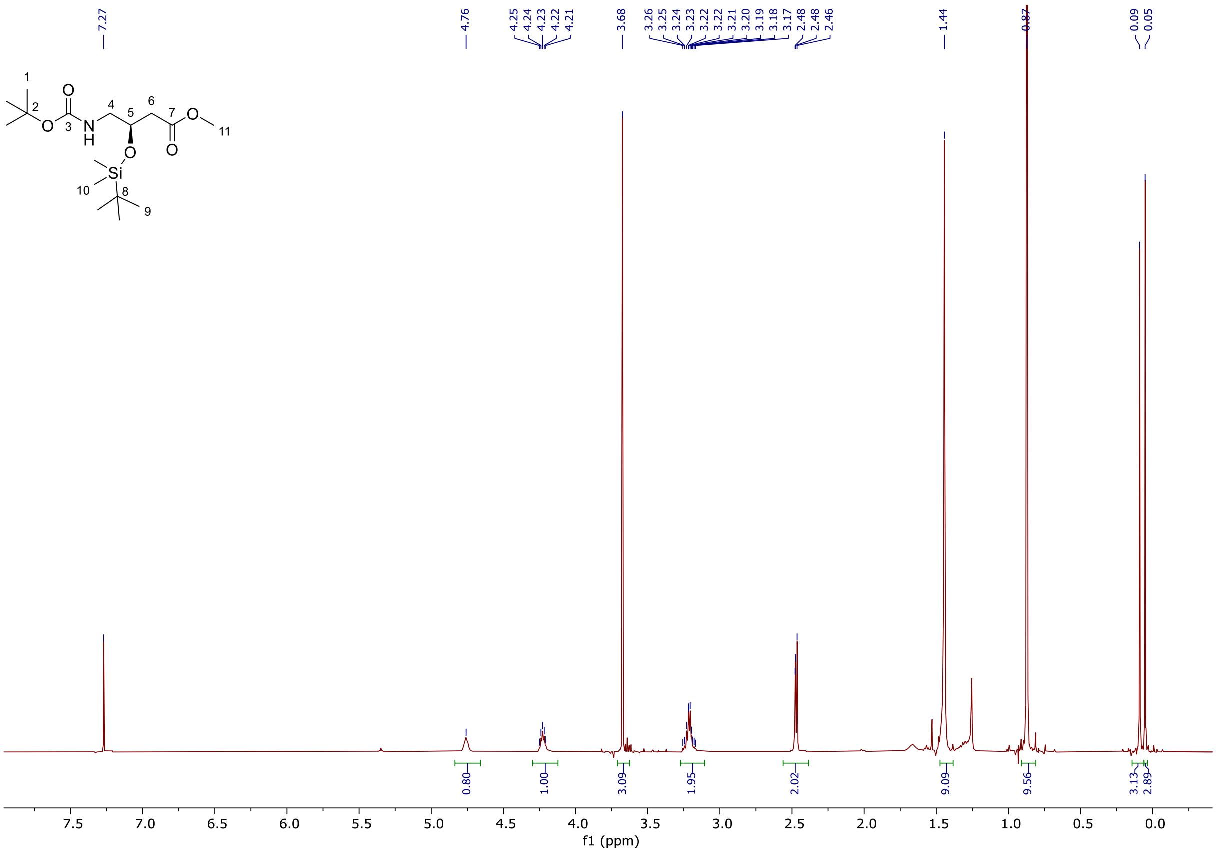

^{13}C-NMR (125 MHz, $CDCl_3$):

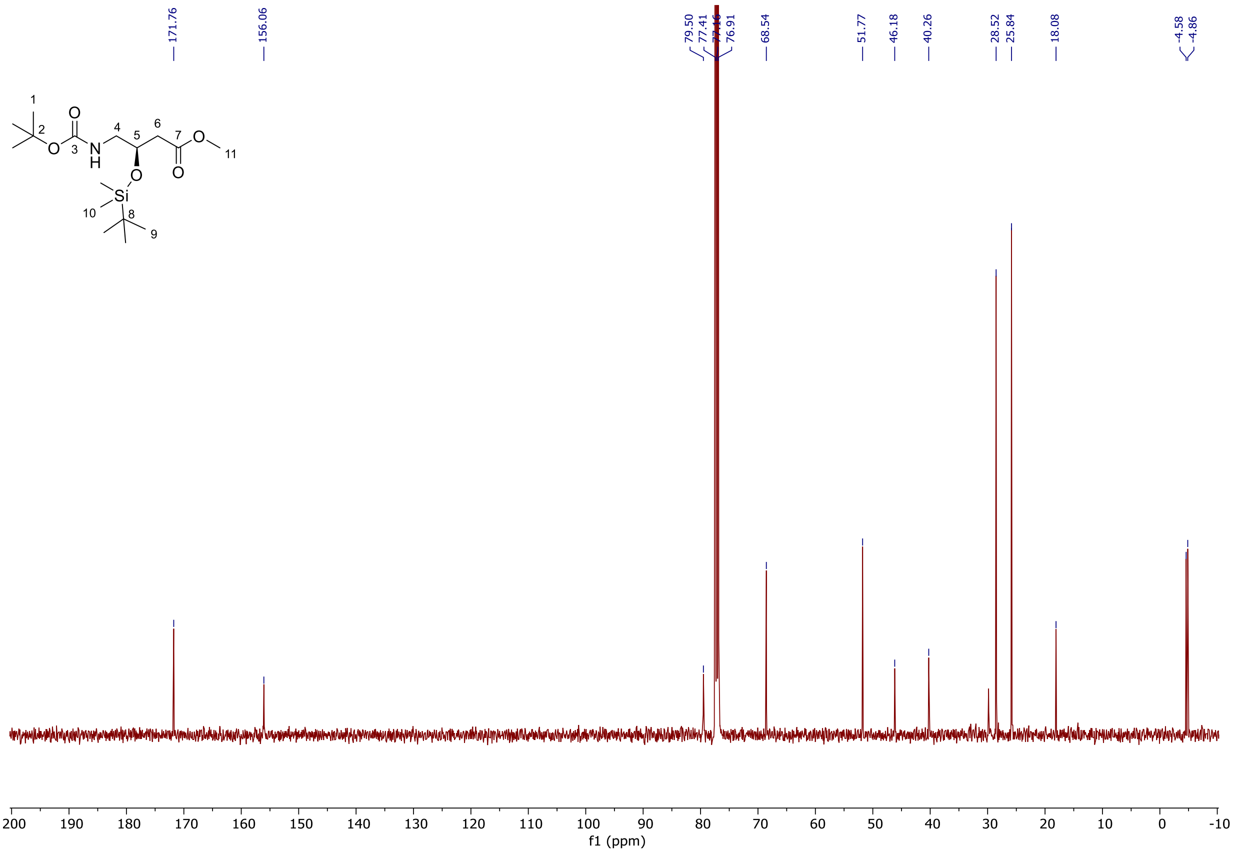

(((3a*S*,5*R*,6*S*,6a*S*)-6-Azido-2,2-dimethyltetrahydrofuro[2,3-d][1,3]dioxol-5-yl)methoxy)(*tert*-butyl)dimethylsilane 11a

^{1}H-NMR (400 MHz, $CDCl_3$):

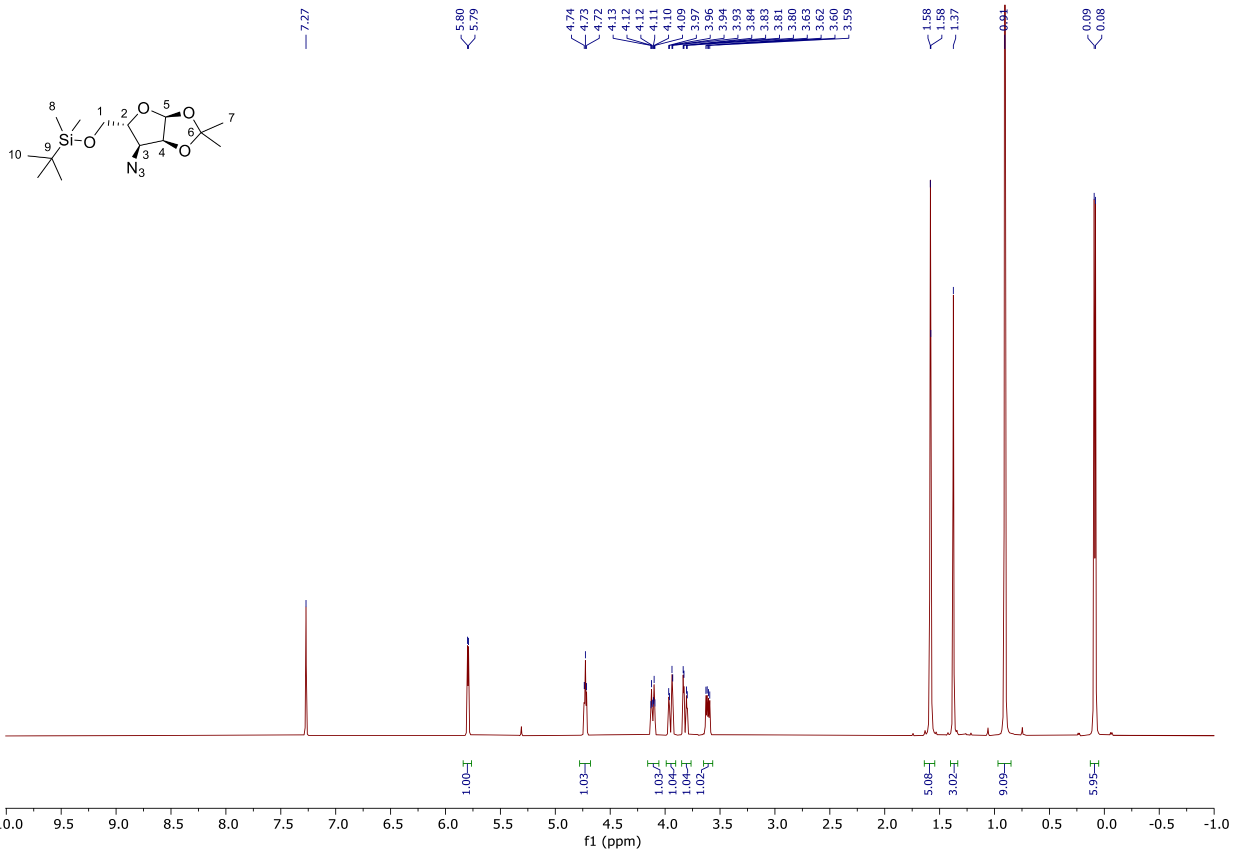

^{13}C-NMR (100 MHz, $CDCl_3$):

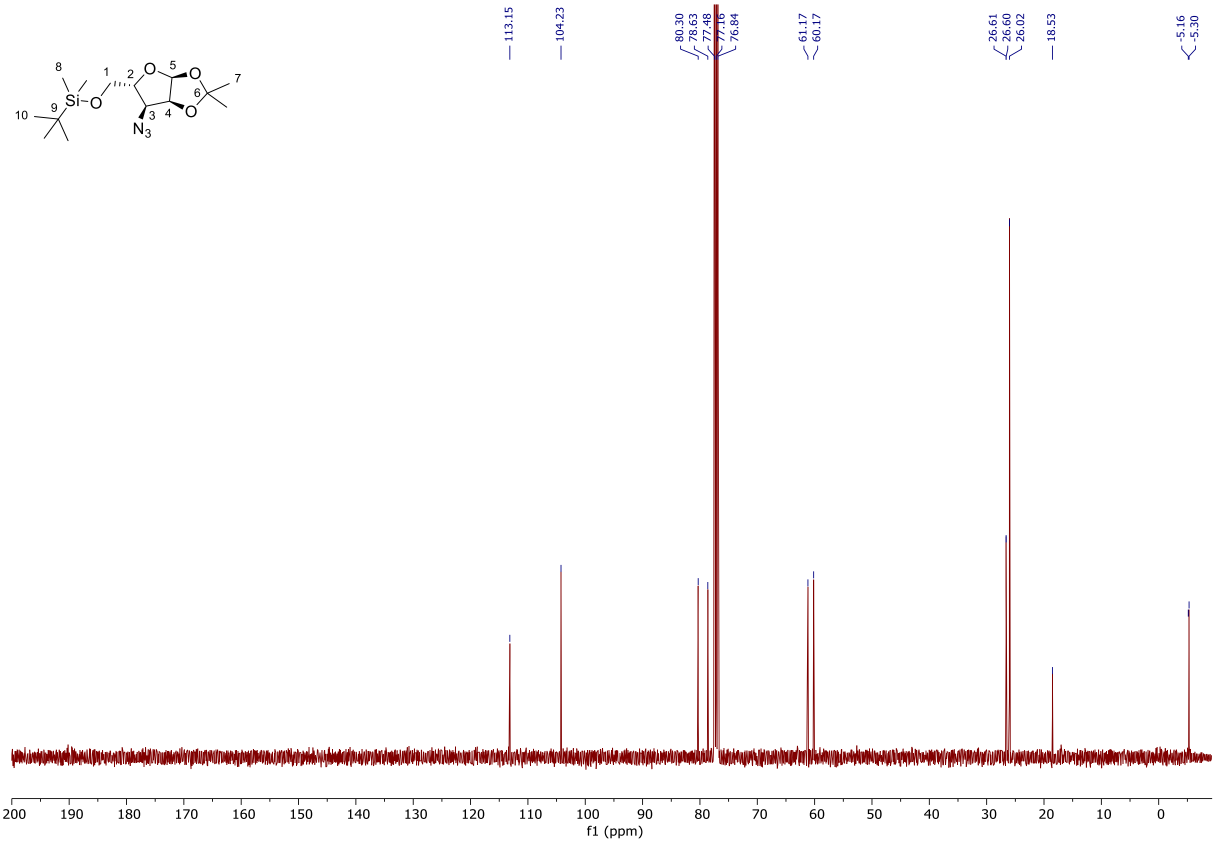

(((3a*S*,5*S*,6*S*,6a*S*)-6-Azido-2,2-dimethyltetrahydrofuro[2,3-d][1,3]dioxol-5-yl)methoxy)(*tert*-butyl)dimethylsilane 11b

^{1}H-NMR (500 MHz, $CDCl_3$):

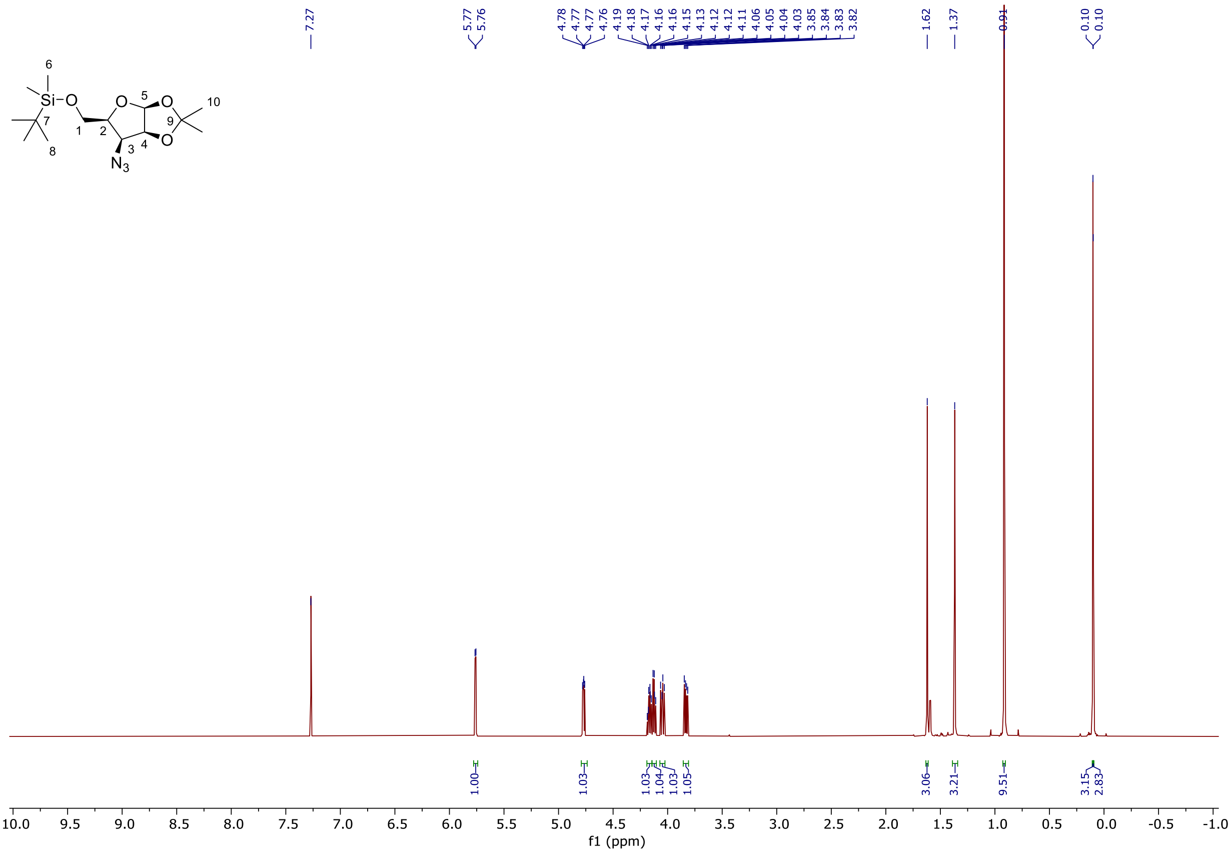

^{13}C-NMR (125 MHz, $CDCl_3$):

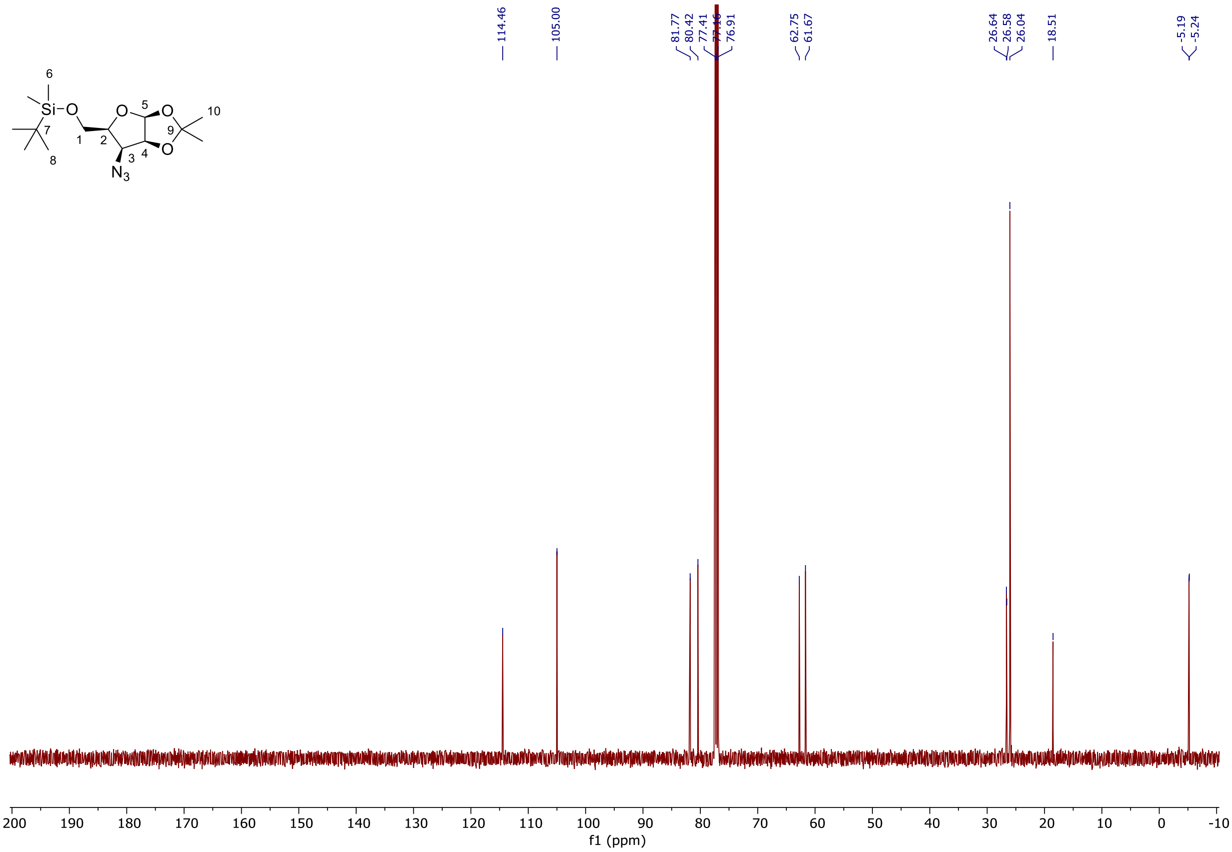

(2*R*,3*S*,4*R*,5*R*)-2-Allyl-4-azido-5-(((*tert*-butyldimethylsilyl)oxy)methyl)tetrahydrofuran-3-ol 12a

[1]**H-NMR** (400 MHz, DMSO-d$_6$):

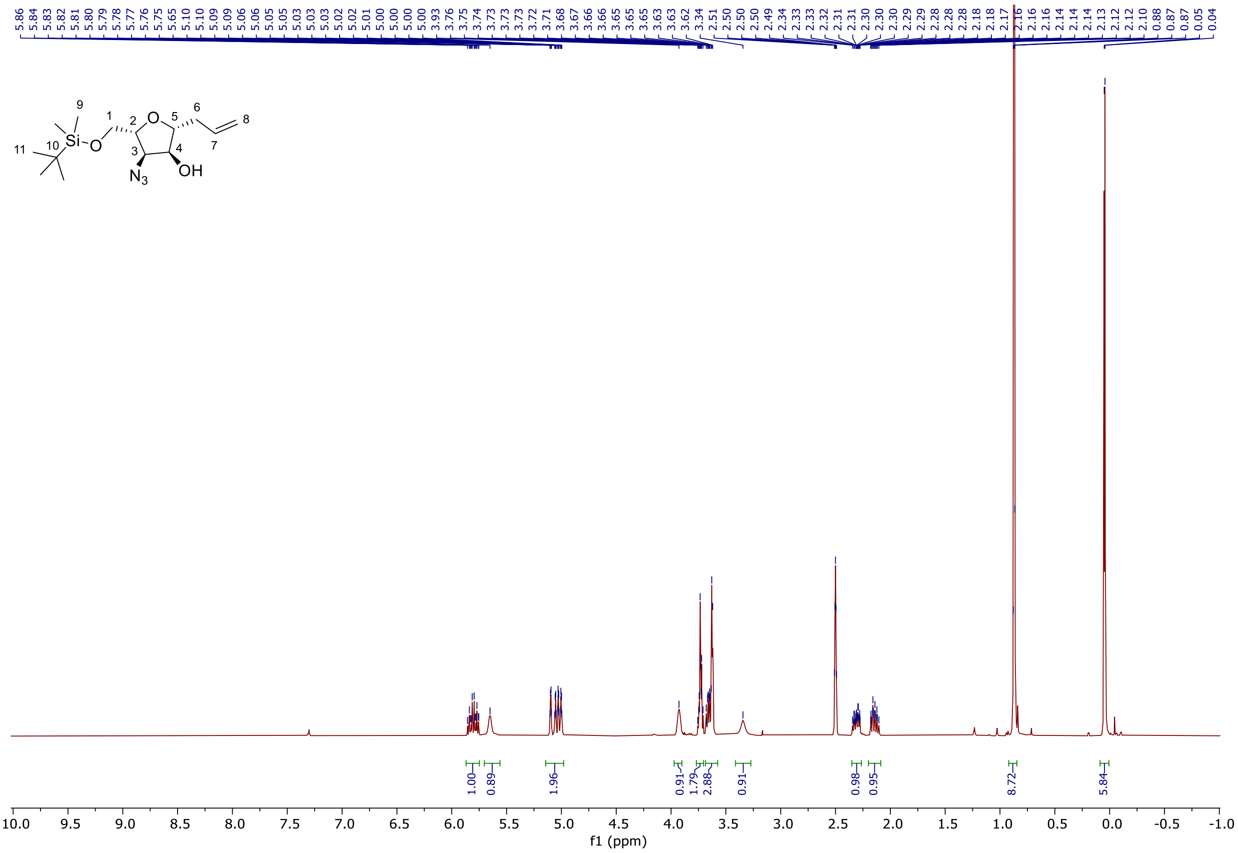

[13]**C-NMR** (100 MHz, DMSO-d$_6$):

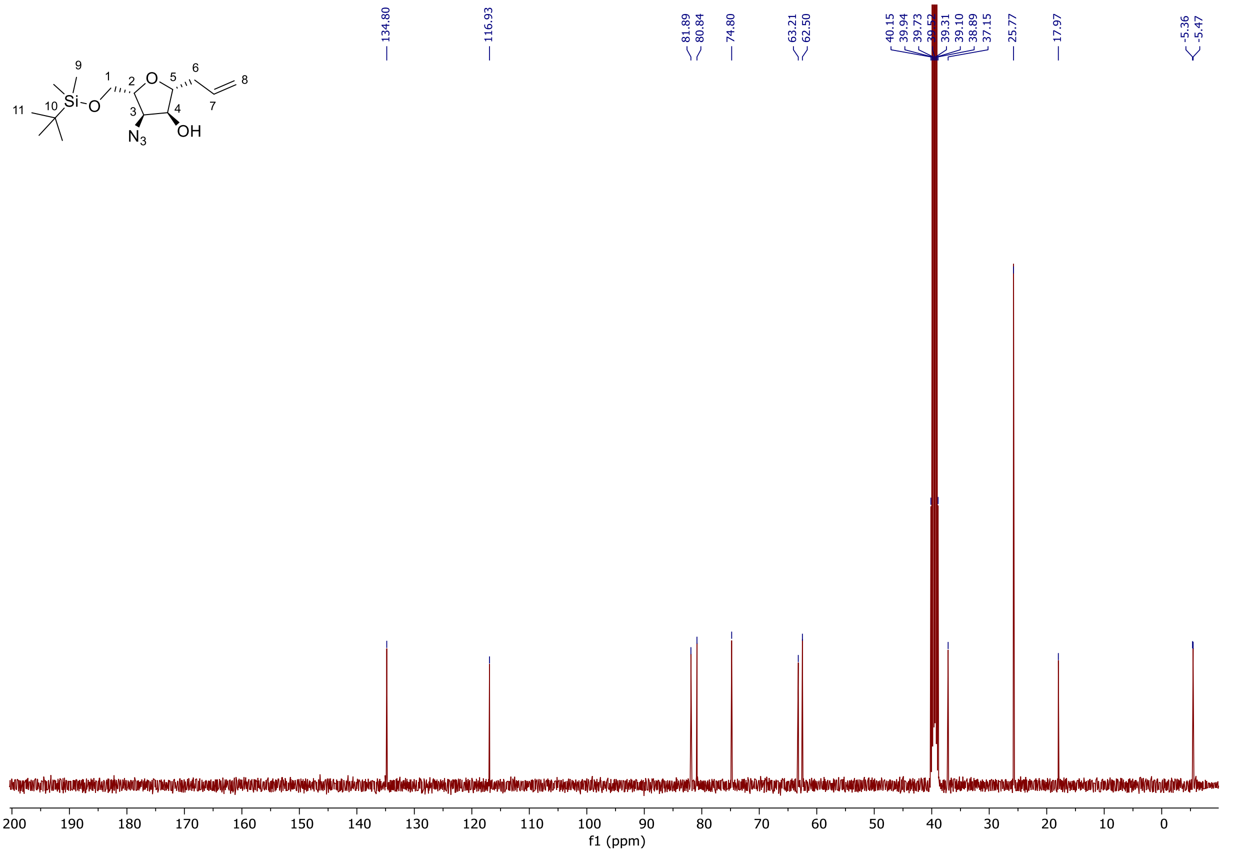

(2*S*,3*S*,4*R*,5*R*)-2-Allyl-4-azido-5-(((*tert*-butyldimethylsilyl)oxy)methyl)tetrahydrofuran-3-ol 12b

^{1}H-NMR (500 MHz, DMSO-d$_6$):

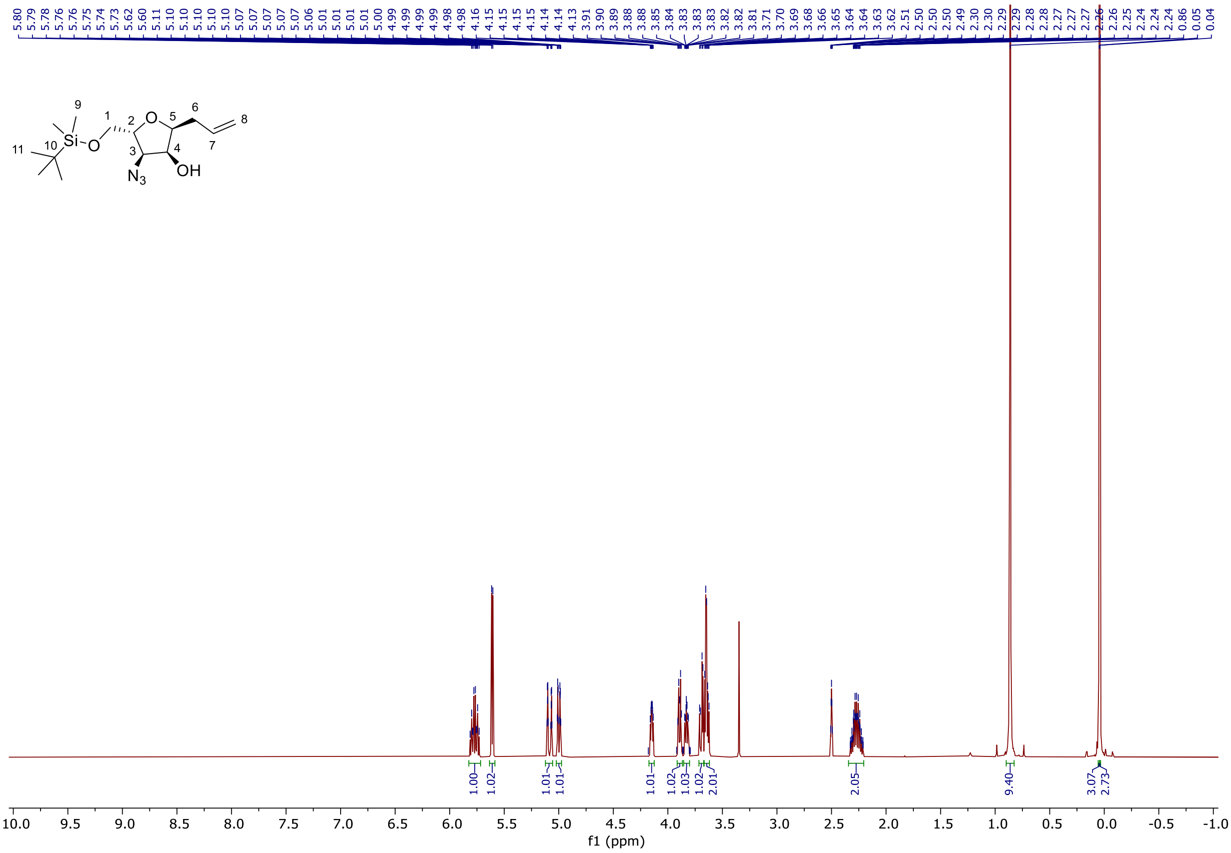

^{13}C-NMR (125 MHz, DMSO-d$_6$):

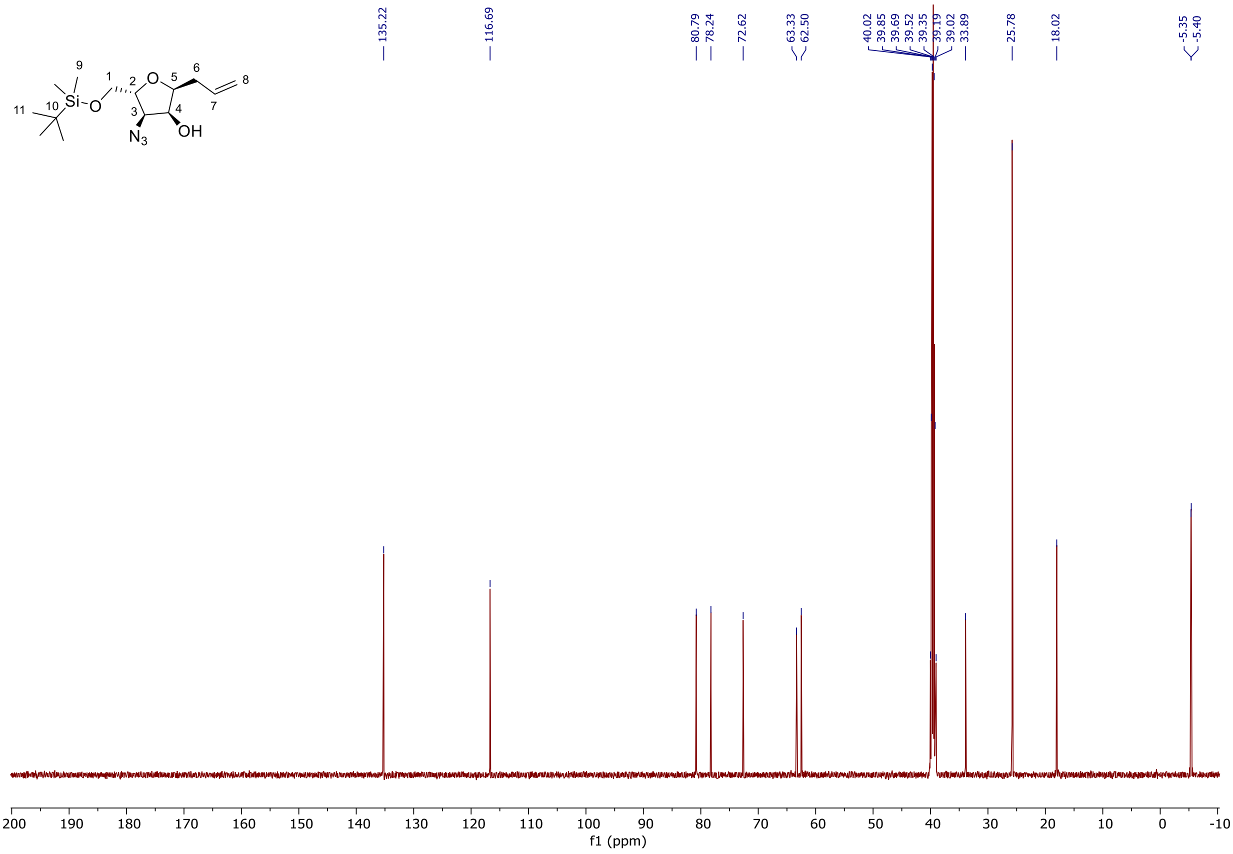

(2*R*,3*S*,4*R*,5*S*)-2-Allyl-4-azido-5-(((*tert*-butyldimethylsilyl)oxy)methyl)tetrahydrofuran-3-ol 12c

^{1}H-NMR (500 MHz, DMSO-d$_6$): 1wt% EtOAc as impurity

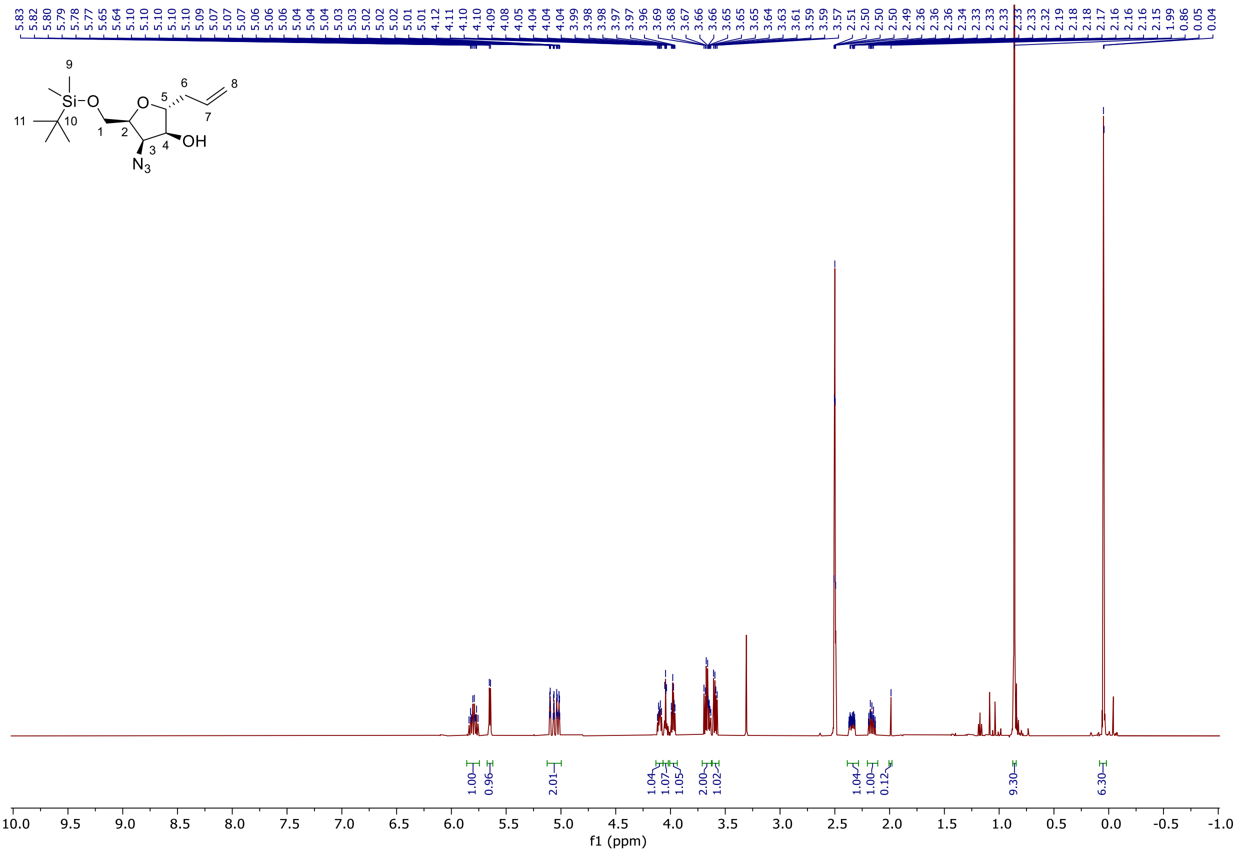

^{13}C-NMR (125 MHz, DMSO-d$_6$):

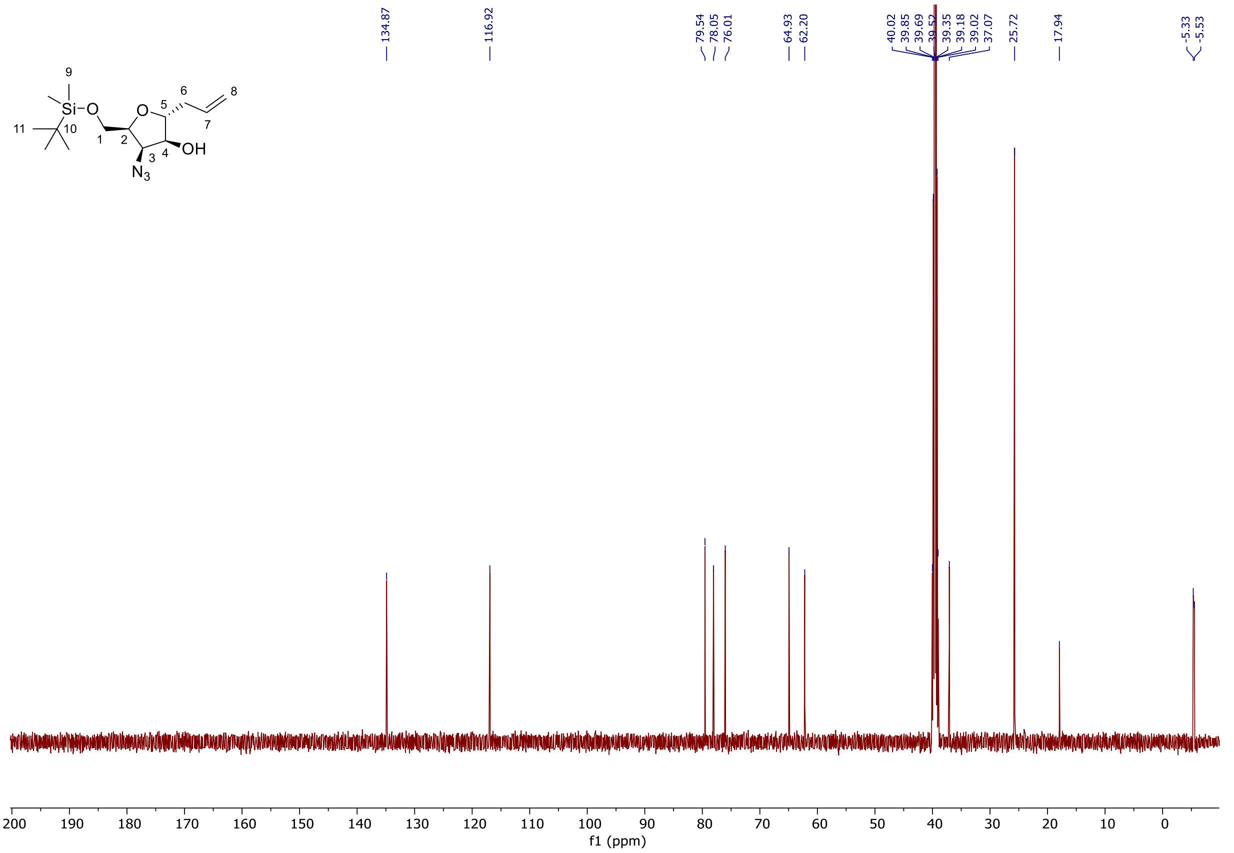

(2*S*,3*S*,4*R*,5*S*)-2-Allyl-4-azido-5-(((*tert*-butyldimethylsilyl)oxy)methyl)tetrahydrofuran-3-ol 12d

^{1}H-NMR (500 MHz, $CDCl_3$):

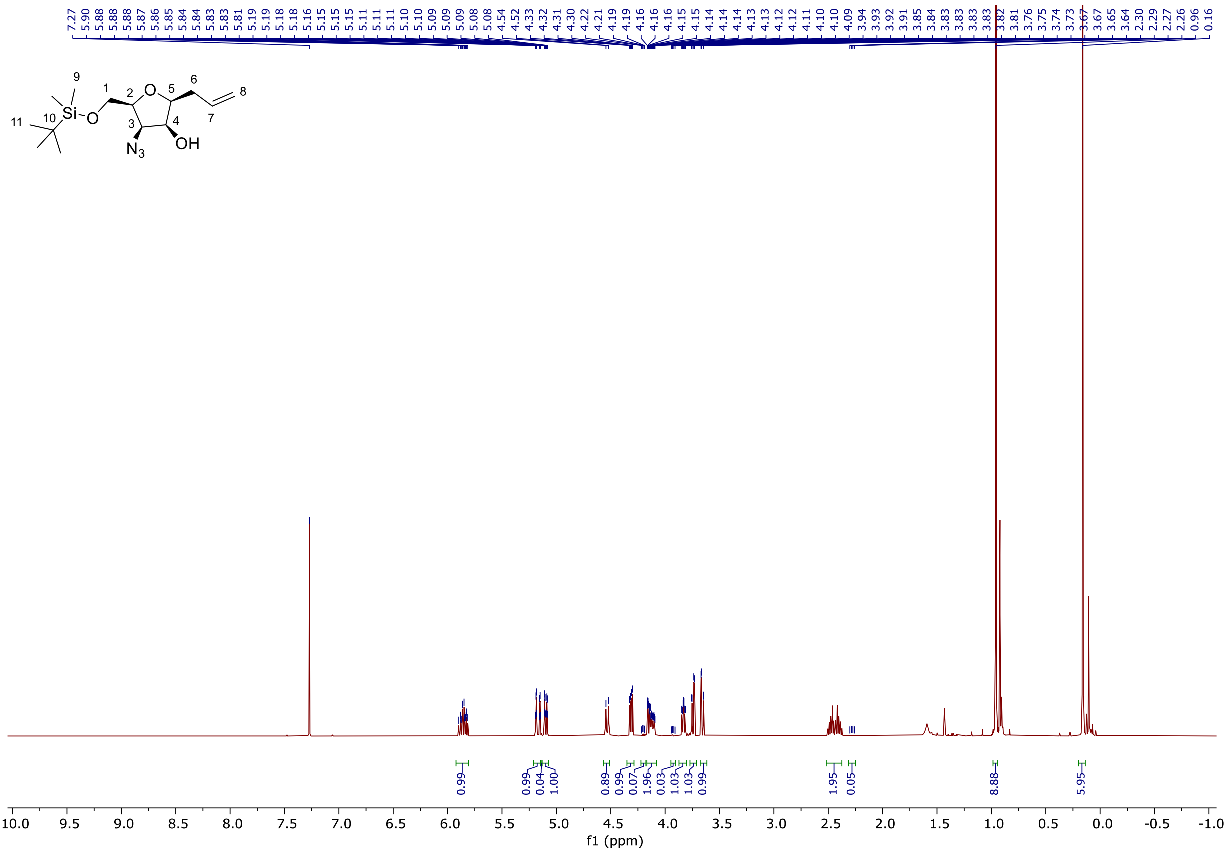

^{13}C-NMR (125 MHz, $CDCl_3$):

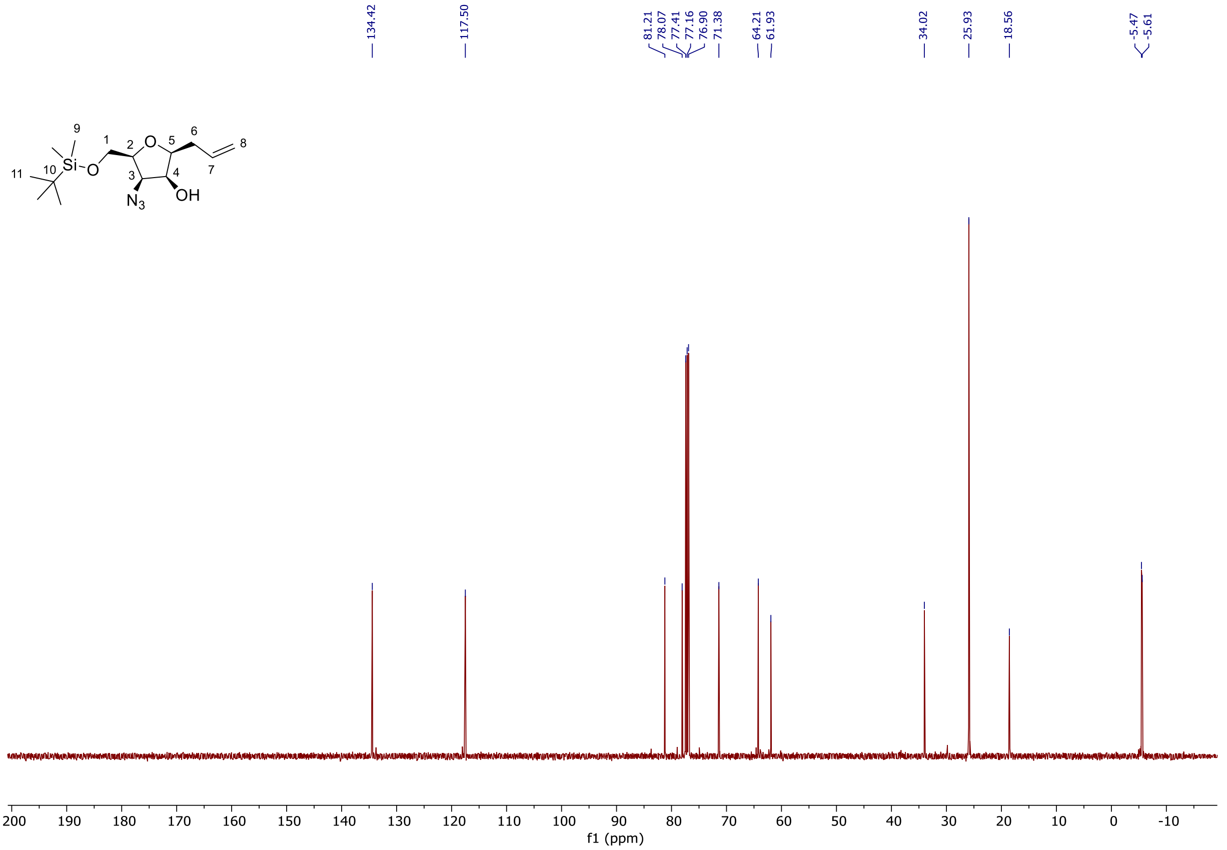

2-((2*R*,3*S*,4*R*,5*R*)-4-Azido-5-(((*tert*-butyldimethylsilyl)oxy)methyl)-3-hydroxytetrahydrofuran-2-yl)acetaldehyde SI-2

^{1}H-NMR (400 MHz, DMSO-d$_6$):

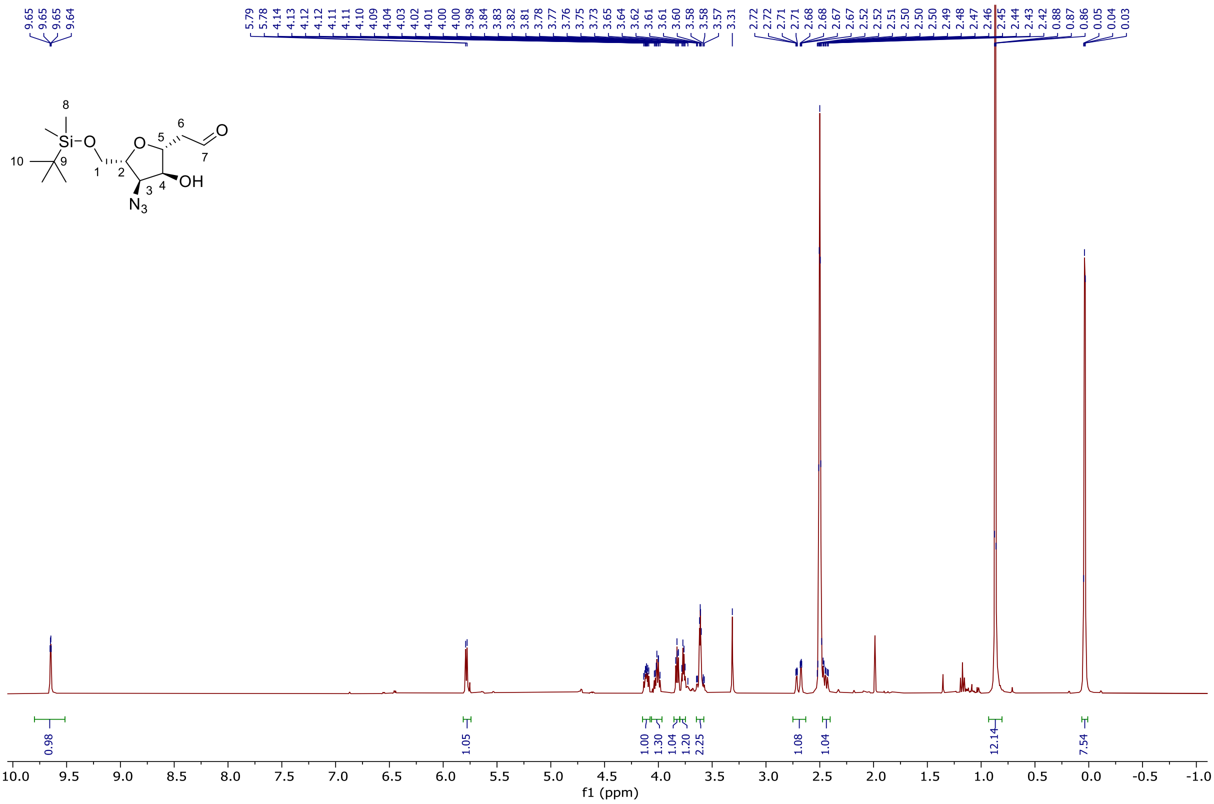

^{13}C-NMR (100 MHz, DMSO-d$_6$):

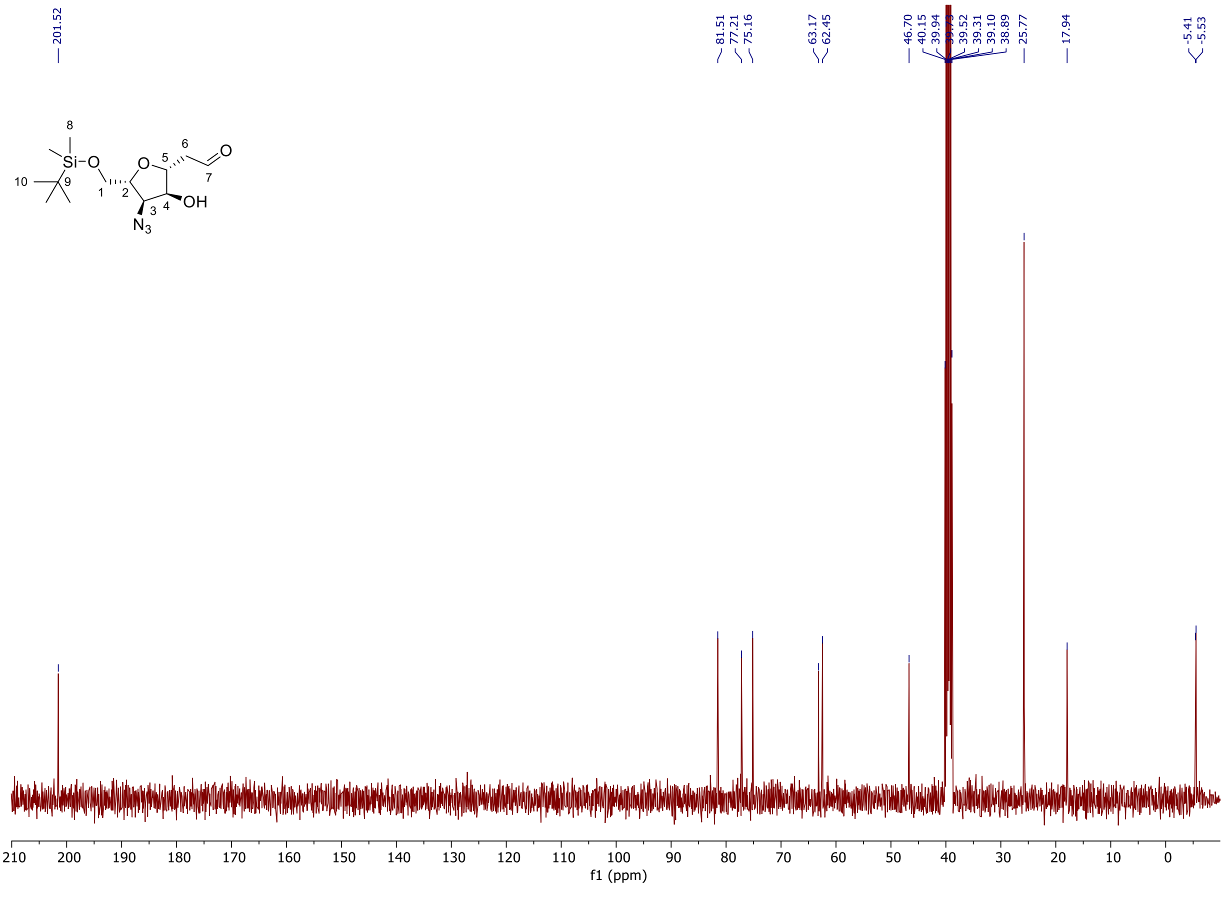

(3a*S*,5*R*,6*S*,6a*S*)-6-Azido-5-(((*tert*-butyldimethylsilyl)oxy)methyl)tetrahydrofuro[3,2-b]furan-2(3*H*)-one SI-3

^{1}H-NMR (400 MHz, $CDCl_3$):

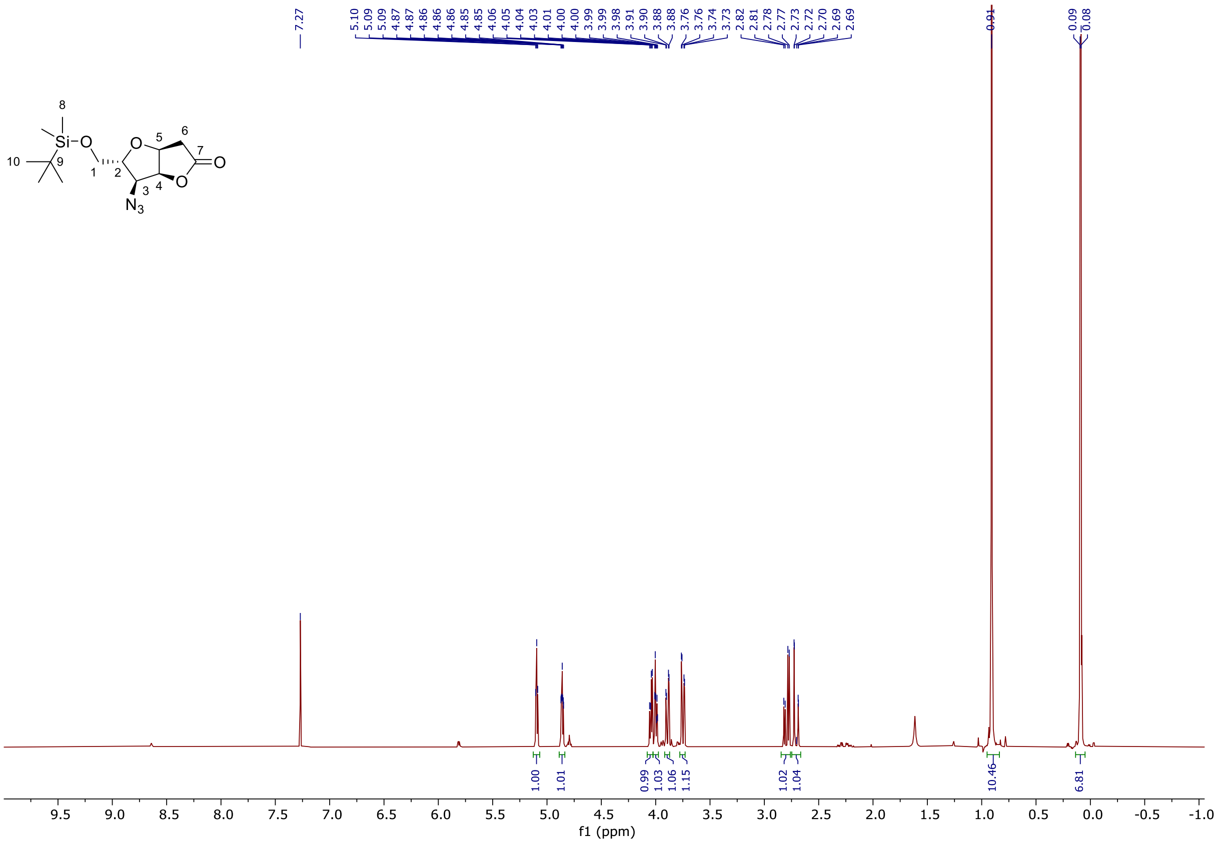

^{13}C-NMR (100 MHz, $CDCl_3$):

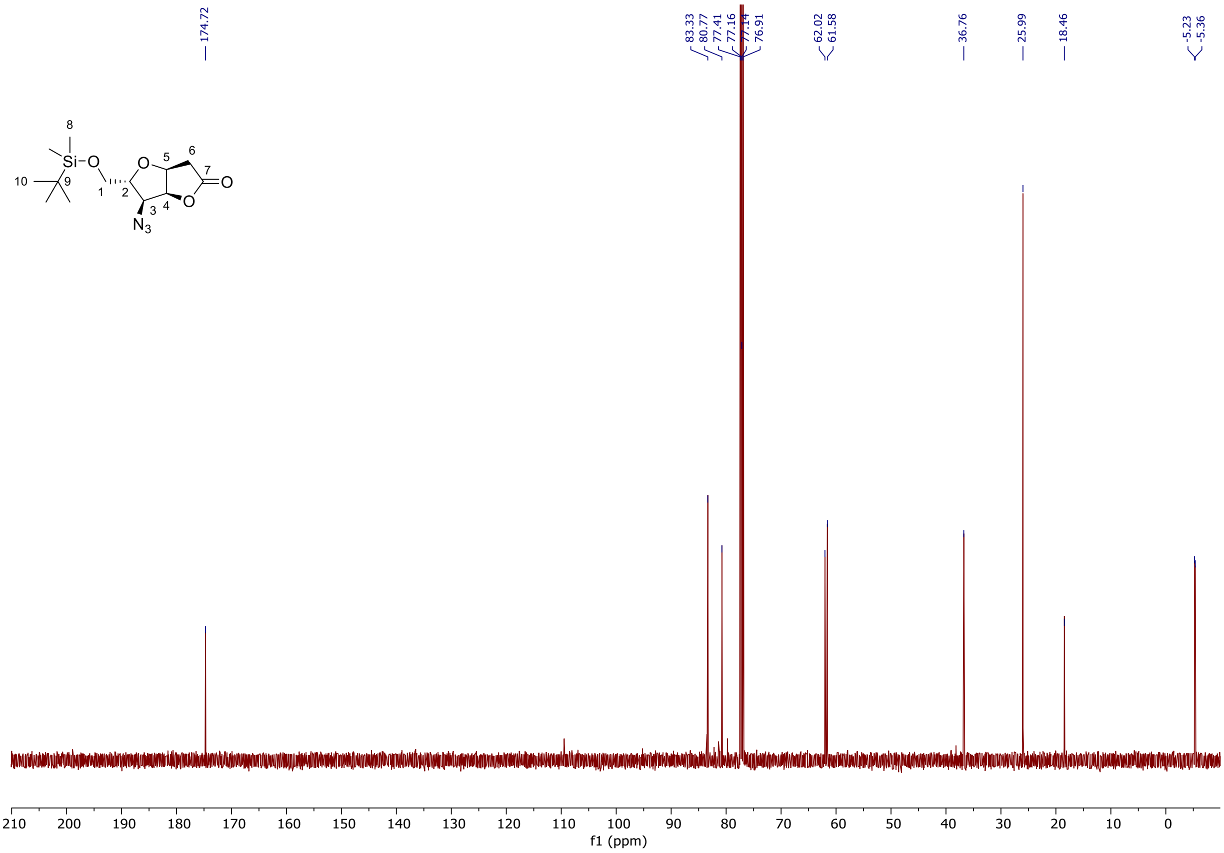

2-((2*R*,3*S*,4*R*,5*S*)-4-Azido-5-(((*tert*-butyldimethylsilyl)oxy)methyl)-3-hydroxytetrahydrofuran-2-yl)acetaldehyde SI-4

^{1}H-NMR (500 MHz, $CDCl_3$):

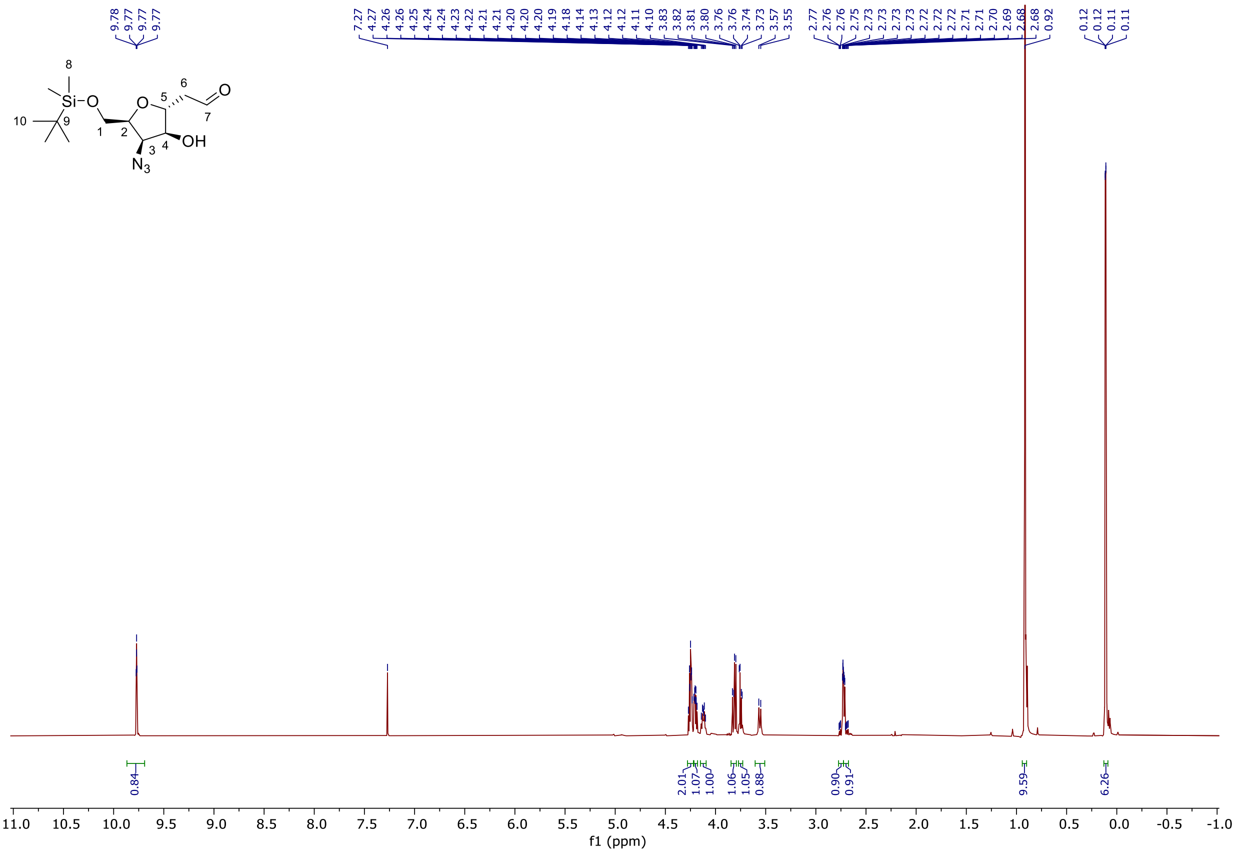

^{13}C-NMR (125 MHz, $CDCl_3$):

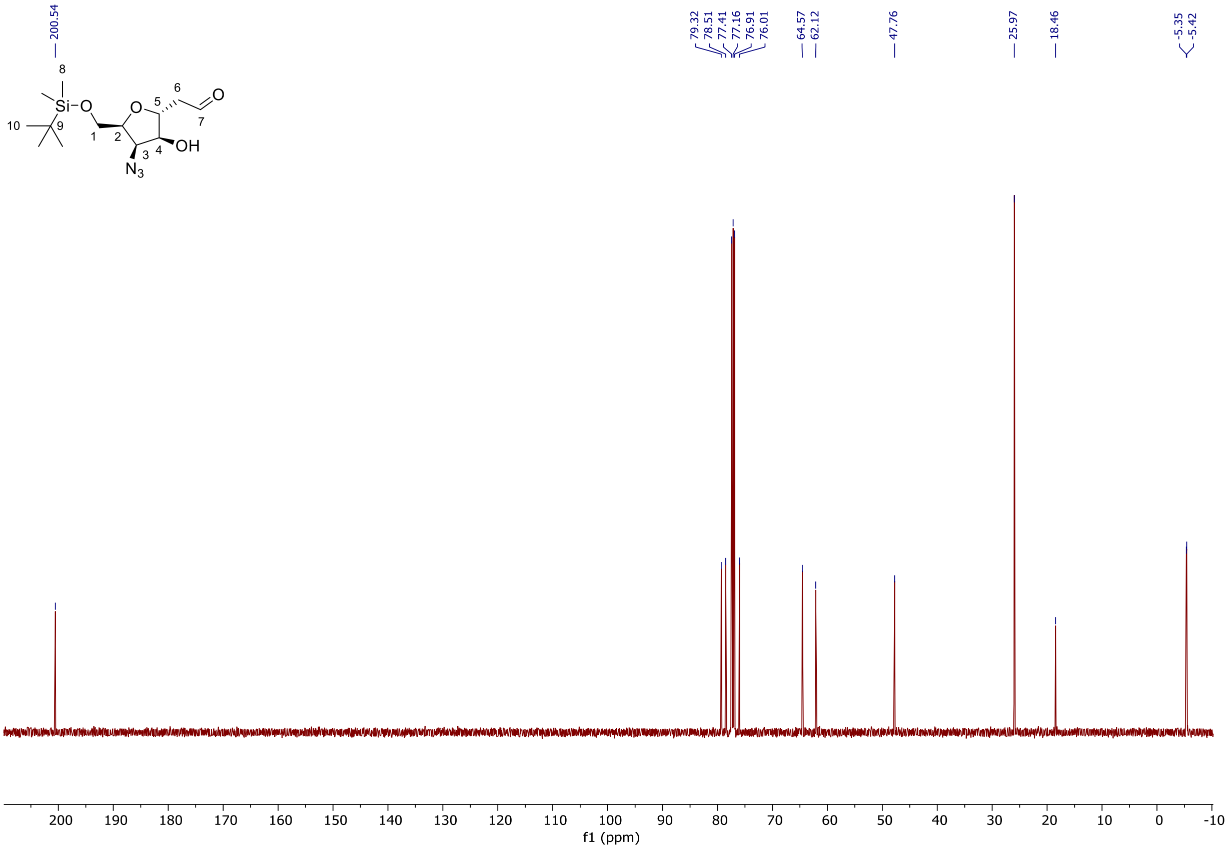

(3a*S*,5*S*,6*S*,6a*S*)-6-Azido-5-(((*tert*-butyldimethylsilyl)oxy)methyl)tetrahydrofuro[3,2-b]furan-2(3*H*)-one SI-5

^{1}H-NMR (500 MHz, $CDCl_3$):

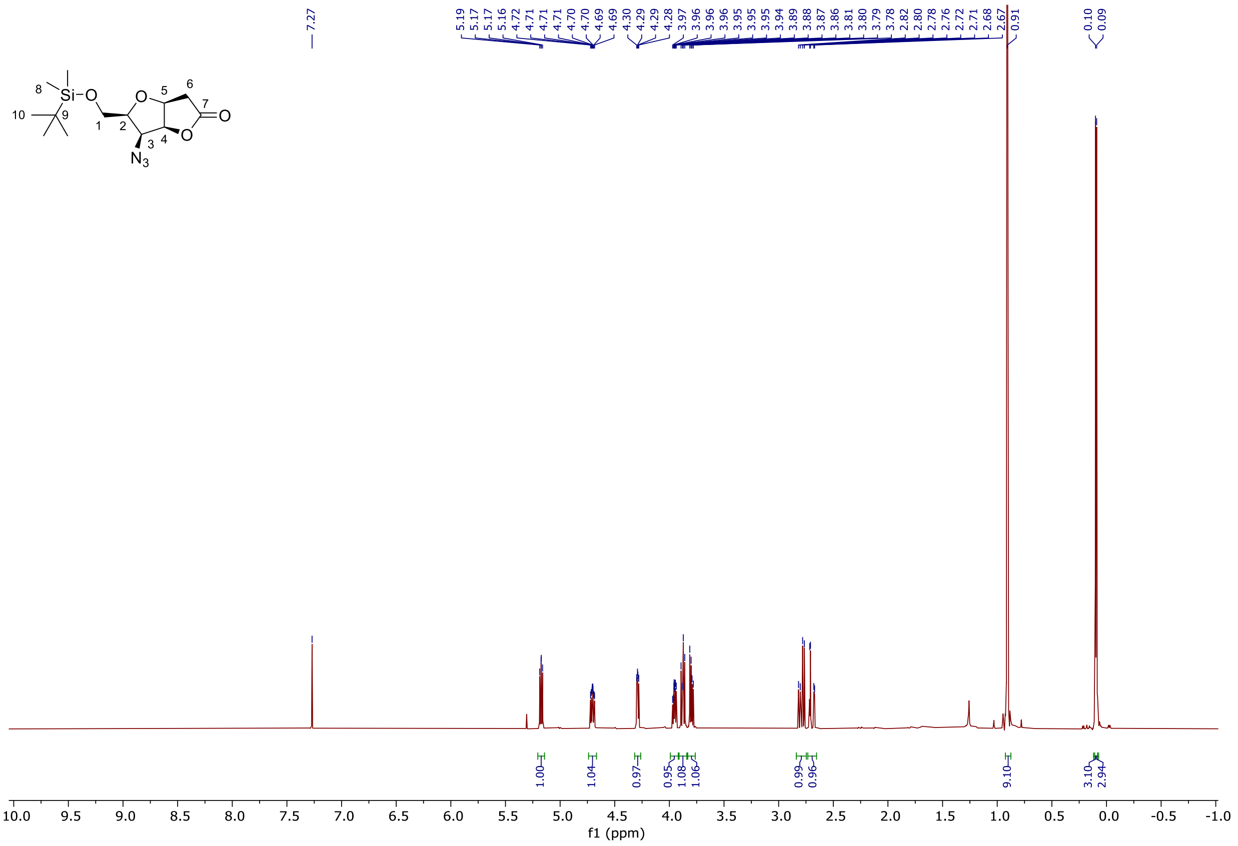

^{13}C-NMR (125 MHz, $CDCl_3$):

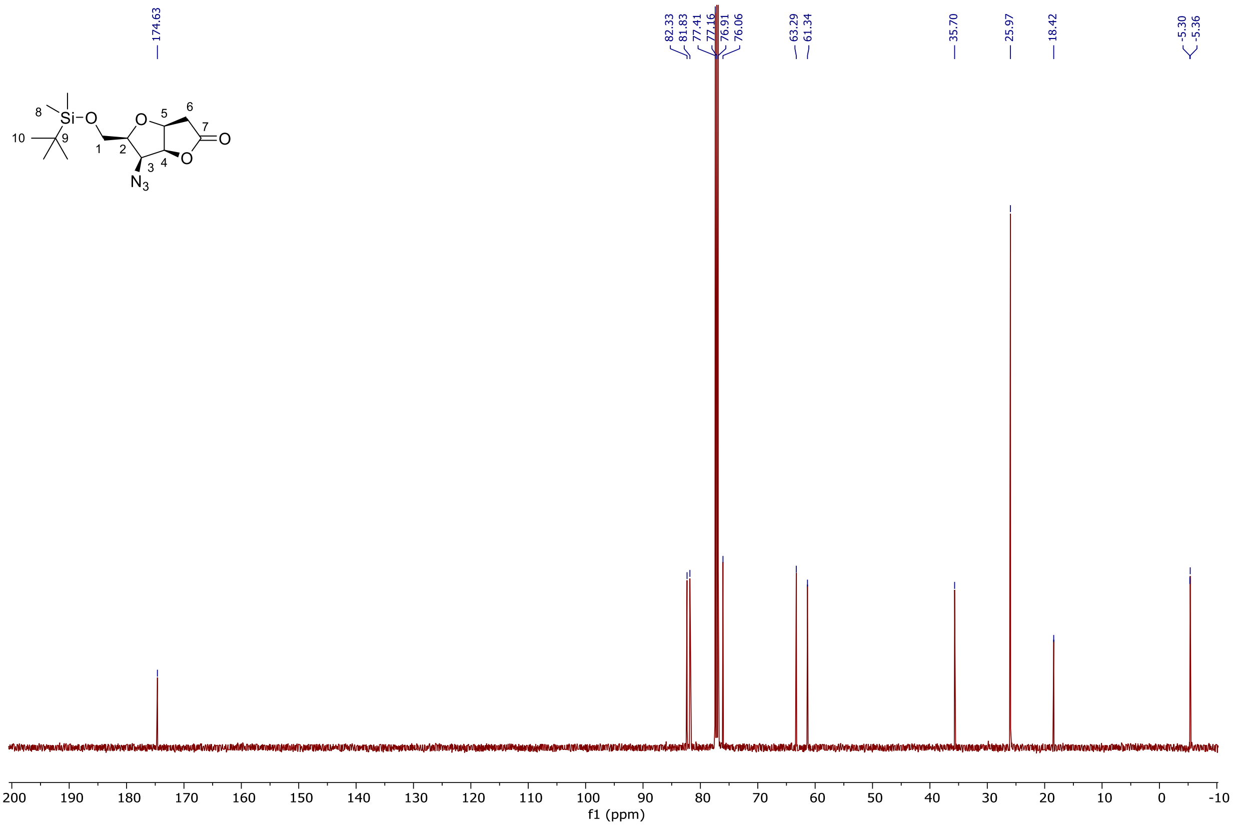

(((2*R*,3*S*,4*S*,5*R*)-5-Allyl-3-azido-4-((*tert*-butyldimethylsilyl)oxy)tetrahydrofuran-2-yl)methoxy)-(*tert*-butyl)dimethylsilane 13a

^{1}H-NMR (500 MHz, $CDCl_3$):

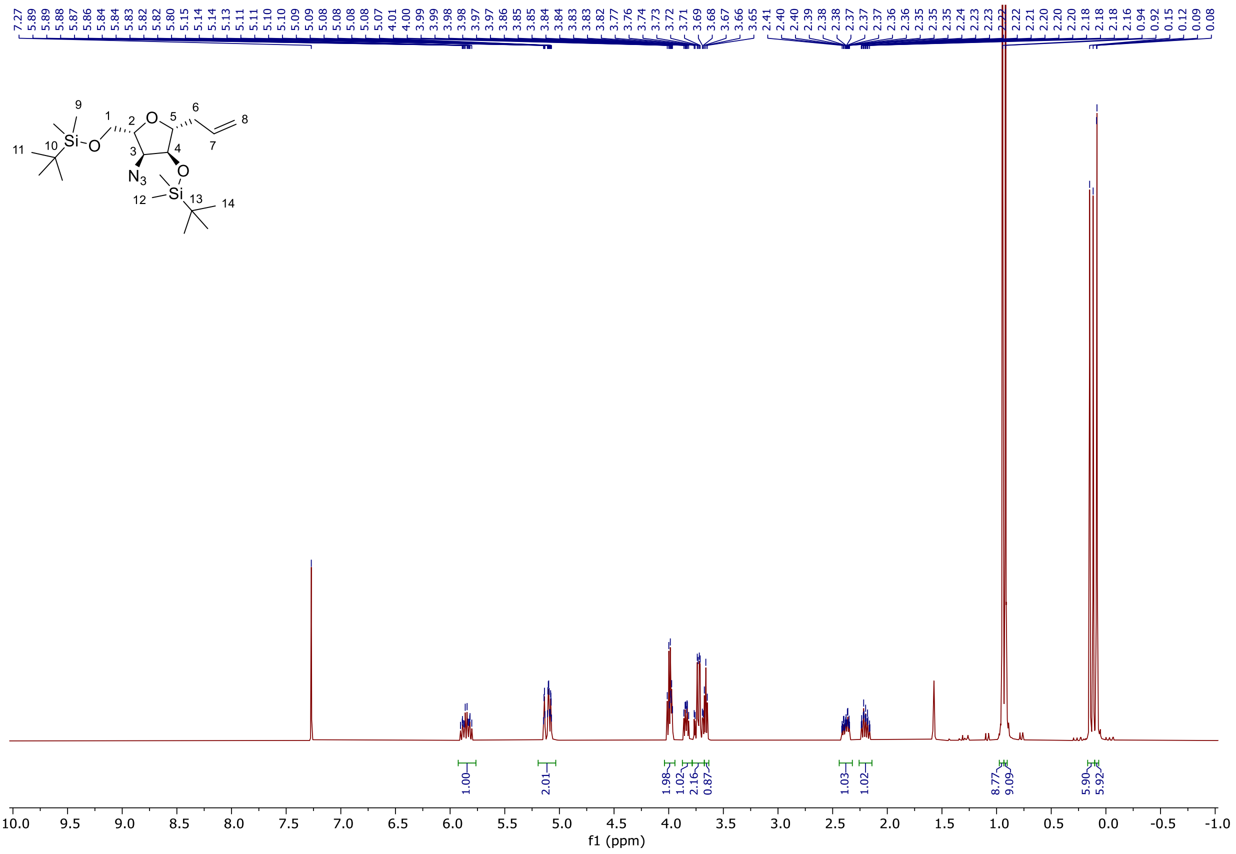

^{13}C-NMR (125 MHz, $CDCl_3$):

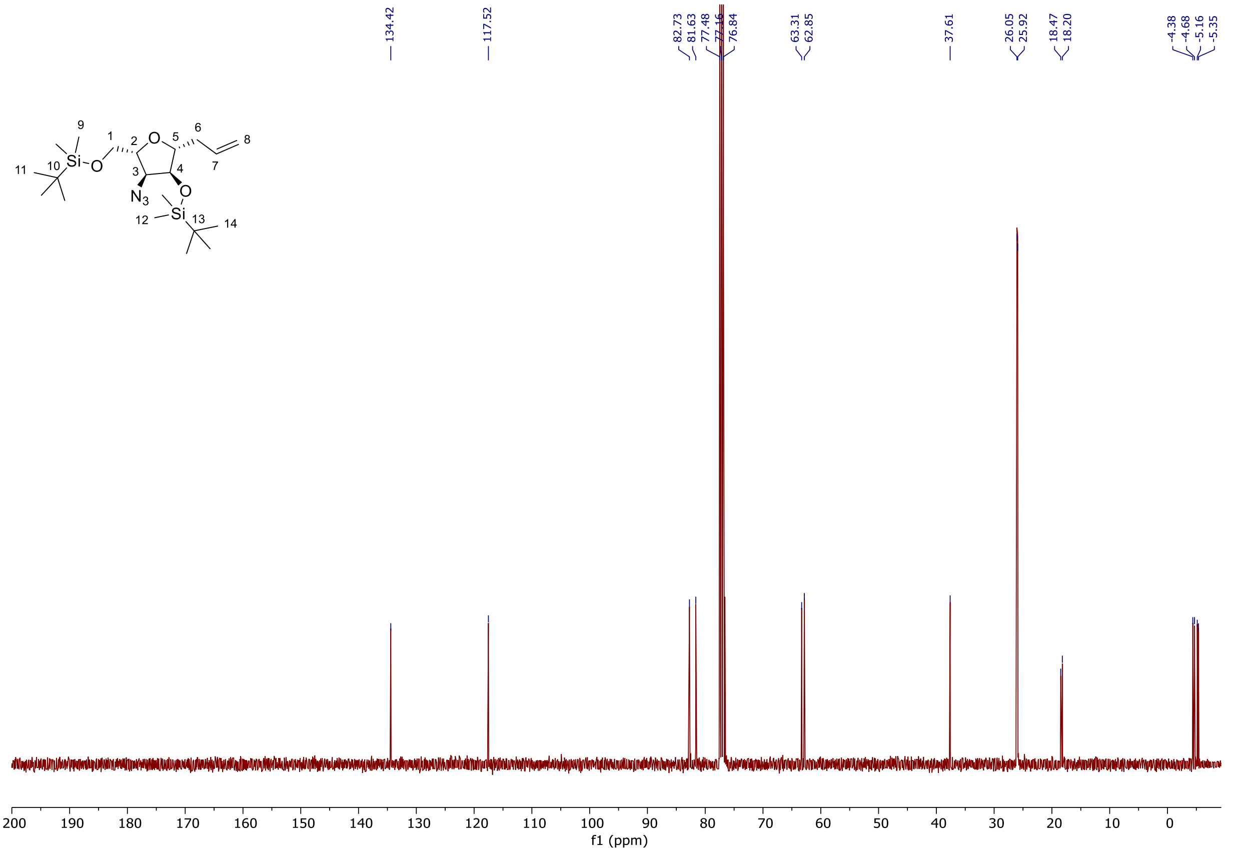

(((2*S*,3*S*,4*S*,5*R*)-5-Allyl-3-azido-4-((*tert*-butyldimethylsilyl)oxy)tetrahydrofuran-2-yl)methoxy) (*tert*-butyl)dimethylsilane 13c

^{1}H-NMR (500 MHz, $CDCl_3$):

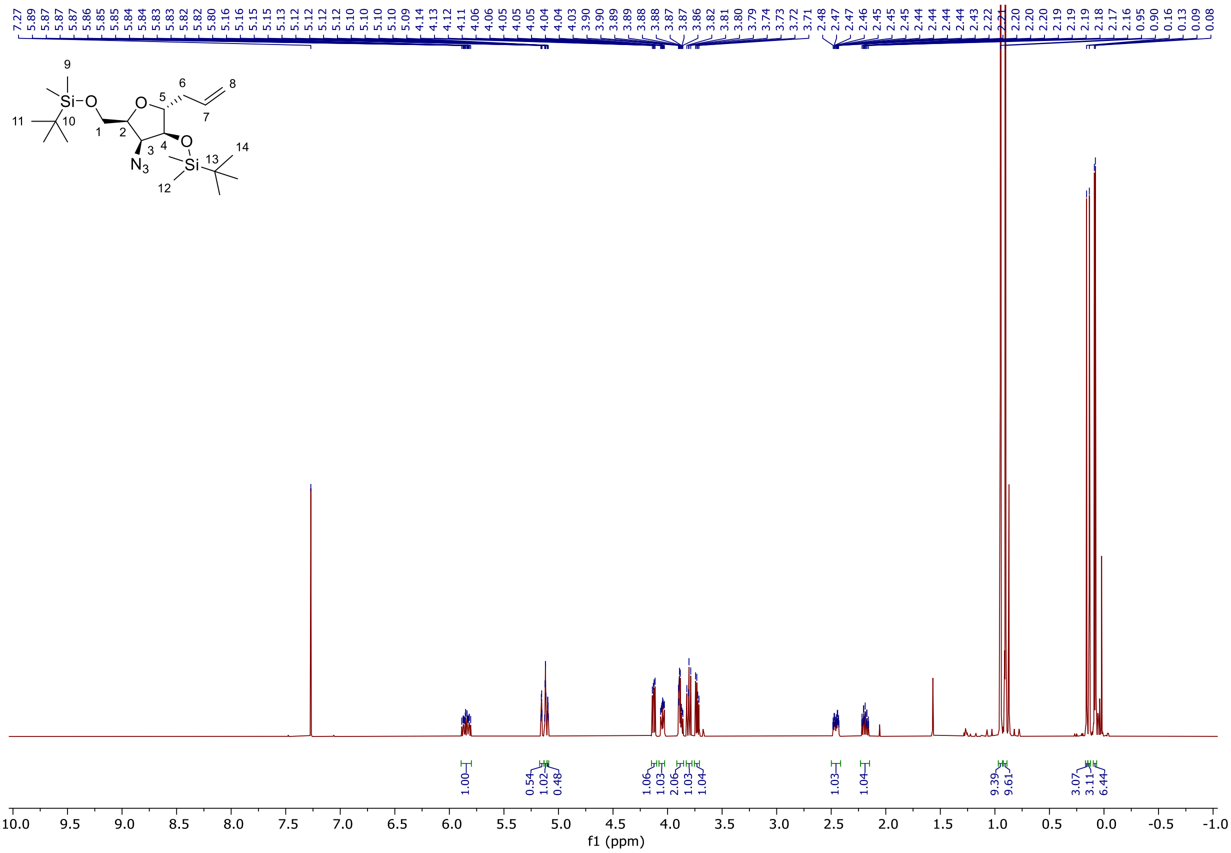

^{13}C-NMR (125 MHz, $CDCl_3$):

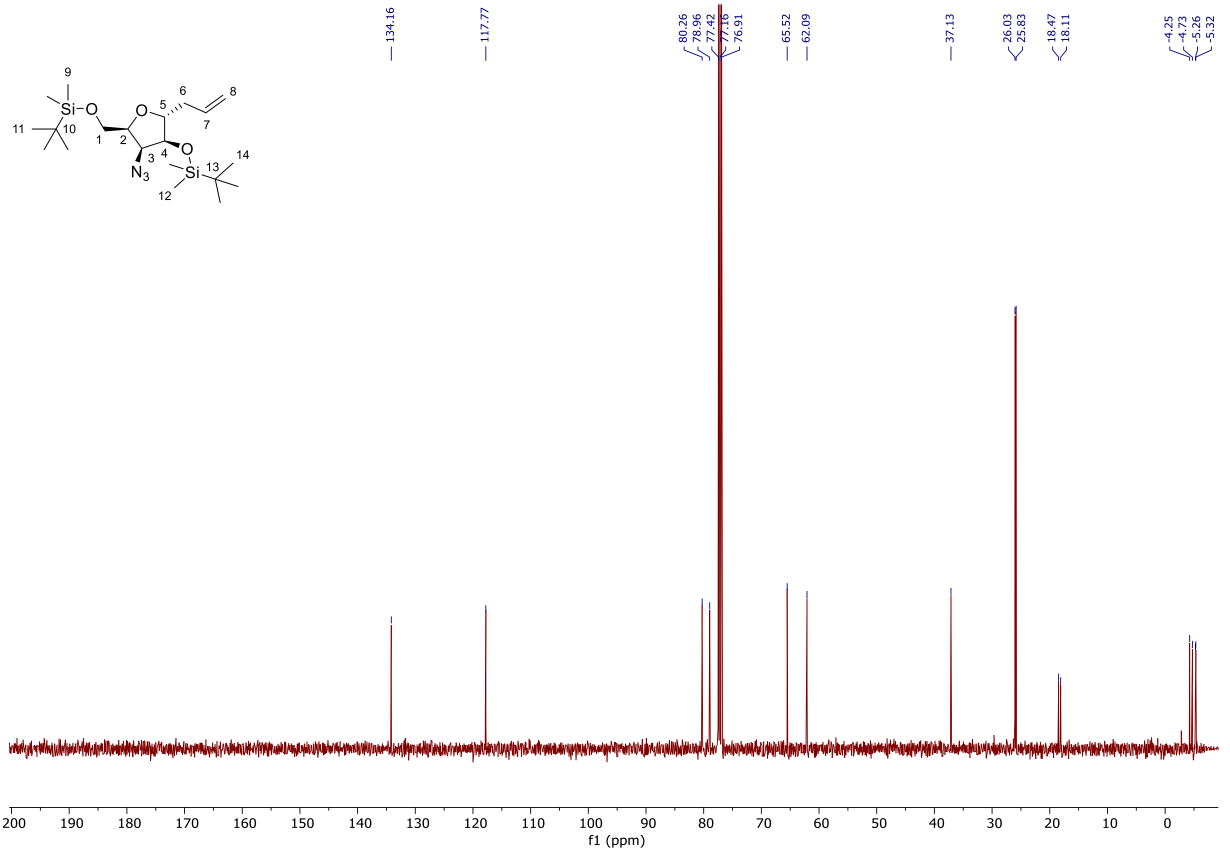

((2*R*,3*S*,4*S*,5*R*)-Allyl-3-azido-4-((*tert*-butyldimethylsilyl)oxy)tetrahydrofuran-2-yl) methanol 14a

^{1}H-NMR (500 MHz, $CDCl_3$):

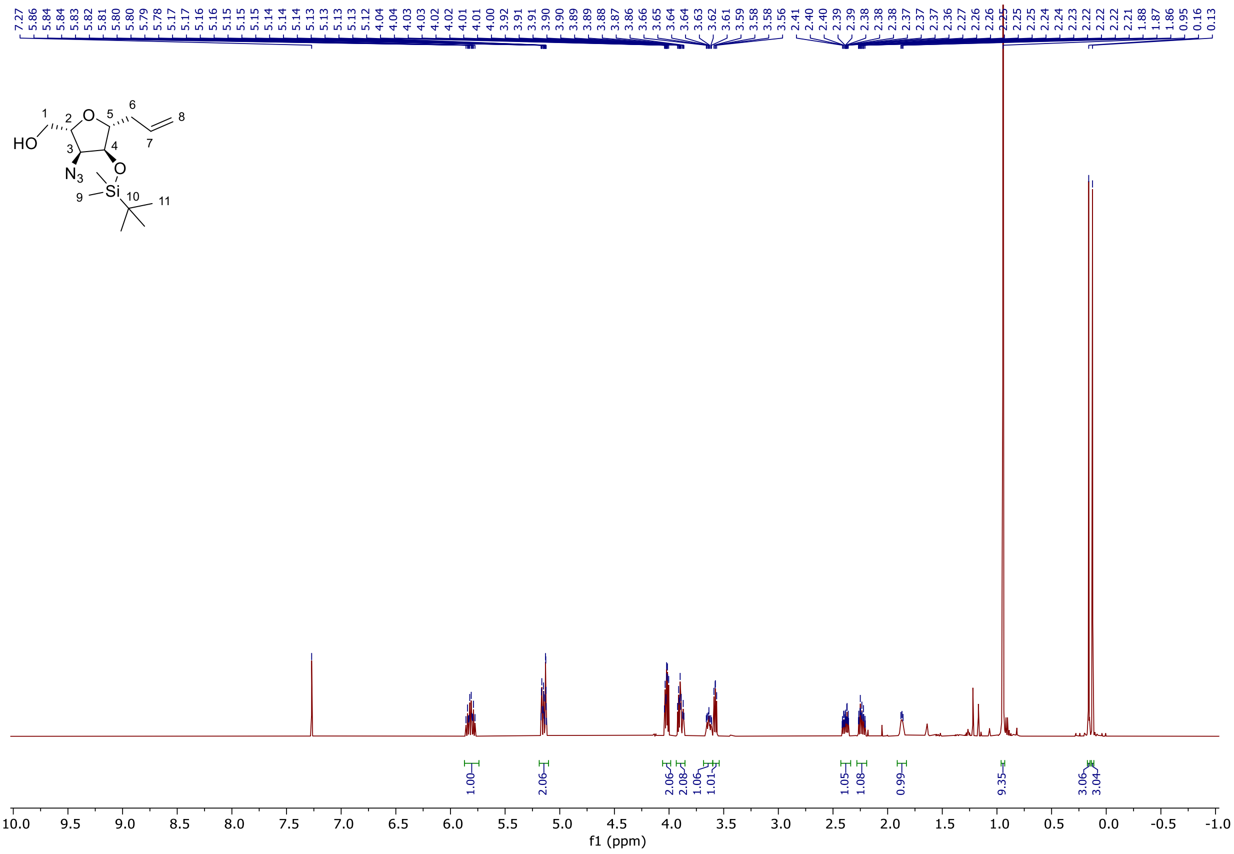

^{13}C-NMR (125 MHz, $CDCl_3$):

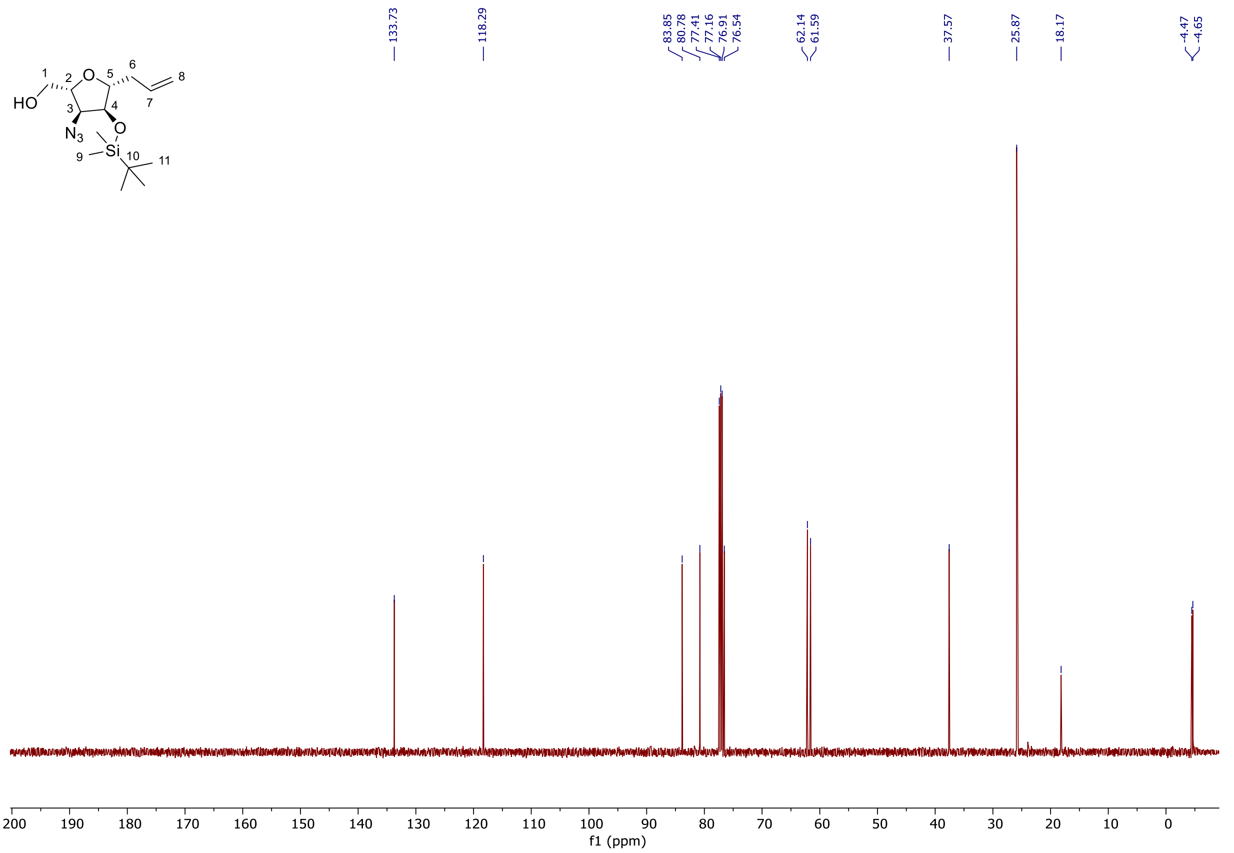

((2*S*,3*S*,4*S*,5*R*)-Allyl-3-azido-4-((*tert*-butyldimethylsilyl)oxy)tetrahydrofuran-2-yl)methanol 14c

^{1}H-NMR (500 MHz, $CDCl_3$): 2wt% EtOAc as impurity

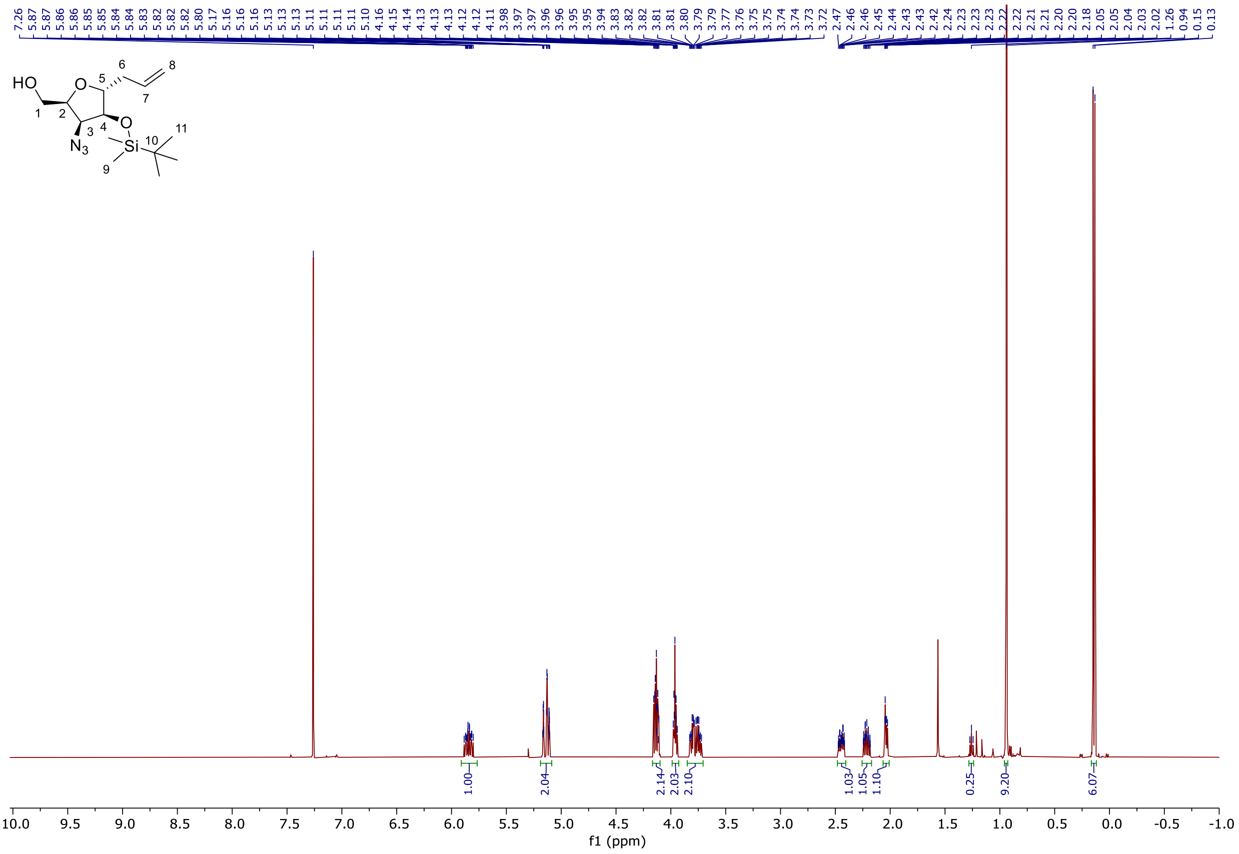

^{13}C-NMR (125 MHz, $CDCl_3$):

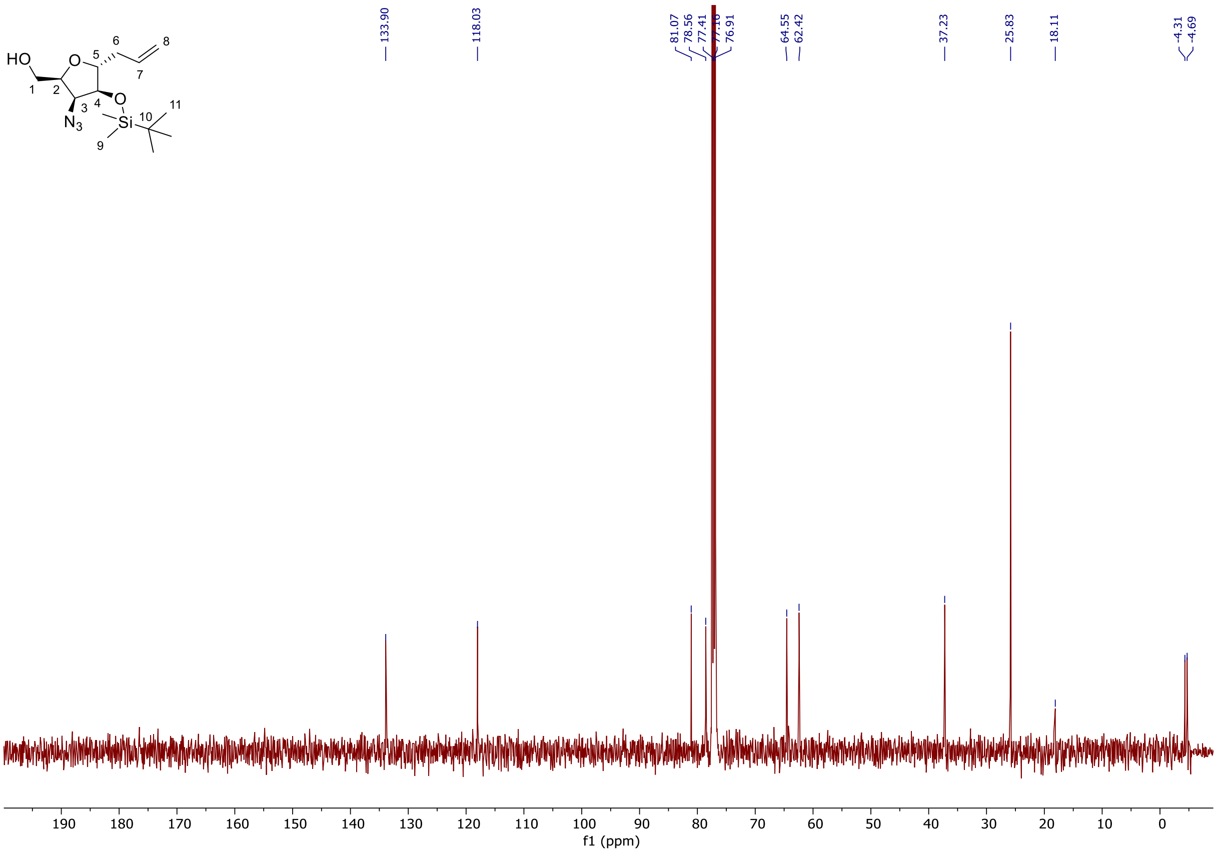

Methyl (2*R*,3*S*,4*S*,5*R*)-5-allyl-3-azido-4-((*tert*-butyldimethylsilyl)oxy)tetrahydrofuran-2-carboxylate 15a

^{1}H-NMR (500 MHz, $CDCl_3$):

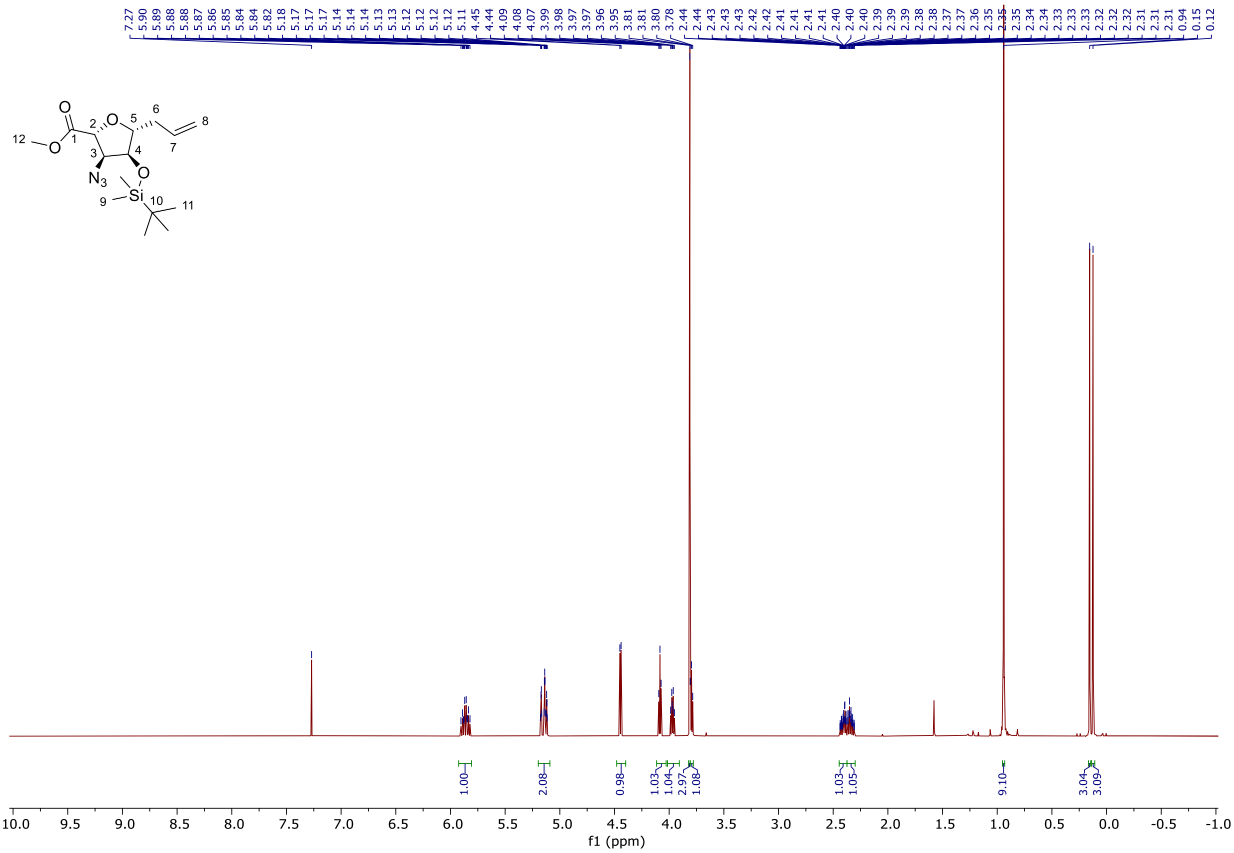

^{13}C-NMR (125 MHz, $CDCl_3$):

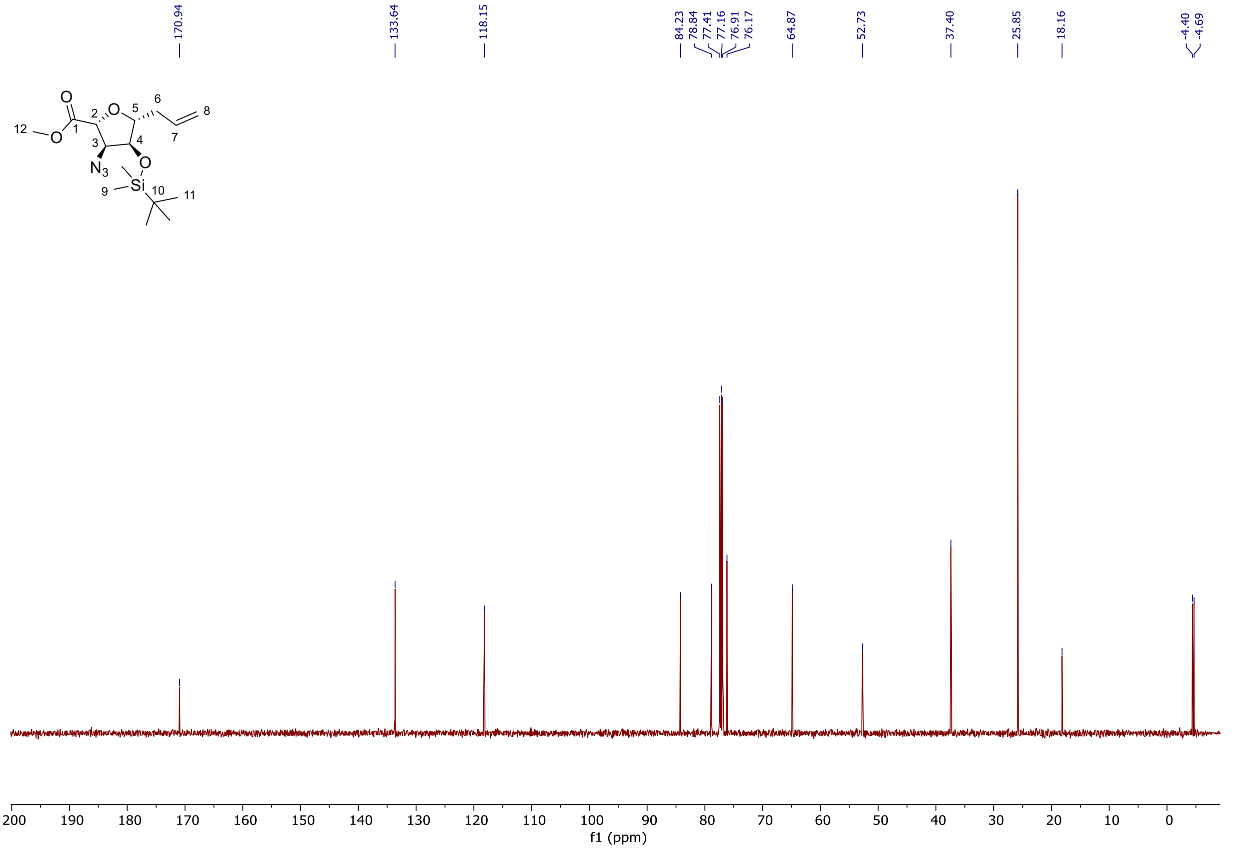

Methyl (2*S*,3*S*,4*S*,5*R*)-5-allyl-3-azido-4-((*tert*-butyldimethylsilyl)oxy)tetrahydrofuran-2-carboxylate 15c

^{1}H-NMR (500 MHz, $CDCl_3$):

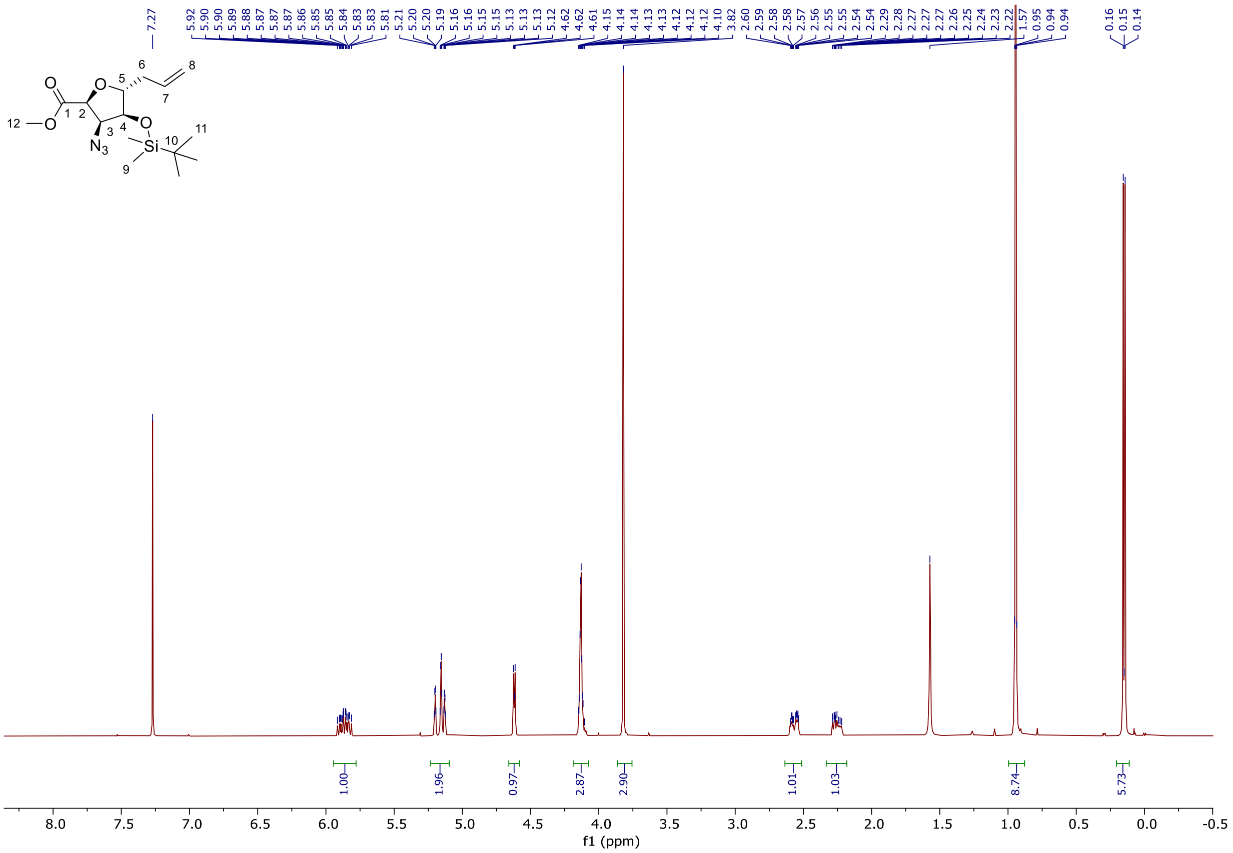

^{13}C-NMR (125 MHz, $CDCl_3$):

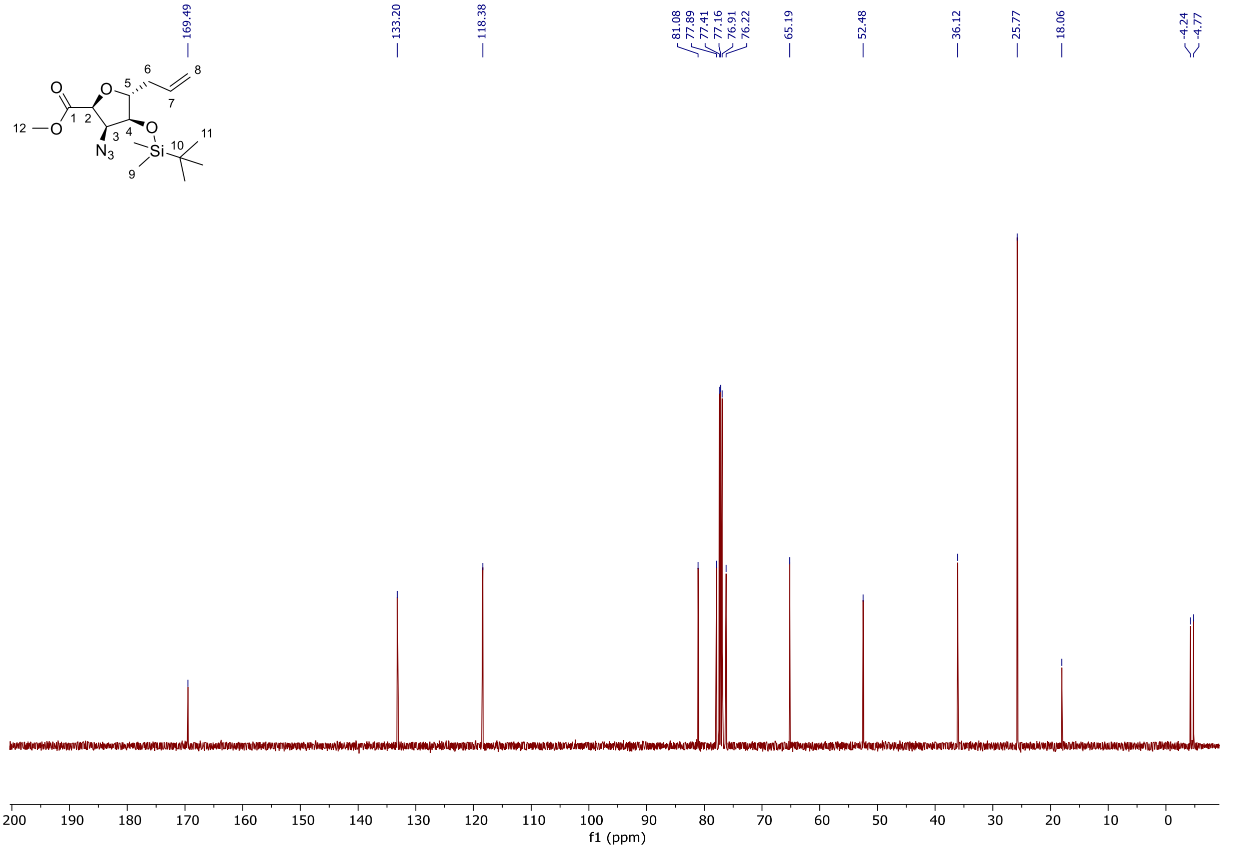

Methyl (2*R*,3*S*,4*S*,5*R*)-5-allyl-3-((*R*)-4-((*tert*-butoxycarbonyl)amino)-3-((*tert*-butyldimethylsilyl)-oxy)butanamido)-4-((*tert*-butyldimethylsilyl)oxy)tetrahydrofuran-2-carboxylate 17a

^{1}H-NMR (500 MHz, $CDCl_3$):

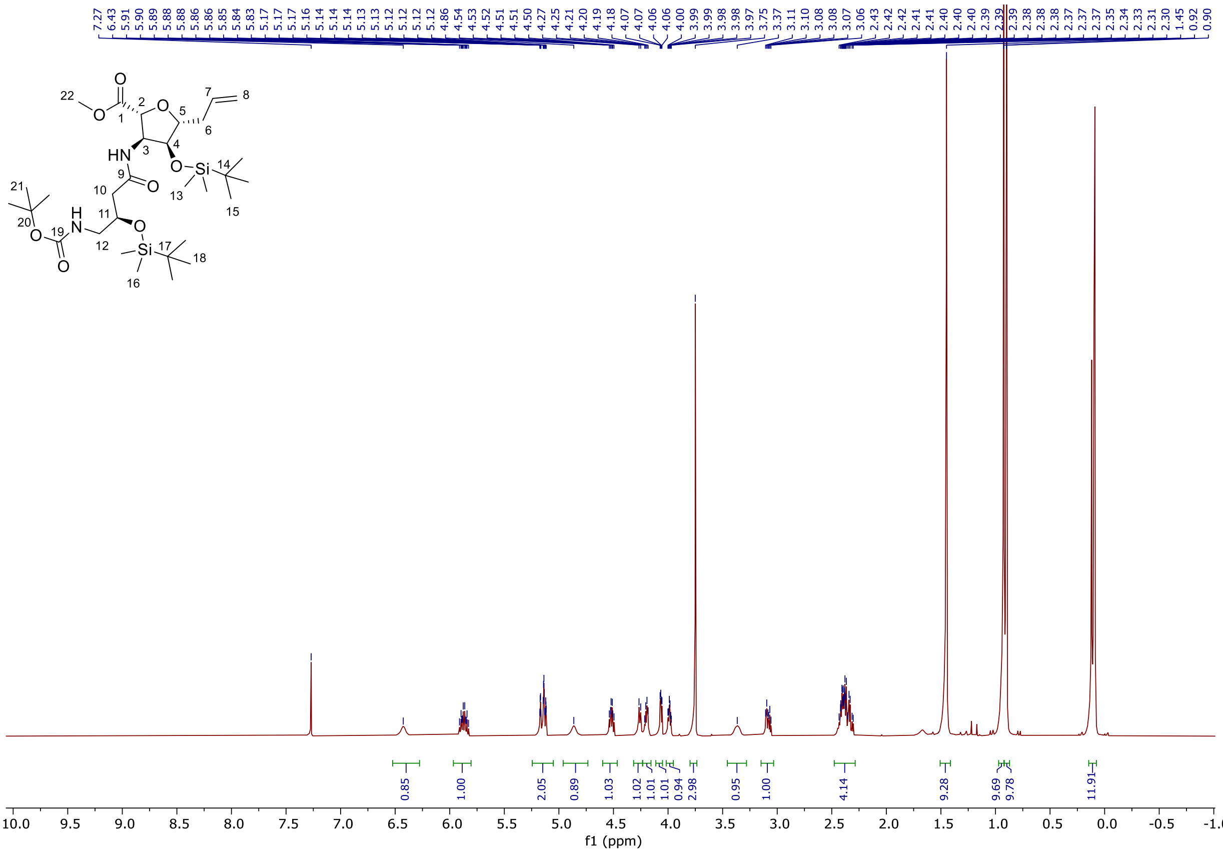

^{13}C-NMR (125 MHz, $CDCl_3$):

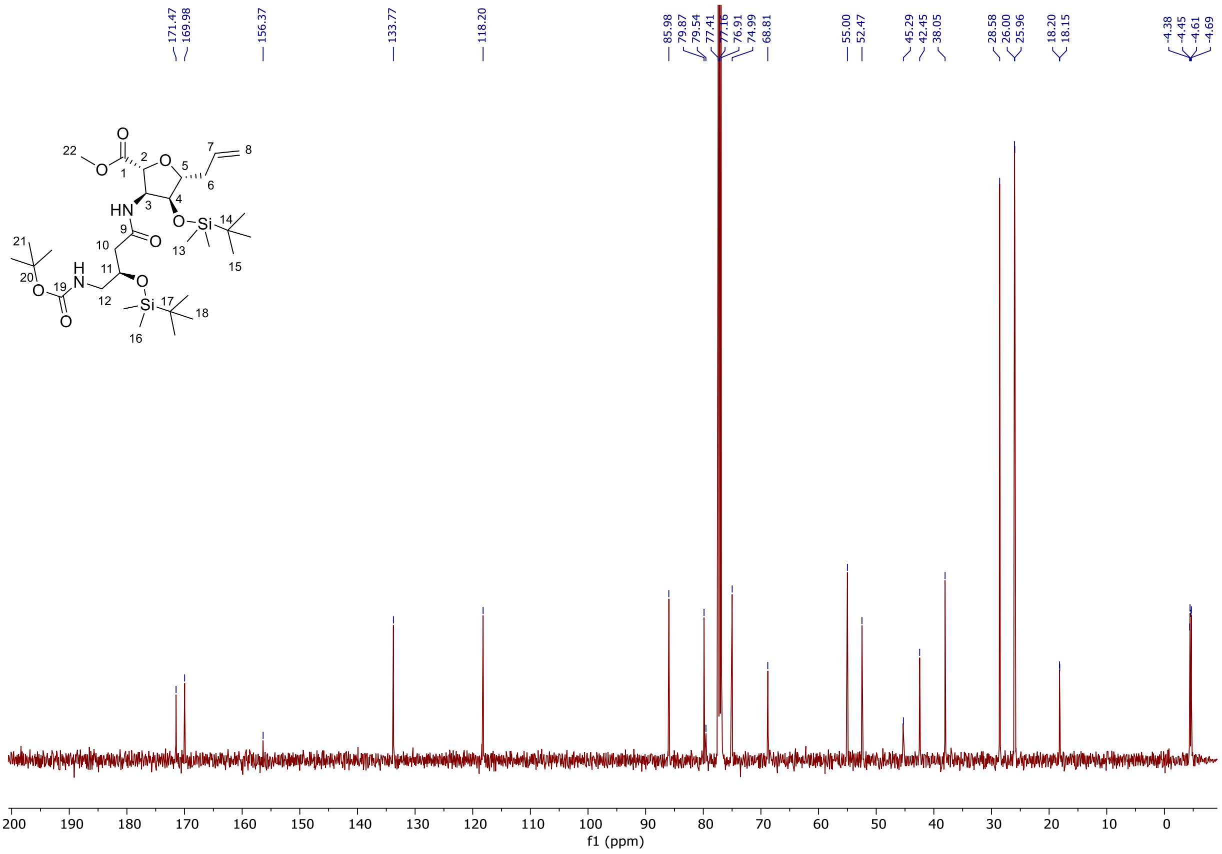

Methyl (2*R*,3*R*,4*S*,5*S*)-5-allyl-3-((*R*)-4-((*tert*-butoxycarbonyl)amino)-3-((*tert*-butyldimethylsilyl)-oxy)butanamido)-4-hydroxytetrahydrofuran-2-carboxylate 17b

^{1}H-NMR (500 MHz, $CDCl_3$):

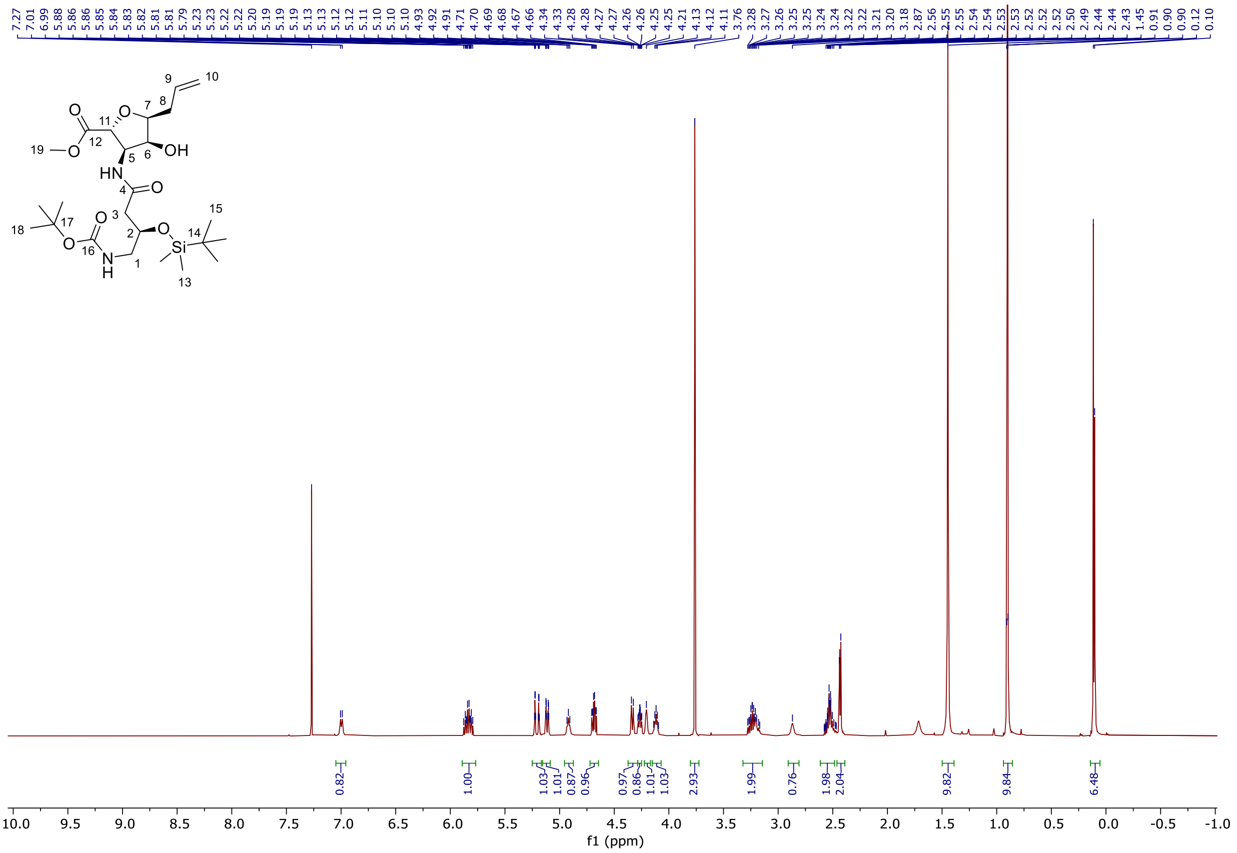

^{13}C-NMR (125 MHz, $CDCl_3$):

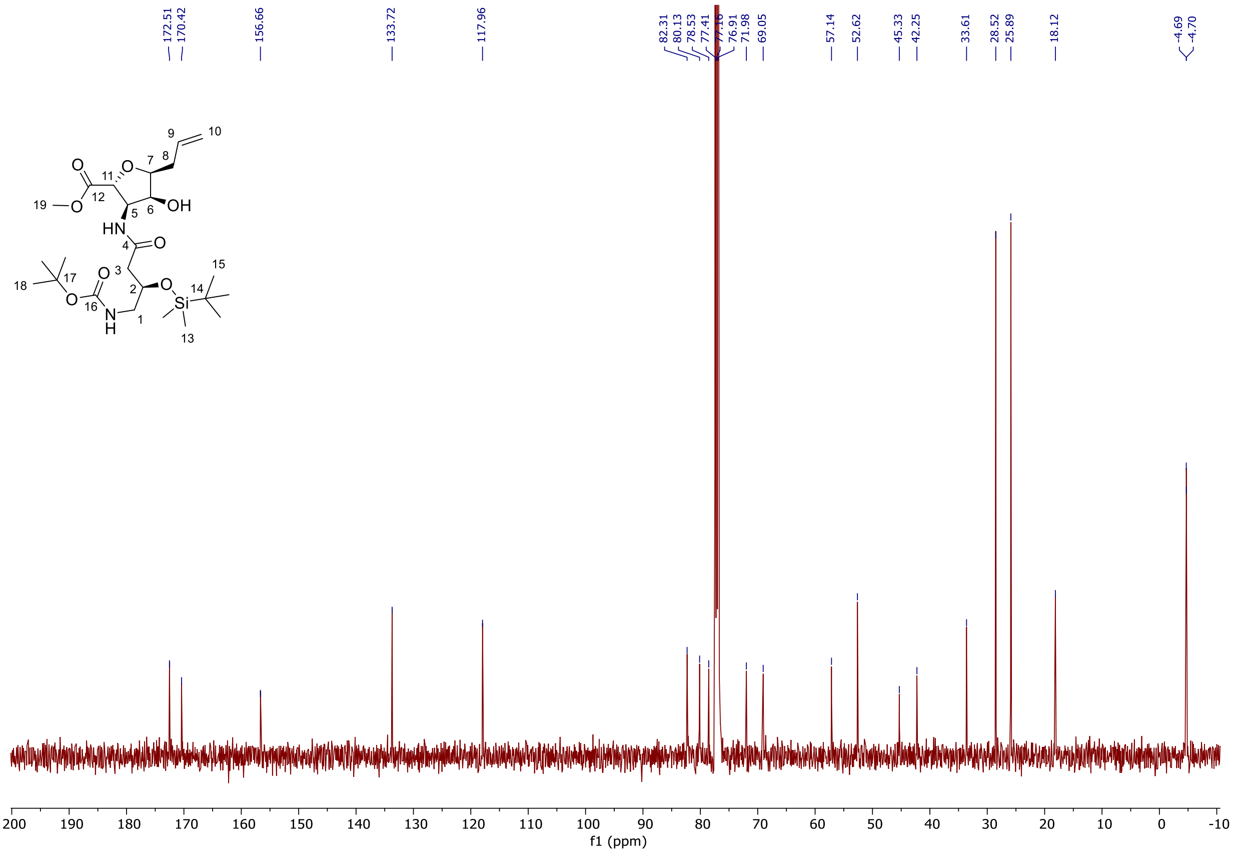

Methyl (2*S*,3*S*,4*S*,5*R*)-5-allyl-3-((*R*)-4-((*tert*-butoxycarbonyl)amino)-3-((*tert*-butyldimethylsilyl)-oxy)butanamido)-4-((*tert*-butyldimethylsilyl)oxy)tetrahydrofuran-2-carboxylate 17c

^{1}H-NMR (500 MHz, $CDCl_3$):

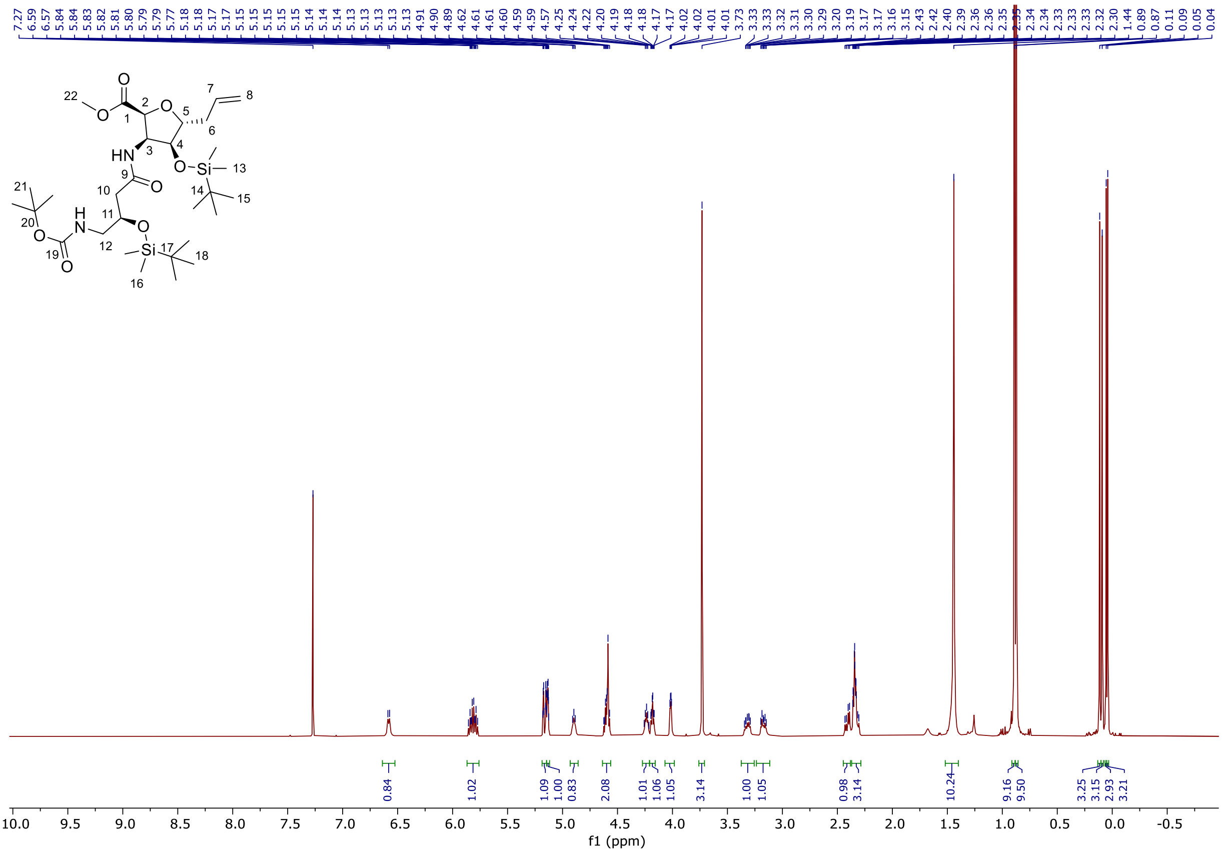

^{13}C-NMR (100 MHz, $CDCl_3$):

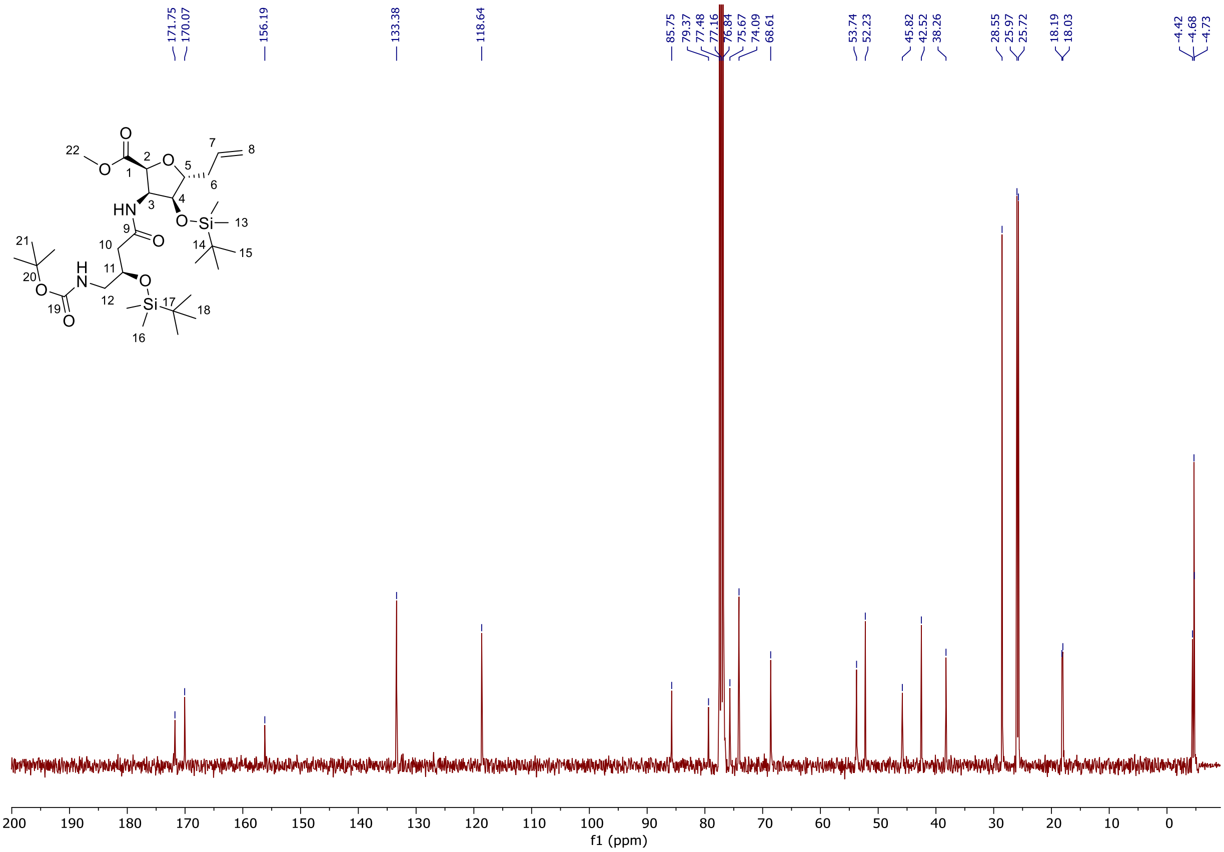

***tert*-Butyl ((*R*)-4-(((2*R*,3*R*,4*S*,5*S*)-5-allyl-2-(((*tert*-butyldimethylsilyl)oxy)methyl)-4-hydroxy-tetrahydrofuran-3-yl)amino)-2-((*tert*-butyldimethylsilyl)oxy)-4-oxobutyl)carbamate 19b**

^{1}H-NMR (500 MHz, $CDCl_3$):

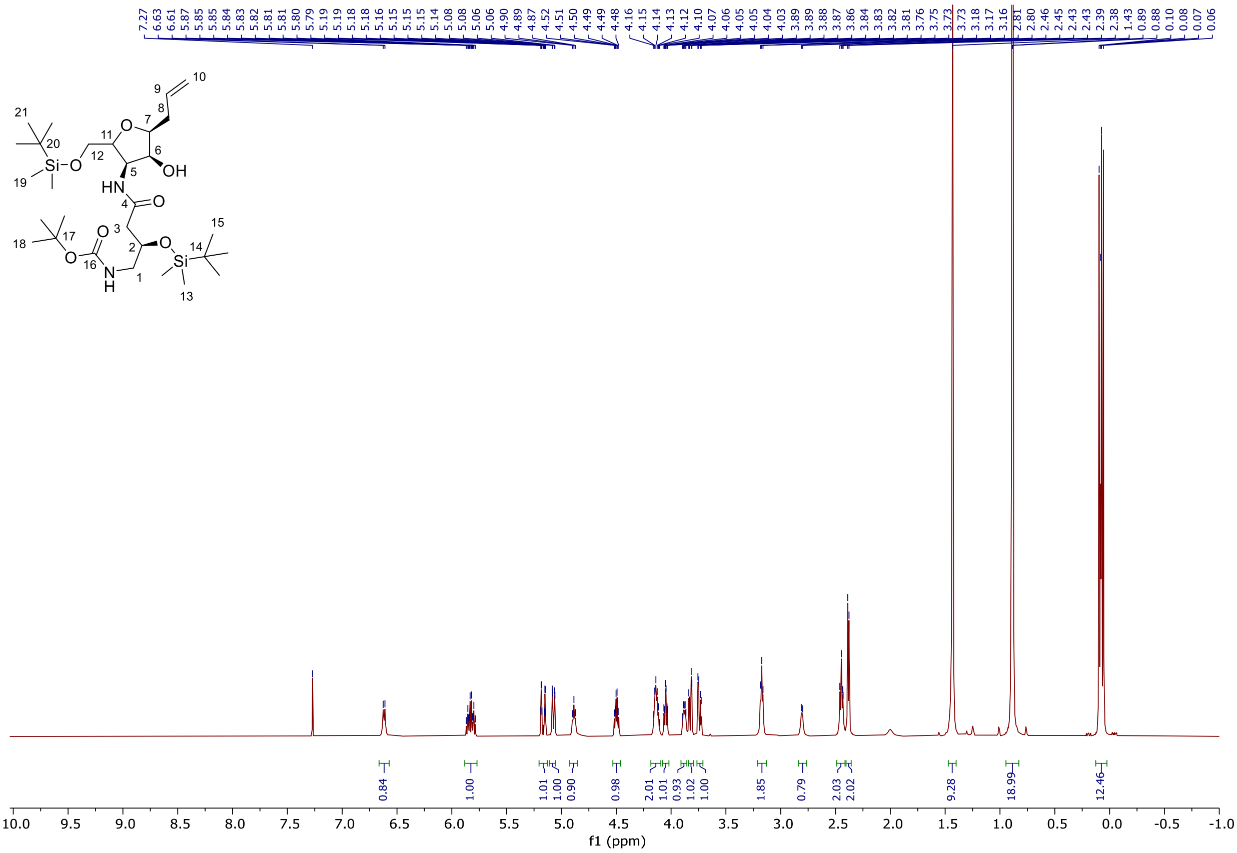

^{13}C-NMR (125 MHz, $CDCl_3$):

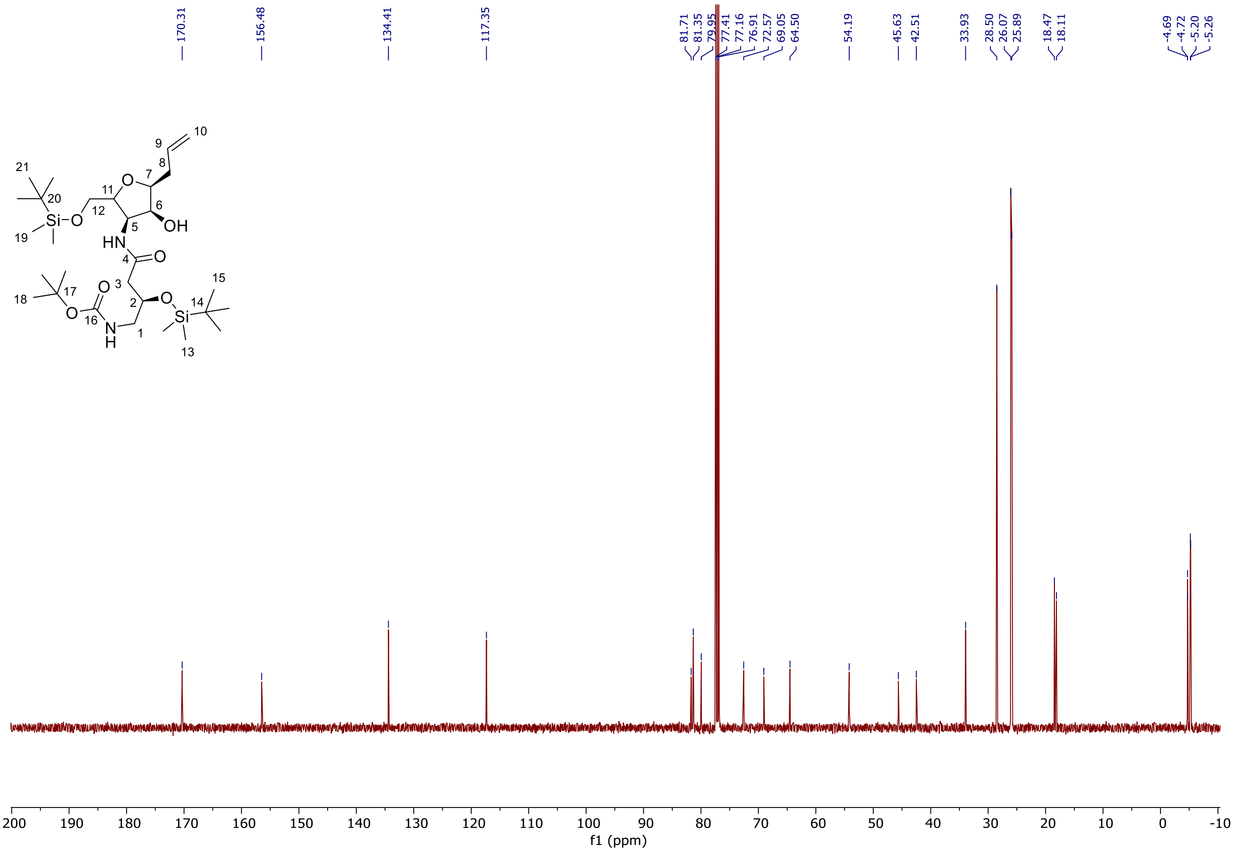

***tert*-Butyl ((*R*)-4-(((2*R*,3*R*,4*S*,5*S*)-5-allyl-4-hydroxy-2-(hydroxymethyl)tetrahydrofuran-3-yl)amino)-2-((*tert*-butyldimethylsilyl)oxy)-4-oxobutyl)carbamate 20b**

^{1}H-NMR (500 MHz, $CDCl_3$):

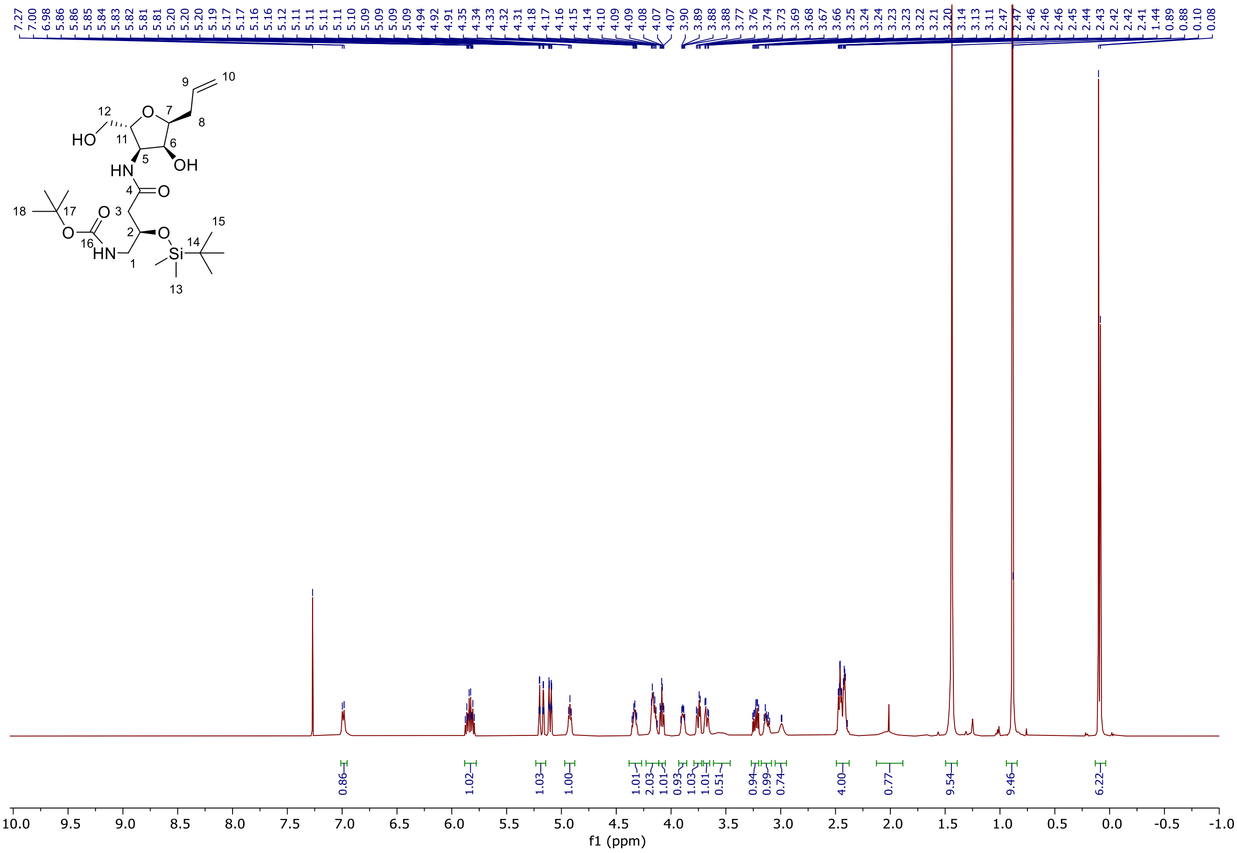

^{13}C-NMR (125 MHz, $CDCl_3$):

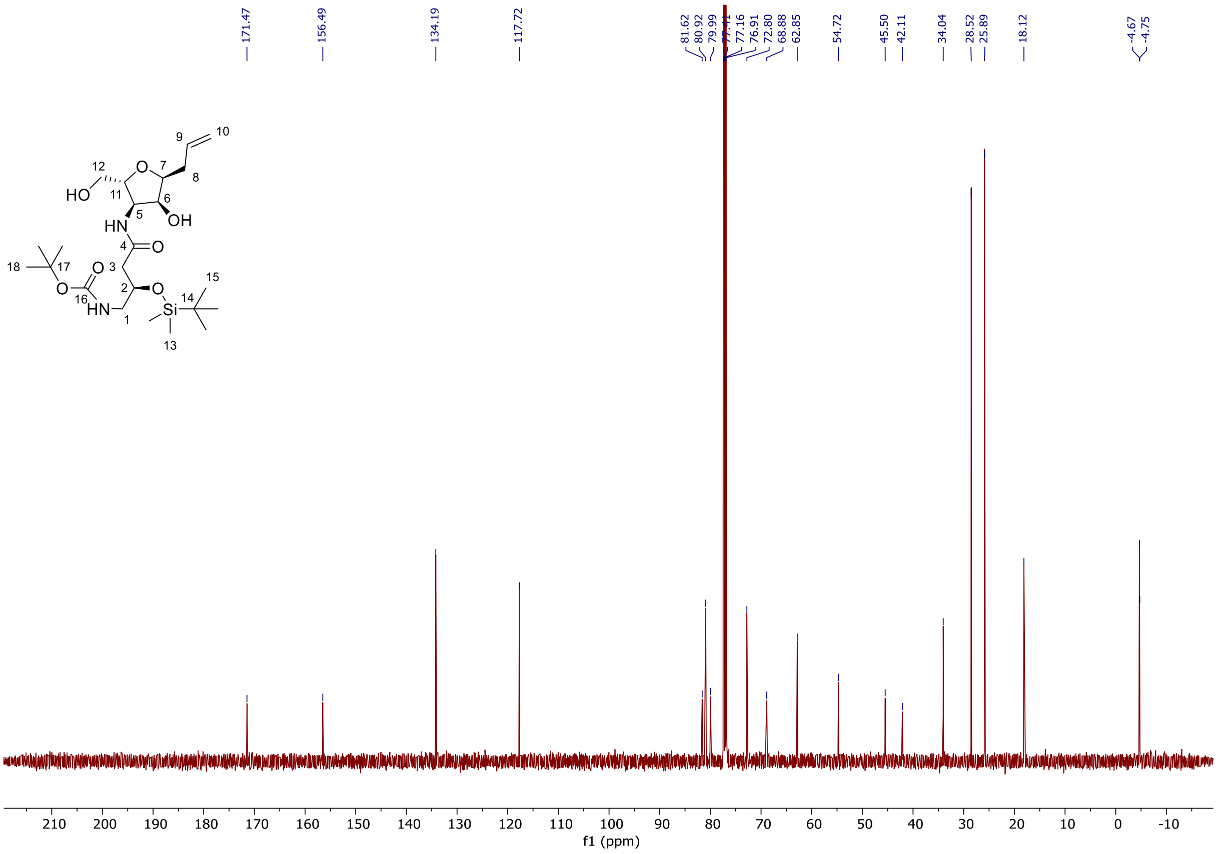

***tert*-Butyl ((*R*)-4-(((2*S*,3*R*,4*S*,5*S*)-5-allyl-4-hydroxy-2-(hydroxymethyl)tetrahydrofuran-3-yl)amino)-2-((*tert*-butyldimethylsilyl)oxy)-4-oxobutyl)carbamate 20d**

^{1}H-NMR (500 MHz, $CDCl_3$):

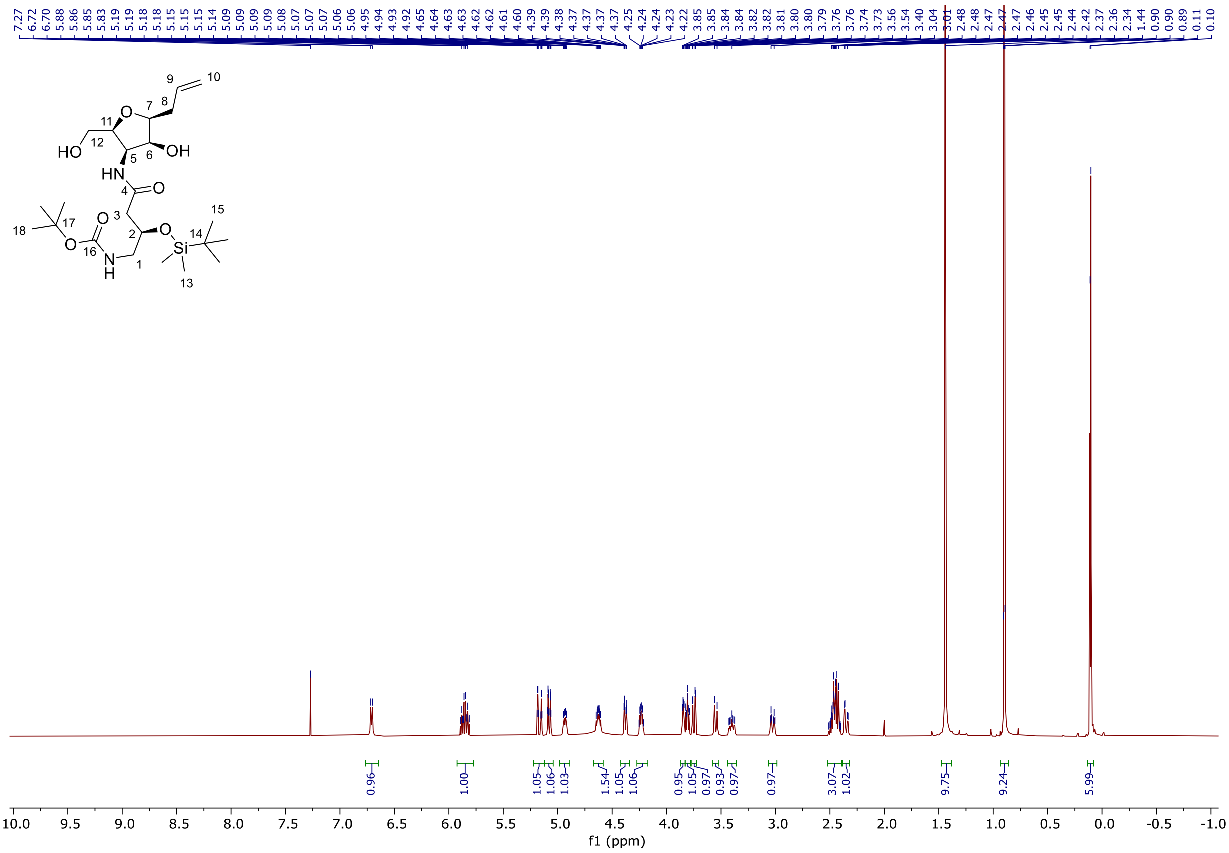

^{13}C-NMR (125 MHz, $CDCl_3$):

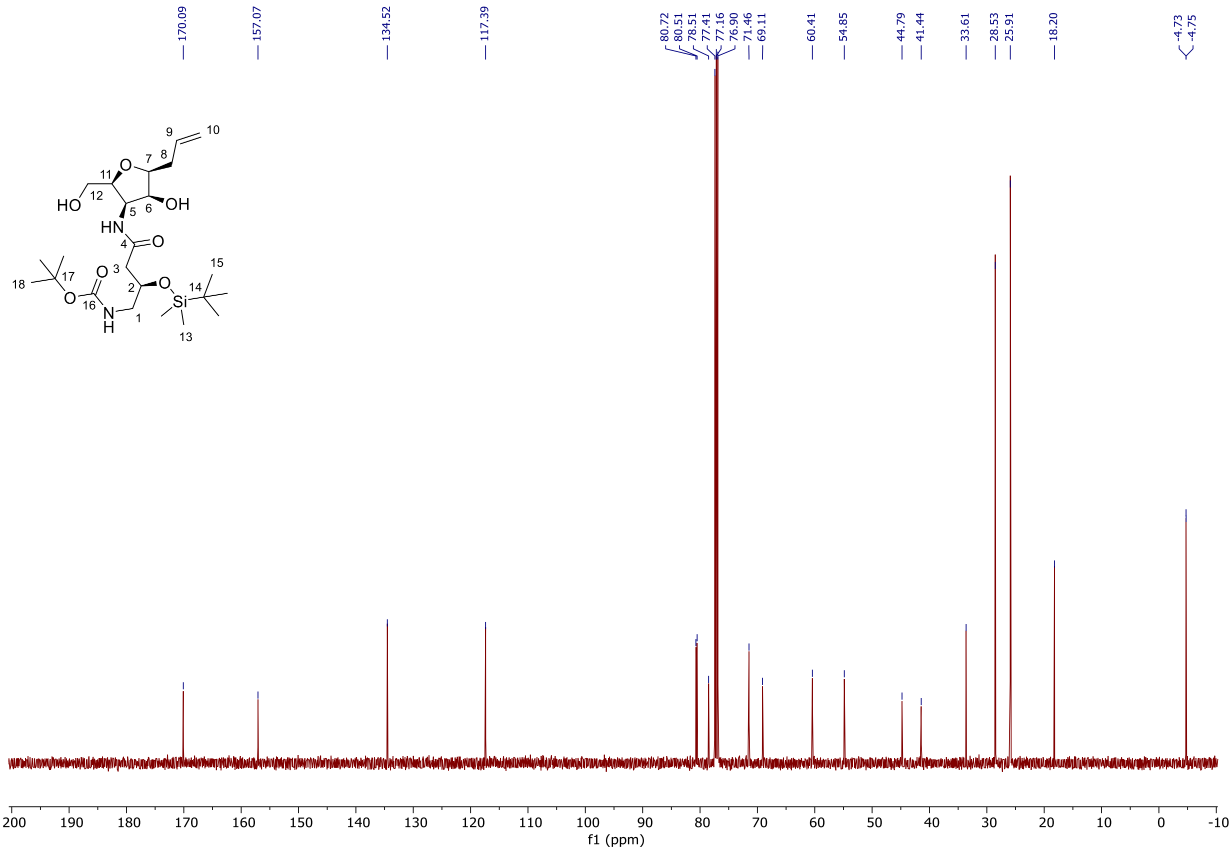

Methyl (2*S*,3*S*,4*S*,5*R*)-5-allyl-3-((tert-butoxycarbonyl)amino)-4-((*tert*-butyldimethylsilyl)oxy)-tetrahydrofuran-2-carboxylate 21

^{1}H-NMR (400 MHz, $CDCl_3$):

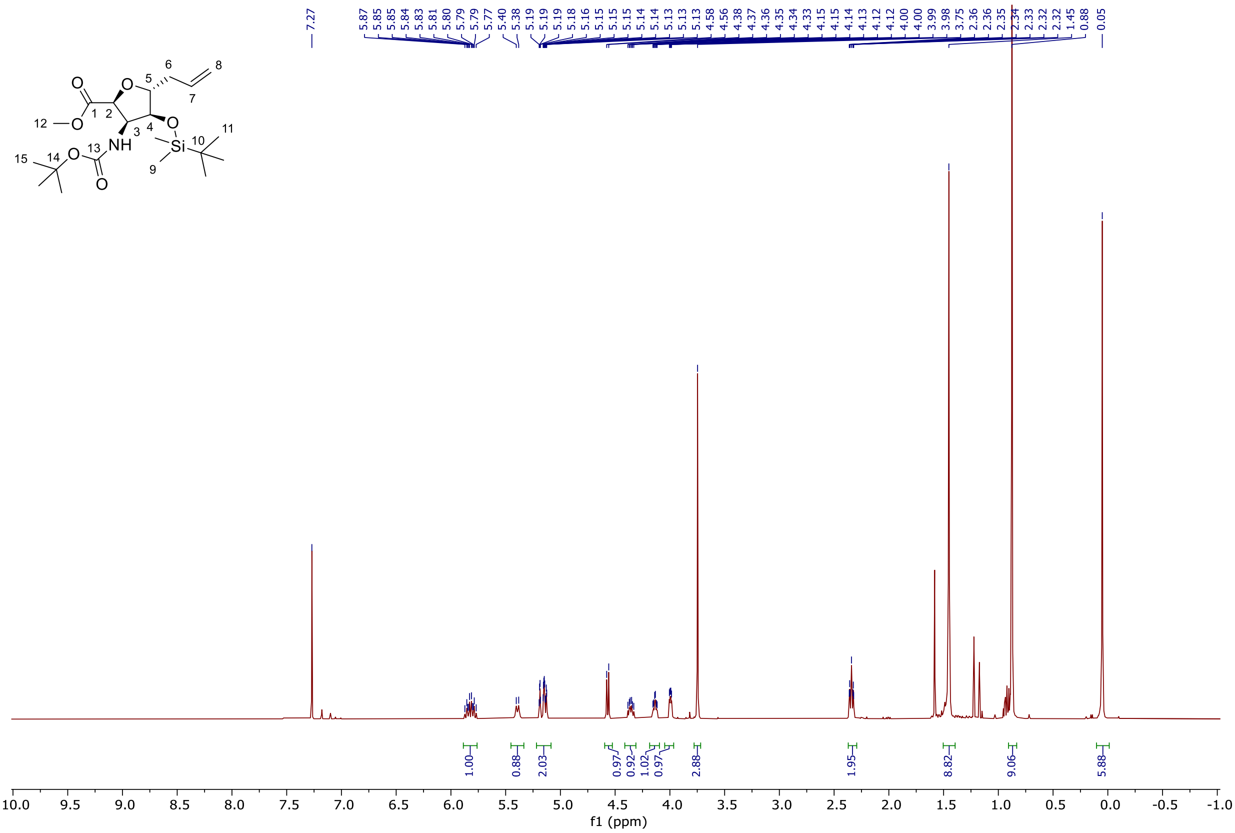

^{13}C-NMR (100 MHz, $CDCl_3$):

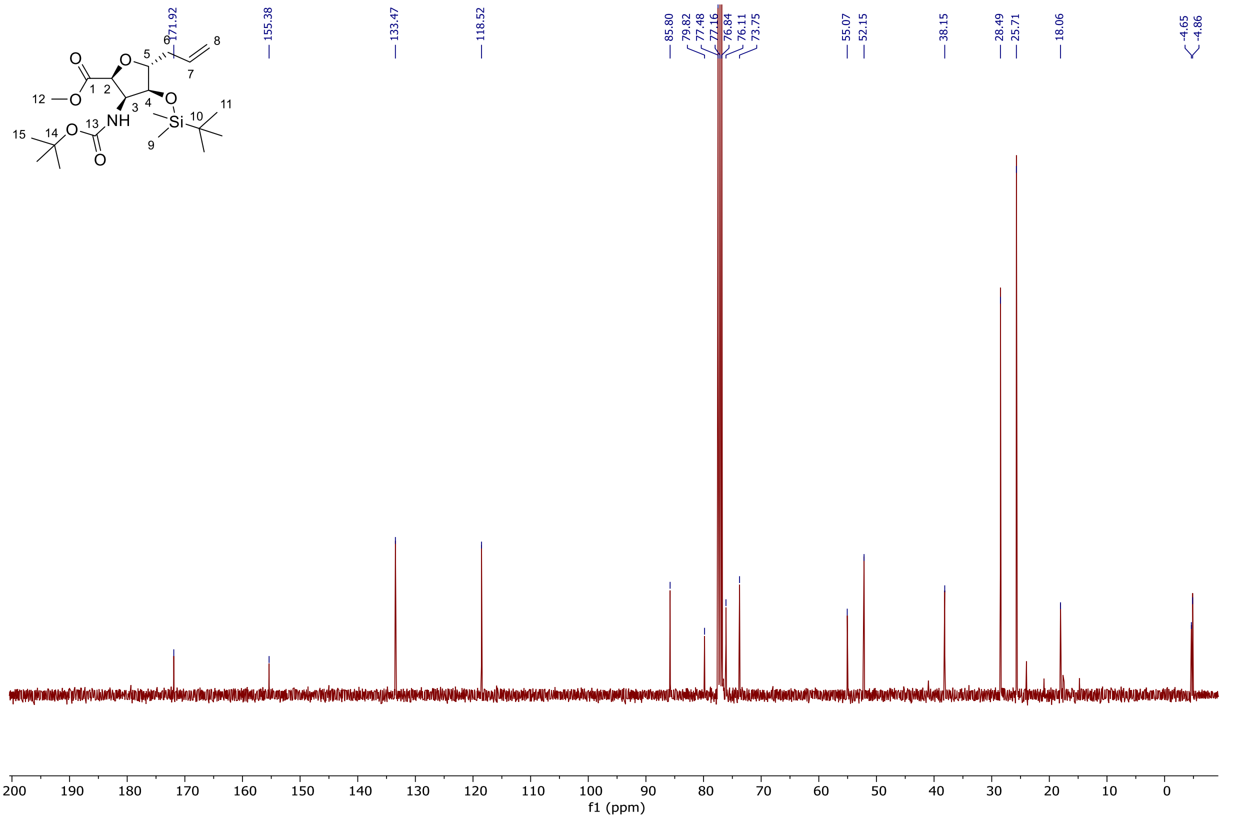

Methyl *N*-((*Z*)-2-((*R*)-3-((2*R*,3*S*,4*S*,5*R*)-5-allyl-3-((*R*)-4-((*tert*-butoxycarbonyl)amino)-3-((*tert*-butyldimethylsilyl)oxy)butanamido)-4-((*tert*-butyldimethylsilyl)oxy)tetrahydrofuran-2-carboxamido)-5-oxopyrrolidin-2-ylidene)acetyl)-*N*-methylglycinate 22a

^{1}H-NMR (500 MHz, DMSO-d$_6$, 373 K):

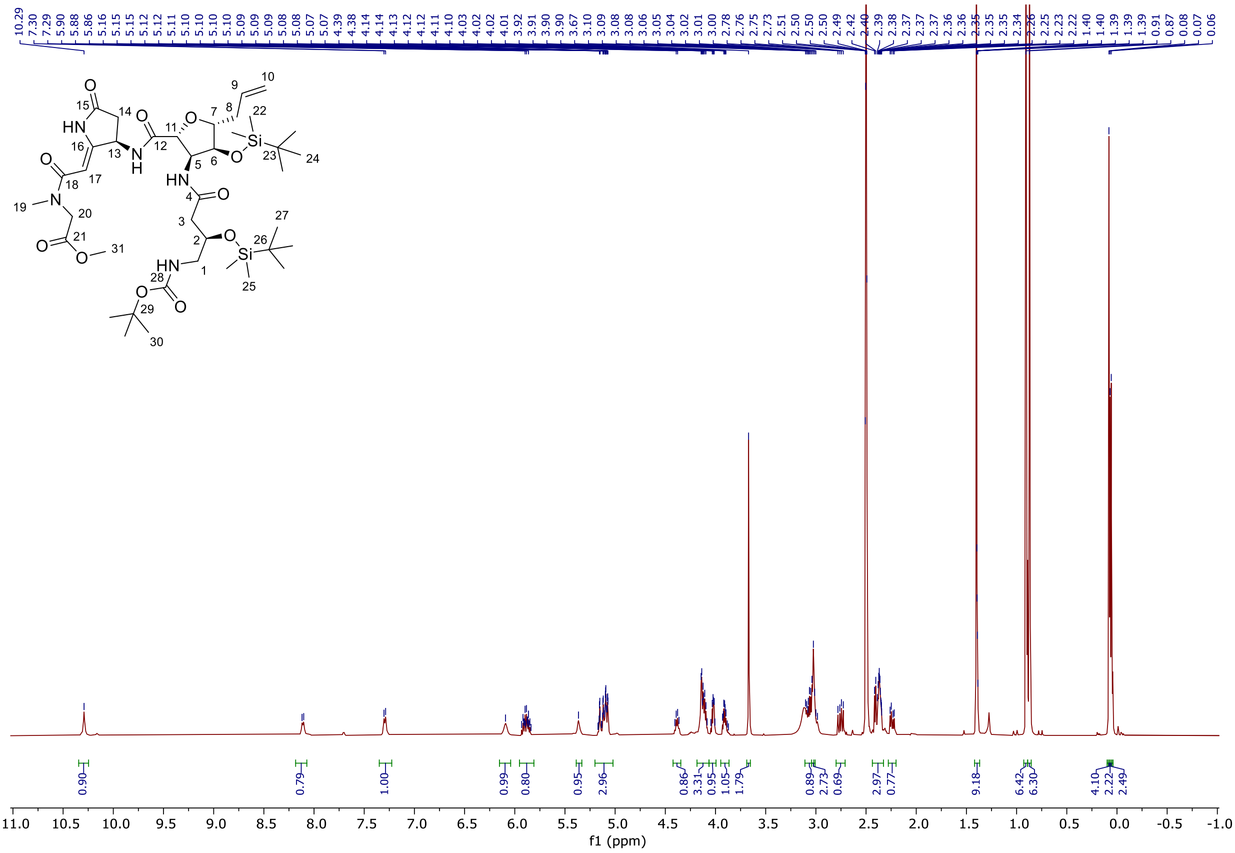

^{13}C-NMR (125 MHz, DMSO-d$_6$, 298 K):

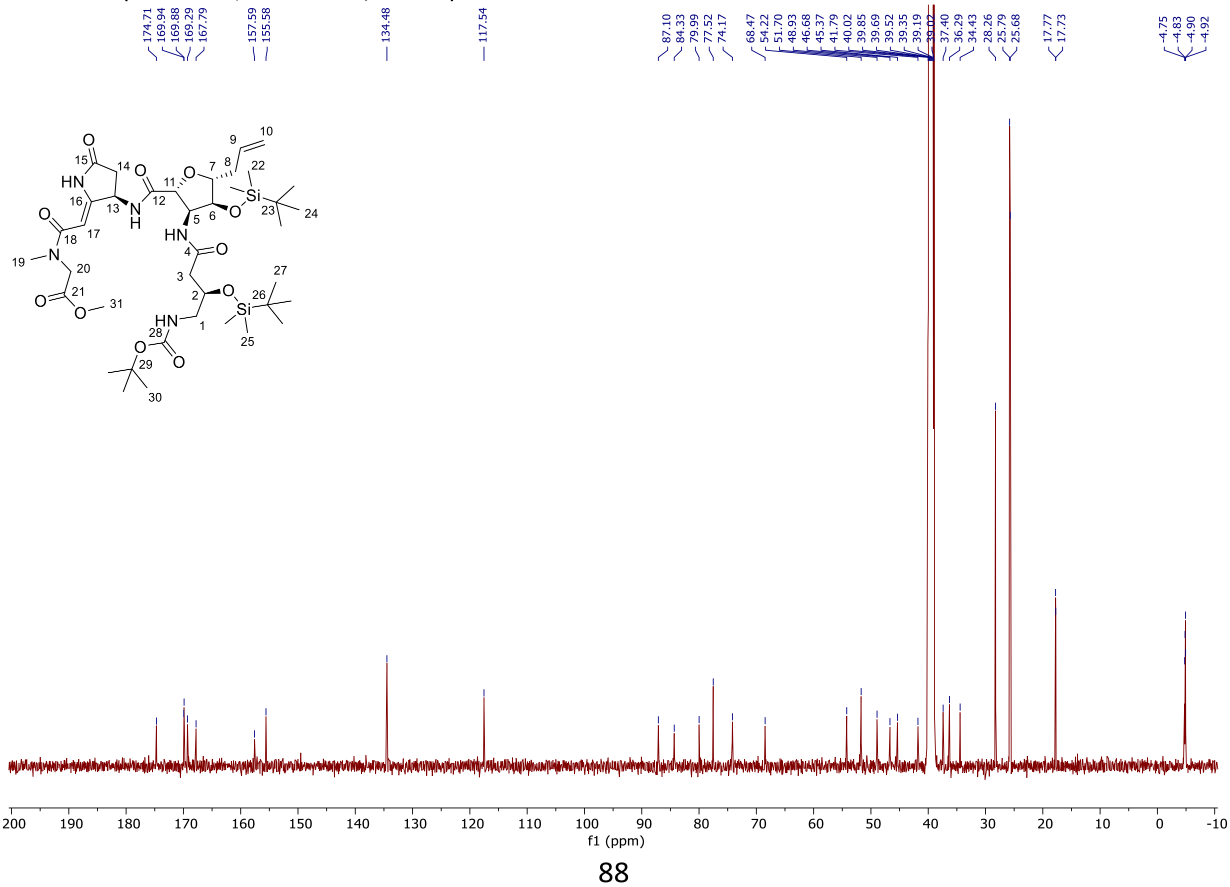

Methyl (*tert*-butoxycarbonyl)-D-tryptophylglycinate SI-6

^{1}H-NMR (500 MHz, $CDCl_3$):

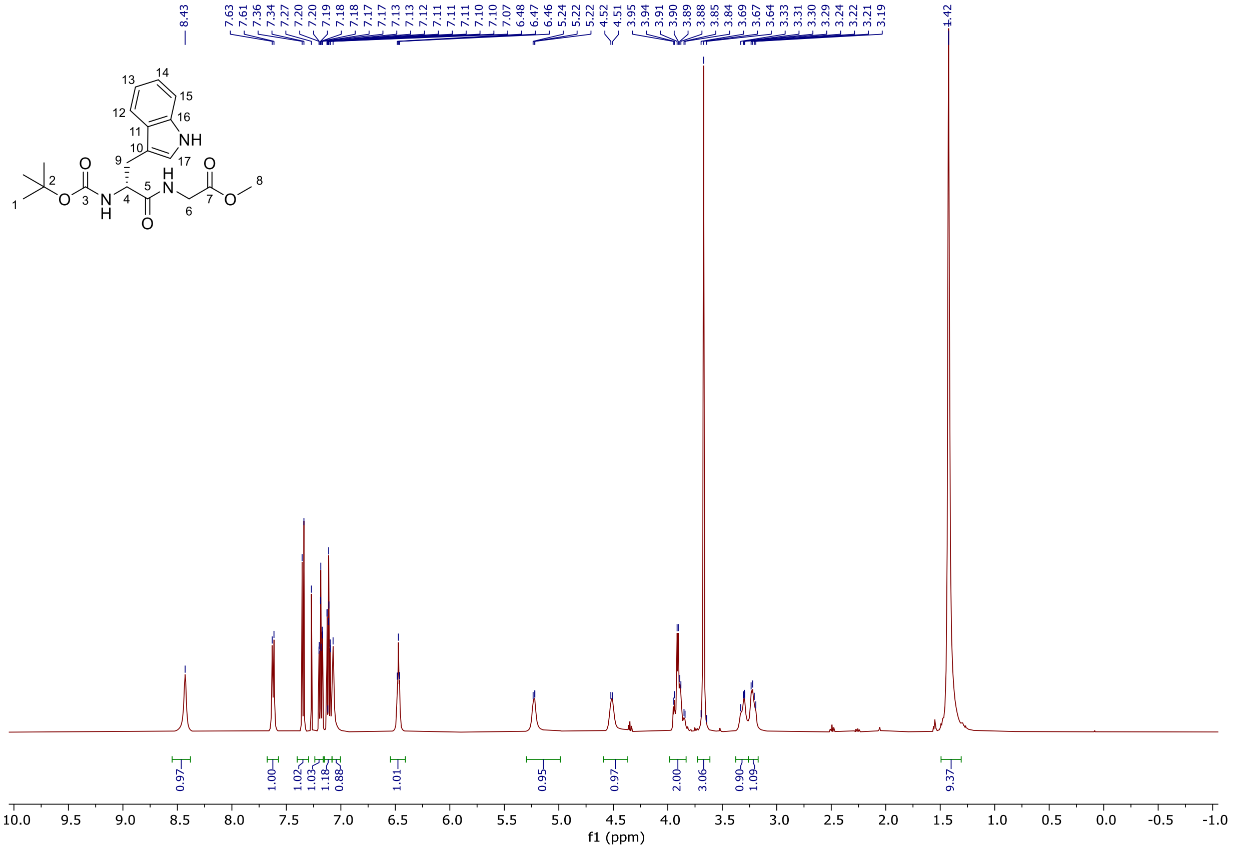

^{13}C-NMR (125 MHz, $CDCl_3$):

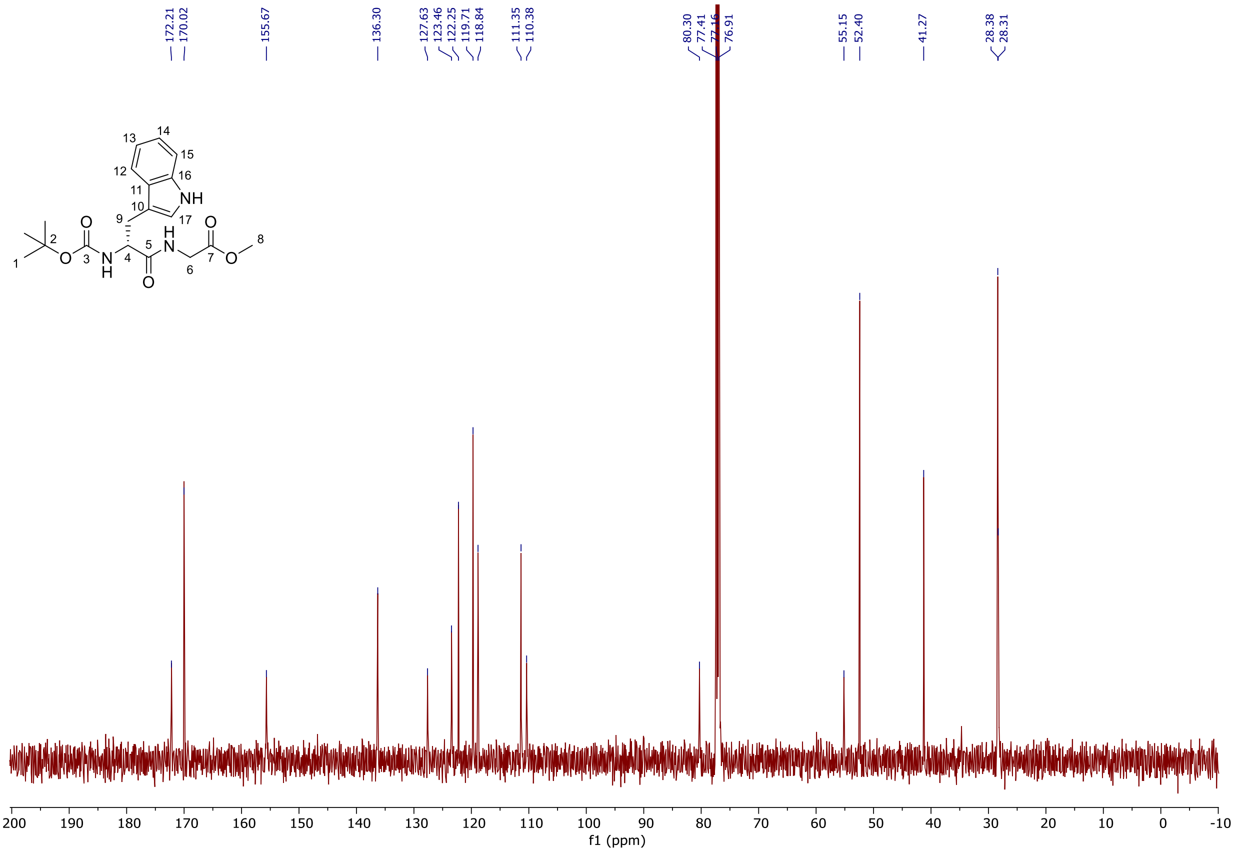

Methyl *N*-((*Z*)-2-((*R*)-3-((2*R*,3*S*,4*S*,5*R*)-5-allyl-3-((*R*)-4-((*tert*-butoxycarbonyl)amino)-3-((*tert*-butyldimethylsilyl)oxy)butanamido)-4-((*tert*-butyldimethylsilyl)oxy)tetrahydrofuran-2-carboxamido)-5-oxopyrrolidin-2-ylidene)acetyl)-*N*-methylglycyl-D-tryptophylglycinate 23a

^{1}H-NMR (500 MHz, DMSO-d$_6$):

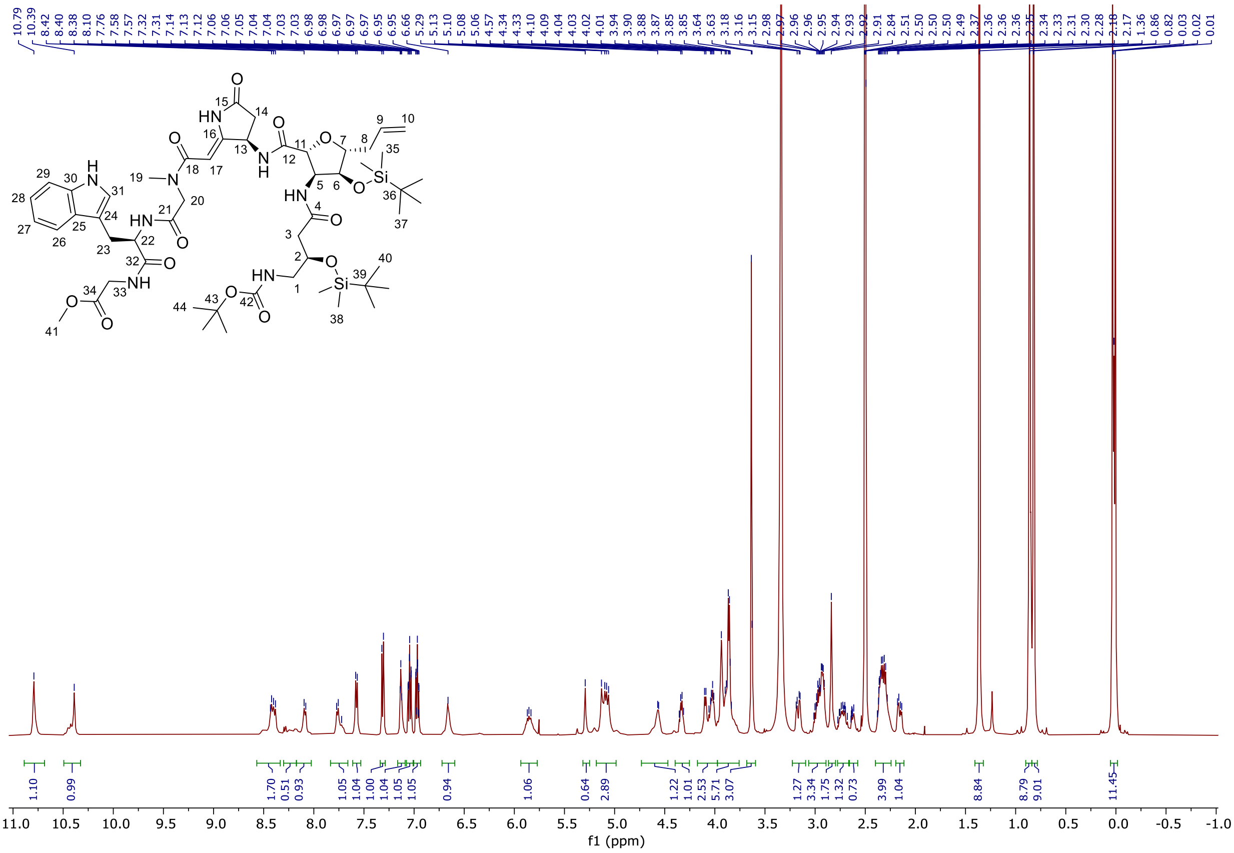

^{13}C-NMR (125 MHz, DMSO-d$_6$):

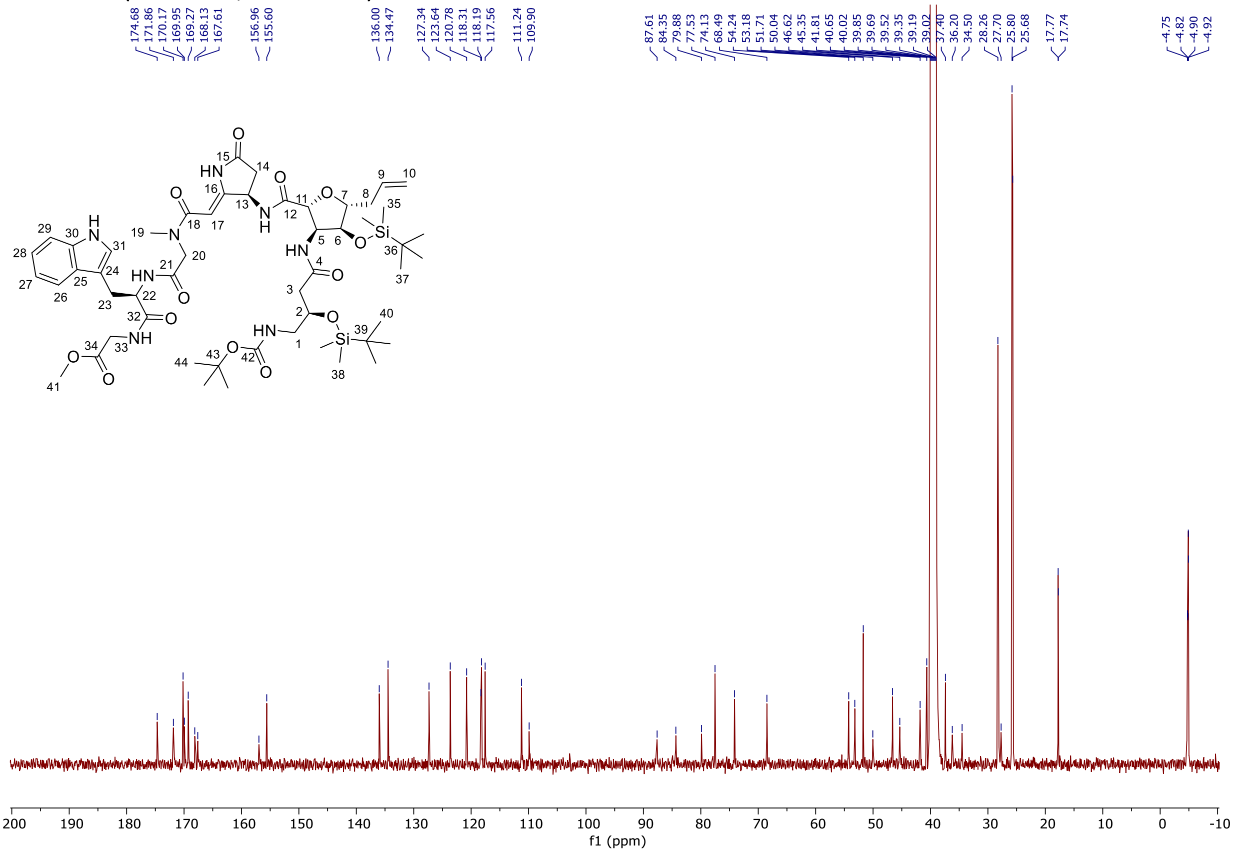

(2*R*,3*S*,3a*S*,7*R*,14*R*,23a*R*,25a*R*,*Z*)-14-((1*H*-Indol-3-yl)methyl)-2-allyl-3,7-bis((*tert*-butyldimethyl-silyl)oxy)-18-methyl-2,3,3a,6,7,8,9,11,12,14,15,17,18,23a,24,25a-hexadecahydrofuro[2,3-q]-pyrrolo[2,3-m][1,4,7,10,15,19]hexaazacyclotricosine-5,10,13,16,19,22,25(4*H*,21*H*,23*H*)-heptaone 25a

^{1}H-NMR (500 MHz, DMSO-d$_6$):

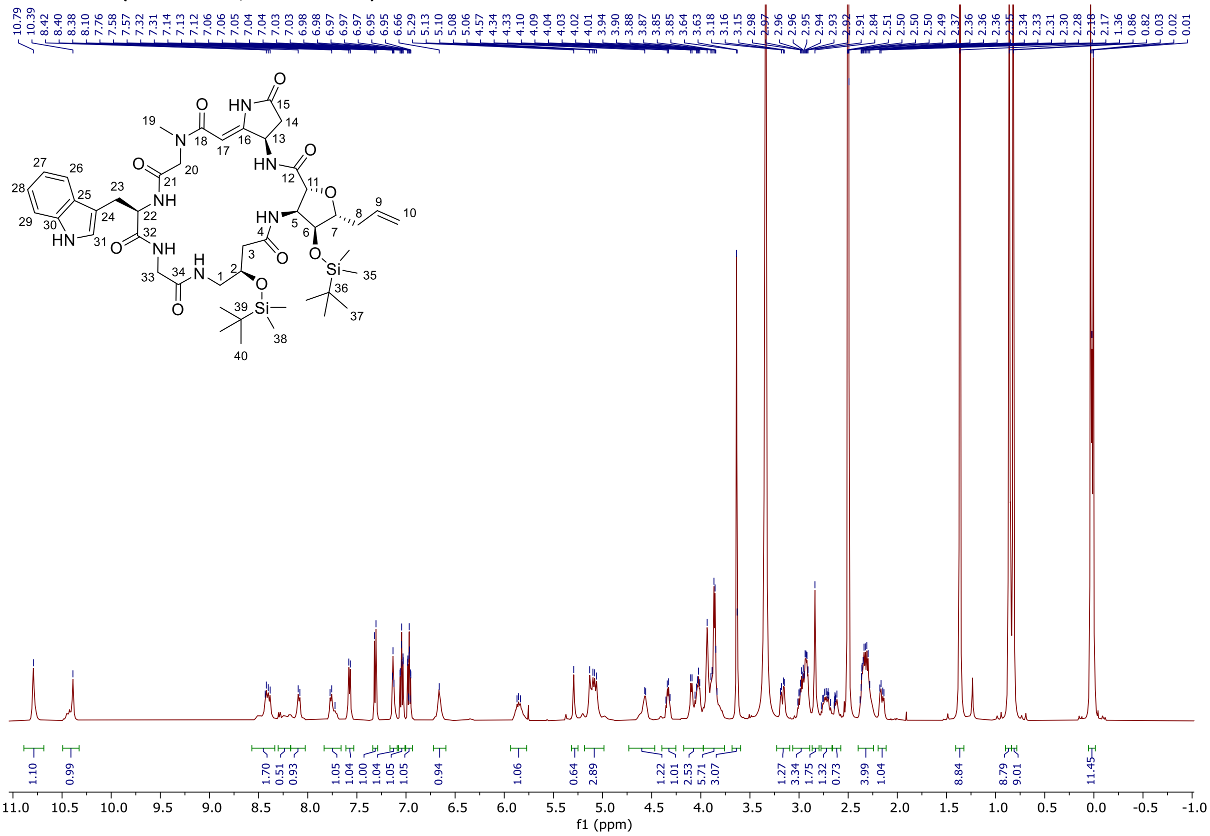

^{13}C-NMR (125 MHz, DMSO-d$_6$):

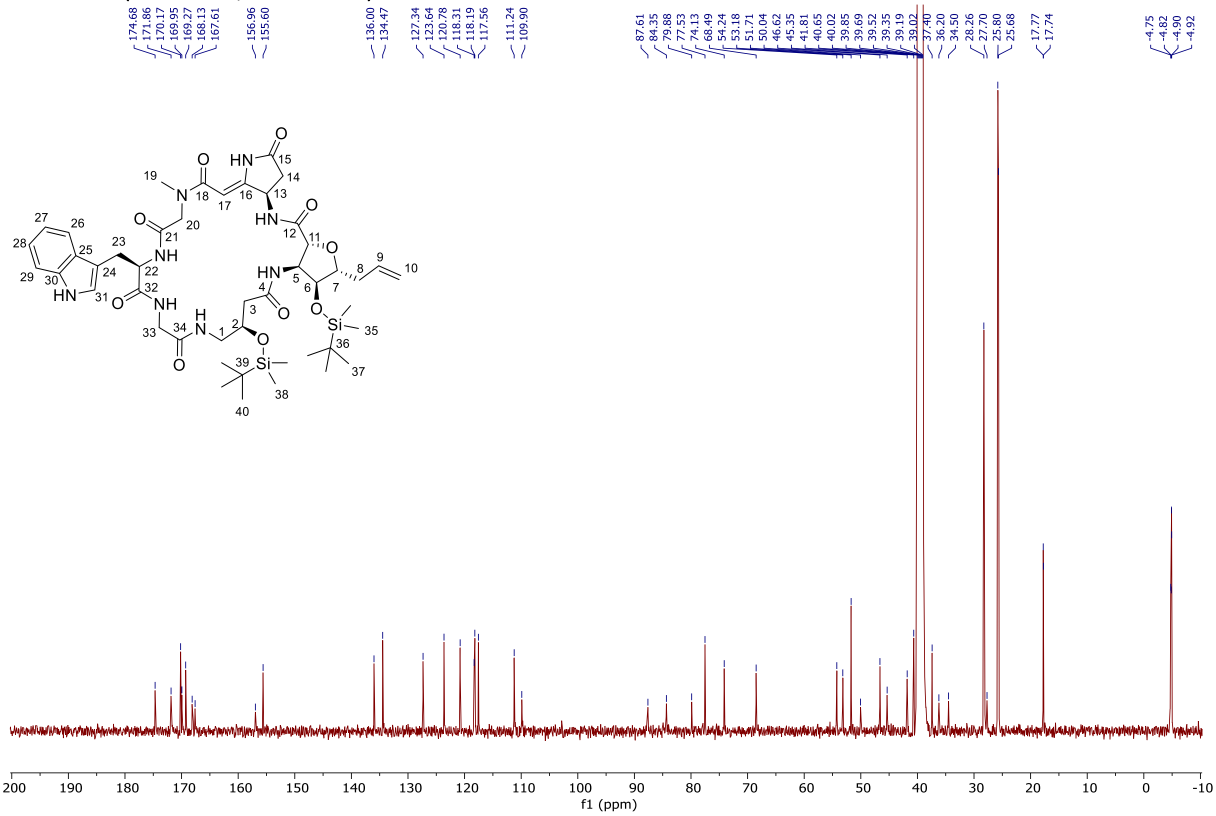

Simplified dehydrosocein 27a

^{1}H-NMR (500 MHz, DMSO-d$_6$):

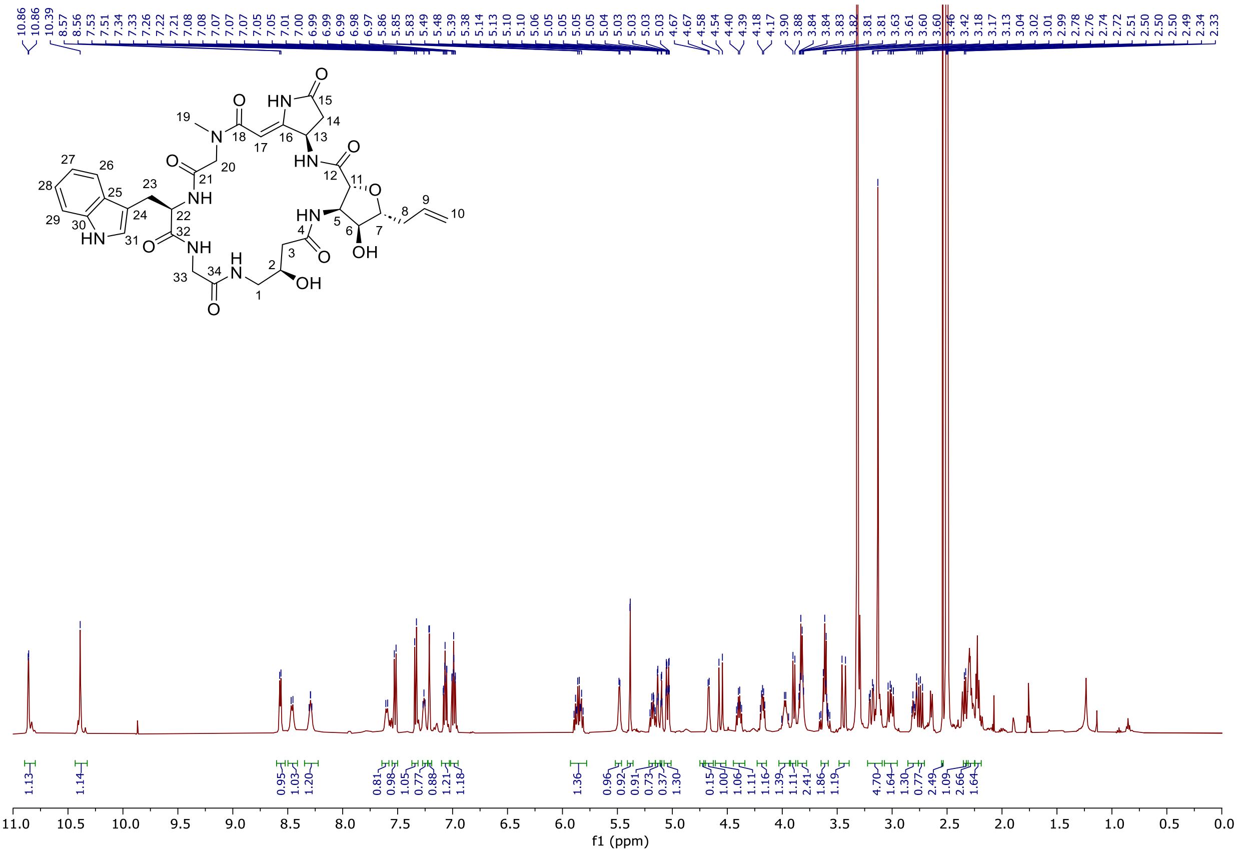

^{13}C-NMR (125 MHz, DMSO-d$_6$):

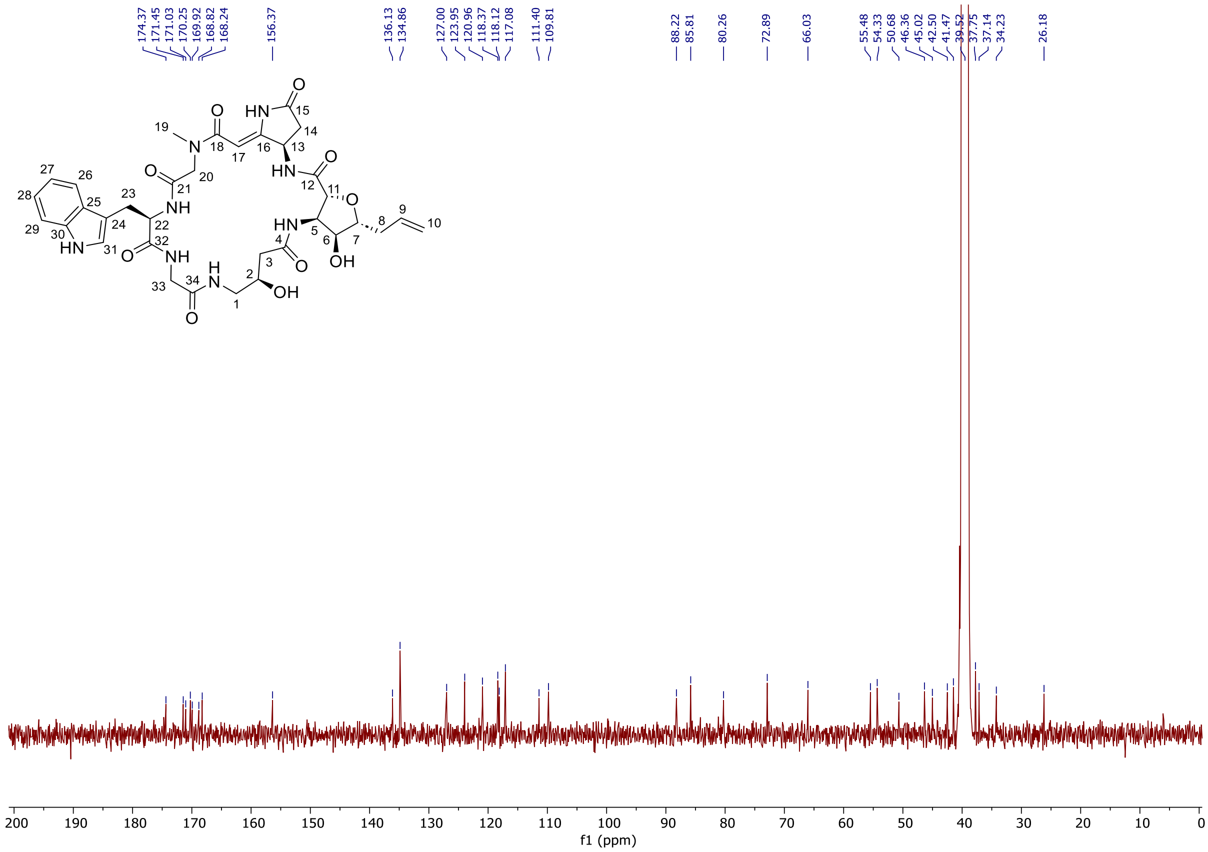

Simplified dehydrosocein 28a

^{1}H-NMR (500 MHz, DMSO-d$_6$):

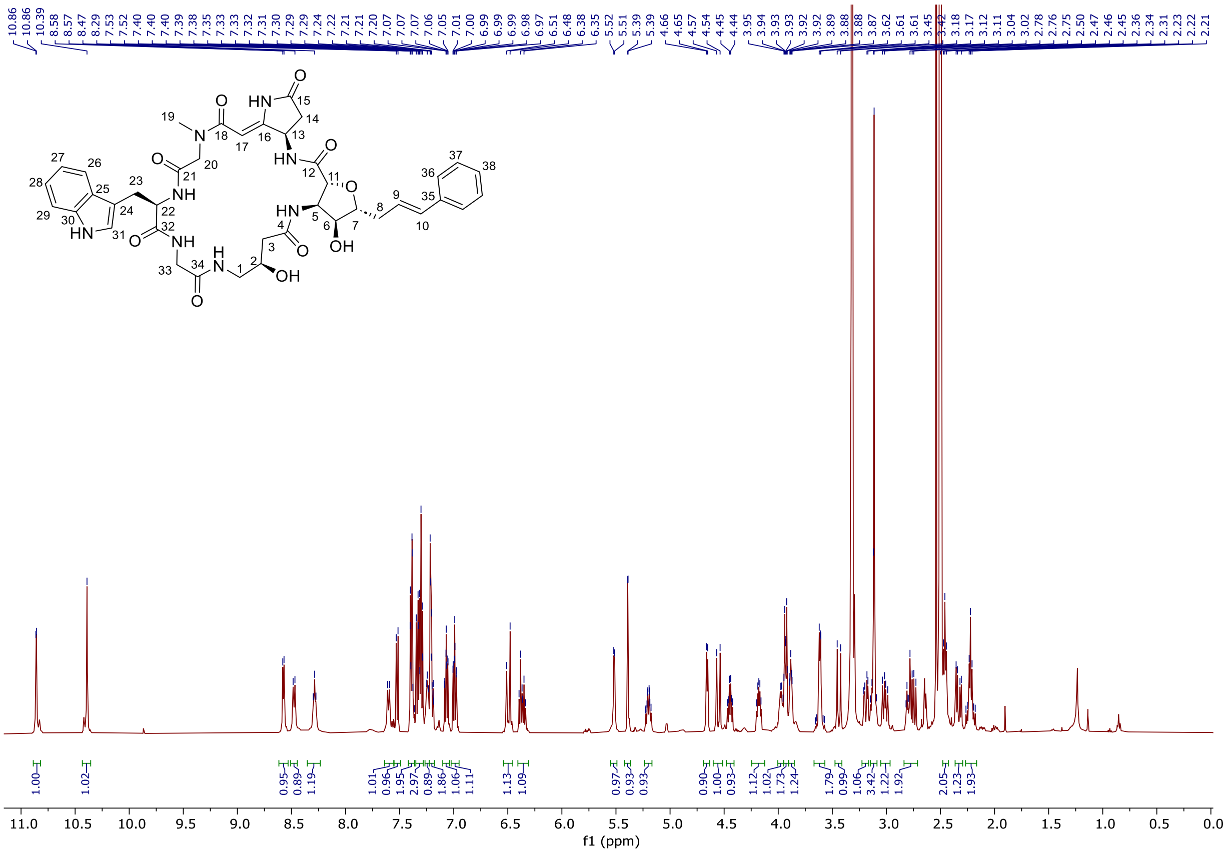

^{13}C-NMR (125 MHz, DMSO-d$_6$):

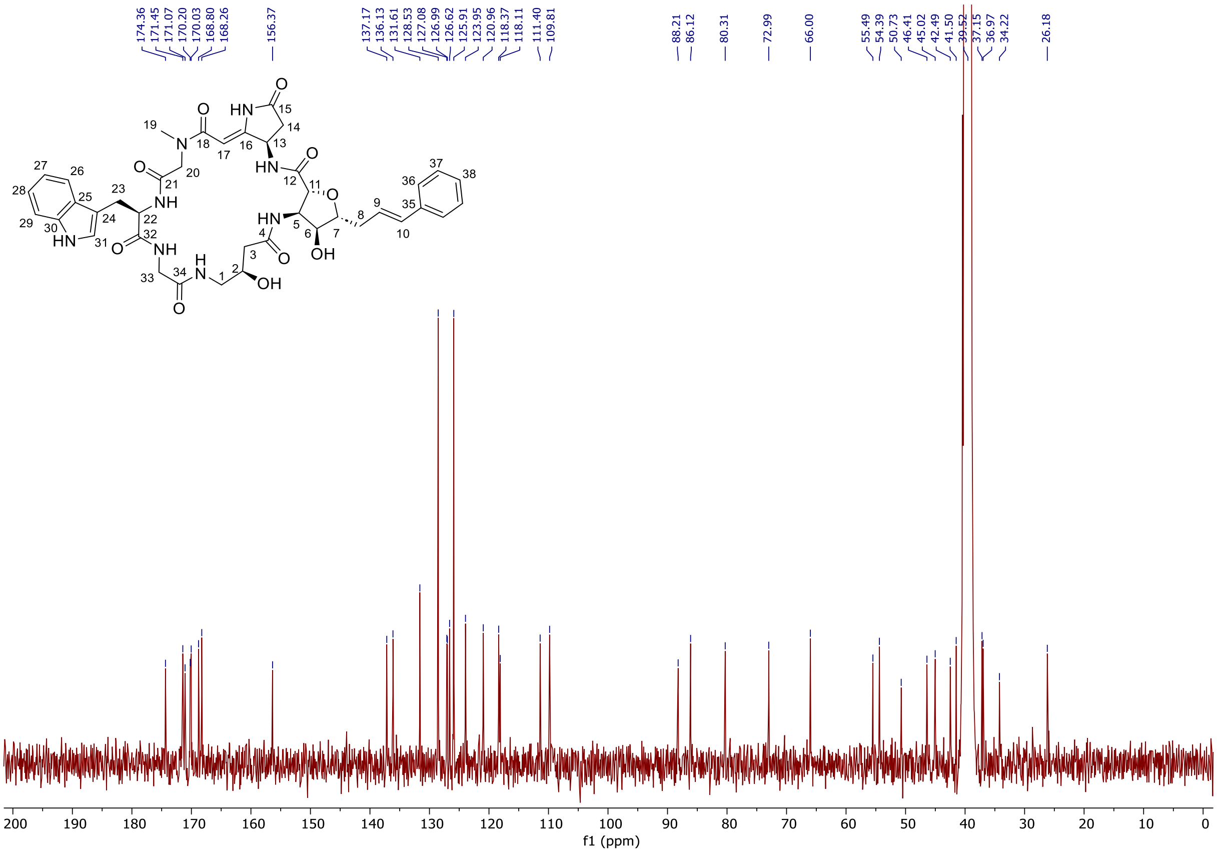

Methyl *N*-((*Z*)-2-((*R*)-3-((2*S*,3*S*,4*S*,5*R*)-5-allyl-3-((*R*)-4-((*tert*-butoxycarbonyl)amino)-3-((*tert*-butyldimethylsilyl)oxy)butanamido)-4-((*tert*-butyldimethylsilyl)oxy)tetrahydrofuran-2-carboxamido)-5-oxopyrrolidin-2-ylidene)acetyl)-*N*-methylglycinate 22c

^{1}H-NMR (500 MHz, DMSO-d$_6$, 373 K):

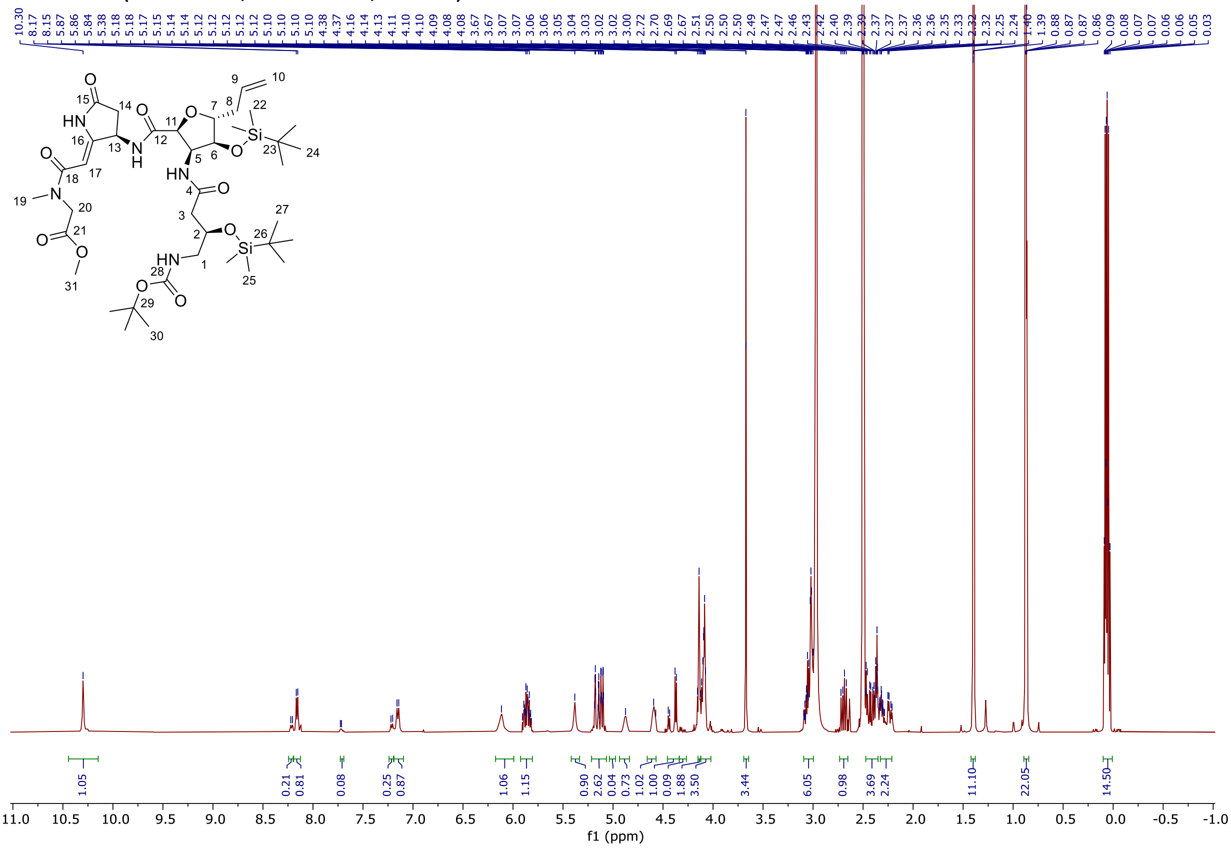

^{13}C-NMR (125 MHz, DMSO-d$_6$, 373 K):

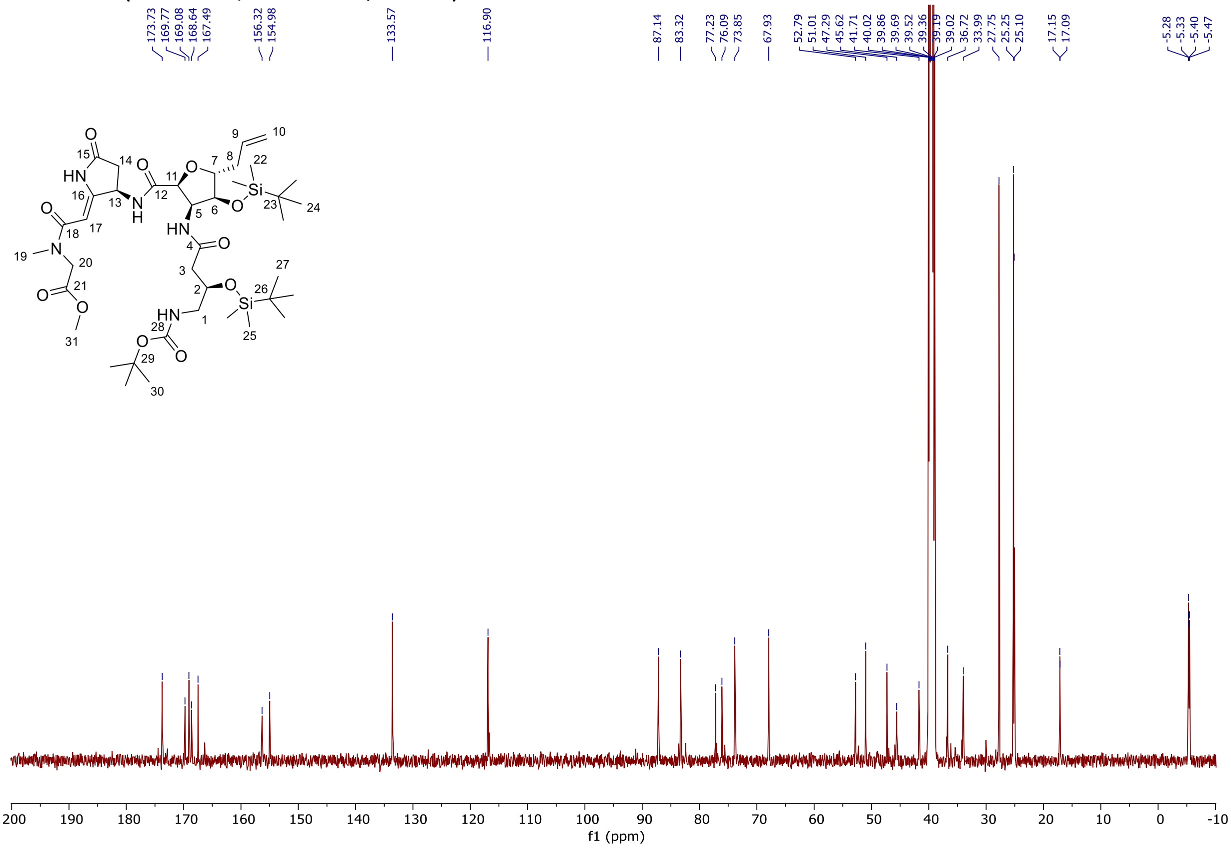

Methyl *N*-((*Z*)-2-((*R*)-3-((2*R*,3*R*,4*S*,5*S*)-5-allyl-3-((*R*)-4-((*tert*-butoxycarbonyl)amino)-3-((*tert*-butyldimethylsilyl)oxy)butanamido)-4-hydroxytetrahydrofuran-2-carboxamido)-5-oxopyrrolidin-2-ylidene)acetyl)-*N*-methylglycinate 22b

^{1}H-NMR (500 MHz, DMSO-d$_6$, 373 K):

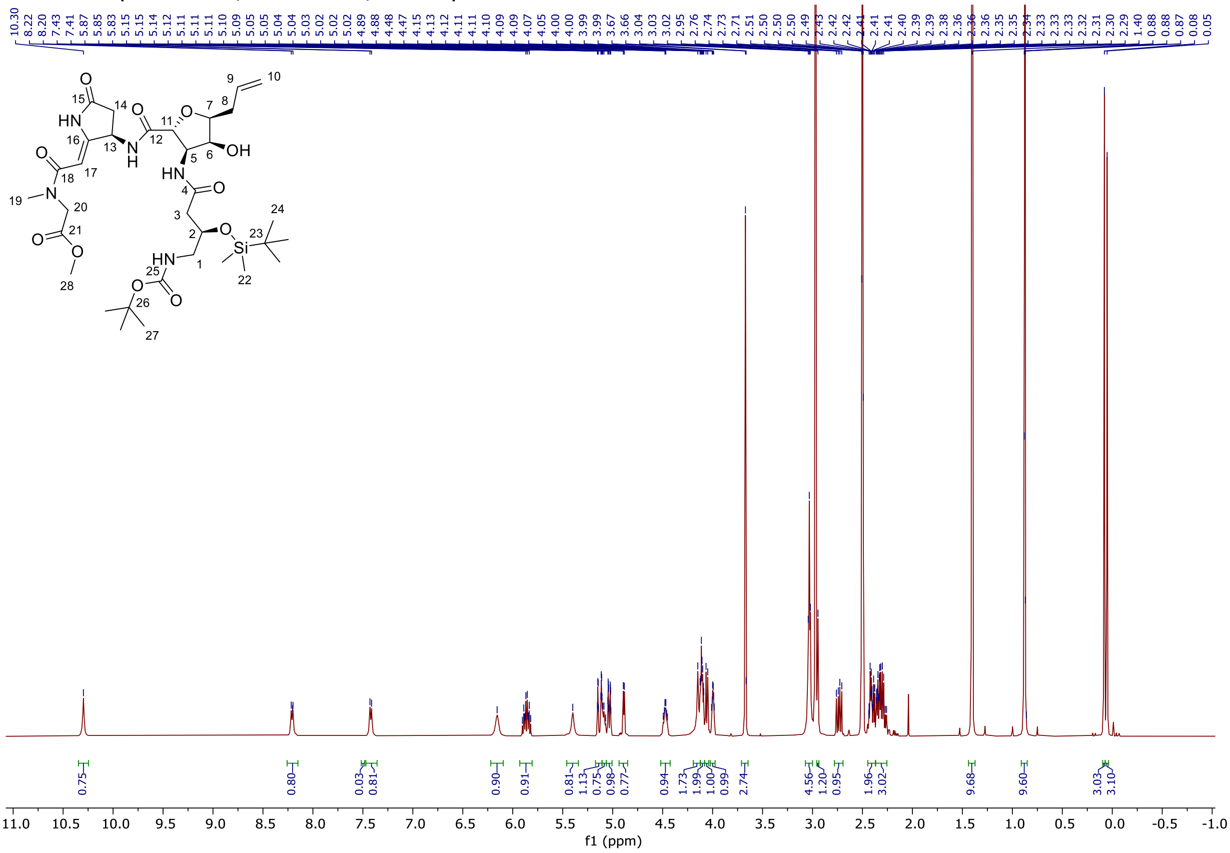

^{13}C-NMR (125 MHz, DMSO-d$_6$, 373 K):

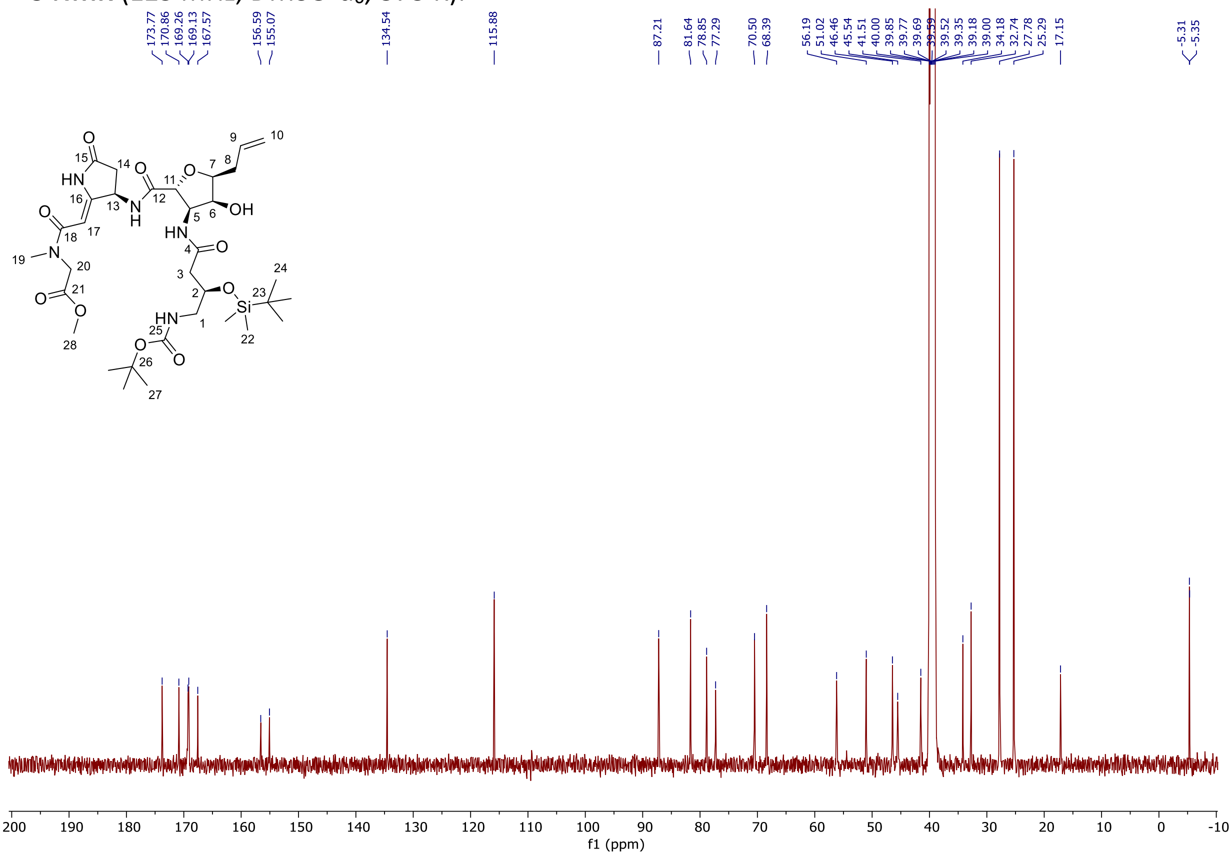

Methyl *N*-((*Z*)-2-((*R*)-3-((2*S*,3*S*,4*S*,5*R*)-5-allyl-3-((*R*)-4-((*tert*-butoxycarbonyl)amino)-3-((*tert*-butyldimethylsilyl)oxy)butanamido)-4-((*tert*-butyldimethylsilyl)oxy)tetrahydrofuran-2-carboxamido)-5-oxopyrrolidin-2-ylidene)acetyl)-*N*-methylglycyl-D-tryptophylglycinate 23c

^{1}H-NMR (500 MHz, DMSO-d$_6$):

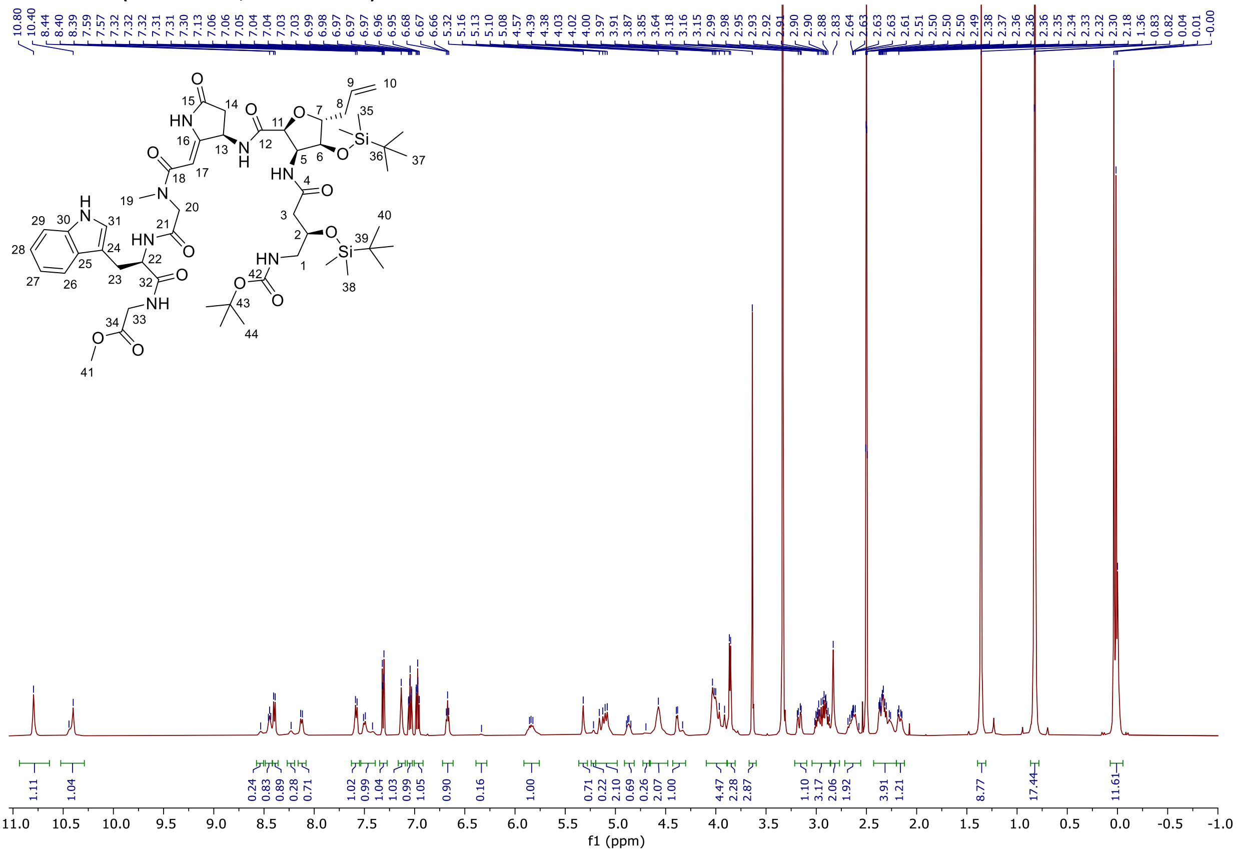

^{13}C-NMR (125 MHz, DMSO-d$_6$):

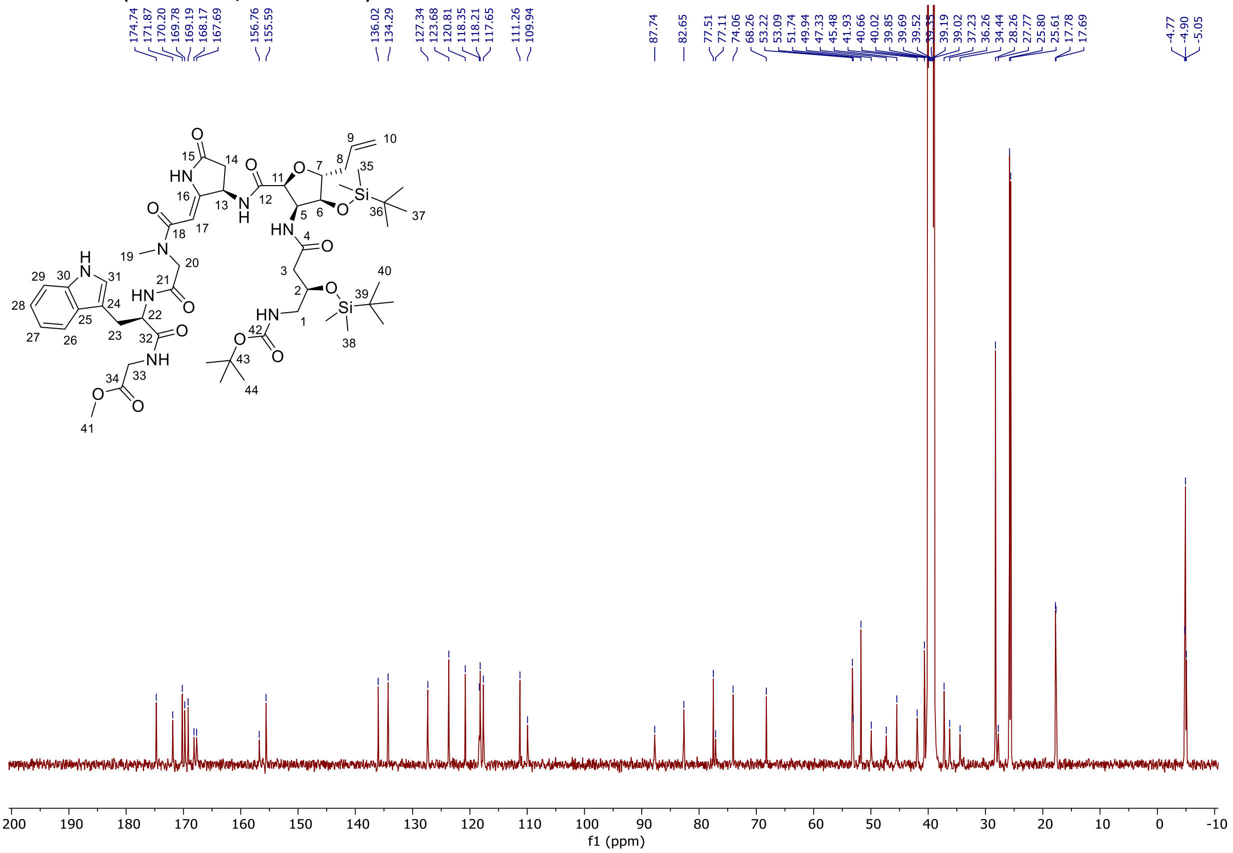

Methyl *N*-((*Z*)-2-((*R*)-3-((2*R*,3*R*,4*S*,5*S*)-5-allyl-3-((*R*)-4-((*tert*-butoxycarbonyl)amino)-3-((*tert*-butyldimethylsilyl)oxy)butanamido)-4-hydroxytetrahydrofuran-2-carboxamido)-5-oxopyrrolidin-2-ylidene)acetyl)-*N*-methylglycyl-D-tryptophylglycinate 23b

^{1}H-NMR (500 MHz, DMSO-d$_6$): 2wt% DCM as impurity

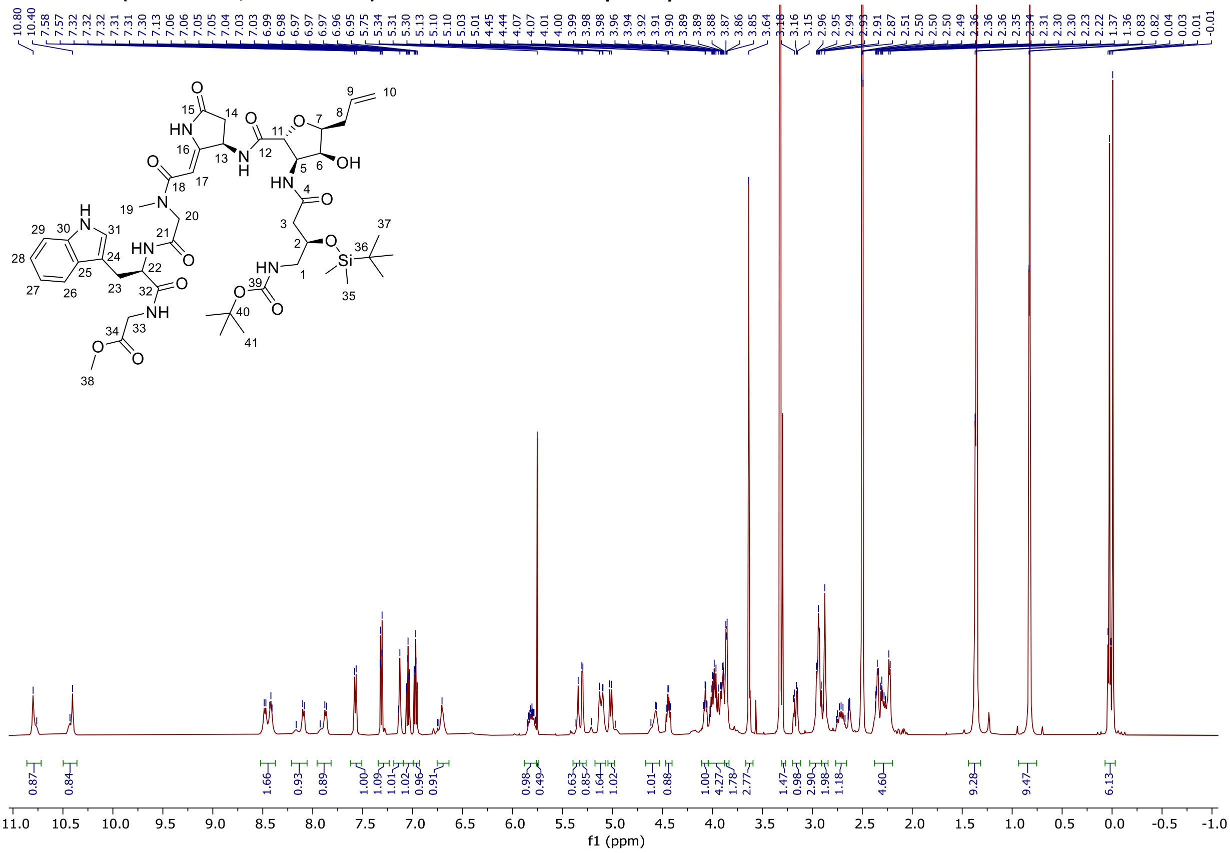

^{13}C-NMR (125 MHz, DMSO-d$_6$):

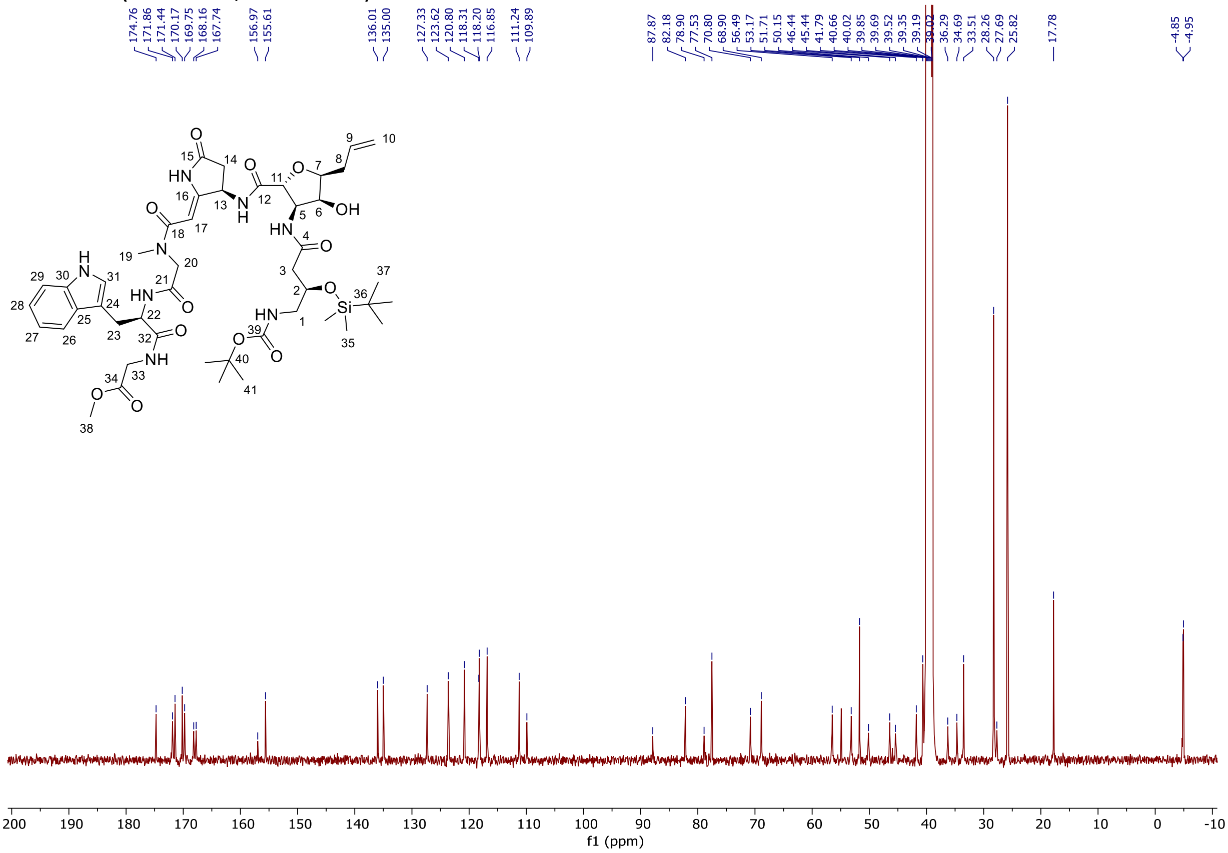

(2*R*,3*S*,3a*S*,7*R*,14*R*,23a*R*,25a*S*,*Z*)-14-((1*H*-Indol-3-yl)methyl)-2-allyl-3,7-bis((*tert*-butyldimethylsilyl)oxy)-18-methyl-2,3,3a,6,7,8,9,11,12,14,15,17,18,23a,24,25a-hexadecahydrofuro[2,3-q]pyrrolo[2,3-m][1,4,7,10,15,19]hexaazacyclotricosine-5,10,13,16,19,22,25(4*H*,21*H*,23*H*)-heptaone 25c

^{1}H-NMR (500 MHz, DMSO-d$_6$):

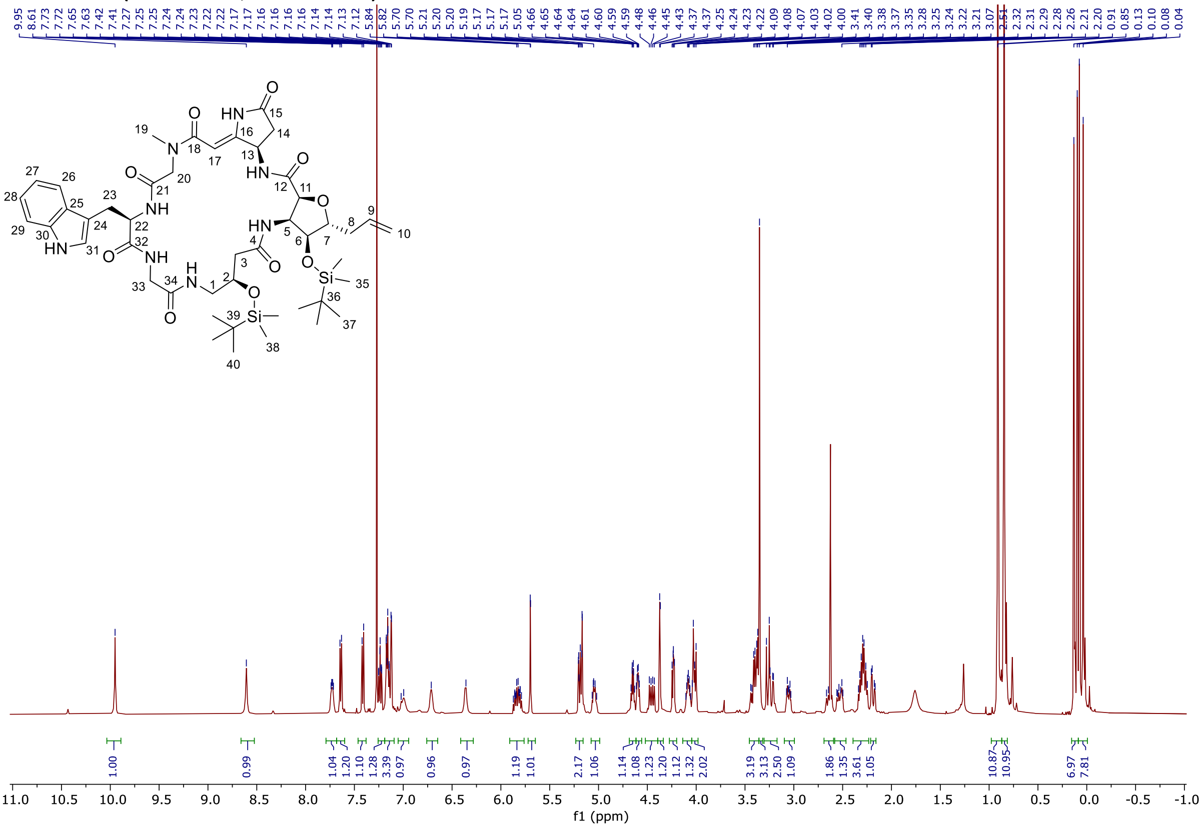

^{13}C-NMR (125 MHz, DMSO-d$_6$):

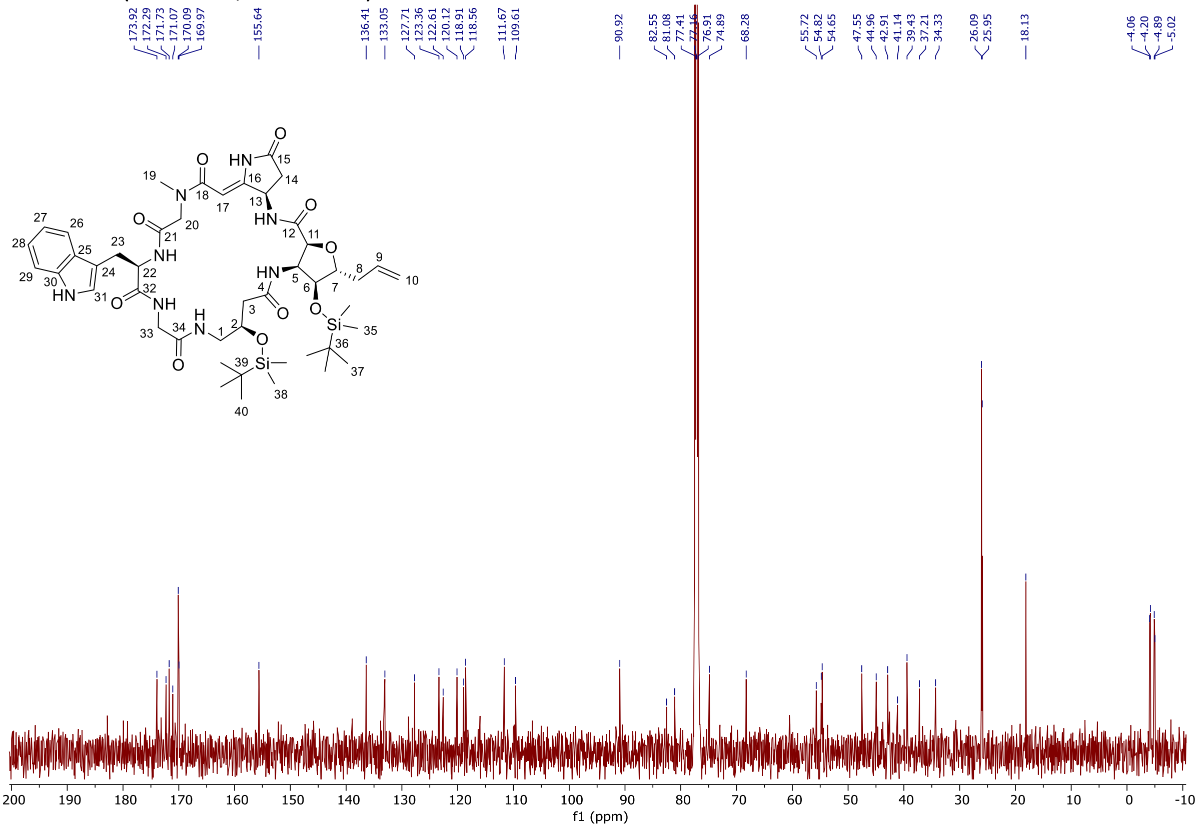

Simplified dehydrosocein 28c

^{1}H-NMR (500 MHz, MeOD-d$_4$): (2:1 rotamers)

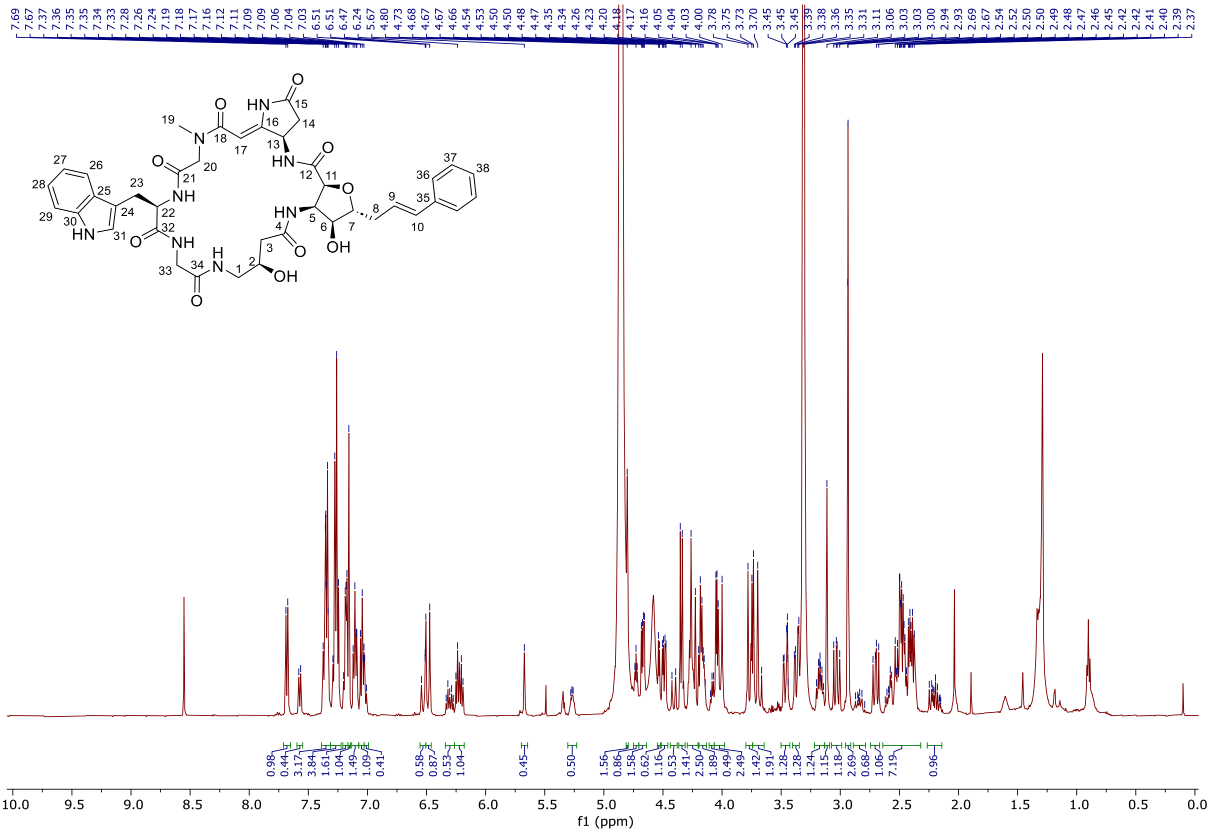

^{13}C-NMR (125 MHz, MeOD-d$_4$): (2:1 rotamers)

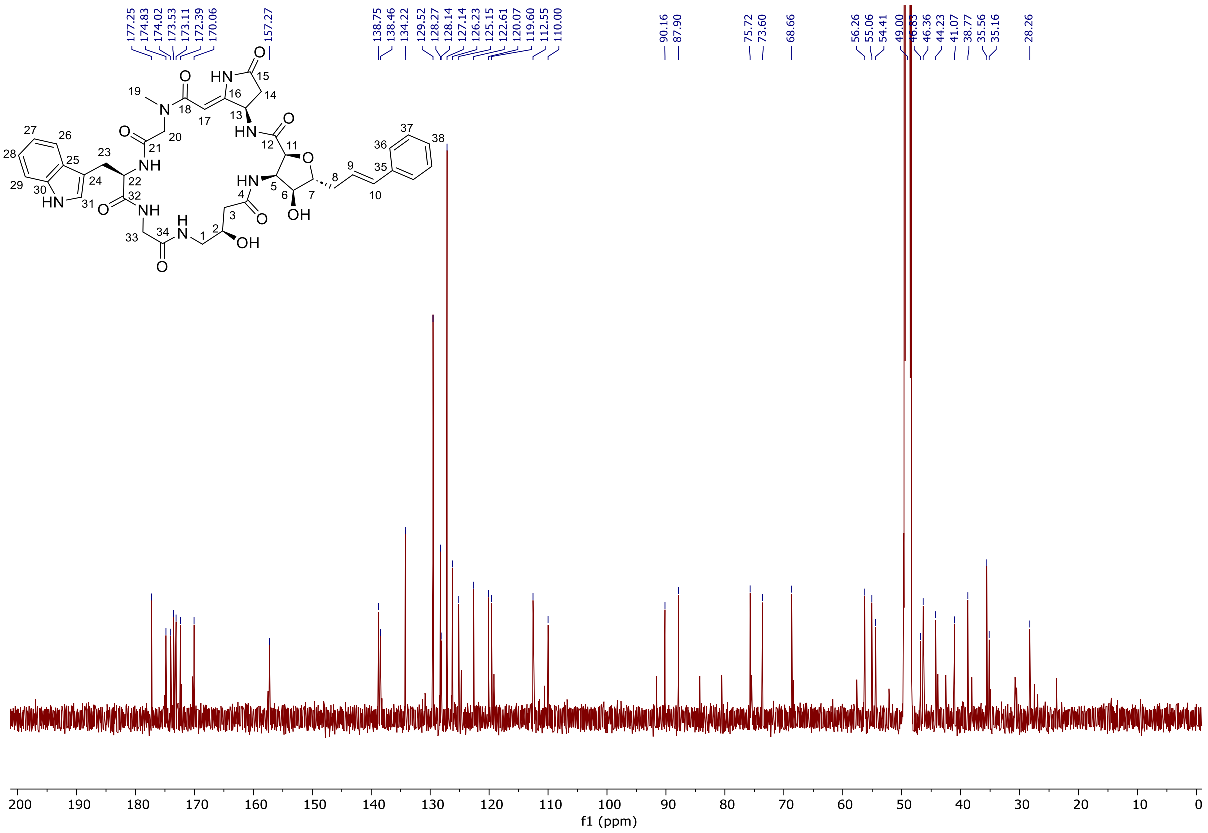

^{1}H-NMR (500 MHz, DMSO-d$_6$): (1:1 rotamers)

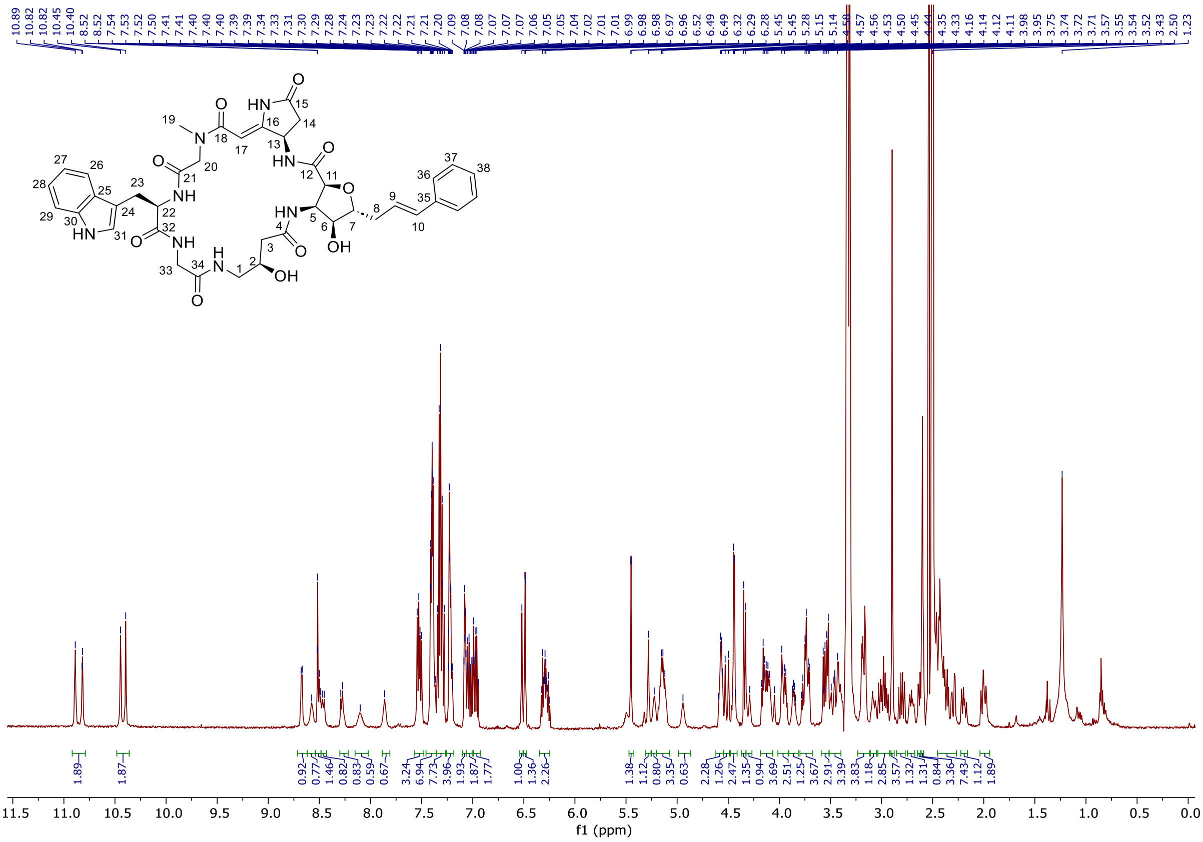

Simplified dehydrosocein 27c

^{1}H-NMR (500 MHz, MeOD-d$_4$): (2:1 rotamers)

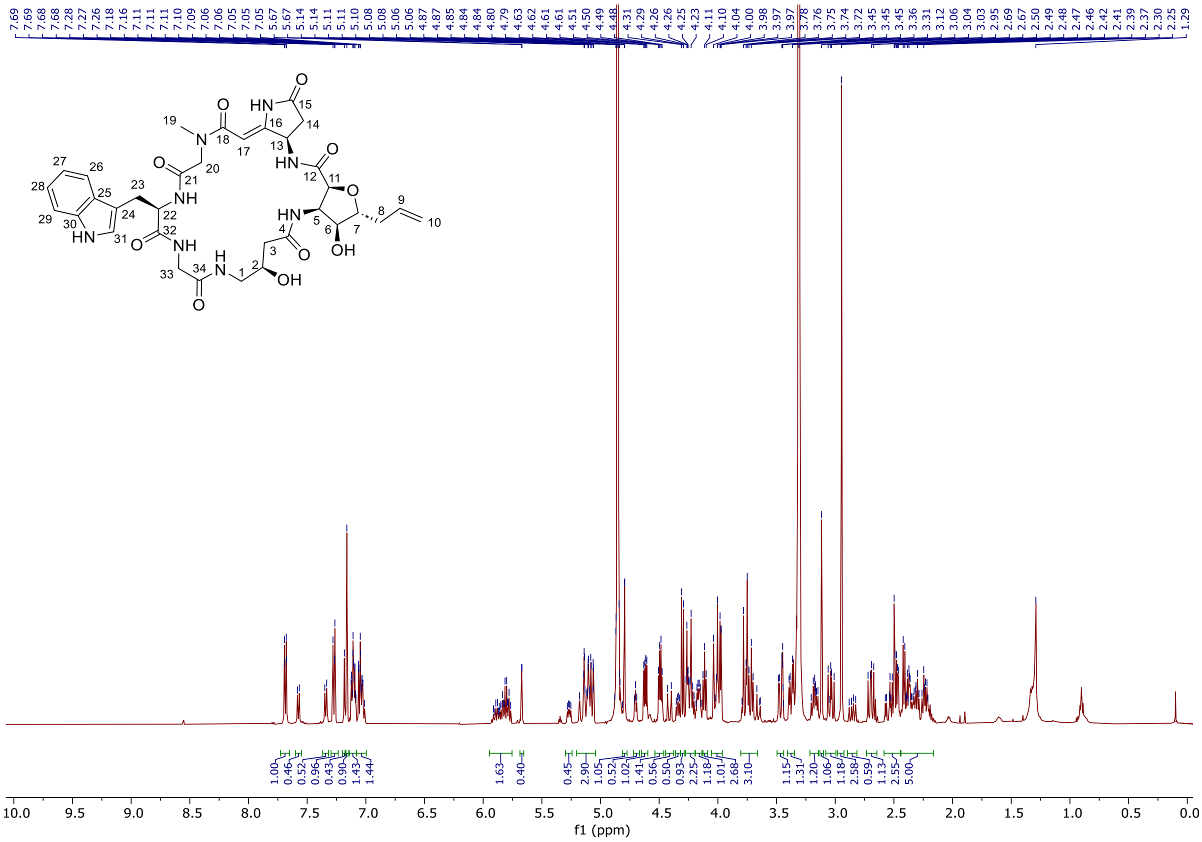

^{13}C-NMR (125 MHz, MeOD-d$_4$): (2:1 rotamers)

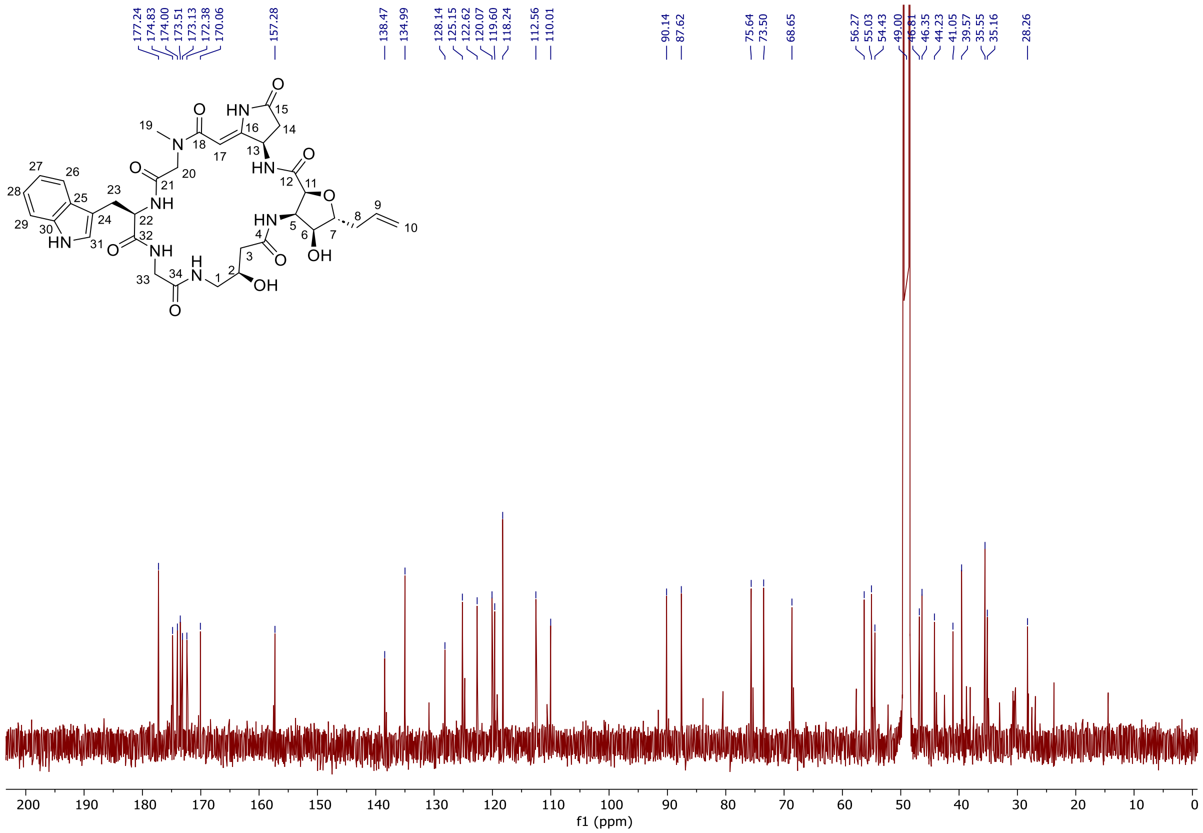

^{1}H-NMR (500 MHz, DMSO-d$_6$): (1:1 rotamers)

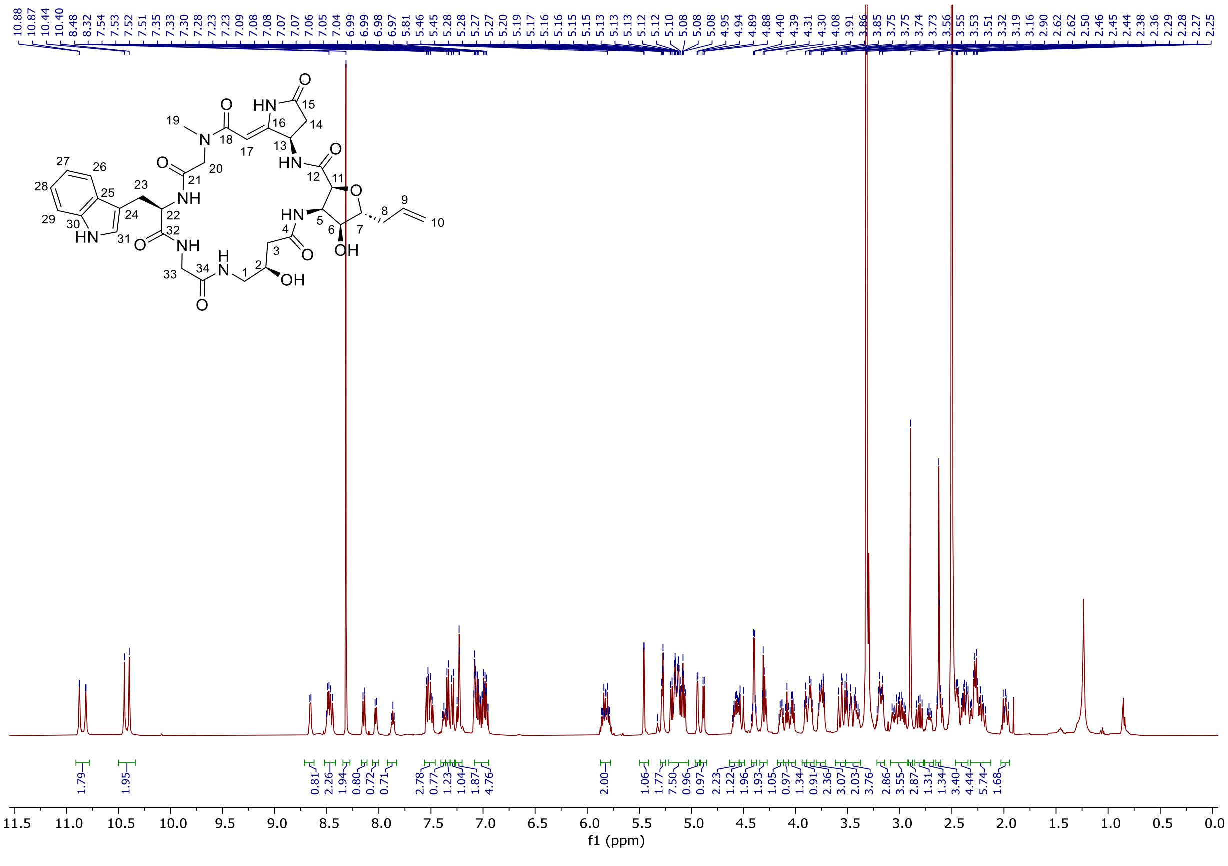

^{13}C-NMR (125 MHz, DMSO-d$_6$): (1:1 rotamers)

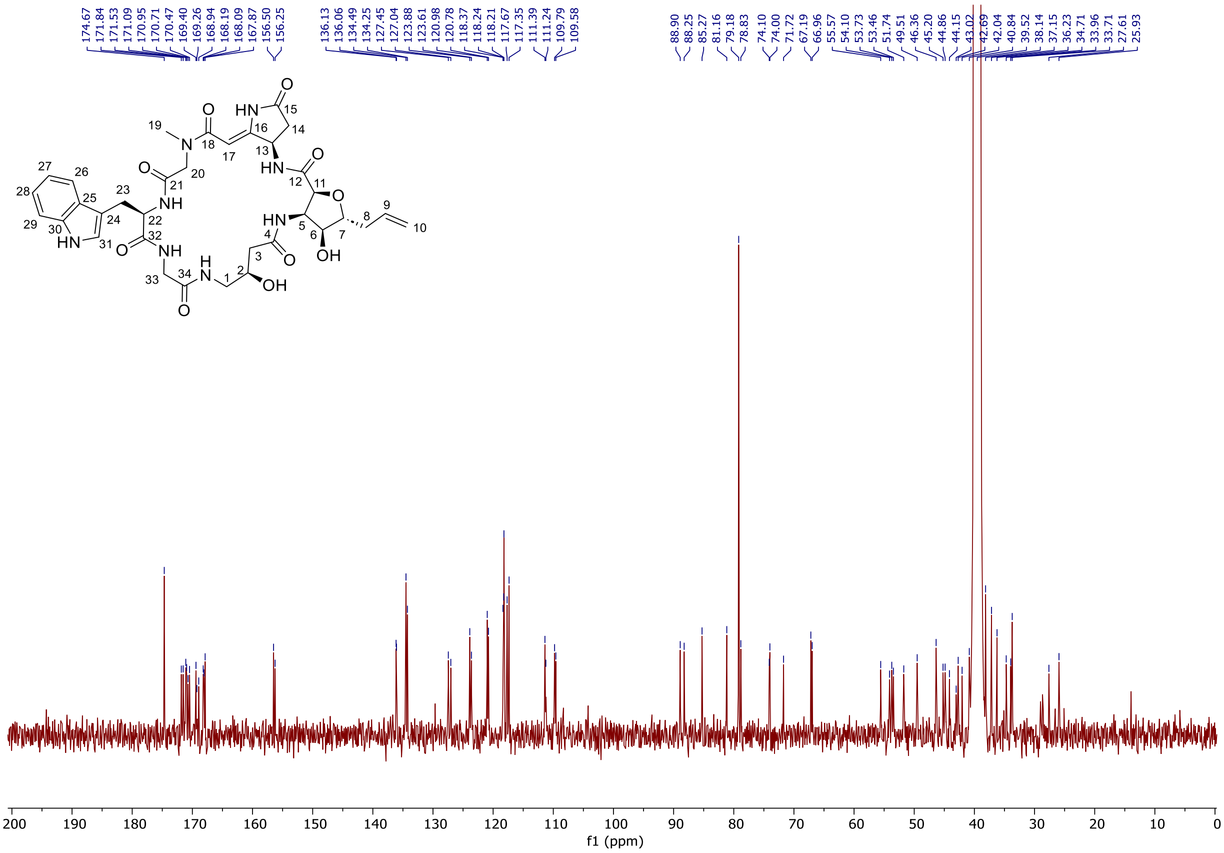

Simplified dehydrosocein 27b

^{1}H-NMR (500 MHz, DMSO-d$_6$): (rotamers 8:2)

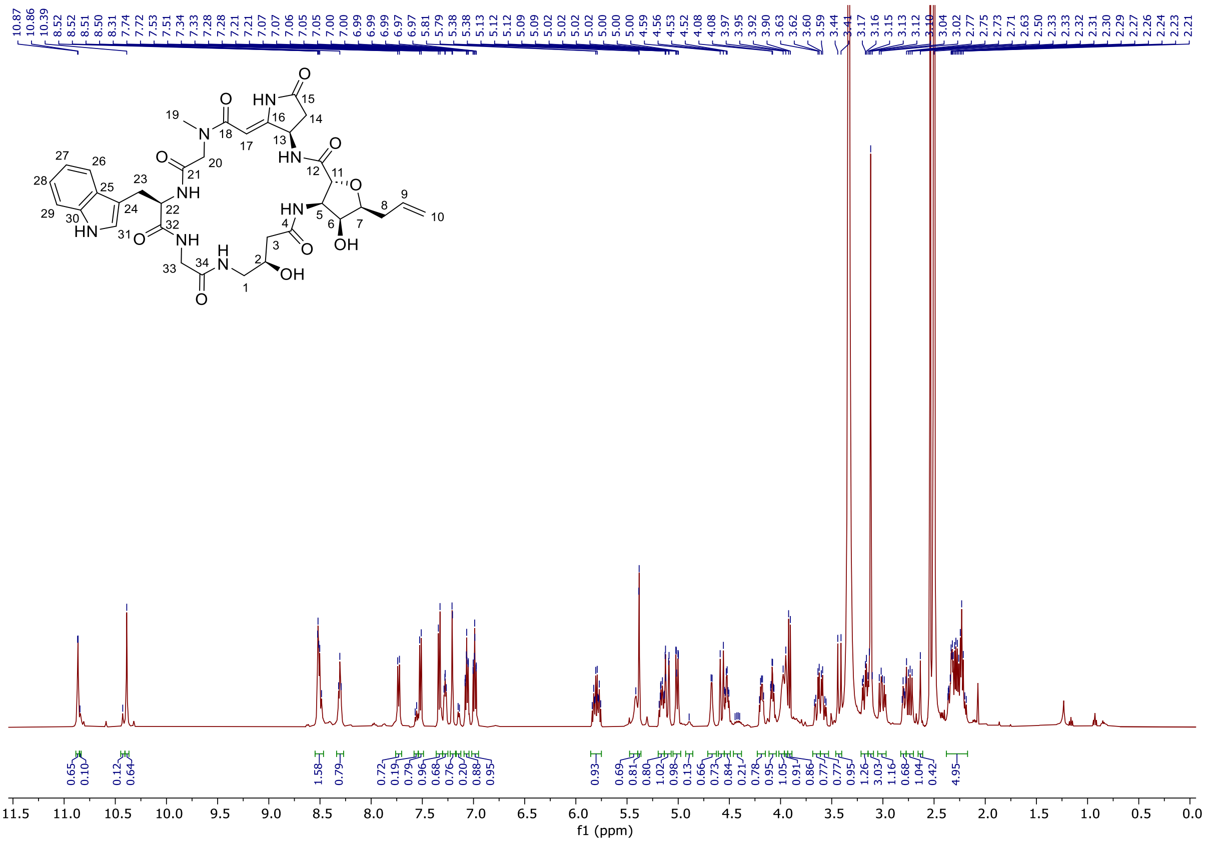

^{13}C-NMR (125 MHz, DMSO-d$_6$):

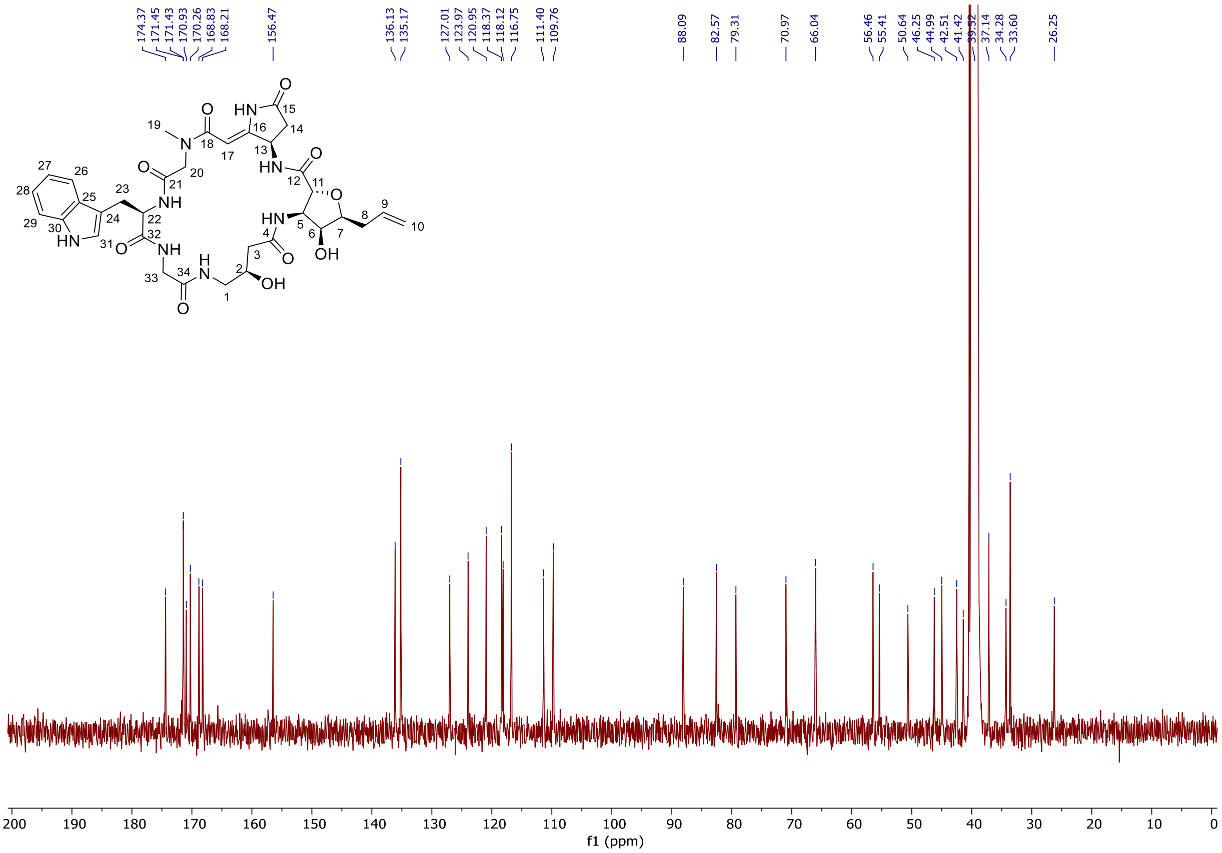

Simplified dehydrosocein 28b

^{1}H-NMR (500 MHz, DMSO-d$_6$):

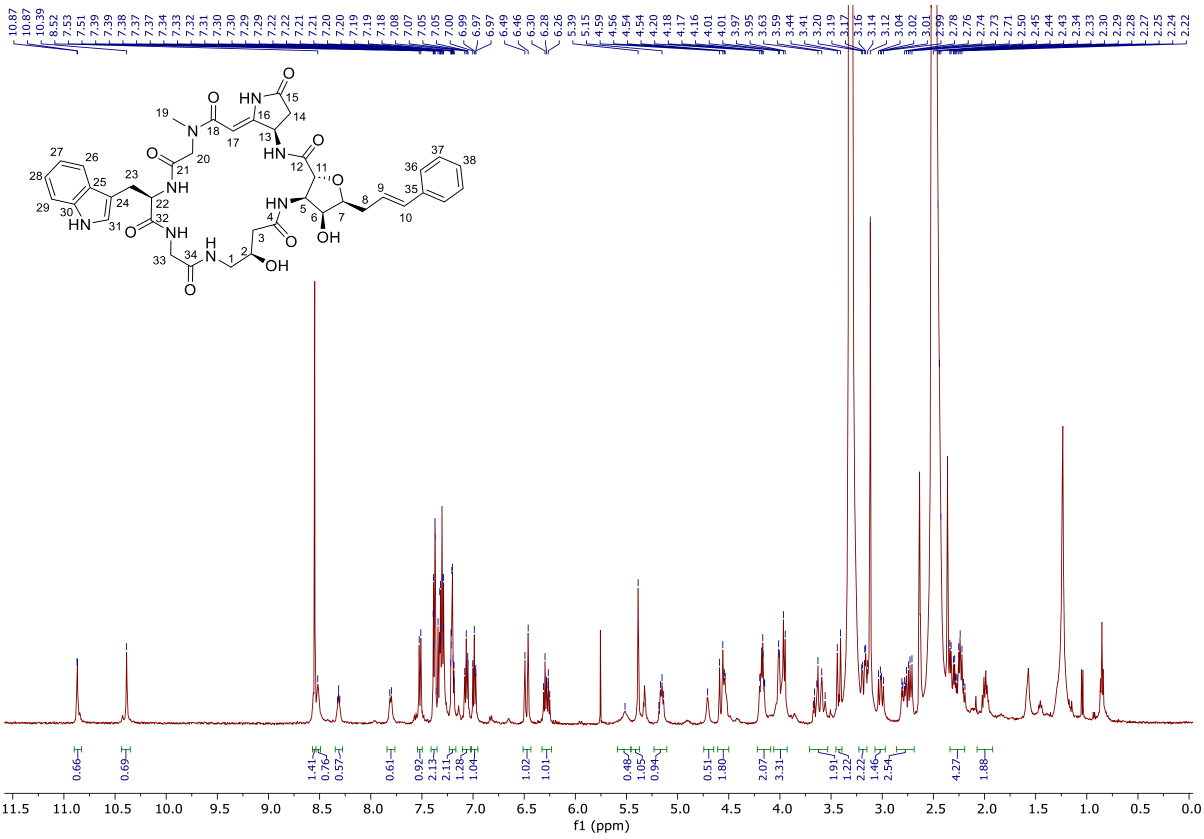

HSQCED-1D-projektion (125 MHz, DMSO-d$_6$):

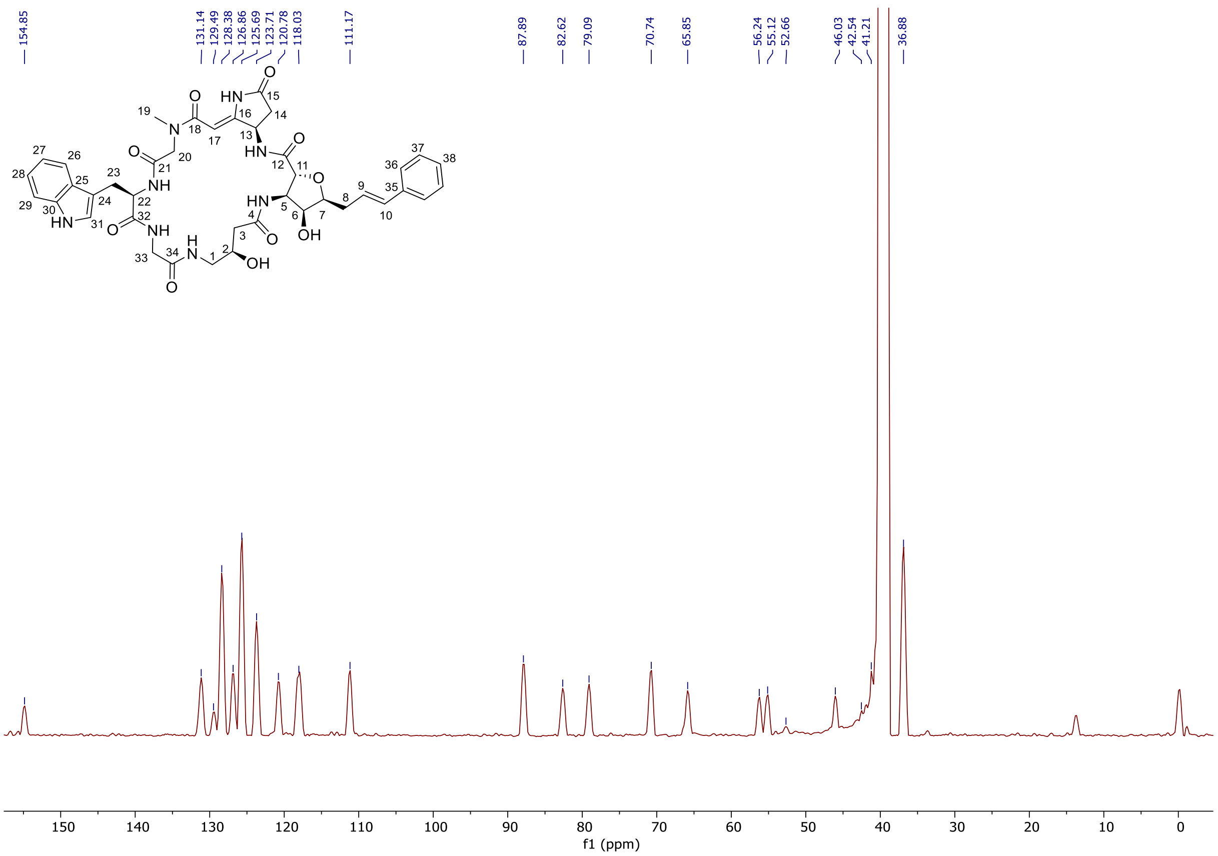

HMBC-1D-projektion (125 MHz, DMSO-d$_6$):

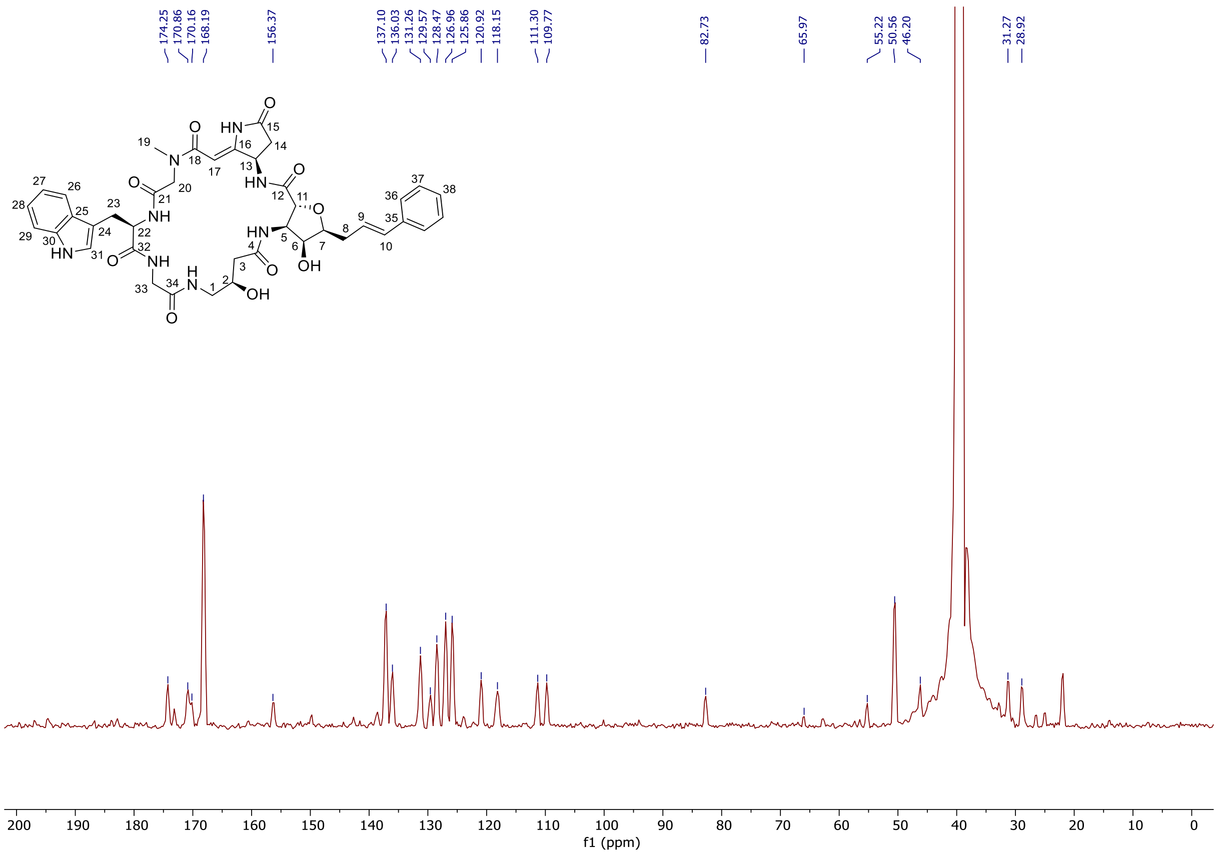

1.1 HPLC-Chromatograms of the Target Compounds

Simplified dehydrosocein 27a

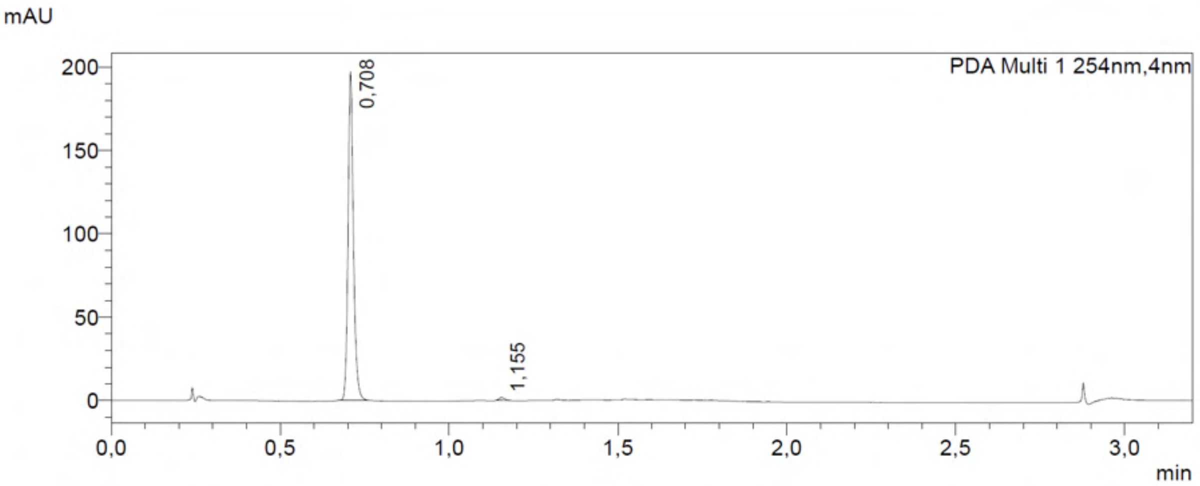

Peak Table

PDA Ch1 254nm

Peak#	Ret. Time	Area%
1	0,708	99,105
2	1,155	0,895
Total		100,000

Simplified dehydrosocein 28a

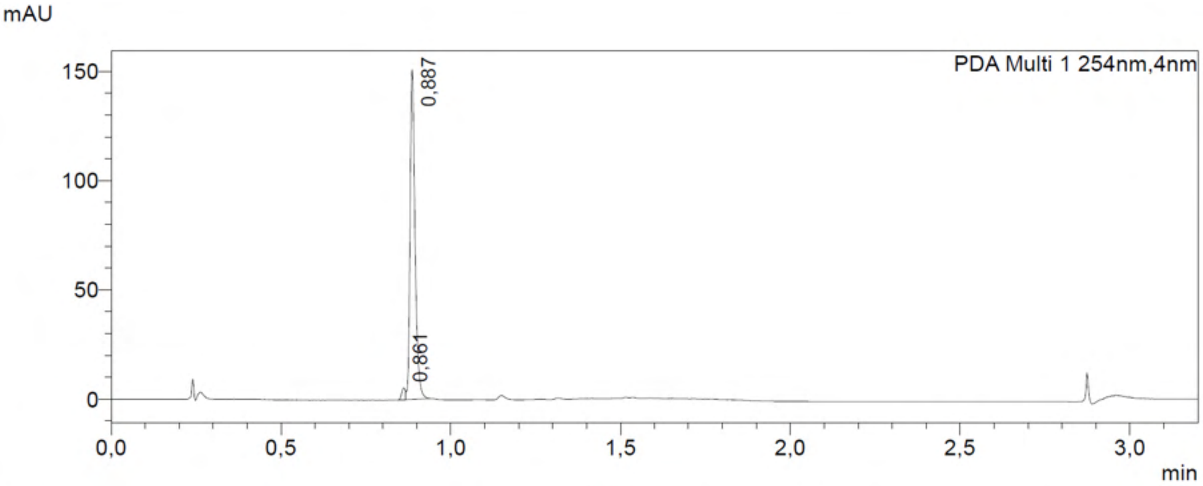

Peak Table

PDA Ch1 254nm

Peak#	Ret. Time	Area%
1	0,861	2,454
2	0,887	97,546
Total		100,000

Simplified dehydrosocein 28c

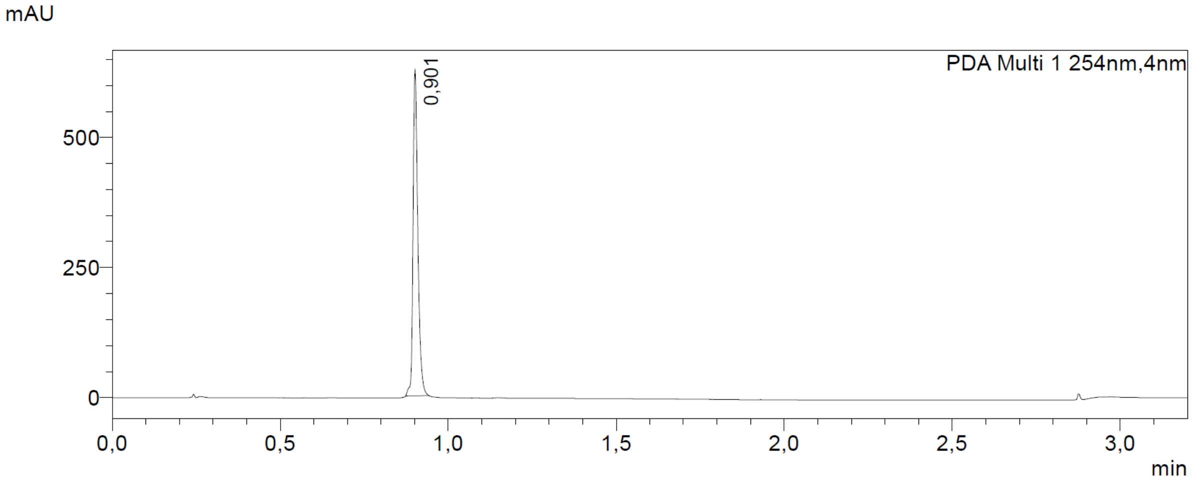

Peak Table

PDA Ch1 254nm

Peak#	Ret. Time	Area%
1	0,901	100,000
Total		100,000

Simplified dehydrosocein 27c

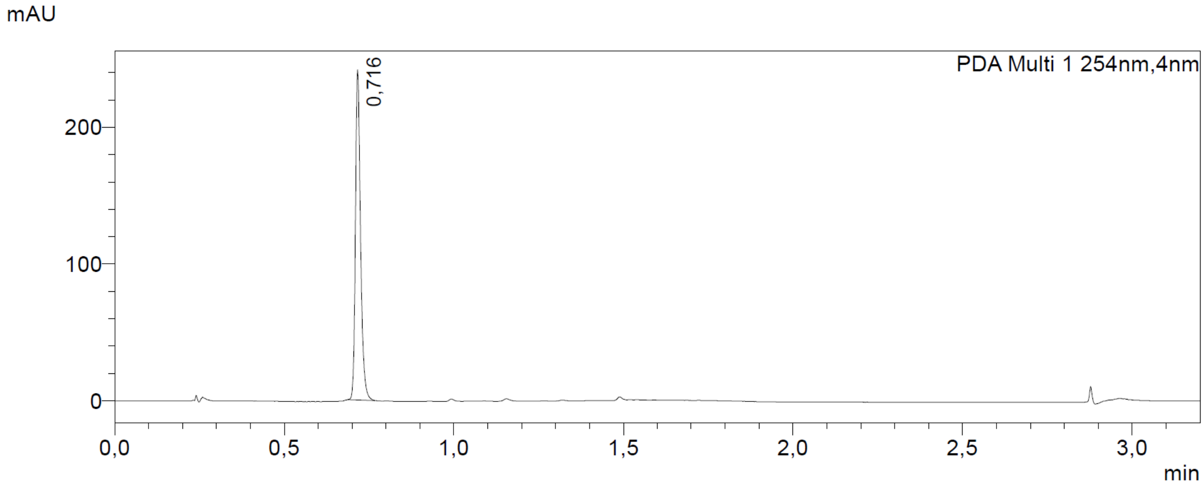

Peak Table

PDA Ch1 254nm

Peak#	Ret. Time	Area%
1	0,716	100,000
Total		100,000

Simplified dehydrosocein 27b

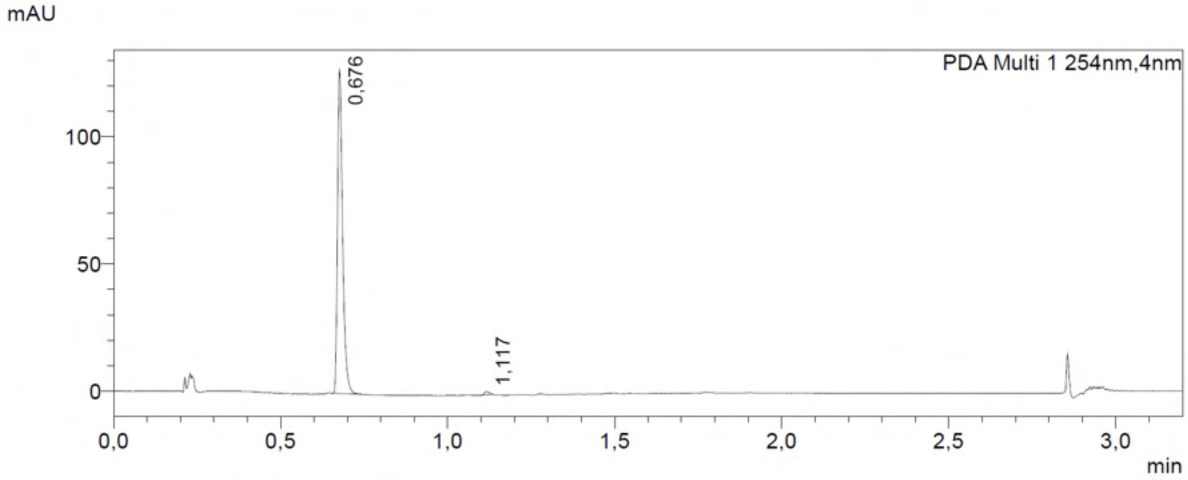

Peak Table

PDA Ch1 254nm

Peak#	Ret. Time	Area%
1	0,676	99,041
2	1,117	0,959
Total		100,000

Simplified dehydrosocein 28b

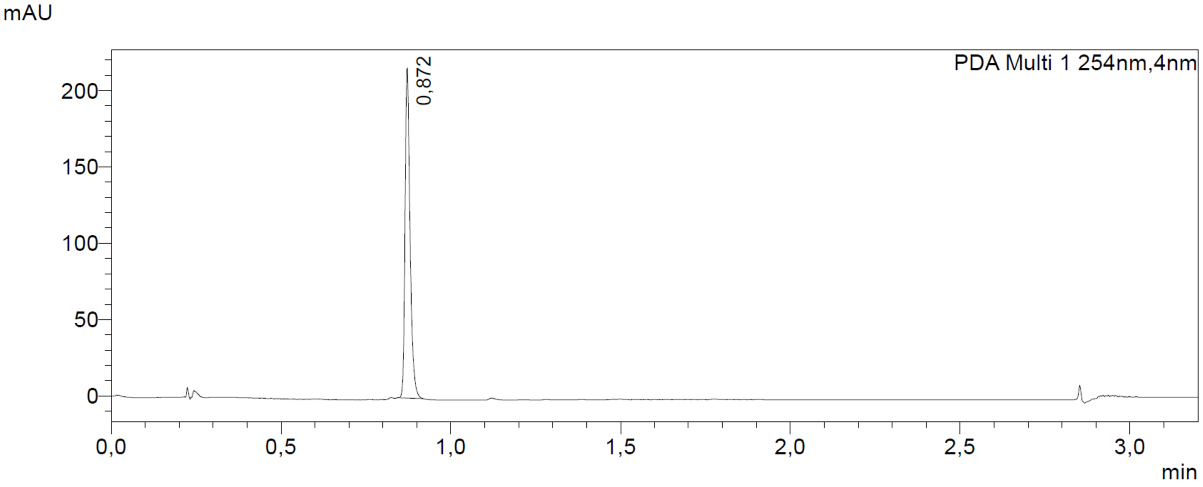

Peak Table

PDA Ch1 254nm

Peak#	Ret. Time	Area%
1	0,872	100,000
Total		100,000
